# Alkanes C_1_–C_6_ C–H Bond Activation via a Barrierless Potential Energy Path: Trifluoromethyl Carbenes Enhance Primary C–H Bond Functionalization

**DOI:** 10.1021/jacs.4c13065

**Published:** 2024-11-25

**Authors:** Jonathan Martínez-Laguna, Julia Altarejos, M. Ángeles Fuentes, Giuseppe Sciortino, Feliu Maseras, Javier Carreras, Ana Caballero, Pedro J. Pérez

**Affiliations:** † Departamento de Química and Laboratorio de Catálisis Homogénea, Unidad Asociada al CSIC, CIQSO-Centro de Investigación en Química Sostenible, Universidad de Huelva, Huelva 21007, Spain; ‡ Departamento de Química Orgánica y Química Inorgánica, Instituto de Investigación Química “Andrés M. del Río” (IQAR), Universidad de Alcalá, Alcalá de Henares, Madrid 28805, Spain; § The Barcelona Institute of Science and Technology, Institute of Chemical Research of Catalonia (ICIQ-CERCA), Avgda. Països Catalans, 16, Tarragona 43007, Spain; ∥ Departament de Química, Universitat Autònoma de Barcelona, Bellaterra 08193, Spain

## Abstract

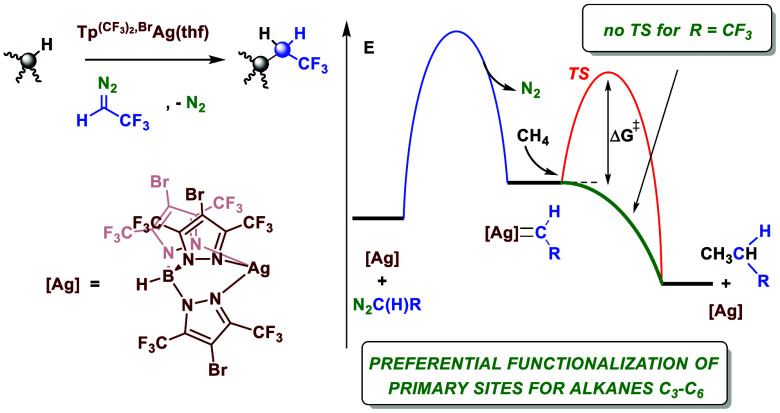

In this mixed computational and experimental study, we report a catalytic system for alkane C_1_–C_6_ functionalization in which the responsible step for C–H bond activation shows no barrier in the potential energy path. DFT modeling of three silver-based catalysts and four diazo compounds led to the conclusion that the Tp^F^Ag=C(H)CF_3_ (Tp^F^ = fluorinated trispyrazolylborate ligand) carbene intermediates interact with methane without a barrier in the potential energy surface, a prediction validated by experimentation using N_2_=C(H)CF_3_ as the carbene source. The array of alkanes from propane to *n*-hexane led to the preferential functionalization of the primary sites with unprecedented values of selectivity for an acceptor diazo compound. The lack of those barriers implies that selectivity can no longer be controlled by differences in the energy barriers. Molecular dynamics calculations (with propane as the model alkane) are consistent with the preferential functionalization of the primary sites due to a higher concentration of such C–H bonds in the vicinity of the carbenic carbon atom.

## Introduction

Either from natural deposits (natural or shale gas) or from oil refinement, alkanes are fundamental chemicals for society.^1,2^ For example, methane is currently the main source of syngas,^3^ whereas higher liquid alkanes are the main component of fuels. However, their use as starting materials for synthetic purposes is quite reduced, mainly due to their high bond dissociation energies^4^ and low polarities.^5^ Moreover, when different reaction sites are available in the alkane molecule, selectivity issues affect the reaction outcome, making the control of their reactivity far from simple and yet constituting a challenge.^6^

**Scheme 1:**
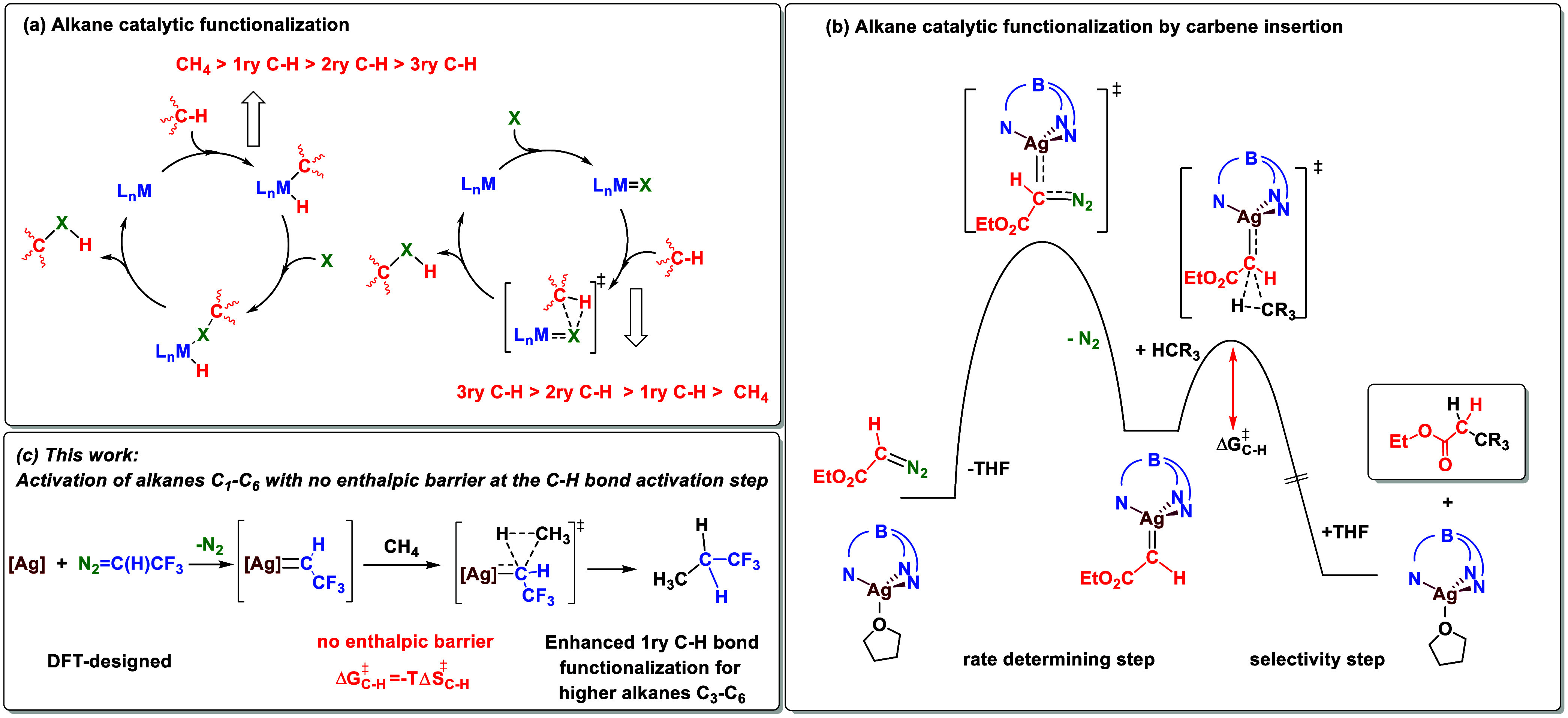
Alkane Catalytic Functionalization via Carbene Transfer

There are two general strategies for the modification of alkane C–H bonds through organometallic catalysis.^7^ On the one hand, mechanisms based on the activation of the alkane by the metal center provide a metal–carbon bond in the first stages, before functionalization takes place (Scheme 1a). Seminal work by Bergman demonstrated that the order of reactivity for this reaction is methane > primary (1ry) C–H > secondary (2ry) C–H > tertiary (3ry) C–H,^8^ i.e., the inverse of bond dissociation energy (BDE).^4^ This was explained as the result of the order of stability of the intermediate M–CR_3_. Unfortunately, being a more reactive primary site does not mean it can be better functionalized since the stability of the metal–alkyl intermediate makes the next step more difficult.

A second strategy consists in the functionalization of the C–H bond upon its interaction with the ligand of a highly electrophilic intermediate containing a moiety such as M = X, where X stands for a carbene, nitrene, or oxo ligands.^7^ In this case, the reactivity follows the trend 3ry C–H > 2ry C–H > 1ry C–H > methane, with some deviations depending on the nature of the M = X intermediate, which corresponds to that of the BDE.

We have been interested in the use of the latter strategy for alkane functionalization,^9^ particularly with methane and light alkanes,^10,11^ using group 11 metals to promote the insertion of a carbene group into the C–H bonds of those hydrocarbons. Given the less favored situation of the primary sites within this methodology,^12,13^ the preferential functionalization of primary sites still defies researchers, despite the outstanding development in this area promoted in the past decade.^14,15^

With the idea of developing catalysts for the preferential modification of primary sites in alkanes, we formulated the following strategy. Previous work from our group has shown^12^ that the modification of methane with silver catalysts, which transfer a carbene group from ethyl diazoacetate, displays a barrier of 8.1 kcal mol^–1^ (Scheme 1b) for the step in which methane is activated. Since that is the step in which the selectivity is decided when an alkane with two or more distinct reaction sites is available, the design of catalysts that display the lowest possible barrier for that step with methane should enhance the selectivity toward the primary sites for any alkane. The barriers for the activation of the C–H bonds of alkanes have enthalpic and entropic contributions, and due to electronic factors, the highest enthalpic barrier is that of methane. To decrease such an enthalpic barrier, we aimed at performing in silico evaluation of different catalysts and carbene precursors searching for the minimum activation energy possible for the methane activation step. DFT studies have led to the identification of a silver catalyst and a fluorinated diazo compound which provide an activation step with no enthalpic barrier for the C–H bond of methane (Scheme 1c). Subsequent experimental studies have shown that this in silico-designed catalytic system leads to unprecedented levels of functionalization of the primary C−H bonds (employing an acceptor carbene) for alkanes containing different reaction sites, which are also derived from barrierless steps along the pathway regulating this transformation. DFT and molecular dynamics (MD) studies provide an explanation for this novel behavior.

## Results and Discussion

### DFT Studies on Methane Functionalization by Different Catalyst and Carbene Precursors

The initial in silico screening was performed at the B3LYP-D3 level in a continuous solvent (Supporting Information). All computational results are available in the ioChem-BD repository and can be accessed via https://dx.doi.org/10.19061/iochem-bd-1-355.^16^ We considered several ligands which have already shown their capabilities to activate low reactive C–H bonds of alkanes such as [Tp^Br3^Ag]_2_,^17^ Tp^F27^Ag(thf),^12^ and Tp^(CF3)2,Br^Ag(thf)^11^ (Table 1). As carbene precursors, diazo compounds N_2_=C(H)CO_2_Et, N_2_=C(Ph)CO_2_Et, N_2_=C(*p*-C_6_H_4_–CF_3_)CO_2_CH_2_CF_3_, and N_2_=C(H)CF_3_ (**2**) were selected. We also computed the step from the precursor complex to the metallocarbene to confirm that the reaction was not hindered by the presence of the fluorine substituents (see the Supporting Information, Figures SC2 and SC3) and that the mechanistic picture of Scheme 1b was valid. Methane activation by carbene insertion has been analyzed by free energy calculations at the DFT B3LYP-D3 theory level, using *n*-hexane as an implicit solvent. The reaction starts with the formation of a supramolecular adduct between Tp^*x*^Ag=C(R^1^)R^2^ and an incoming CH_4_ molecule located above the separated reactants followed by CH_3_–H bond breaking and concerted H_3_C–CH(R^1^)R^2^ bond formation (Table 1). The data of the most favored transition states (TSs) are gathered in Table 1, while the complete series of all of the conformers is reported in Table SC1. It is worth mentioning that the high reactivity of these silver-carbene species has precluded their observation, even at low temperatures. However, with the less reactive copper Tp^*x*^Cu cores, several carbene complexes of composition Tp^*x*^Cu=C(R^1^)(R^2^) could be detected at low temperatures.^18^

**Table 1:**
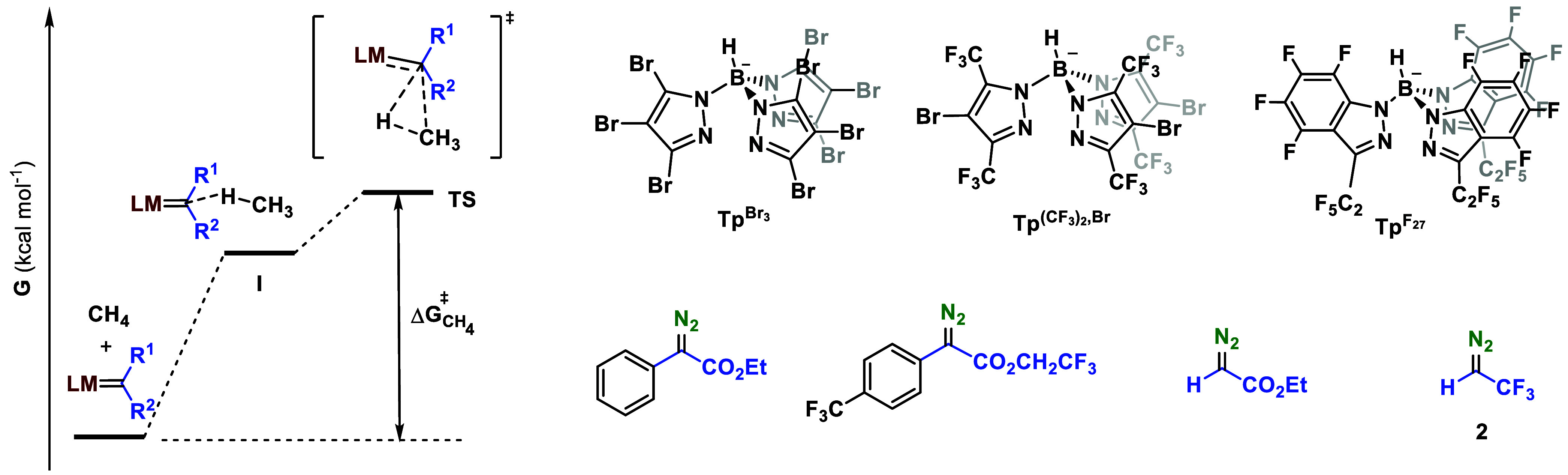
B3LYP-D3-Characterized TSs for CH_3_–H Activation by Tp^*x*^Ag=C(R^1^)R^2^ and (OAc)_4_Rh_2_=C(R^1^)R^2^ Carbenes

Entry	LM	R^1^	R^2^	Δ*G*^‡^CH_4_ (kcal mol^–1^)^a^
1	Tp^Br3^Ag	Ph	CO_2_Et	26.8^b^
2	Tp^(CF3),2Br^Ag	Ph	CO_2_Et	28.3^c^
3	Tp^F27^Ag	Ph	CO_2_Et	26.8^c^
4	Rh_2_(OAc)_4_	Ph	CO_2_Et	32.5^c^
5	Tp^Br3^Ag	*p*-C_6_H_4_–CF_3_	CO_2_CH_2_CF_3_	24.3^c^
6	Tp^(CF3),2Br^Ag	*p*-C_6_H_4_–CF_3_	CO_2_CH_2_CF_3_	22.0^c^
7	Tp^F27^Ag	*p*-C_6_H_4_–CF_3_	CO_2_CH_2_CF_3_	22.8^c^
8	Rh_2_(OAc)_4_	*p*-C_6_H_4_–CF_3_	CO_2_CH_2_CF_3_	26.3^c^
9	Tp^Br3^Ag	H	CO_2_Et	10.7^c^
10	Tp^(CF3),2Br^Ag	H	CO_2_Et	9.1^c^
11	Tp^F27^Ag	H	CO_2_Et	8.1^c^
12	Rh_2_(OAc)_4_	H	CO_2_Et	15.7^c^
13	Tp^Br3^Ag	H	CF_3_	6.6
14	Tp^(CF3),2Br^Ag	H	CF_3_	**No TS**
15	Tp^F27^Ag	H	CF_3_	**No TS**
16	Rh_2_(OAc)_4_	H	CF_3_	15.3

a Continuum solvation model for *n*-hexane.

b C–H activation with the methane incoming from the ester face containing the carbonyl oxygen.

c C–H activation with the methane incoming from the ester face containing the −OR group.

The barriers are generally within a 2 kcal mol^–1^ interval for the three silver Tp^*x*^Ag cores when using diazoacetates, either with aryl (entries 1–3 and 5–7) or just hydrogen substituents (entries 9–11). Notably, the latter values of 8.1–10.7 kcal mol^–1^ are significantly lower than those for the donor–acceptor diazo compounds (22.0–28.3 kcal mol^–1^), highlighting the substantial effect of the enhanced electrophilic character at the carbene carbon atom. The fourth diazo compound (**2**) bearing H and CF_3_ substituents^19^ provides the lowest values for the computed barriers. With the Tp^Br3^Ag complex, a 6.6 kcal mol^–1^ barrier has been calculated, whereas, remarkably, no TSs could be found when using the Tp^F27^Ag and Tp^(CF3)2,Br^Ag cores. For the sake of comparison, dirhodium tetraacetate was also computed (entries 4, 8, 12, and 16) with the four diazo compounds, in all cases the barriers being higher than those found for silver-based catalysts, even for the CF_3_-containing diazo reagent.

The disappearance of the barrier for the Tp^F27^Ag and Tp^(CF3)2,Br^Ag cores is easier to understand by realizing that the TS is preceded in all cases by an intermediate with a free energy above the reactants (**I**, Table 1). For instance, for entry 10 in the table, intermediate **I** is 6.6 kcal mol^–1^ above reactants, and the corresponding values for entries 11 and 13 are 6.0 and 5.7 kcal mol^–1^, respectively. This means that the separation between the intermediate and TS in these three cases is 2.5, 2.1, and 0.9 kcal mol^–1^, respectively. It is thus completely reasonable to expect this difference to become negative for entries 14 and 15. This brings an important qualitative consequence: once the TS gets below the intermediate, both structures disappear from the potential energy surface.

**Scheme 2:**
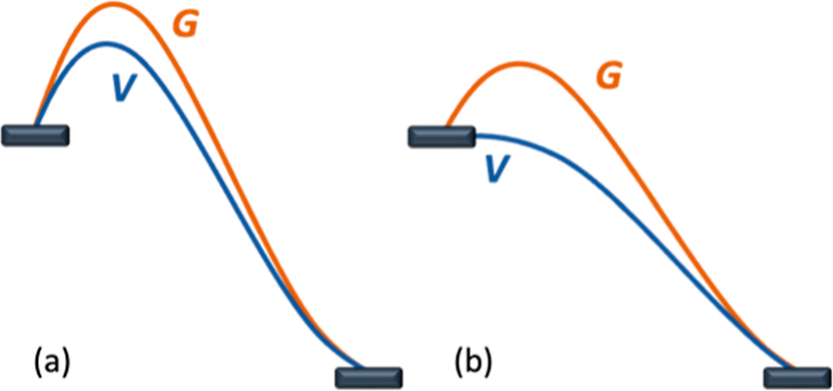
Energy Profiles for Bimolecular Reactions: (a) Usual, with Transition State, (b) Unusual, with No TS

The lack of a TS does not mean that the bimolecular process is completely barrierless as a purely entropic barrier remains in the Gibbs energy (Scheme 2). The concept of TS is associated with a barrier in the potential energy (V), roughly associated with enthalpy. When such a barrier exists, the usual procedure in computational chemistry is the calculation of a free energy (G) correction on this structure, which is then used as the free energy barrier. The absence of a barrier in potential energy precludes the direct application of this treatment. This is not critical in terms of reaction kinetics as the step will be very fast. However, the absence of an enthalpic barrier has important consequences. The first of them is that the rate will not be affected by any intrinsic electronic property of methane hindering its reactivity. The second one is that there is no selectivity control ruled by the relative stability of the now absent TSs.

**Scheme 3:**
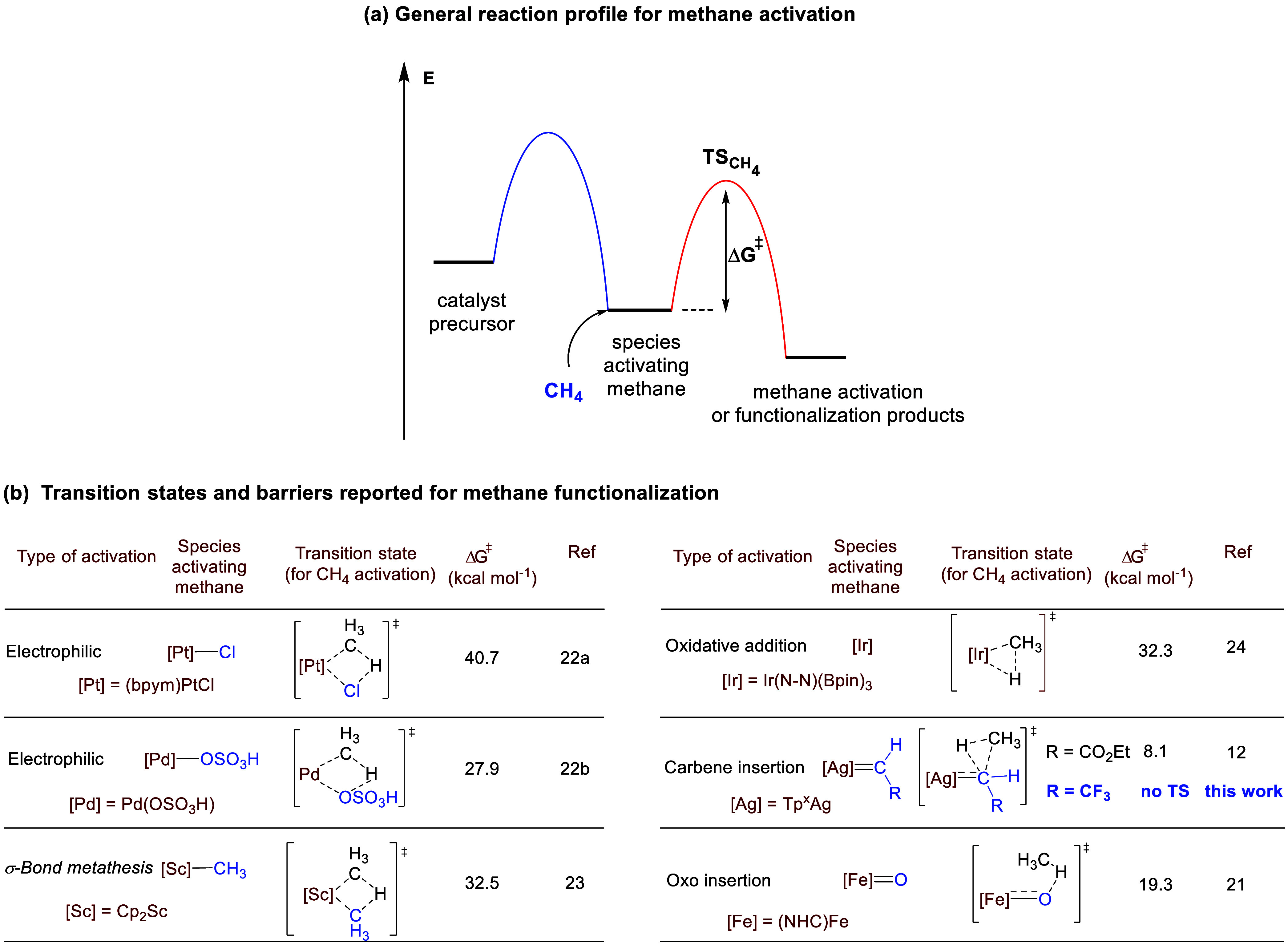
Reported Barriers for the Methane Activation Step in Catalytic Systems

The lack of a TS for the step in which methane is activated by the organometallic species is at variance with the previous reports for methane catalytic activation and functionalization. To date, the carbon–hydrogen bonds of methane have been catalytically converted into C–O, C–C, C–Si, C–N, and C–B bonds,^20^ in processes where methane interacts either with the metal center or with a ligand. Among the former, the activation step of the methane C–H bond takes place following electrophilic, σ-bond metathesis or oxidative addition steps, whereas the latter is known by concerted insertion of a carbene ligand^10^ or the stepwise (hydrogen atom transfer and rebound) process of a metal-oxo and the C–H bond.^21^ Usually, the active species that is responsible for methane activation is generated from a catalyst precursor with the corresponding barrier(s) (Scheme 3a). Then, such species interacts with methane in the first step of the methane functionalization pathway. DFT studies have previously provided Δ*G*^‡^ values for the steps in which methane is activated. As shown in Scheme 3b, the activation energies differ for distinct activation modes. The electrophilic activation computed for the catalytic system, based on (bpym)PtCl_2_, led to a barrier of 40.7 kcal mol^−1^,^22a^ whereas Pd-based catalysts reached 27.9 kcal mol^–1^ in the addition of CH_4_ to Pd(HSO_4_)_2_ to give Pd(HSO_4_)(CH_3_)(H_2_SO_4_).^22b^ In the case of scandium-catalyzed methane functionalization, which occurs through σ-bond metathesis, calculations for the reaction of Cp_2_Sc-CH_3_ with methane led to barriers of 32.5 kcal mol^–1^.^23^ Iridium-catalyzed methane borylation occurs through an oxidative addition step,^24^ for which a barrier of 32.3 kcal mol^–1^ has been calculated. Regarding the ligand-based functionalization pathways, the functionalization of methane by concerted carbene insertion displays an 8.1 kcal mol^–1^ activation barrier,^12^ whereas the methane activation step for the oxidation of the C–H bond presents a barrier of 19.3 kcal mol^–1^.^21^

### Light Alkane Functionalization Using Trifluorodiazoethane

**Scheme 4:**
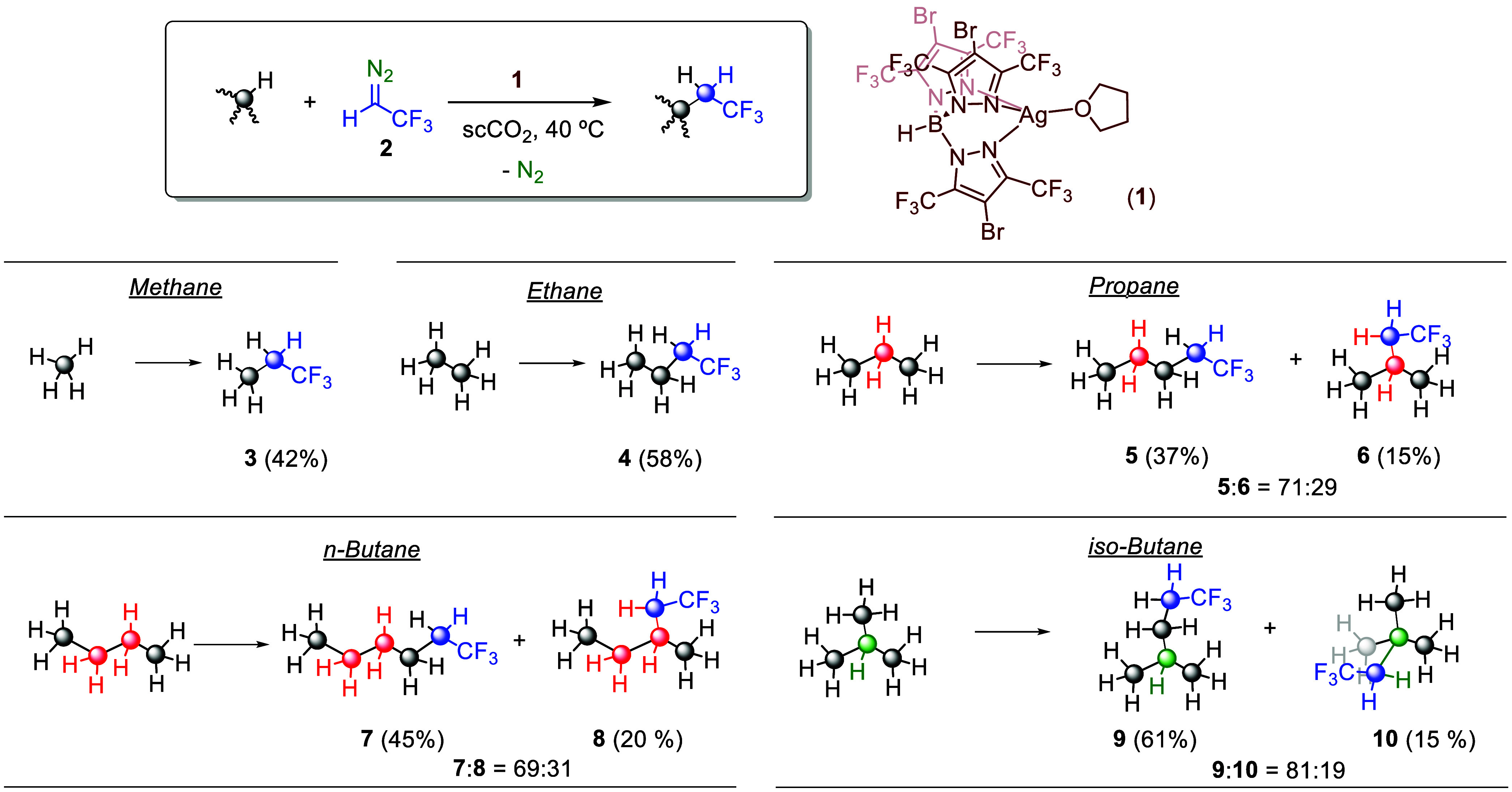
Methane and C_2_–C_4_ Gaseous Alkanes Conversion into 1,1,1,-Trifluoromethylalkanes Using Silver Catalyst 1 and Trifluorodiazoethane (TFDE) as the Carbene Source Reaction conditions: 0.01 mmol of catalyst **1**, 1 mmol of TFDE. Relative [cat]:[TFDE]:[alkane] ratios are 1:100:31776 for methane, 1:100:7075 for ethane, 1:100:1738 for propane, 1:100:267 for butane, and 1:100:399 for isobutane. See the Supporting Information for details.

The above predictions about a quite favored methane functionalization by silver-catalyzed carbene transfer using 2,2,2-trifluorodiazoethane (TFDE, N_2_=C(H)CF_3_, **2**) led us to evaluate such a transformation in the laboratory. We employed supercritical carbon dioxide (scCO_2_) as the reaction medium^10,11^ since this strategy leads to a homogeneous fluid single phase formed by methane and CO_2_, with no other potentially reactive C–H bond competing for the silver-carbene intermediates. Tp^(CF3)2,Br^Ag(thf) (**1**) was chosen as a catalyst, given its higher availability from a synthetic point of view compared to that of Tp^F27^Ag(thf). As shown in Scheme 4, methane was converted into 1,1,1-trifluoropropane (**3**) in a 42% yield (diazo-based) with that silver catalyst. Extension to the other four C_2_–C_4_ gaseous alkanes afforded the corresponding fluorinated alkanes. Ethane was converted into 1,1,1-trifluorobutane (**4**) in a 58% yield, whereas for those displaying two different reaction sites (propane, *n*-butane, and *i*-butane), mixtures derived from the insertion of the carbene at both available positions were obtained. Propane led to **5** and **6** in a 71:29, 1ry:2ry insertion ratio (52% yield), similar to *n*-butane (**7** and **8**, 69:31, 65% yield). The latter could also be functionalized using liquid butane as the solvent, with the selectivity being maintained. Isobutane was converted into an 81:19 ratio of 1ry:3ry insertion products (**9** and **10**, 76% yield). Catalyst **1** was employed in 1 mol % relative to TFDE, with the alkane being in the range 267–31776-fold excess relative to the catalyst (see Scheme 4 and Supporting Information for details).

Two general comments can be made from the results contained in Scheme 4. On the one hand, a comparison of these yields with those previously reported by our group and by the group of Mindiola^25^ employing ethyl diazoacetate (EDA) shows that the system reported herein is more active toward light alkane functionalization. This must be related to the generation of silver carbenes with exceptional electrophilicity. On the other hand, the percentage of products derived from the primary C–H bond functionalization has notably increased with this new system employing TFDE. For example, propane gives a 71:29, 1ry:2ry ratio of products with TFDE, whereas when using ethyl diazoacetate and catalyst **1**, it gave 44:56^12^ and Mindiola’s catalyst provided 17:83. It is also worth mentioning that this is the first example of a metal-catalyzed system for methane and light alkanes being converted into the corresponding trifluoroalkanes.^26−29^

### Regioselectivity in Alkane Functionalization: Influence of the Catalyst and Diazo Compound

**Scheme 5:**
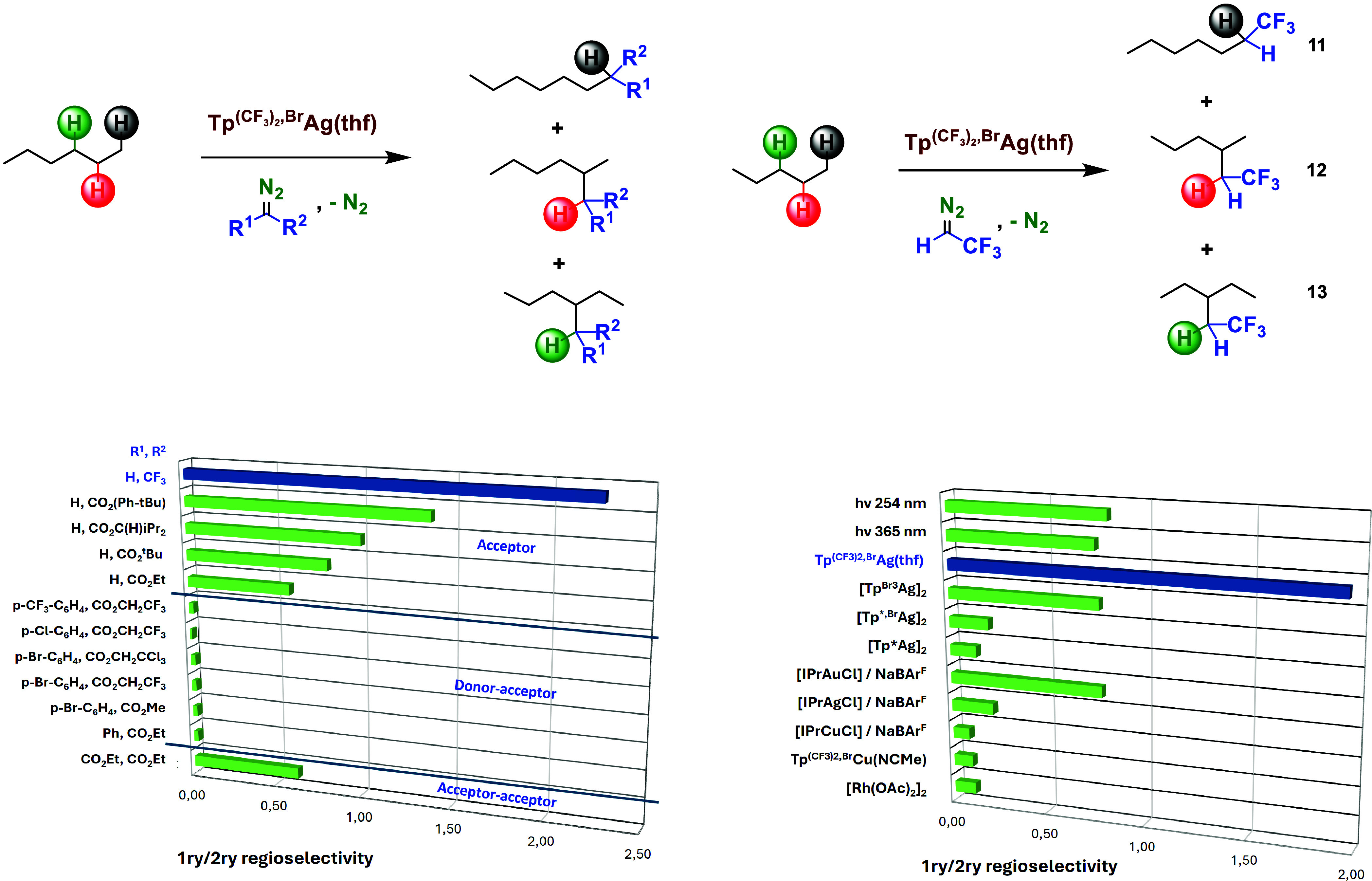
Diazo Compound (Left) and Catalyst (Right) Screenings for Regioselectivity Optimization with Linear Alkanes as Models Pentane for catalyst screening and hexane for diazo screening. See the Supporting Information for details.

The large preferential primary functionalization observed with TFDE prompted us to evaluate the singularity of these data. First, we wondered about the effect of the diazo compound in the functionalization of a linear alkane such as hexane as a representative substrate to evaluate the primary/secondary selectivity, using complex **1** as the catalyst (Scheme 5). The series of donor–acceptor diazo compounds^13^ did not provide significant amounts of the products derived from the CH_3_-sites, with only secondary C–H bond modification being observed. This selectivity changed when moving to the series of acceptor diazo compounds of general formula N_2_=C(H)R.^13^ In this case, with R being a carboxylate CO_2_R’, the 1ry:2ry selectivity depended on the size of the R′ group: the higher the volume of R′, the better the selectivity toward primary sites. However, when changing from R = CO_2_R’ to R = CF_3_, a significant increase of such selectivity was found, assessing the profound effect of this substitution at the diazo functionality in this transformation. The acceptor–acceptor diazo compound did not improve the selectivity, whereas the donor–donor combination did not lead to C–H bond functionalization, promoting carbene coupling in an exclusive manner.^13^

Once the outstanding performance of TFDE in comparison with other diazo reagents was identified, a catalyst screening was performed using such a carbene source and pentane as the substrate (Scheme 5). Well-known catalysts for carbene transfer such as dirhodium tetraacetate or the IPrMCl (M = coinage metal) series induced carbene insertion into secondary C–H bonds in a preferential manner (1ry/2ry < 1). Complexes bearing trispyrazolylborate ligands with the cores Tp^(CF3)2,Br^Cu, Tp*Ag, Tp*^,Br^Ag, and Tp^Br3^Ag also favored those sites. However, complex **1** led to a completely different reaction outcome with the primary sites being preferred for the carbene insertion reaction.

In some cases, it has been proposed^30^ that the carbene moiety may dissociate from the metal-carbene intermediate before interaction with the nucleophile takes place. To discard such a possibility, experiments carried out in the absence of catalysts and under irradiation (254 and 365 nm) to generate free carbenes led to selectivities quite different from those generated by the silver catalysts. Also, the involvement of radical species^31^ has been ruled out upon running twin experiments with and without BHT as a radical trap, which showed the same degree of functionalization via carbene transfer (see the Supporting Information).

### Enhanced Functionalization of Primary Sites with TFDE as the Carbene Source

**Figure 1:**
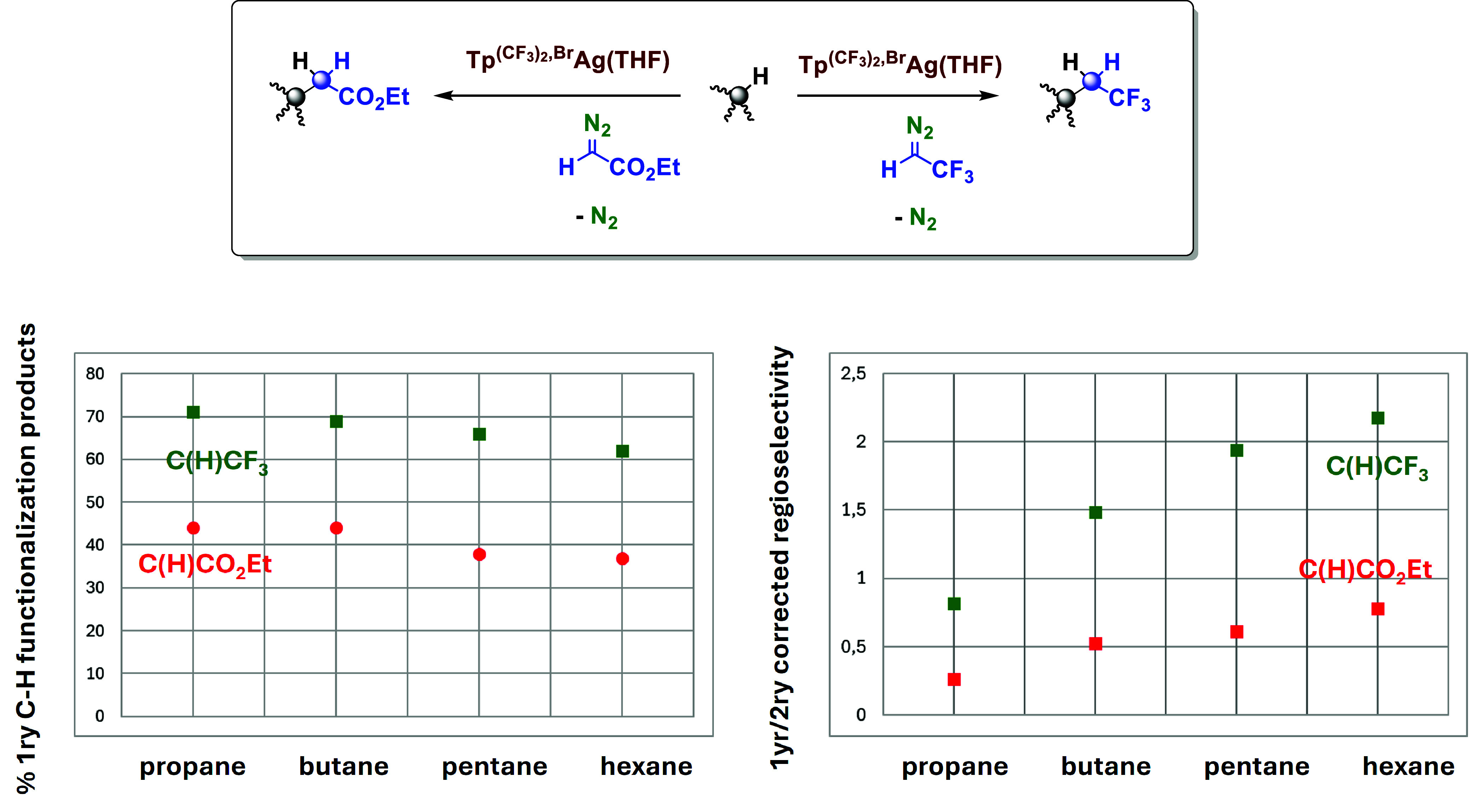
Influence of the carbene nature in the regioselectivity for the C_3_–C_6_ alkane functionalization. Left: variation of the percentage of 1ry C−H bond functionalization product. Right: 1ry/2ry insertion corrected with the number of C–H bonds of each type. For propane and butane, scCO_2_ was employed as the reaction medium. Pentane and hexane were employed as the solvents. See the Supporting Information for details.

The selective functionalization of alkane C–H bonds by carbene insertion is still limited to a few examples. Davies has disclosed three rhodium-based catalytic systems for the exclusive functionalization of primary, secondary, and tertiary sites of alkanes.^14^ Apart from rhodium, only silver is known to promote selective transformations, with examples provided by Bi^15^ (tertiary sites) and our group (secondary sites).^13^ With the available data resulting from the use of complex **1** as a catalyst, a comparison of the outcome for the propane–hexane series when using TFDE and EDA^11^ is shown in Figure 1 (left). The exceptional preference for primary sites observed with TFDE seems general for the series, with 60–70% of the products derived from CH_3_ functionalization. With EDA, such values decreased by ca. 25%, with the same trend being observed.

When the distribution of products is corrected by the number of C–H bonds of each type, e.g., including the statistic factor, the variation is shown in Figure 1 (right). With EDA, the 1ry:2ry selectivity is lower than 1 in all cases. On the other hand, with TFDE, such a ratio is higher than 1 for the butane–hexane series, with only that of propane being below. With this comparison, the influence of the carbene group on the regioselectivity and the exceptional behavior of the carbene C(H)CF_3_ is demonstrated. It is worth recalling the selectivity observed with isobutane (Scheme 4), where a highly reactive tertiary site is available; with EDA,^11^ the 3ry:1ry ratio was found as 5.0, whereas in the TFDE case, the ratio decreases to 2.0, again showing the preferential selectivity toward the primary sites when using the **1**-TFDE couple.

### DFT and MD Study with Propane as the Substrate

**Figure 2:**
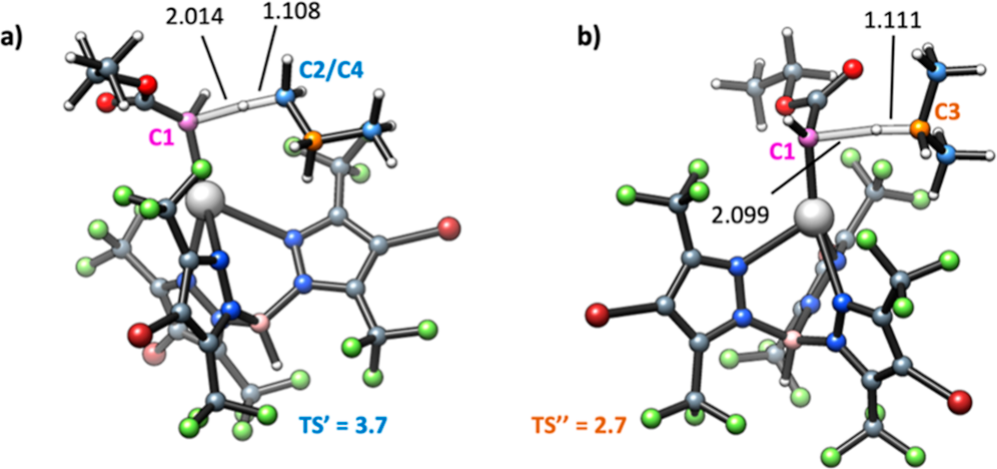
B3LYP-D3-characterized TSs for (a) primary and (b) secondary C–H activations of propane. Free energy barriers are also shown in kcal mol^–1^ relative to the energy of the [Ag] = C(H)CO_2_Et·C_3_H_8_ adduct. Carbene (C1), 1ry (C2/C4), and 2ry (C3) carbons are depicted in purple, blue, and orange, respectively. Bonds forming and breaking are depicted as transparent lines, and their distances are reported in Å.

The reactive behavior of both C(H)CO_2_Et and C(H)CF_3_ carbene units has been analyzed by a multilevel computational approach using propane (C_3_H_8_) as a cosubstrate. The reaction with EDA leading to [Ag]=C(H)CO_2_Et follows previously established patterns and is included here for comparison. This reaction confirms our previous reports showing a high exergonic (Δ*G*_sol_ = −56 kcal mol^–1^) one-step process with low activation barriers for either the 1ry or 2ry C−H bond molecule located at 6.0 kcal mol^–1^ above the reactants. From this intermediate, we could locate the TSs and the corresponding energy barriers, which highlighted an energetic preference of about 1.0 kcal mol^–1^ toward 2ry C−H (Table SC2). The geometry of the lowest TS for the 1ry and 2ry C–H activations is depicted in Figure 2. The comparison of the energy barriers gives a computational prediction for selectivity, in agreement with experiment.

The same computational approach cannot be applied to the system with the C(H)CF_3_ carbene. No supramolecular adduct was characterized, with all of the simulations evolving toward the formation of both products and the selectivity depending on the starting disposition of the propane. The lack of a TS for the reaction is fully consistent with the ready methane activation with this carbene.

**Figure 3:**
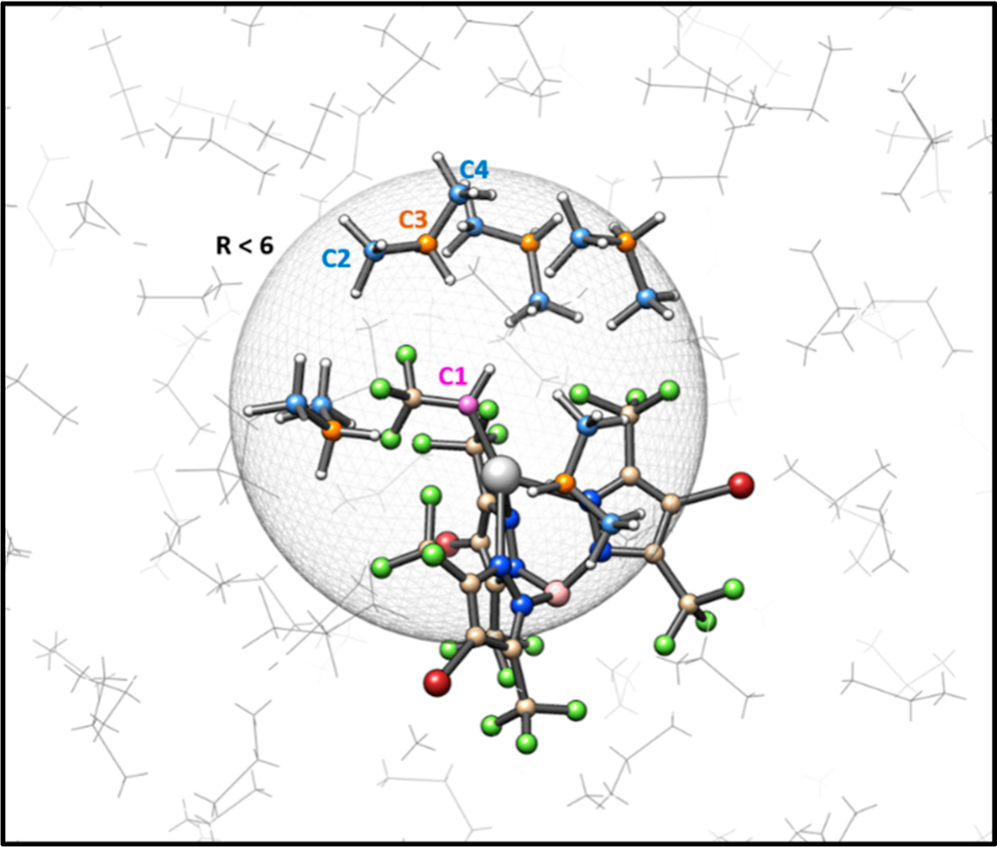
Representative MD frame of the propane-solvated [Ag] = C(H)CF_3_. The propane molecules within 6.0 Å are shown in ball and stick, whereas the bulk is depicted as gray wires. Carbene (C1), 1ry (C2/C4), and 2ry (C3) carbons are depicted in purple, blue, and orange, respectively.

Having these data in hand, we turned our attention to the solvation structure of the catalysts. With the aim to rationalize the observed selectivity in terms of distribution of prereactive conformations, we set up classic bonded MD simulations of the solvated silver carbene along 100 ns at 300 K. The use of force-field-based bonded MD vs ab initio techniques ensured the possibility of widely exploring solvent distribution, avoiding bond breaking and indeed freezing the prereactive state. The computed pair radial distribution function for C1–C2/C4 and C1–C3 along the whole trajectory (Figure SC1) showed a maximum of probability of finding the primary carbon in the range of 5.0–6.5 Å from the metal center, whereas the range for the secondary carbon extended further away at 5.0–7.5 Å. Moreover, the visual inspection of the structure shows that the 1ry carbons are in a clear prereactive disposition, while the 2ry ones are mainly located on top of the silver carbene or with the H atoms opposite with respect to the carbene (see Figure 3). The MD calculations indicate that it is more likely to find the primary carbons close to the carbene. This proximity is consistent with the experimental outcome, which can be computationally rationalized, even in the absence of TSs. We emphasize that the selectivity opposes the prediction from enthalpy: it thus has a purely entropic origin.

## Conclusions

We herein report a catalytic system for the functionalization of alkanes C_1_–C_6_ (methane–hexane) that occurs through a pathway that has no barrier in the potential energy path for the step where the alkane C–H bond is activated. This behavior, anticipated by DFT studies, brings an enhancement of the selectivity toward the primary sites of the alkanes studied. This occurs when a transient silver-carbene species is formed from N_2_=C(H)CF_3_, the interaction of this species with alkane primary and secondary sites lacking enthalpic barriers, as inferred from DFT studies. The explanation for the preference for primary sites arises from MD studies, showing that there is a higher probability of finding a methyl C–H bond close to the carbene carbon atom than for the methylene C–H bond. These results demonstrate that with the appropriate design of the catalytic system, the highly energetic carbon–hydrogen bonds of methane (and other low-reactive light alkanes) can be activated at nearly no energetic cost and that the selectivity toward activation of primary carbon–hydrogen bonds can be subsequently enhanced.

## Supporting Information Available

All procedures and characterization data, computational data, and Cartesian coordinates of the optimized structures (PDF)
