# Supplementary material for: Alkanes C1–C6 C–H Bond Activation via a Barrierless Potential Energy Path: Trifluoromethyl Carbenes Enhance Primary C–H Bond Functionalization
Source: J Am Chem Soc. 2024 Nov 25;146(49):34014–22. doi: 10.1021/jacs.4c13065 (PMC11638901; doi:10.1021/jacs.4c13065)
Supplement: Supplementary file 1 — ja4c13065_si_001.pdf [file ja4c13065_si_001.pdf]

Supporting Information for

Alkanes C_1-C_6 C-H Bond Activation via a Barrierless Potential Energy Path: Trifluoromethyl Carbenes Enhance Primary C-H Bond Functionalization

Jonathan Martínez-Laguna,[‡] Julia Altarejos,[§] M. Ángeles Fuentes,[‡] Giuseppe Sciortino,[¥,||] Feliu Maseras,[*, ¥,||] Javier Carreras,[*,§] Ana Caballero,[*, ‡] Pedro J. Pérez[*,‡]

[‡]Laboratorio de Catálisis Homogénea, Unidad Asociada al CSIC, CIQSO-Centro de Investigación en Química Sostenible and Departamento de Química, Universidad de Huelva, 21007 Huelva, Spain.

[§]Departamento de Química Orgánica y Química Inorgánica, Instituto de Investigación Química "Andrés M. del Río" (IQAR), Universidad de Alcalá, 28805 Alcalá de Henares, Madrid, Spain.

[¥]Institute of Chemical Research of Catalonia (ICIQ-CERCA), Avgda. Països Catalans, 16, The Barcelona Institute of Science and Technology, 43007 Tarragona, Spain.

[||]Departament de Química, Universitat Autònoma de Barcelona, 08193 Bellaterra, Spain.

Table of Contents

1. General information S1

2. Catalytic experiments S1

a. Reactions performed with neat alkanes as reaction medium S1

b. Reactions performed with $scCO_2$ as reaction medium S3

3. Synthesis of pure samples of trifluoromethyl alkanes S4

a. General procedure I S5

b. General procedure II S5

4. Experimental data and characterization S7

5. NMR spectra data for catalytic experiments S14

6. NMR spectra data for organic compounds S18

7. GC traces for methane and ethane functionalization S66

8. Computational details S67

9. References for experimental section S72

10. References for computational section S72

11. Cartesian coordinates S74

1. General information

All air- and moisture-sensitive manipulations were carried out with standard Schlenk techniques under nitrogen atmosphere or in a glovebox (MBRAUN UNILAB) under an atmosphere of purified nitrogen. All reactants were purchased from Sigma-Aldrich and used without further purification. Solvents were purchased from Scharlau and dried using standard protocols: cyclohexane, n-hexane and n-pentane were refluxed over sodium/benzophenone and separated by distillation under nitrogen. Gaseous alkanes were obtained from Air Liquide and used as received. The complex $Tp^{(CF_3)_2,Br}Ag(thf)$ (**1**) was prepared following the reported procedure.[1] 2,2,2-Trifluorodiazoethane ($N_2C(H)CF_3$, TFDE, **2**) was prepared according with the literature method and kept in a stock solution of hexafluorobenzene.[2] The concentration of the stock solution of the diazo compound in hexafluorobenzene was determined by ^{19}F NMR spectroscopy employing α,α,α-trifluorotoluene as internal standard and was established as ca. 1 M. Nuclear magnetic resonance (NMR) spectra were recorded at room temperature from solutions in $CDCl_3$ (unless otherwise noted) on Bruker spectrometer operating at 400 MHz (^1H NMR) and were referenced to residual solvent peak (chloroform: 7.26 ppm for ^1H NMR). For gaseous alkanes, the experiments were performed in a PARR Micro Bench Top reactor with a Teflon container connected to a commercial Iberfluid supercritical plant for the experiments using $scCO_2$.

2. Catalytic experiments

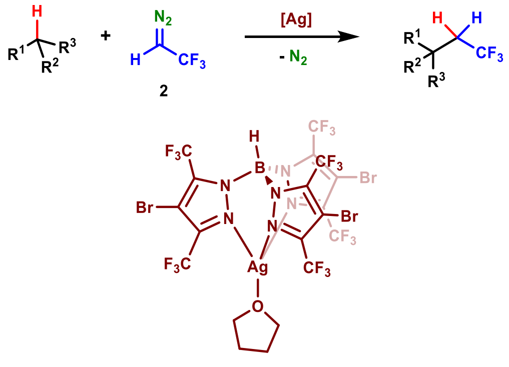

[Ag] = $Tp^{(CF_3)_2,Br}Ag(thf)$ (**1**)

Scheme S1. Silver-catalyzed functionalization of C1-C6 alkanes with TFDE.

a. Reactions performed with neat liquid alkanes as reaction medium.

In a Schlenk flask, the catalyst (0.05 mmol; 5% or 0.01 mmol; 1%) was dissolved in the neat alkane (n-pentane, n-hexane, cyclohexane or n-butane (at -30 °C); 5 mL) at the desired temperature. 2,2,2-Trifluorodiazoethane (TFDE, **2**) (1.0 mmol) in hexafluorobenzene (1 mL) was added in one portion, and the mixture was stirred for 1 h. For the case of n-butane, 5 mL of DCM were added at the end of the reaction. When the reaction was completed, the mixture was allowed to reach room temperature and yields were determined by ^{19}F NMR analysis employing fluorobenzene as internal standard. Trifluoroalkanes were identified by NMR through comparation with reported data or by its direct comparation with authentic samples independently prepared.[3]

For experiments under irradiation conditions with n-hexane as substrate, the above protocol was employed, in the absence of catalyst and using a UV lamp (254 or 365 nm) attached to the Schlenk flask.

Table S1. Results obtained in the catalyst optimization.

Entry	Catalyst	Substrate (Solvent)	Catalyst charge (mol%)[d]	Yield (%)	C1/C2/C2' selectivity[e]	C1/C2/C2' selectivity (normalized)
1[a]	$[Tp^{*}Ag]_2$	n-Pentane	5	7	12/69/19	1.0/5.8/1.6
2[a]	$[Tp^{*,Br}Ag]_2$	n-Pentane	5	16	17/67/16	1.1/4.2/1.0
3[a,c]	$[Tp^{Br3}Ag]_2$	n-Pentane	5	71	48/41/11	4.4/3.7/1.0
4[a]	$[Rh(OAc)_2]_2$	n-Pentane	5	15	10/69/21	1.0/6.9/2.1
5[a,b]	[IPrCuCl] / $NaBAr^F$	n-Pentane	5	12	8/67/25	1.0/8.4/3.1
6[a,b]	[IPrAgCl] / $NaBAr^F$	n-Pentane	5	8	18/55/27	1.0/3.1/1.5
7[a,b]	[IPrAuCl] / $NaBAr^F$	n-Pentane	5	16	44/39/17	2.6/2.3/1.0
8[c]	$Tp^{(CF_3)_2,Br}Cu(NCMe)$	n-Pentane	1	68	9/83/8	1.1/10.4/1.0
9[c]	$Tp^{(CF_3)_2,Br}Ag(thf)$ (**1**)	n-Pentane	1	80	66/30/4	16.5/7.5/1.0
10	*hv* 254 nm	n-Hexane	-	<1	43/31/26	1.7/1.2/1.0
11	*hv* 365 nm	n-Hexane	-	8	45/30/25	1.8/1.2/1.0
12	none	Cyclohexane	-	n. d.	-	-

[a]5 mL of DCM was added as co-solvent. [b]NaBArF was added along with the catalyst as halide scavenger (0.05 mmol; 5%). [c]Starting diazo compound has been completely consumed. [d]Referred to the diazo compound (mol%). [e]Distribution of products. n. d. = not detected.

*Table S2. Results obtained in reactions performed with neat alkanes as reaction media with catalyst **1*** (1 mol% referred to diazo compound).

Entry	Substrate (Solvent)	T (ºC)	Yield (%)	C1/C2/C2' selectivity[c]	C1/C2/C2' selectivity (normalized)
1	n-Hexane	25	76	62/28/10	6.2/2.8/1.0
2	n-Hexane	-30	89	63/27/10	6.3/2.7/1.0
3	n-Pentane	25	80	66/30/4	16.5/7.5/1.0
4	n-Pentane	-30	88	67/27/6	11.2/4.5/1.0
5[b]	n-Butane	-30	82	69/31	2.2/1.0
6	Cyclohexane	25	98	-	-

[a]1 mol% as catalyst referred to the diazo compound. [b]The reaction was carried out employing n-butane as solvent in a high-pressure J-Young ampoule. [c]Percentage distribution of products.

b. Reactions performed with $scCO_2$ as reaction medium.

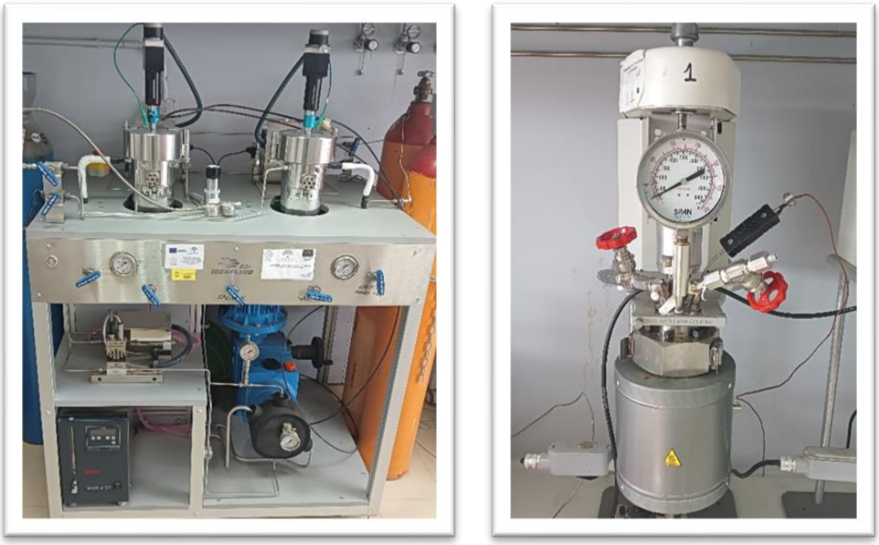

Figure S1. Iberfluid supercritical plant for the experiments using $scCO_2$ (left) and PARR Micro Bench Top reactor with a Teflon container (right).

The complex **1** (0.01 mmol; 1%) was placed into a polypropylene tube, and both sides were closed with a permeable cap. It was fixed to the mixer axis of a 100 mL high-pressure reactor. A solution of TFDE (**2**, 1.0 mmol) in hexafluorobenzene (1 mL) was added into a Teflon cap, also fixed ath the same axis. The reactor was closed and pressurized first with the alkane (1.5 atm for isobutane; 1 atm for n-butane; 7 atm for propane; 30 atm for ethane; 160 atm for methane) and then with 90 atm of carbon dioxide at 40 °C. After 14 h of stirring at 40 °C, the reactor was cooled in a water/ice bath and the system was depressurized through two cold traps containing 10 mL of DCM at -78 °C. The solutions were collected and analysed

by GC and NMR spectroscopy. Although the products are quite volatile, they could be identified by NMR spectroscopy. Yields were determined by ^{19}F NMR analysis employing fluorobenzene as internal standard (experiments with propane, n-butane and isobutane) or GC (external calibration, experiments with methane and ethane).

Trifluoroalkanes were identified by NMR through comparation with reported data or by its direct comparation with authentic samples prepared by organic synthesis.[4]

*Table S3. Results obtained in reactions performed with $scCO_2$ as reaction media employing **1** as catalyst.*

Entry	Substrate	Catalyst:diazo:alkane ratio (mmol)	Yield (%)	C1/C2/C3 selectivity	C1/C2/C3 selectivity (normalized)
1	Isobutane	1.0:100:399	75	81/ - /19	4.3/ - /1.0
2	n-Butane	1.0:100:267	65	69/31/ -	2.2/1.0/ -
3	Propane	1.0:100:1738	52	71/29/ -	2.4/1.0/ -
4	Ethane	1.0:100:7075	58	-	-
5	Methane	1.0:100:31776	42	-	-

3. Synthesis of pure samples of trifluoromethyl alkanes

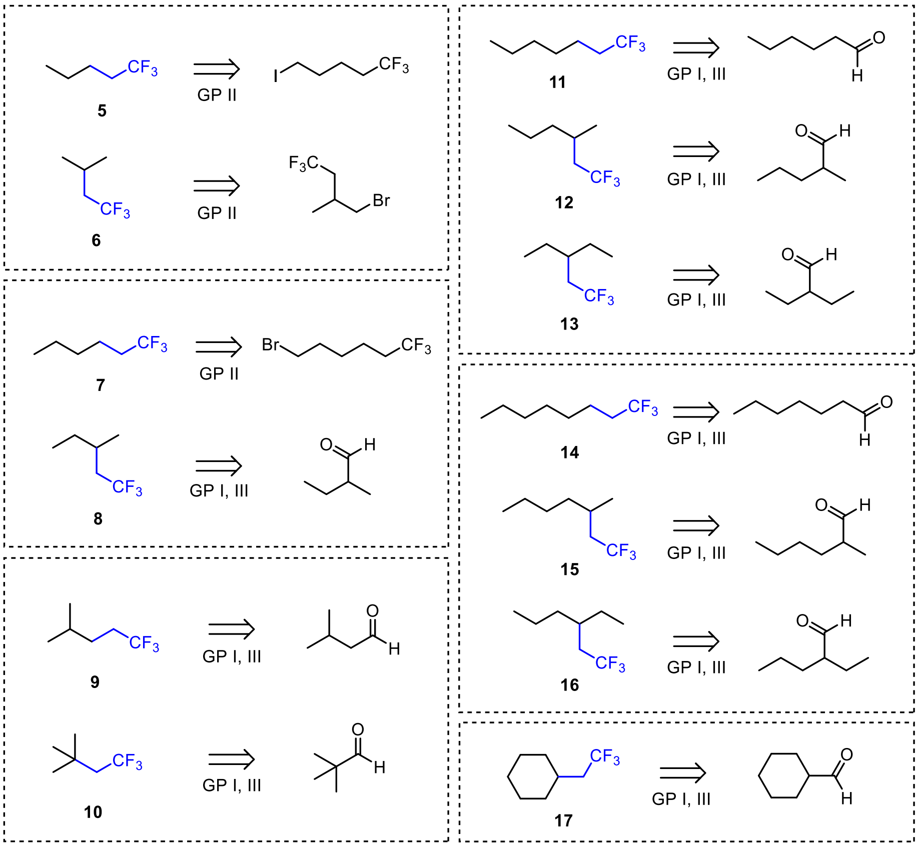

Figure S2. General procedures employed for the preparation of the trifluoromethyl alkanes.

a) General procedure I

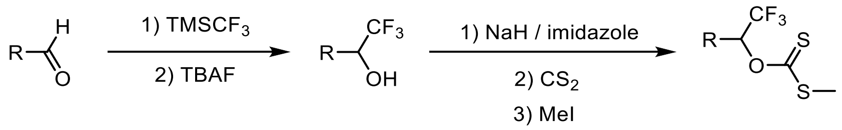

Scheme S2. Synthetic route for general procedure I.

The corresponding aldehyde (10 mmol) and $TMSCF_3$ (1.2 equiv.) were stirred at 0 °C in dry DMF (0.4 M). To the reaction mixture, a TBAF solution (1 M in THF, 0.01 equiv.) was added and stirred at the same temperature for 10 min. Then, the ice-water bath was removed, and the reaction was stirred at rt for 6 h. After cooling at 0 °C, TBAF (2.5 equiv.) was added, and the resulting mixture was stirred at rt for 1.5 h. The mixture was extracted with diethyl ether (4 times) and washed with brine. The organic layers were collected and dried with Na_2SO_4, filtered and concentrated (220 mbar). The resulting alcohol was used without further purification. It was added at 0 °C to a solution of NaH (2.5 equiv.) and imidazole (0.05 equiv.) in THF for 30 min. Then, CS_2 (2.5 equiv.) was added dropwise to the mixture and stirred for 30 min. Later, MeI (2.5 equiv.) was added and stirred for another 30 min. When the reaction is completed, acetic acid in water (3 equiv.) was added dropwise and the reaction was extracted with DCM (3 times). The organic layers were washed with brine, dried, filtered and concentrated under vacuum. The crude residue was purified by column chromatography (hexane) to obtain the corresponding xanthate. The synthesis of both alcohols[5] and xanthates[6] are based on reported procedures.

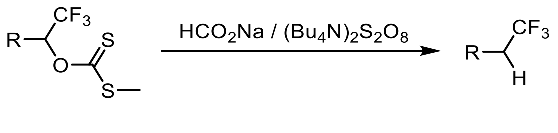

Scheme S3. Reaction conditions for general procedure III.

A mixture of the xanthate generated as above (0.8 mmol), sodium formate (6 equiv.) and $(Bu_4N)_2S_2O_8$ (3 equiv.) in dry DMF (0.2 M) was stirred at 65 °C for 30 min. Upon completion of the reaction, the mixture was poured into water and the product was extracted with C_6D_{12} (2 x 0.4 mL) and washed with water (8 x 0.5 mL). The organic layer was dried with Na_2SO_4 and filtered through a short pad of silica, affording a solution of the alkane in C_6D_{12}. This Barton-McCombie reaction procedure is based on a reported procedure.[6]

b) General procedure II

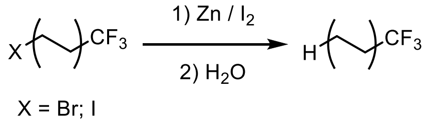

Scheme S4. Reaction conditions for general procedure II.

In a Schlenk flask at 70 °C, zinc (2 equiv.) was activated with a small portion of I_2 under inert atmosphere. Then, dry DMA was added and stirred until the red colour disappear. The corresponding halogenated compound was added dropwise, and the reaction was stirred for 8 hours at 70 °C. Next, H_2O (3 equiv.)

was added at the same temperature and stirred overnight. Once the reaction is completed, the trifluoromethyl alkane was purified by distillation. The procedure has been slightly modified from a reported procedure.[7]

4. Experimental data and characterization

S-Methyl *O*-(1,1,1-trifluoro-3-methylpentan-2-yl) carbonodithioate (8')

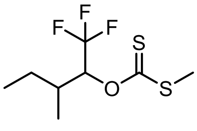

8'

Alcohol was prepared following the general procedure I from 2-methylbutanal (1.1 mL, 10.0 mmol) and $TMSCF_3$ (1.8 mL, 12.0 mmol). Xanthate was prepared from the corresponding alcohol (1.0 g, 6.4 mmol), NaH (641 mg, 16.0 mmol), imidazole (19.1 mg, 0.3 mmol), CS_2 (0.9 mL, 16.0 mmol) and MeI (0.9 mL, 16.0 mmol). Pale yellow liquid (0.9 g, 34% (2 steps)).

^{1}H NMR (500 MHz, $CDCl_3$) δ 6.32 – 6.21 (m, 1H, diast. A), 6.19 – 6.09 (m, 1H, diast. B), 2.61 (s, 6H, diast. A+B), 2.14 – 2.02 (m, 2H, diast. A+B), 1.68 – 1.61 (m, 1H, diast. A), 1.57 – 1.49 (m, 1H, diast. A), 1.35 – 1.26 (m, 2H, diast. B), 1.11 – 1.05 (m, 6H, diast. A+B), 1.00 – 0.91 (m, 6H, diast. A+B) ppm. **^{13}C{^{1}H} NMR** (126 MHz, $CDCl_3$) δ 216.3 (diast. A), 216.1 (diast. B), 123.9 (q, *J* = 282.6 Hz), 80.3 (q, *J* = 30.5 Hz, diast. B), 79.0 (q, *J* = 30.7 Hz, diast. A), 35.2, 35.1, 26.1, 24.5, 19.5, 14.7 (q, *J* = 1.8 Hz,), 14.1 (q, *J* = 1.6 Hz), 11.7, 11.2 ppm. **^{19}F NMR** (376 MHz, $CDCl_3$) δ -71.8 (d, *J* = 7.4 Hz, diast. A), -72.7 (d, *J* = 5.8 Hz, diast. B) ppm. **HRMS-APCI** m/z calculated for $C_8H_{13}F_3OS_2$ $[M+H]^+$ 247.0433, found 247.0434.

S-methyl *O*-(1,1,1-trifluoro-4-methylpentan-2-yl) carbonodithioate (9')

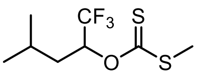

9'

Alcohol was prepared following the general procedure I from cyclohexanecarbaldehyde (1.1 mL, 10.0 mmol) and $TMSCF_3$ (1.8 mL, 12.0 mmol). Xanthate was prepared from the corresponding alcohol (1.4 g, 9.0 mmol), NaH (896 mg, 22.4 mmol), imidazole (30.5 mg, 0.5 mmol), CS_2 (1.4 mL, 22.4 mmol) and MeI (1.40 mL, 22.4 mmol). Pale yellow liquid (0.61 g, 25% (2 steps)).

^{1}H NMR (400 MHz, $CDCl_3$) δ 6.22 (dqd, *J* = 9.9, 6.4, 3.1 Hz, 1H), 2.51 (s, 3H), 1.81 (ddd, *J* = 14.3, 10.2, 4.3 Hz, 1H), 1.70 – 1.57 (m, 1H), 1.53 (ddd, *J* = 14.0, 9.1, 3.1 Hz, 1H), 0.88 (d, *J* = 6.6 Hz, 3H), 0.87 (d, *J* = 6.5 Hz, 3H) ppm. **^{13}C{^{1}H} NMR** (101 MHz, $CDCl_3$) δ 216.1, 123.7 (q, *J* = 281.5 Hz), 75.8 (q, *J* = 32.0 Hz), 37.0 (q, *J* = 1.5 Hz), 24.0, 23.2, 22.1j, 19.6 ppm. **^{19}F NMR** (376 MHz, $CDCl_3$) δ -76.5 (d, *J* = 6.4 Hz) ppm. **HRMS-ESI** m/z calcd for $C_8H_{13}F_3OS_2Na$ $[M+Na]^+$ 269.0252, found 269.0424.

S-methyl *O*-(1,1,1-trifluoro-3,3-dimethylbutan-2-yl) carbonodithioate (10')

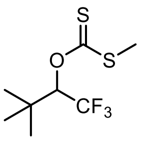

10'

Alcohol was prepared following the general procedure I from cyclohexanecarbaldehyde (1.1 mL, 10.0 mmol) and $TMSCF_3$ (1.8 mL, 12.0 mmol). Xanthate was prepared from the corresponding alcohol (1.5 g, 9.3 mmol), NaH (960 mg, 24.0 mmol), imidazole (32.7 mg, 0.5 mmol), CS_2 (1.5 mL, 24.0 mmol) and MeI (1.50 mL, 24.0 mmol). Pale yellow liquid (1.25 g, 51% (2 steps)).

^{1}H NMR (400 MHz, $CDCl_3$) δ 6.07 (q, *J* = 7.7 Hz, 1H), 2.62 (s, 3H), 1.11 (q, *J* = 1.2 Hz, 9H) ppm. **^{13}C{^{1}H} NMR** (101 MHz, $CDCl_3$) δ 216.5, 123.9 (q, *J* = 284.2 Hz), 82.5 (q, *J* = 29.5 Hz), 34.9, 26.5 (q, *J* = 2.1 Hz), 19.5 ppm. **^{19}F NMR** (376 MHz, $CDCl_3$) δ -69.1 (d, *J* = 7.5 Hz) ppm. **HRMS-ESI** m/z calcd for $C_8H_{13}F_3OS_2K$ $[M+K]^+$ 284.9991, found 285.0116.

S-Methyl *O*-(1,1,1-trifluoroheptan-2-yl) carbonodithioate (11')

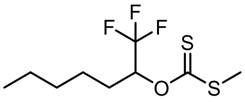

11'

Alcohol was prepared following the general procedure I from hexanal (1.2 mL, 10.0 mmol) and $TMSCF_3$ (1.8 mL, 12.0 mmol). Xanthate was prepared from the corresponding alcohol (0.9 g, 5.3 mmol), NaH (533 mg, 13.3 mmol), imidazole (13.5 mg, 0.2 mmol), CS_2 (0.8 mL, 13.3 mmol) and MeI (0.8 mL, 13.3 mmol). Pale yellow liquid (400 mg, 15% (2 steps)).

^{1}H NMR (500 MHz, $CDCl_3$) δ 6.29 – 6.17 (m, 1H), 2.61 (s, 3H), 1.92 – 1.87 (m, 2H), 1.46 – 1.39 (m, 2H), 1.36 – 1.30 (m, 4H), 0.89 (t, *J* = 7.1 Hz, 3H) ppm. **^{13}C{^{1}H} NMR** (126 MHz, $CDCl_3$) δ 216.0, 123.7 (q, *J* = 281.4 Hz), 77.2 (q, *J* = 32.0 Hz), 31.5, 28.2 (q, *J* = 1.5 Hz), 24.1, 22.4, 19.6, 14.0. **^{19}F NMR** (376 MHz, $CDCl_3$) δ -76.2 (d, *J* = 6.5) ppm. **HRMS-ESI** m/z calculated for $C_9H_{15}F_3OS_2$ $[M\text{-}CS]^+$ 216.0796, found 216.0870.

S-Methyl *O*-(1,1,1-trifluoro-3-methylhexan-2-yl) carbonodithioate (12')

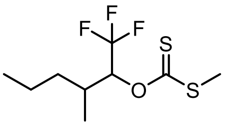

12'

Alcohol was prepared following the general procedure I from 2-methylpentanal (1.2 mL, 10.0 mmol) and $TMSCF_3$ (1.8 mL, 12.0 mmol). Xanthate was prepared from the corresponding alcohol (1.4 g, 8.2 mmol), NaH (821 mg, 20.5 mmol), imidazole (25.4 mg, 0.4 mmol), CS_2 (1.2 mL, 20.5 mmol) and MeI (1.2 mL, 20.5 mmol). Pale yellow liquid (1.2 g, 46% (2 steps)).

^{1}H NMR (400 MHz, $CDCl_3$) δ 6.23 (qd, *J* = 7.4, 3.7 Hz, 1H, diast. A), 6.12 (qd, *J* = 7.0, 6.9 Hz, 1H, diast. B), 2.61 (s, 6H, diast. A+B), 2.24 – 2.08 (m, 2H, diast. A+B), 1.58 – 1.20 (m, 8H, diast. A+B), 1.07 (d, *J* = 6.9 Hz, 6H, diast. A+B), 0.91 (t, *J* = 6.9 Hz, 3H, diast. B), 0.90 (t, *J* = 7.1 Hz, 3H, diast. A) ppm. **^{13}C{^{1}H} NMR** (101 MHz, $CDCl_3$) δ 216.2 (diast. A), 216.1 (diast. B), 123.8 (q, *J* = 282.8 Hz), 80.4 (q, *J* = 30.4 Hz, diast. B), 79.2 (q, *J* = 30.6 Hz, diast. A), 35.3, 33.7, 33.4, 33.2, 20.2, 20.0, 19.5, 15.2, 14.4, 14.1, 14.0 ppm. **^{19}F NMR** (282 MHz, $CDCl_3$) δ -71.7 (d, *J* = 7.2 Hz, diast. A), -72.6 (d, *J* = 7.0 Hz, diast. B) ppm. **HRMS-APCI** m/z calculated for $C_9H_{15}F_3OS_2$ $[M+H]^+$ 261.0589, found 261.0589.

S-Methyl *O*-(1,1,1-trifluoro-3-ethylpentan-2-yl) carbonodithioate (13')

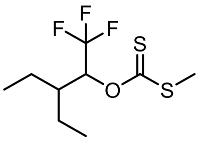

13'

Alcohol was prepared following the general procedure I from 2-ethylbutanal (1.2 mL, 10.0 mmol) and $TMSCF_3$ (1.8 mL, 12.0 mmol). Xanthate was prepared from the corresponding alcohol (1.4 g, 8.2 mmol), NaH (821 mg, 20.5 mmol), imidazole (25.4 mg, 0.4 mmol), CS_2 (1.2 mL, 20.5 mmol) and MeI (1.2 mL, 20.5 mmol). Pale yellow liquid (1.71 g, 65% (2 steps)).

^{1}H NMR (400 MHz, $CDCl_3$) δ 6.29 (qd, *J* = 7.4, 4.7 Hz, 1H), 2.61 (s, 3H), 1.98 – 1.79 (m, 1H), 1.74 – 1.61 (m, 1H), 1.50 (dt, *J* = 13.8, 6.9 Hz, 2H), 1.39 (dq, *J* = 14.9, 7.4 Hz, 1H), 0.96 (td, *J* = 7.5, 3.7 Hz, 6H) ppm. **^{13}C{^{1}H} NMR** (101 MHz, $CDCl_3$) δ 216.1, 124.0 (q, *J* = 282.8 Hz), 78.3 (q, *J* = 30.6 Hz), 41.4, 21.9, 21.4, 19.5, 11.5, 11.4 ppm. **^{19}F NMR** (376 MHz, $CDCl_3$) δ -72.4 (d, *J* = 7.7 Hz, CF_3) ppm. **HRMS-ESI** m/z calculated for $C_9H_{15}F_3OS_2$ $[M+H]^+$ 261.0589, found 261.0599.

S-Methyl *O*-(1,1,1-trifluorooctan-2-yl) carbonodithioate (14')

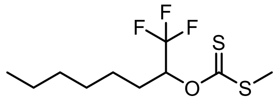

14'

Alcohol was prepared following the general procedure I from heptanal (1.4 mL, 10.0 mmol) and $TMSCF_3$ (1.8 mL, 12.0 mmol). Xanthate was prepared from the corresponding alcohol (1.2 g, 7.1 mmol), NaH (713 mg, 17.8 mmol), imidazole (20.3 mg, 0.3 mmol), CS_2 (1.0 mL, 17.8 mmol) and MeI (1.0 mL, 17.8 mmol). Pale yellow liquid (920 mg, 35% (2 steps)).

^{1}H NMR (500 MHz, $CDCl_3$) δ 6.28 – 6.17 (m, 1H), 2.61 (s, 3H), 1.95 – 1.84 (m, 2H), 1.49 – 1.32 (m, 4H), 1.32 – 1.23 (m, 4H), 0.88 (t, *J* = 6.9 Hz, 3H) ppm. **^{13}C{^{1}H} NMR** (126 MHz, $CDCl_3$) δ 216.0, 123.7 (q, *J* = 281.4 Hz), 77.2 (q, *J* = 32.0 Hz), 31.6, 29.0, 28.2 (q, *J* = 1.4 Hz), 24.4, 22.6, 19.5, 14.1 ppm. **^{19}F NMR** (376 MHz, $CDCl_3$) δ -76.1 (d, *J* = 6.5 Hz) ppm. **HRMS-ESI** m/z calculated for $C_{10}H_{18}F_3OS_2$ $[M+H]^+$ 275.0745, found 275.0791.

S-Methyl *O*-(1,1,1-trifluoro-3-methylheptan-2-yl) carbonodithioate (15')

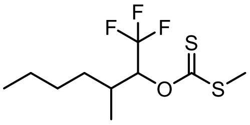

15'

2-methylhexanal was prepared following the described procedure from Stewart *et al.*[8] Alcohol was prepared following the general procedure I from 2-methylhexanal (1.7 mL, 10.0 mmol) and $TMSCF_3$ (1.8 mL, 12.0 mmol). Xanthate was prepared from the corresponding alcohol (1.3 g, 7.1 mmol), NaH (713 mg, 17.8 mmol), imidazole (20.3 mg, 0.3 mmol), CS_2 (1.0 mL, 17.8 mmol) and MeI (1.0 mL, 17.8 mmol). Pale yellow liquid (1.10 g, 40% (2 steps)).

^{1}H NMR (400 MHz, $CDCl_3$) δ 6.23 (qd, *J* = 7.4, 3.7 Hz, 1H, diast. A), 6.11 (qd, *J* = 7.2, 6.1 Hz, 1H, diast. B), 2.61 (s, 6H, diast. A+B), 2.21 – 2.09 (m, 2H, diast. A+B), 1.40 – 1.22 (m, 12H, diast. A+B), 1.07 (d, *J* = 7.1 Hz, 6H, diast. A+B), 0.92 – 0.88 (m, 6H, diast. A+B) ppm. **^{13}C{^{1}H} NMR** (101 MHz, $CDCl_3$) δ 216.2 (diast. A), 216.1 (diast. B), 123.9 (q, *J* = 283.0 Hz), 80.5 (q, *J* = 30.4 Hz, diast. B), 79.2 (q, *J* = 30.6 Hz, diast. A), 33.6, 33.5, 32.8, 31.3, 29.3, 29.0, 22.8, 22.7, 19.5, 15.3 (q, *J* = 1.9 Hz), 14.6 (q, *J* = 1.9 Hz), 14.1 ppm. **^{19}F NMR** (376 MHz, $CDCl_3$) δ -71.7 (d, *J* = 7.3 Hz, diast. A), -72.6 (d, *J* = 7.5 Hz, diast. B) ppm. **HRMS-APCI** m/z calculated for $C_{10}H_{17}F_3OS_2$ $[M+H]^+$ 275.0746, found 275.0751.

S-Methyl *O*-(1,1,1-trifluoro-3-ethylhexan-2-yl) carbonodithioate (16')

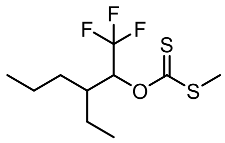

16'

2-ethylpentanal was prepared following the described procedure I from Wang *et al.*[9] Alcohol was prepared following the general procedure from 2-ethylpentanal (1.1 g, 10.0 mmol) and $TMSCF_3$ (1.8 mL, 12.0 mmol). Xanthate was prepared from the corresponding alcohol (1.5 g, 8.1 mmol), NaH (625 mg, 15.6 mmol), imidazole (20.3 mg, 0.3 mmol), CS_2 (0.9 mL, 15.6 mmol) and MeI (0.9 mL, 15.6 mmol). Pale yellow liquid (1.4 g, 51% (2 steps)).

^{1}H NMR (400 MHz, $CDCl_3$) δ 6.23 – 6.14 (m, 2H, diast. A+B), 2.61 (s, 6H, diast. A+B), 1.98 – 1.85 (m, 2H, diast. A+B), 1.73 – 1.23 (m, 12H, diast. A+B), 0.97 (dt, *J* = 7.5, 3.7 Hz, 6H, diast. A+B), 0.94 – 0.89 (m, 6H, diast. A+B) ppm. **^{13}C{^{1}H} NMR** (101 MHz, $CDCl_3$) δ 216.1, 124.0 (q, *J* = 282.9 Hz), 78.5 (q, *J* = 30.5 Hz, diast. B), 78.4 (q, *J* = 30.5 Hz, diast. A), 39.8, 39.6, 31.4, 30.9, 22.7, 21.8, 20.4, 20.2, 19.5, 14.3, 14.2, 11.5 ppm. **^{19}F NMR** (376 MHz, $CDCl_3$) δ -

72.3 (d, J = 6.6 Hz, diast. A), -72.4 (d, J = 7.3 Hz, diast. B) ppm. **HRMS-ESI** m/z calculated for $C_{10}H_{17}F_3OS_2$ $[M+H]^+$ 275.0746, found 275.0756.

S-Methyl O-(1-cyclohexyl-2,2,2-trifluoroethyl) carbonodithioate (17')

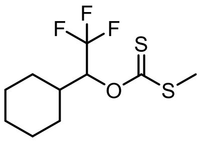

17'

Alcohol was prepared following the general procedure I from cyclohexanecarbaldehyde (1.2 mL, 10.0 mmol) and $TMSCF_3$ (1.8 mL, 12.0 mmol). Xanthate was prepared from the corresponding alcohol (1.7 g, 9.3 mmol), NaH (933 mg, 23.3 mmol), imidazole (31.8 mg, 0.5 mmol), CS_2 (1.4 mL, 23.3 mmol) and MeI (1.45 mL, 23.3 mmol). Pale yellow liquid (1.56 g, 57% (2 steps)).

^{1}H NMR (400 MHz, $CDCl_3$) δ 6.20 – 6.06 (dq, J = 7.2, 7.2 Hz, 1H), 2.61 (s, 3H), 2.05 – 1.94 (m, 1H), 1.87 – 1.74 (m, 4H), 1.70 – 1.64 (m, 1H), 1.39 – 1.03 (m, 5H) ppm. **^{13}C{^{1}H} NMR** (101 MHz, $CDCl_3$) δ 216.3, 123.7 (q, J = 282.8 Hz), 80.4 (q, J = 30.6 Hz), 38.1, 29.0, 27.6, 25.9, 19.6 ppm. **^{19}F NMR** (376 MHz, $CDCl_3$) δ -72.3 (d, J = 7.3 Hz) ppm. **HRMS-ESI** m/z calculated for $C_{10}H_{15}F_3OS_2$ $[M+H]^+$ 273.0589, found 273.0588.

1,1,1-trifluorobutane (4)

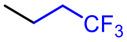

4

An authentic sample was obtained from Apollo Scientific. Colorless gas.

^{1}H NMR (400 MHz, CD_2Cl_2) δ 2.13 – 2.00 (m, 2H), 1.63 – 1.54 (m, 2H), 0.99 (td, J = 7.5, 0.8 Hz, 3H) ppm. **^{13}C{^{1}H} NMR** (101 MHz, CD_2Cl_2) δ 128.3 (q, J = 276.0 Hz), 36.3 (q, J = 28.1 Hz), 16.3 (q, J = 3.2 Hz), 13.7 ppm. **^{19}F NMR** (376 MHz, CD_2Cl_2) δ -67.2 (t, J = 11.1 Hz) ppm.

1,1,1-trifluoropentane (5)

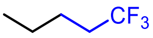

5

Following the general procedure II, 5-iodo-1,1,1-trifluoropentane (0.58 mL, 4.0 mmol) was mixed with zinc (519 mg, 8.0 mmol) in DMA (4 mL). Then, water (0.21 mL, 12.0 mmol) was added to the reaction mixture. The product was purified by distillation at 47 °C/ 760mmHg. Colorless oil (63 mg, 13 %).

^{1}H NMR (400 MHz, $CDCl_3$) δ 2.17 – 1.98 (m, 2H), 1.60 – 1.48 (m, 2H), 1.40 (h, J = 7.4 Hz, 2H), 0.94 (t, J = 7.3 Hz, 3H) ppm. **^{13}C{^{1}H} NMR** (101 MHz, $CDCl_3$) δ 127.5 (q, J = 276.2 Hz), 33.6 (q, J = 28.2 Hz), 24.0 (q, J = 2.9 Hz), 22.0, 13.8 ppm. **^{19}F NMR** (376 MHz, $CDCl_3$) δ -66.5 (t, J = 11.1 Hz) ppm.

1,1,1-trifluoro-3-methylbutane (6)

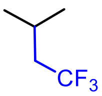

6

Following the general procedure II, 4-bromo-1,1,1-trifluoro-3-methylbutane (0.68 mL, 4.9 mmol) was mixed with zinc (638 mg, 9.8 mmol) in DMA (5 mL). Then, water (0.26 mL, 14.6 mmol) was

added to the reaction mixture. Traces of the product was obtained by distillation at 35 °C/760 mmHg.

^{1}H NMR (400 MHz, $CDCl_3$) δ 2.05 – 1.91 (m, 3H), 1.01 (d, *J* = 5.8 Hz, 6H) ppm. **^{13}C{^{1}H} NMR** (101 MHz, $CDCl_3$) δ 127.3 (q, *J* = 277.3 Hz), 42.0 (q, *J* = 26.9 Hz), 23.2 (q, *J* = 2.5 Hz), 22.8 ppm. **^{19}F NMR** (376 MHz, $CDCl_3$) δ -63.7 (t, *J* = 11.1 Hz) ppm.

1,1,1-trifluorohexane (7)

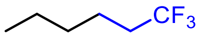

7

Following the general procedure II, 6-bromo-1,1,1-trifluorohexane (0.67 mL, 4.6 mmol) was mixed with zinc (597 mg, 9.1 mmol) in DMA (5 mL). Then, water (0.25 mL, 13.7 mmol) was added to the reaction mixture. The product was purified by distillation at 89 °C/760 mmHg. Colorless oil (288 mg, 45%).

^{1}H NMR (400 MHz, $CDCl_3$) δ 2.14 – 1.97 (m, 2H), 1.59 – 1.52 (m, 2H), 1.37 – 1.33 (m, 4H), 0.91 (t, *J* = 7.3 Hz, 3H) ppm. **^{13}C{^{1}H} NMR** (101 MHz, $CDCl_3$) δ 127.5 (q, *J* = 276.2 Hz), 33.9 (q, *J* = 28.3 Hz), 31.0, 22.4, 21.7 (q, *J* = 2.9 Hz), 13.9 ppm. **^{19}F NMR** (282 MHz, $CDCl_3$) δ -67.5 (t, *J* = 10.9 Hz) ppm.

1,1,1-trifluoro3-methylpentane (8)

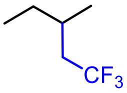

8

Following the general procedure I from *S*-Methyl *O*-(1,1,1-trifluoro-3-methylpentan-2-yl) carbonodithioate **8'** (197 mg, 0.8 mmol), sodium formate (326 mg), $(Bu_4N)_2S_2O_8$ (1.62 g) and DMF (5 mL).

^{1}H NMR (500 MHz, C_6D_{12}) δ 2.12 – 2.00 (m, 1H), 1.88 – 1.74 (m, 2H), 1.49 – 1.41 (m, 1H), 1.35 – 1.23 (m, 1H), 1.02 (dq, *J* = 6.6, 0.9 Hz, 3H), 0.92 (t, *J* = 7.4 Hz, 3H) ppm. **^{13}C{^{1}H} NMR** (126 MHz, C_6D_{12}) δ 126.8 (q, *J* = 277.0 Hz), 39.9 (q, *J* = 27.3 Hz), 29.4, 29.1, 18.7, 10.2 ppm. **^{19}F NMR** (282 MHz, C_6D_{12}) δ -64.8 (t, *J* = 10.8 Hz) ppm.

1,1,1-trifluoro-4-methylpentane (9)

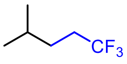

9

Following the general procedure I from *S*-methyl *O*-(1,1,1-trifluoro-4-methylpentan-2-yl) carbonodithioate **9'** (197 mg, 0.8 mmol), sodium formate (326 mg) and $(Bu_4N)_2S_2O_8$ (1.62 g) and DMF (4 mL).

^{1}H NMR (500 MHz, C_6D_{12}) δ 2.04 – 1.91 (m, 2H), 1.63 – 1.52 (m, 1H), 1.48 – 1.40 (m, 2H), 0.92 (d, *J* = 6.6 Hz, 6H) ppm. **^{13}C NMR** (126 MHz, C_6D_{12}) δ 127.9 (q, *J* = 275.7 Hz), 32.8 (q, *J* = 29.0 Hz), 31.5 (q, *J* = 2.6 Hz), 28.3, 22.5 ppm.**^{19}F NMR** (282 MHz, C_6D_{12}) δ -67.4 (t, *J* = 10.6 Hz) ppm.

1,1,1-trifluoro-3,3-dimethylbutane (10)

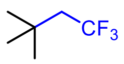

10

Following the general procedure I from *S*-methyl *O*-(1,1,1-trifluoro-3,3-dimethylbutan-2-yl) carbonodithioate **10'** (197 mg, 0.8 mmol), sodium formate (326 mg) and $(Bu_4N)_2S_2O_8$ (1.62 g) and DMF (4 mL).

^{1}H NMR (500 MHz, C_6D_{12}) δ 1.93 (q, *J* = 11.7 Hz, 2H), 1.04 (q, *J* = 1.0 Hz, 9H) ppm. **^{13}C{^{1}H} NMR** (126 MHz, C_6D_{12}) δ 127.6 (q, *J* = 278.3 Hz), 46.7 (q, *J* = 26.4 Hz), 30.2 (q, *J* = 1.4 Hz), 28.9 (q, *J* = 2.3 Hz) ppm. **^{19}F NMR** (282 MHz, C_6D_{12}) δ -61.2 (t, *J* = 12.8 Hz) ppm.

1,1,1-trifluoro-heptane (11)

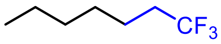

11

Following the general procedure I from using *S*-Methyl *O*-(1,1,1-trifluoroheptan-2-yl) carbonodithioate **11'** (208 mg, 0.8 mmol), sodium formate (326 mg), $(Bu_4N)_2S_2O_8$ (1.62 g) and DMF (5 mL).

^{1}H NMR (500 MHz, C_6D_{12}) δ 2.03 – 1.89 (m, 2H), 1.59 – 1.48 (m, 2H), 1.38 – 1.26 (m, 6H), 0.91 (t, *J* = 7.0 Hz, 3H) ppm. **^{13}C{^{1}H} NMR** (126 MHz, C_6D_{12}) δ 127.7 (q, *J* = 276.0 Hz), 34.7 (q, *J* = 28.7 Hz), 32.3, 29.3, 23.2, 22.7 (q, *J* = 3.0 Hz), 14.3 ppm. **^{19}F NMR** (282 MHz, C_6D_{12}) δ -67.8 (t, *J* = 10.7 Hz) ppm.

1,1,1-trifluoro-3-methylhexane (12)

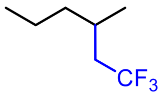

12

Following the general procedure I from using *S*-Methyl *O*-(1,1,1-trifluoro-3-methylhexan-2-yl) carbonodithioate **12'** (208 mg, 0.8 mmol), sodium formate (326 mg), $(Bu_4N)_2S_2O_8$ (1.62 g) and DMF (5 mL).

^{1}H NMR (500 MHz, C_6D_{12}) δ 2.07 – 1.98 (m, 1H), 1.89 – 1.80 (m, 1H), 1.80 – 1.74 (m, 1H), 1.37 – 1.27 (m, 3H), 1.26 – 1.18 (m, 1H), 0.99 (d, *J* = 6.5 Hz, 3H), 0.91 (t, *J* = 7.1 Hz, 3H) ppm. **^{13}C{^{1}H} NMR** (126 MHz, C_6D_{12}) δ 127.8 (q, *J* = 277.0 Hz), 41.2 (q, *J* = 27.4 Hz), 40.1, 28.3 (q, *J* = 2.3 Hz), 20.6, 20.1, 14.3 ppm. **^{19}F NMR** (282 MHz, C_6D_{12}) δ -64.9 (t, *J* = 10.8 Hz) ppm.

1,1,1-trifluoroethylpentane (13)

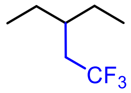

13

Following the general procedure I from *S*-Methyl *O*-(1,1,1-trifluoro-3-ethylpentan-2-yl) carbonodithioate **13'** (208 mg, 0.8 mmol), sodium formate (326 mg), $(Bu_4N)_2S_2O_8$ (1.62 g) and DMF (5 mL).

^{1}H NMR (500 MHz, C_6D_{12}) δ 1.97 (qd, *J* = 11.3, 6.3 Hz, 2H), 1.64 (hept, *J* = 6.2 Hz, 1H), 1.47 – 1.38 (m, 4H), 0.90 (t, *J* = 7.5 Hz, 6H) ppm. **^{13}C{^{1}H} NMR** (126 MHz, C_6D_{12}) δ 127.0 (q, *J* = 276.6 Hz), 36.7 (q, *J* = 27.4 Hz), 35.0 (q, *J* = 2.0 Hz), 29.6, 9.6 ppm. **^{19}F NMR** (470 MHz, C_6D_{12}) δ -64.8 (t, *J* = 11.3 Hz) ppm.

1,1,1-trifluoro-octane (14)

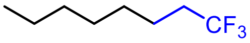

14

Following the general procedure I from *S*-Methyl *O*-(1,1,1-trifluorooctan-2-yl) carbonodithioate **14'** (219 mg, 0.8 mmol), sodium formate (326 mg), $(Bu_4N)_2S_2O_8$ (1.62 g) and DMF (5 mL).

^{1}H NMR (500 MHz, C_6D_{12}) δ 2.05 – 1.92 (m, 2H), 1.63 – 1.52 (m, 2H), 1.39 – 1.30 (m, 8H), 0.92 (t, *J* = 7.1 Hz, 3H) ppm. **^{13}C{^{1}H} NMR** (126 MHz, C_6D_{12}) δ 126.7 (q, *J* = 276.0 Hz), 33.8 (q, *J* = 28.8 Hz), 31.5, 28.8, 28.7, 22.5, 21.8 (q, *J* = 2.8 Hz), 13.4 ppm. **^{19}F NMR** (282 MHz, C_6D_{12}) δ -67.8 (t, *J* = 10.5 Hz) ppm.

1,1,1-trifluoro-3-methylheptane (15)

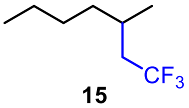

15

Following the general procedure I from *S*-Methyl *O*-(1,1,1-trifluoro-3-methylheptan-2-yl) carbonodithioate **15'** (219 mg, 0.8 mmol), sodium formate (326 mg), $(Bu_4N)_2S_2O_8$ (1.62 g) and DMF (5 mL).

^{1}H NMR (500 MHz, C_6D_{12}) δ 2.10 – 1.97 (m, 1H), 1.88 – 1.74 (m, 2H), 1.36 – 1.19 (m, 6H), 1.00 (d, *J* = 6.6 Hz, 3H), 0.91 (t, *J* = 7.0 Hz, 3H) ppm. **^{13}C{^{1}H} NMR** (126 MHz, C_6D_{12}) δ 127.8 (q, *J* = 277.0 Hz), 41.2 (q, *J* = 27.3 Hz), 37.6, 29.7, 28.6 (q, *J* = 2.3 Hz), 23.6, 20.1, 14.3 ppm. **^{19}F NMR** (282 MHz, C_6D_{12}) δ -64.7 (t, *J* = 10.8 Hz) ppm.

1,1,1-trifluoro-3-ethylhexane (16)

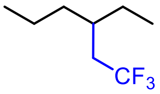

16

Following the general procedure I from *S*-Methyl *O*-(1,1,1-trifluoro-3-ethylhexan-2-yl) carbonodithioate **16'** (219 mg, 0.8 mmol), sodium formate (326 mg), $(Bu_4N)_2S_2O_8$ (1.62 g) and DMF (5 mL).

^{1}H NMR (500 MHz, C_6D_{12}) δ 1.94 (qd, *J* = 11.3, 6.5 Hz, 1H), 1.94 (qd, *J* = 11.3, 6.0 Hz, 1H), 1.68 (hept, *J* = 6.1 Hz, 1H), 1.41 (dt, *J* = 14.2, 7.2 Hz, 2H), 1.36 – 1.26 (m, 4H), 0.91 (t, *J* = 7.1 Hz, 3H), 0.87 (t, *J* = 7.5 Hz, 3H) ppm. **^{13}C{^{1}H} NMR** (126 MHz, C_6D_{12}) δ 128.0 (q, *J* = 276.8 Hz), 38.0 (q, *J* = 27.2 Hz), 36.2, 34.3 (q, *J* = 2.0 Hz), 26.7, 20.2, 14.4, 10.5 ppm. **^{19}F NMR** (282 MHz, C_6D_{12}) δ -64.8 (t, *J* = 11.3 Hz) ppm.

1,1,1-trifluoroethylcyclohexane (17)

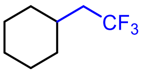

17

Following the general procedure I from *S*-Methyl *O*-(1-cyclohexyl-2,2,2-trifluoroethyl) carbonodithioate **17'** (218 mg, 0.8 mmol), sodium formate (326 mg), $(Bu_4N)_2S_2O_8$ (1.62 g) and DMF (5 mL).

^{1}H NMR (500 MHz, C_6D_{12}) δ 1.93 – 1.84 (m, 2H), 1.84 – 1.81 (m, 2H), 1.76 – 1.62 (m, 4H), 1.29 (qt, *J* = 12.5, 3.2 Hz, 2H), 1.16 (qt, *J* = 12.6, 3.2 Hz, 1H), 1.05 – 0.93 (m, 2H) ppm. **^{13}C{^{1}H} NMR** (126 MHz, C_6D_{12}) δ 127.6 (q, *J* = 277.0 Hz), 41.9 (q, *J* = 27.2 Hz), 34.1, 33.0 (q, *J* = 2.5 Hz) ppm. **^{19}F NMR** (470 MHz, C_6D_{12}) δ -64.5 (t, *J* = 11.3 Hz) ppm.

5. NMR spectra data for catalytic experiments

^{19}F NMR (376 MHz, CH_2Cl_2) spectrum of the functionalization of methane with TFDE using catalyst **1**. Fluorobenzene (▲; 0.25 mmol) was added as internal standard.

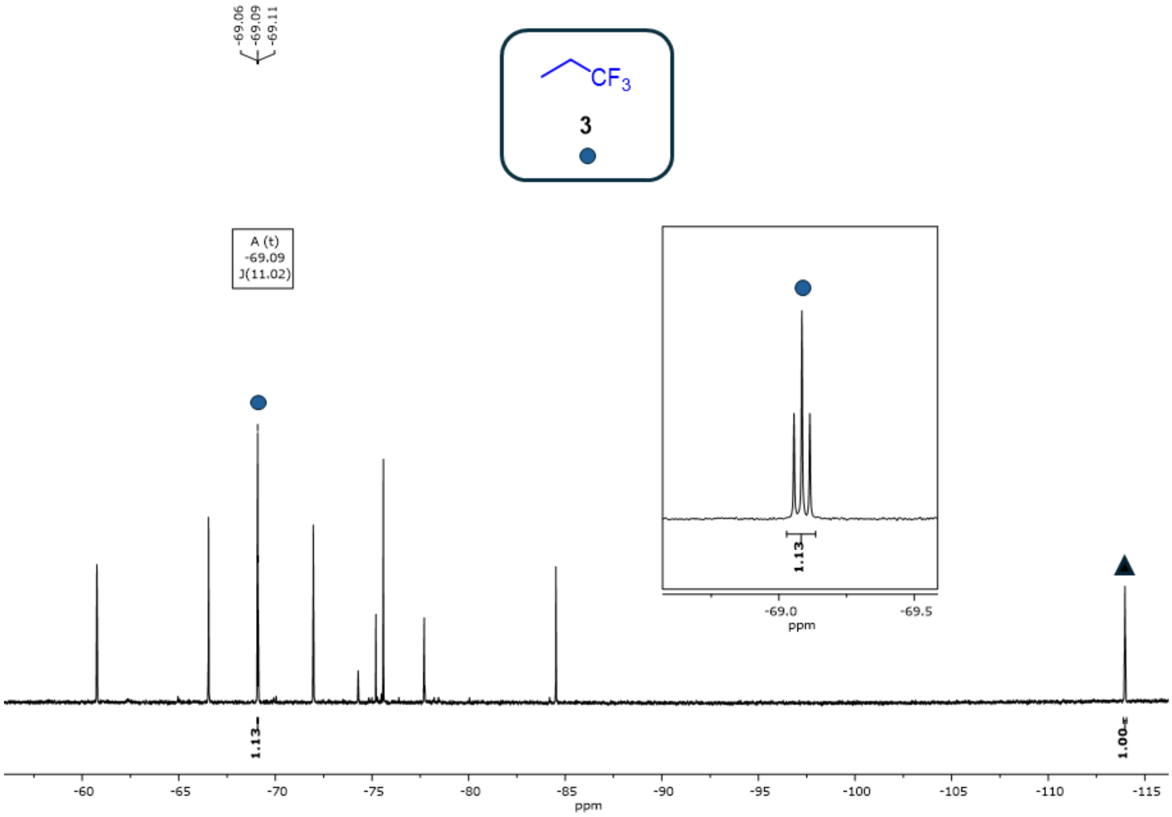

^{19}F NMR (376 MHz, CH_2Cl_2) spectrum of the functionalization of ethane with TFDE using catalyst **1**. Fluorobenzene (▲; 0.25 mmol) was added as internal standard.

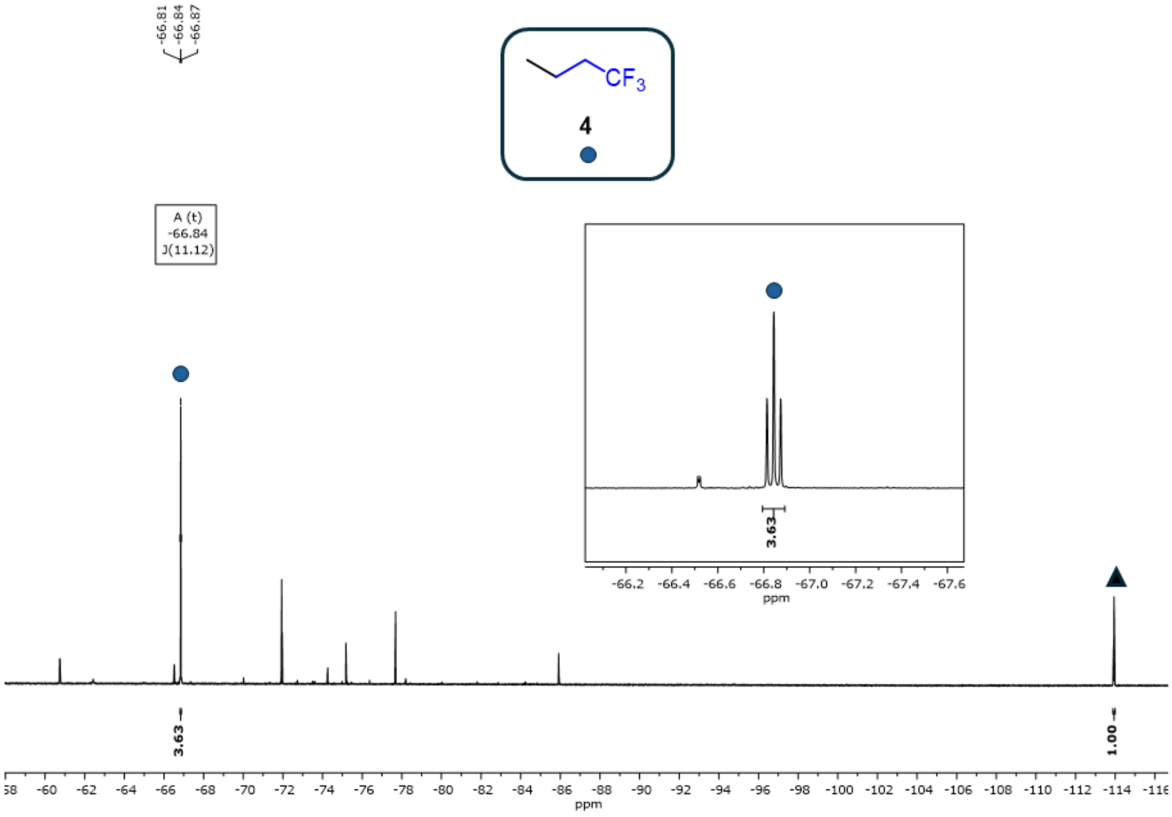

^{19}F NMR (376 MHz, CH_2Cl_2) spectrum of the functionalization of propane with TFDE using catalyst **1**. Fluorobenzene (▲; 0.50 mmol) was added as internal standard.

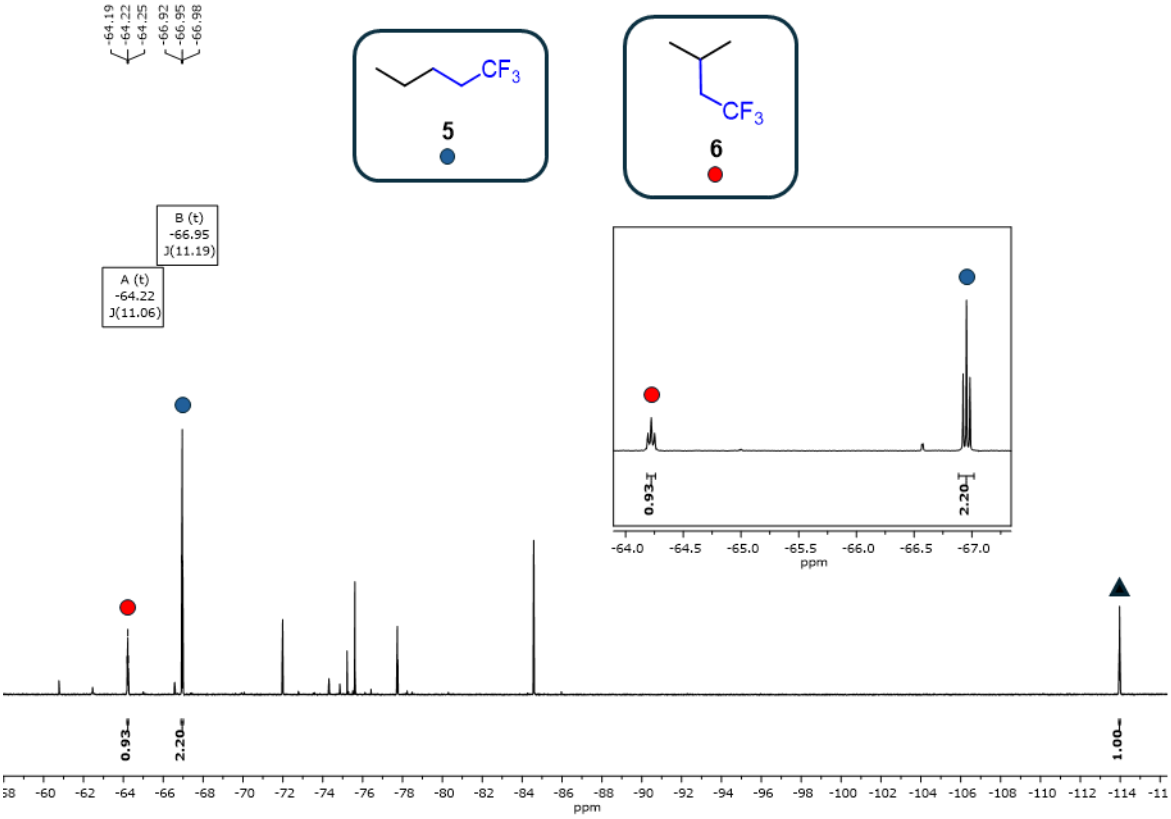

^{19}F NMR (376 MHz, CH_2Cl_2) spectrum of the functionalization of liquid n-butane with TFDE using catalyst **1**. Fluorobenzene (▲; 1.00 mmol) was added as internal standard.

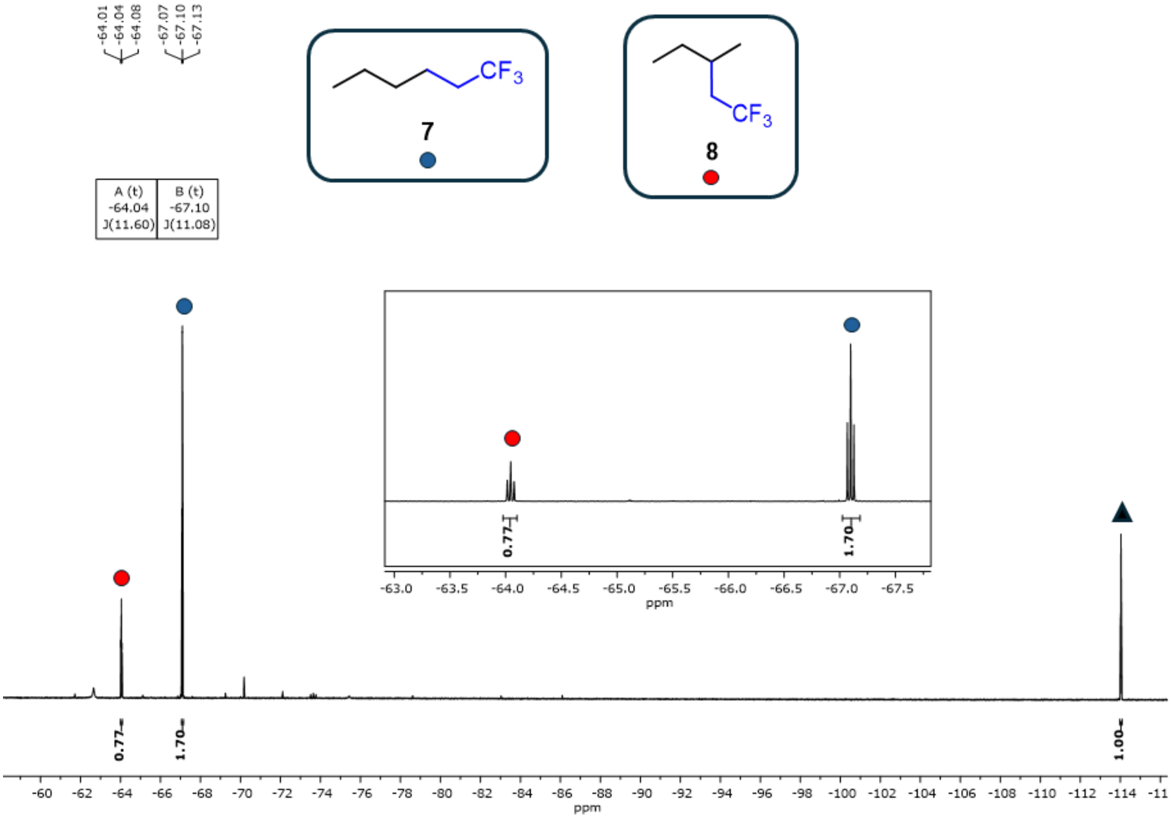

^{19}F NMR (376 MHz, CH_2Cl_2) spectrum of the functionalization of isobutane with TFDE using catalyst **1**. Fluorobenzene (▲; 1.00 mmol) was added as internal standard.

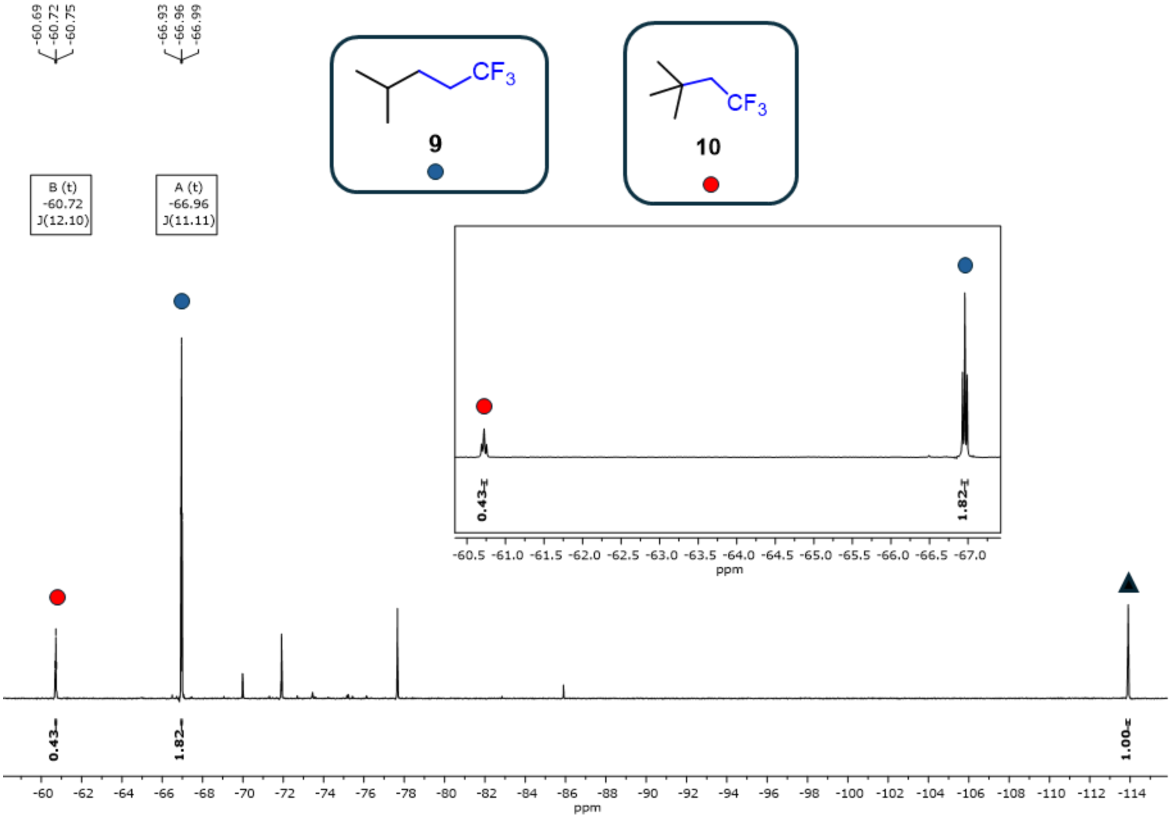

^{19}F NMR (376 MHz, C_6H_{14}) spectrum of the functionalization of n-pentane with TFDE using catalyst **1**. Fluorobenzene (▲; 1.00 mmol) was added as internal standard.

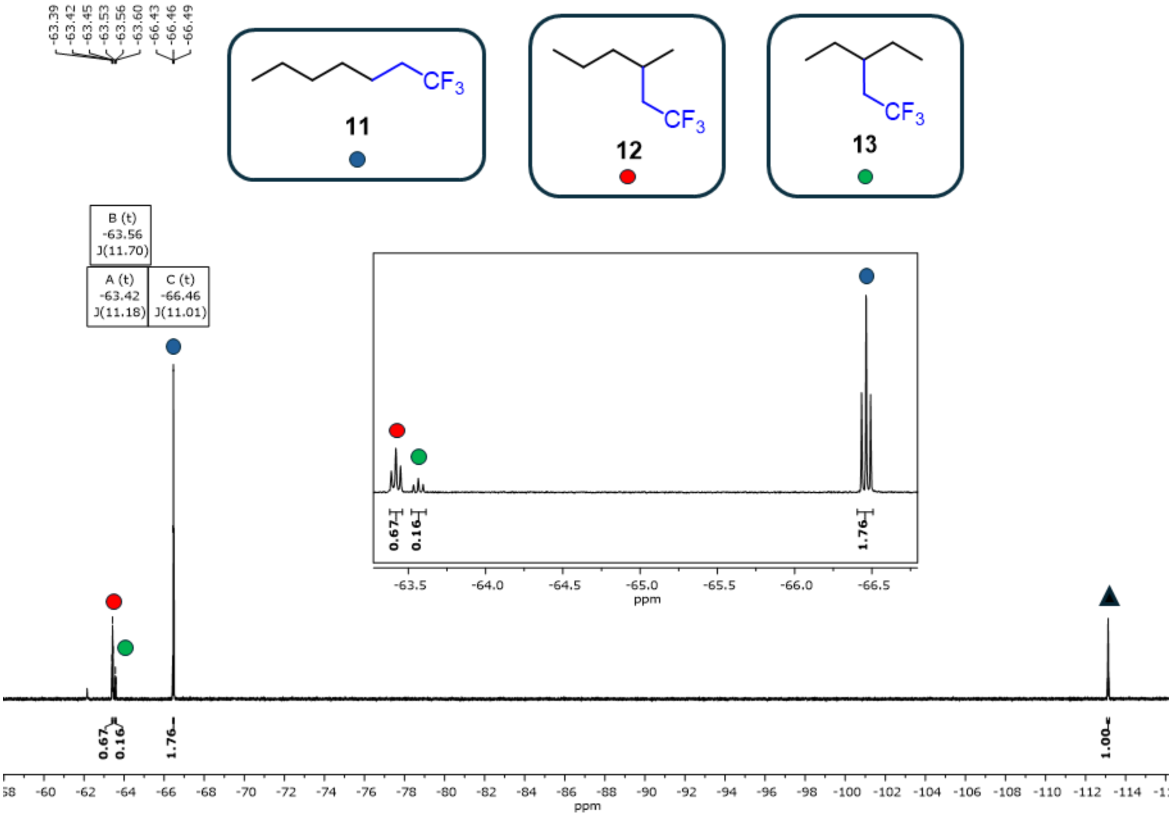

^{19}F NMR (376 MHz, C_5H_{12}) spectrum of the functionalization of n-hexane with TFDE using catalyst **1**. Fluorobenzene (▲; 1.00 mmol) was added as internal standard.

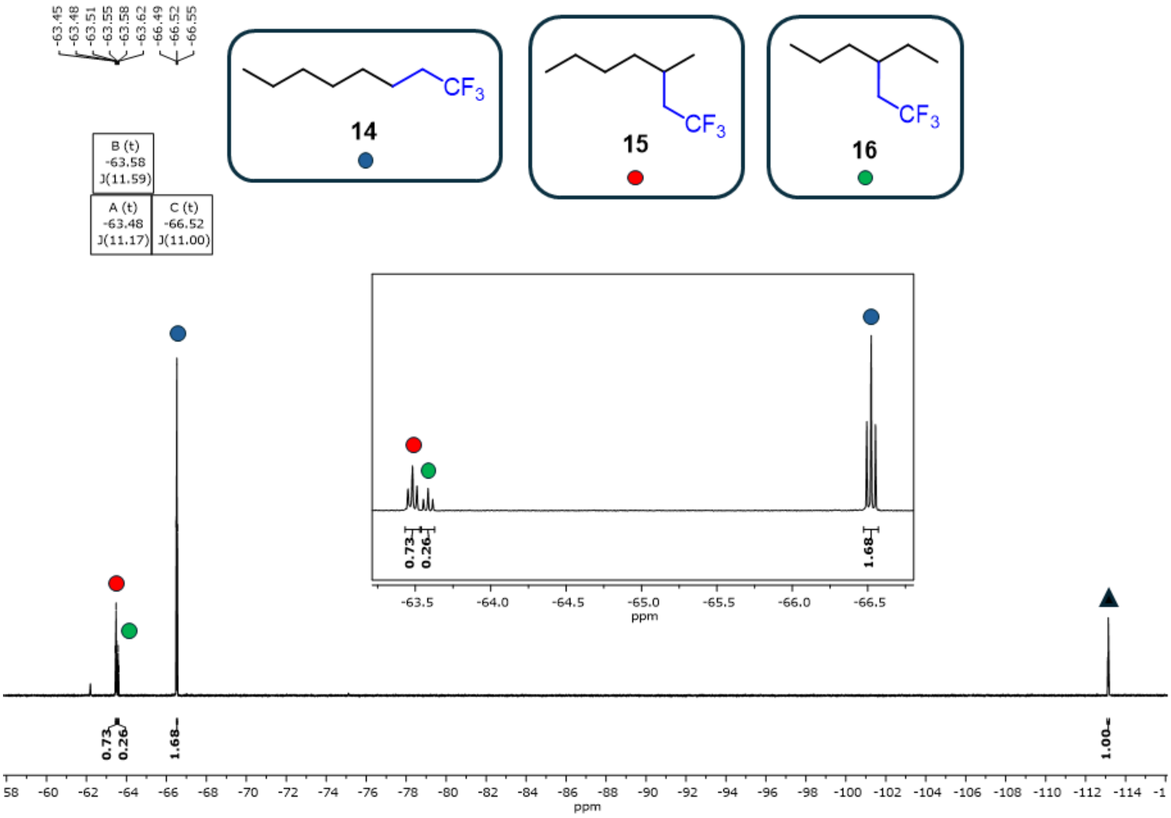

^{19}F NMR (376 MHz, C_6H_{12}) spectrum of the functionalization of cyclohexane with TFDE using catalyst **1**. Fluorobenzene (▲; 1.00 mmol) was added as internal standard.

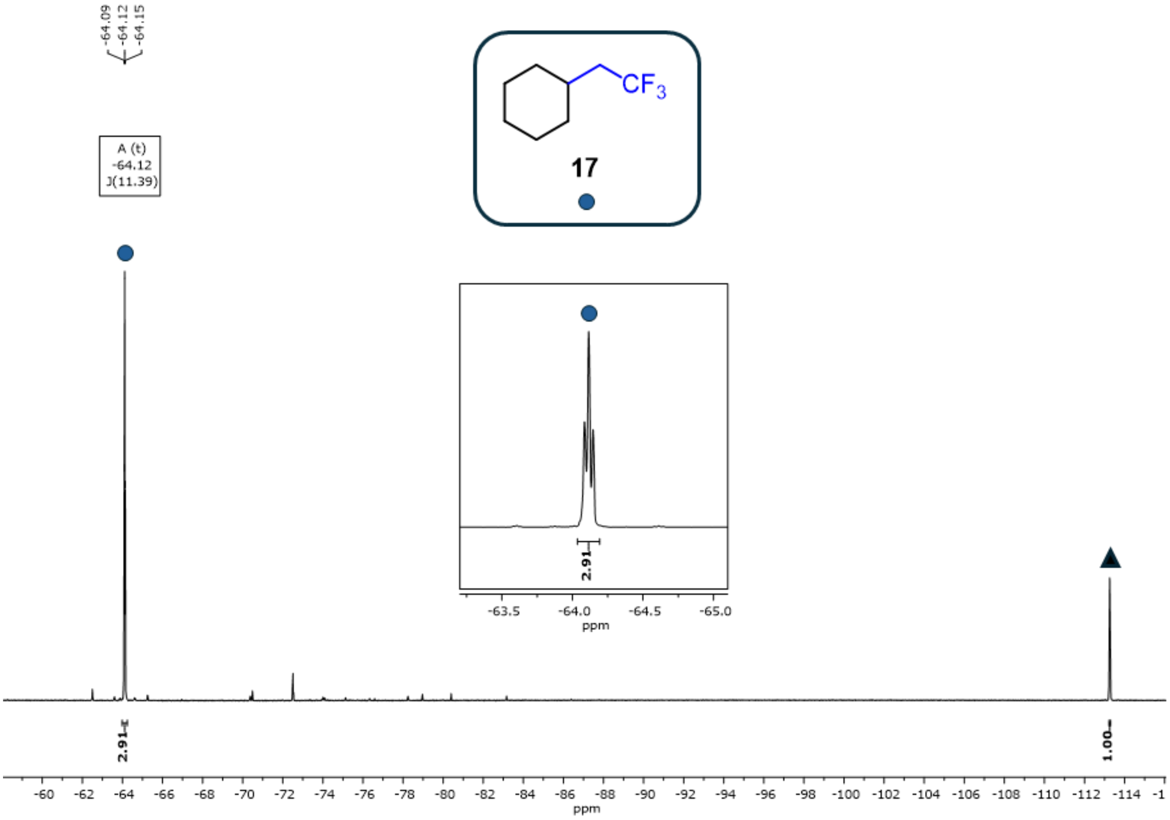

6. NMR spectra data for organic compounds

S-Methyl *O*-(1,1,1-trifluoro-3-methylpentan-2-yl) carbonodithioate (8')

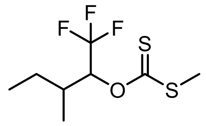

^{1}H NMR 8' (500 MHz, $CDCl_3$)

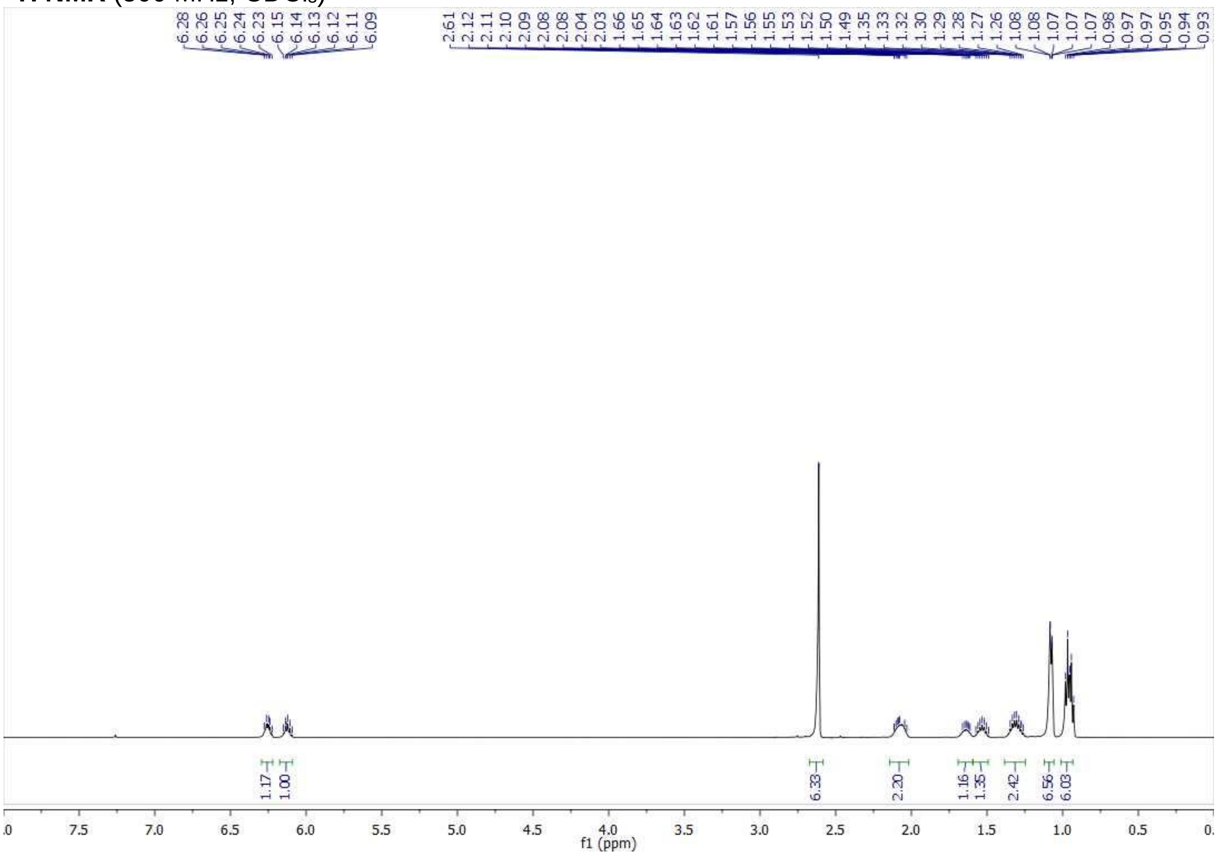

$^{13}C\{^1H\}$ NMR (126 MHz, $CDCl_3$)

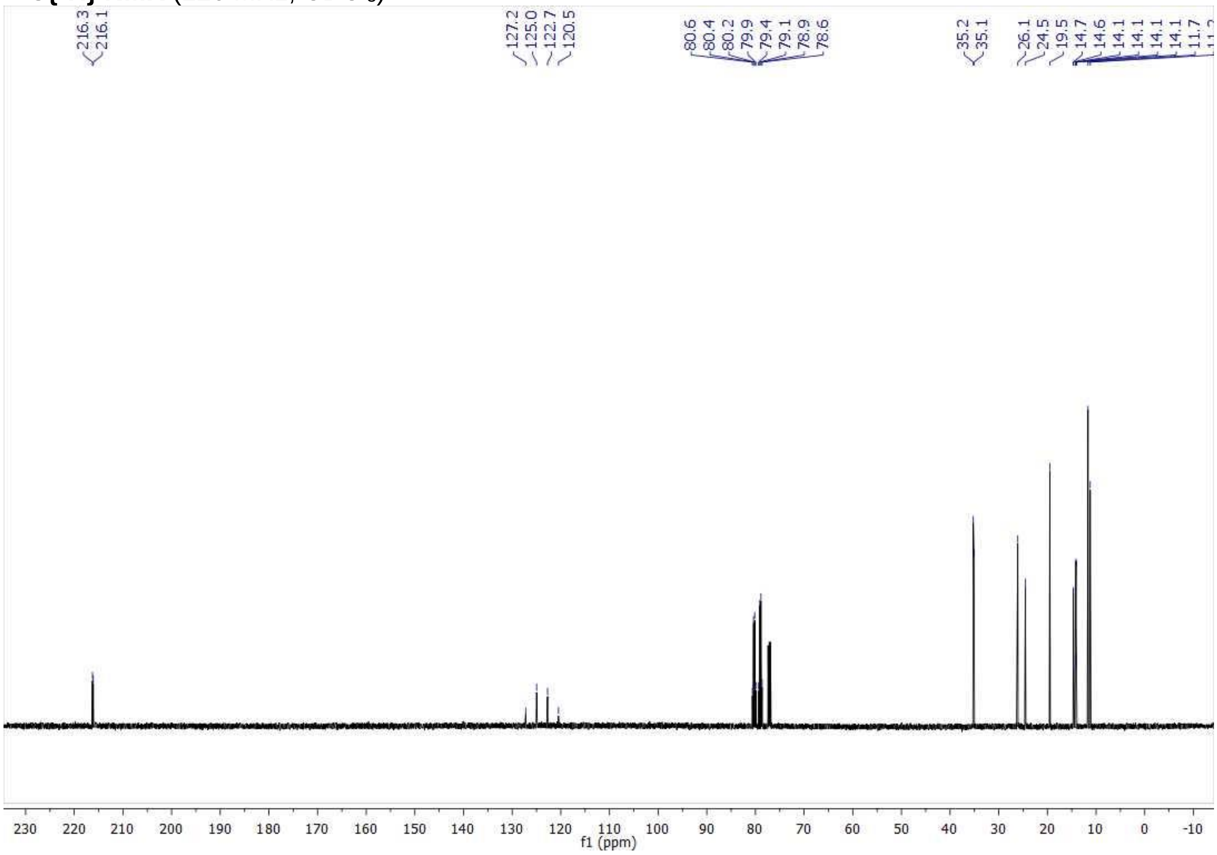

^{19}F NMR (376 MHz, $CDCl_3$)

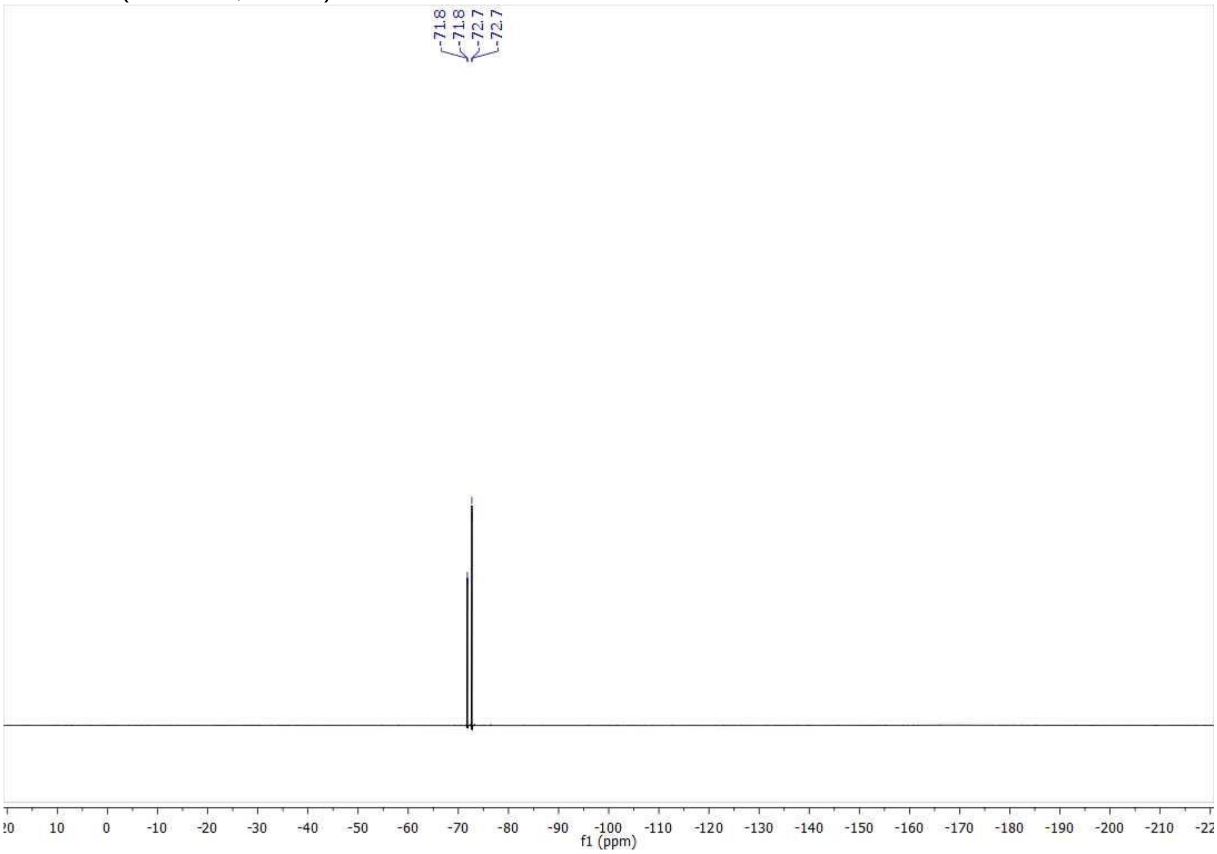

***S*-methyl *O*-(1,1,1-trifluoro-4-methylpentan-2-yl) carbonodithioate (9')**

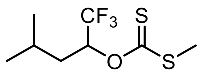

9'

^{1}H NMR (400 MHz, $CDCl_3$)

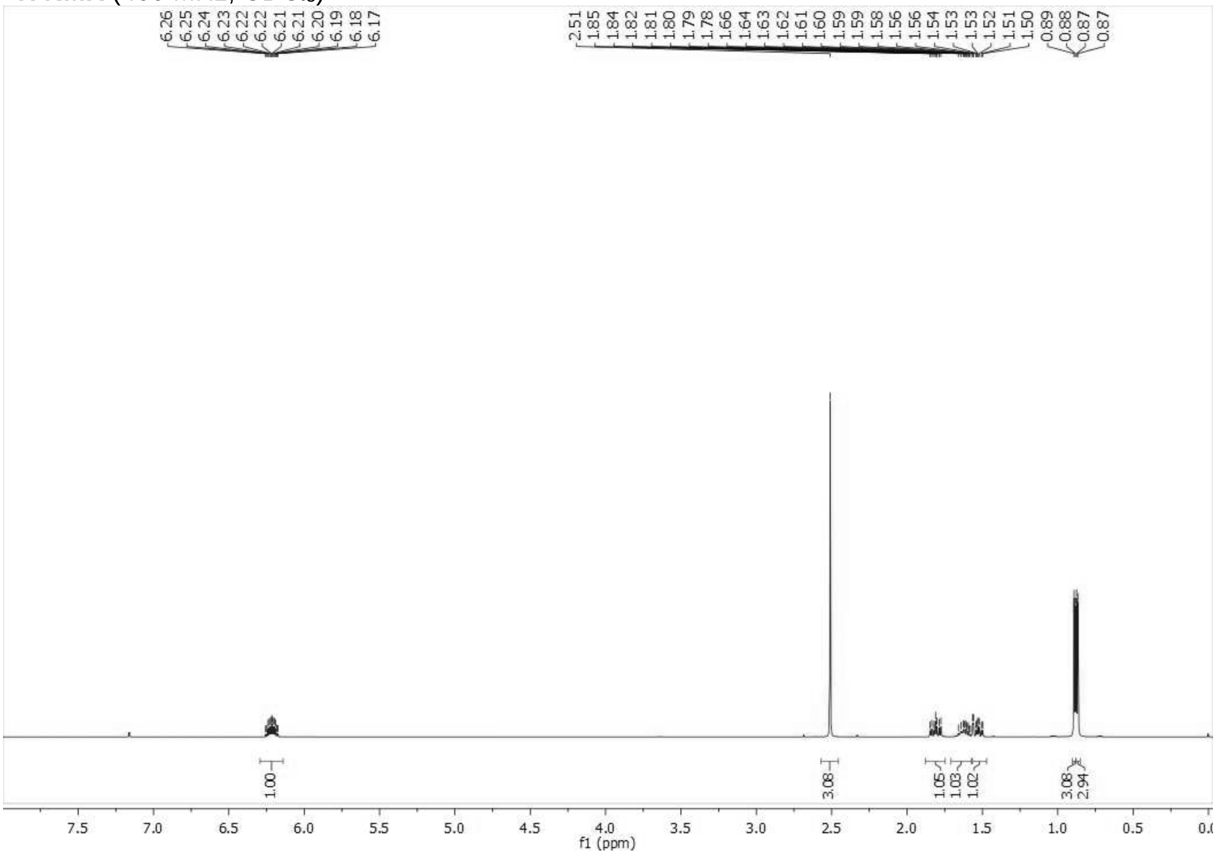

$^{13}C\{^{1}H\}$ NMR (101 MHz, $CDCl_3$)

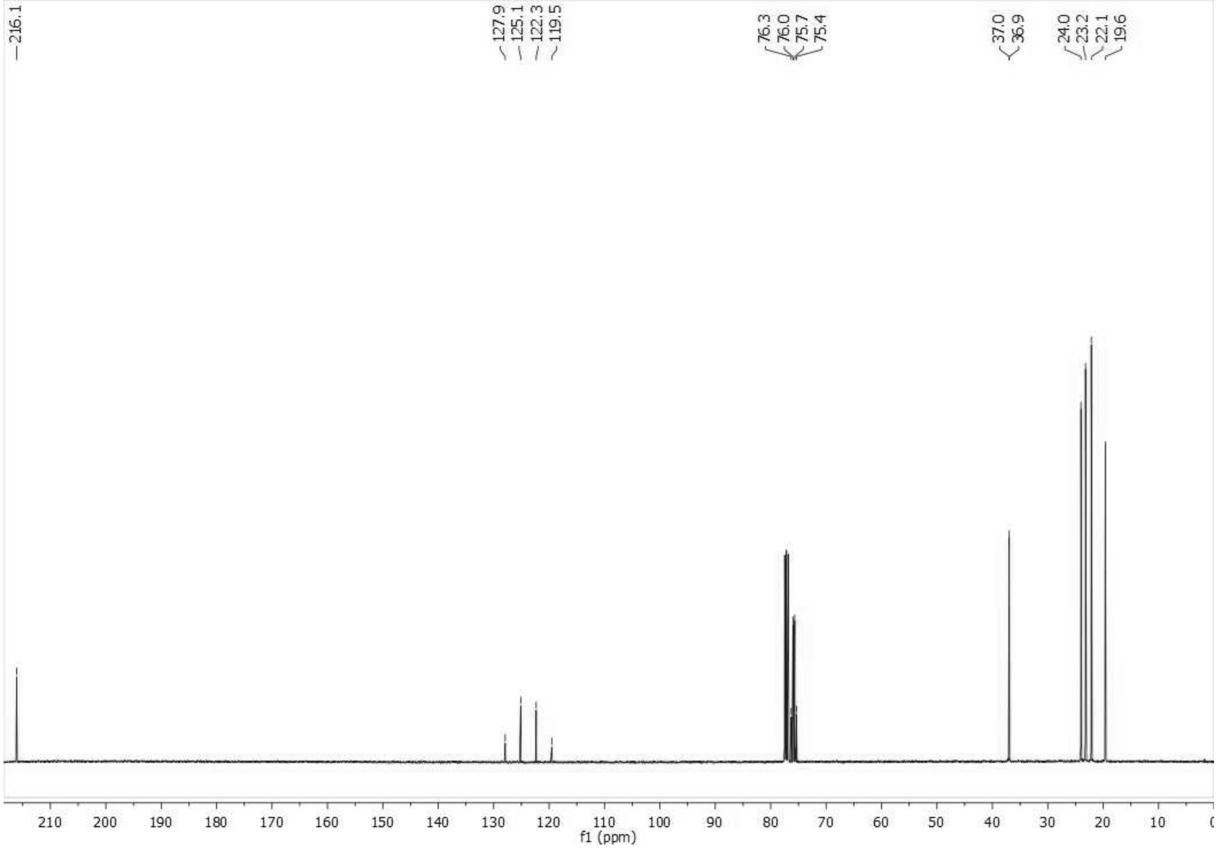

^{19}F NMR (376 MHz, $CDCl_3$)

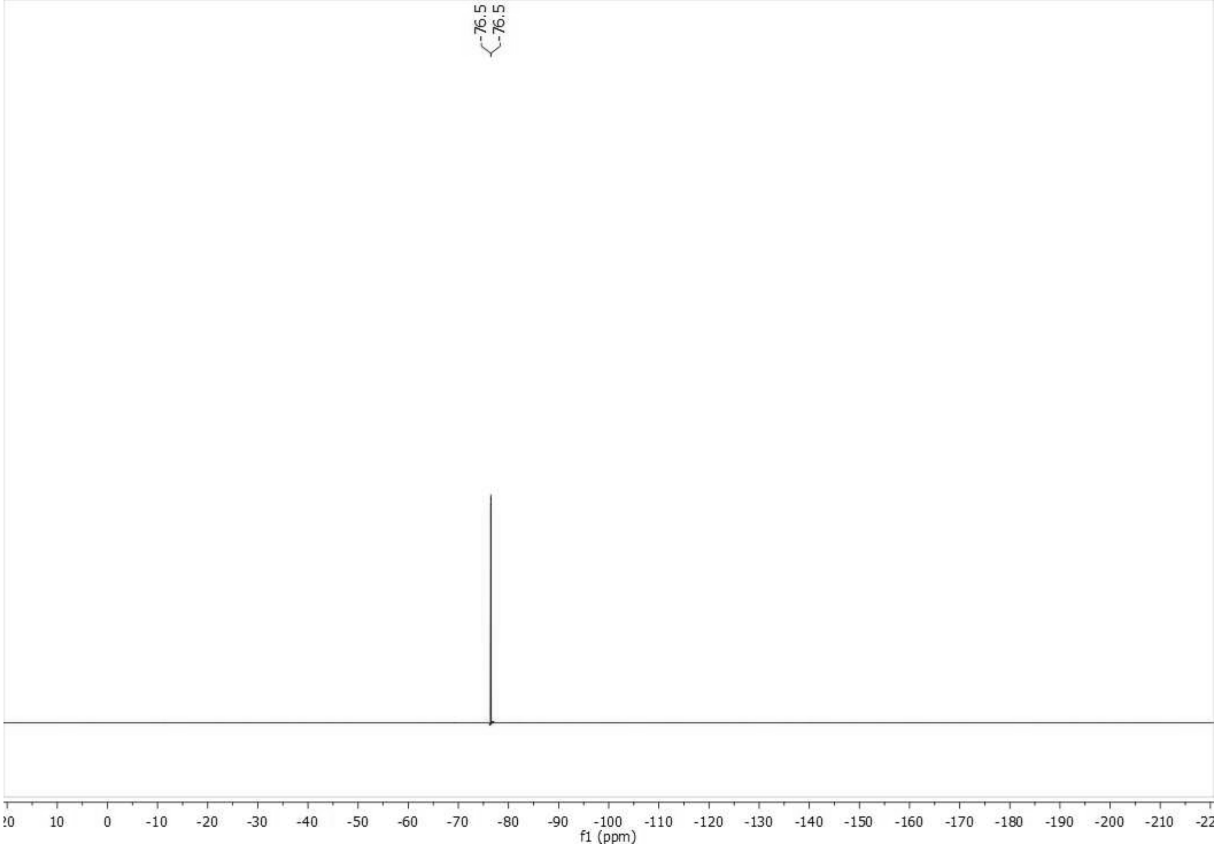

_S_-methyl _O_-(1,1,1-trifluoro-3,3-dimethylbutan-2-yl) carbonodithioate (10')

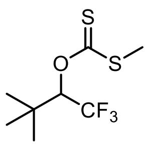

10'

^{1}H NMR (400 MHz, $CDCl_3$)

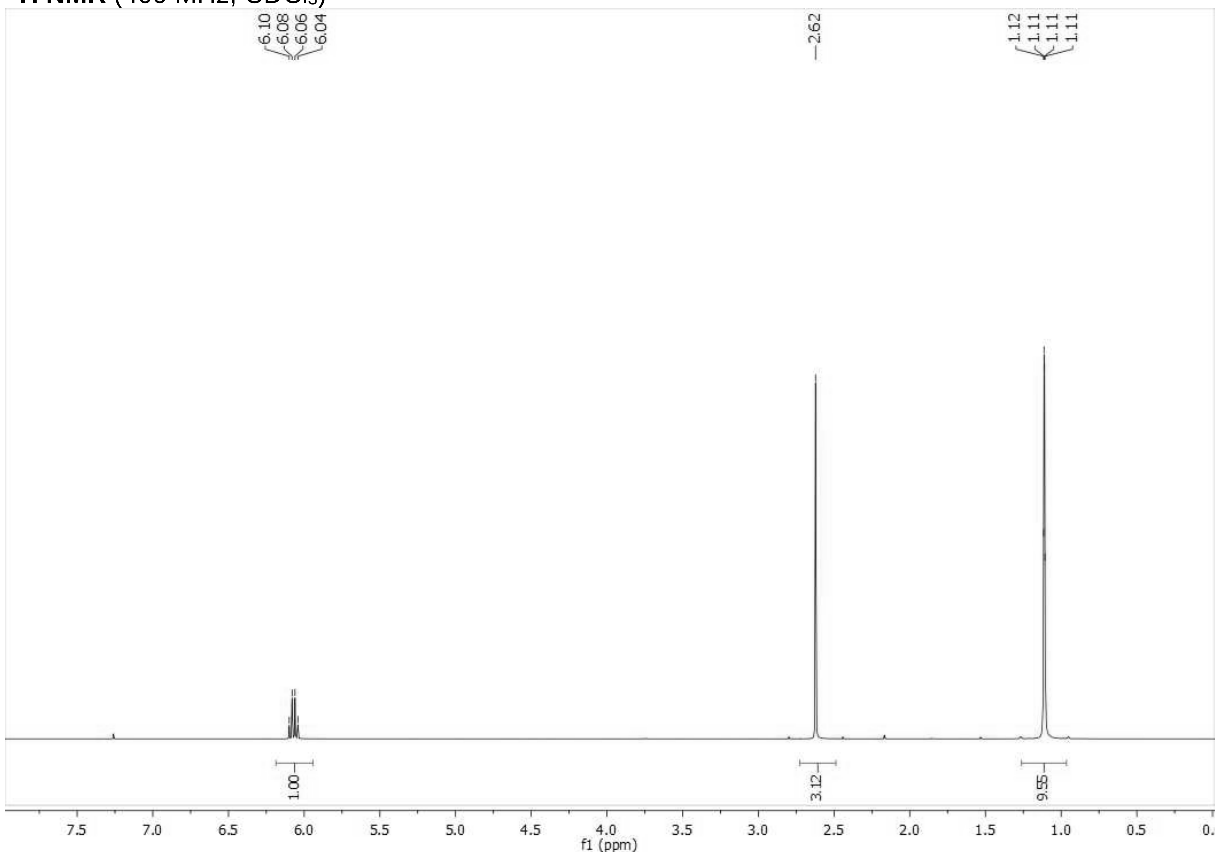

$^{13}C\{^1H\}$ NMR (101 MHz, $CDCl_3$)

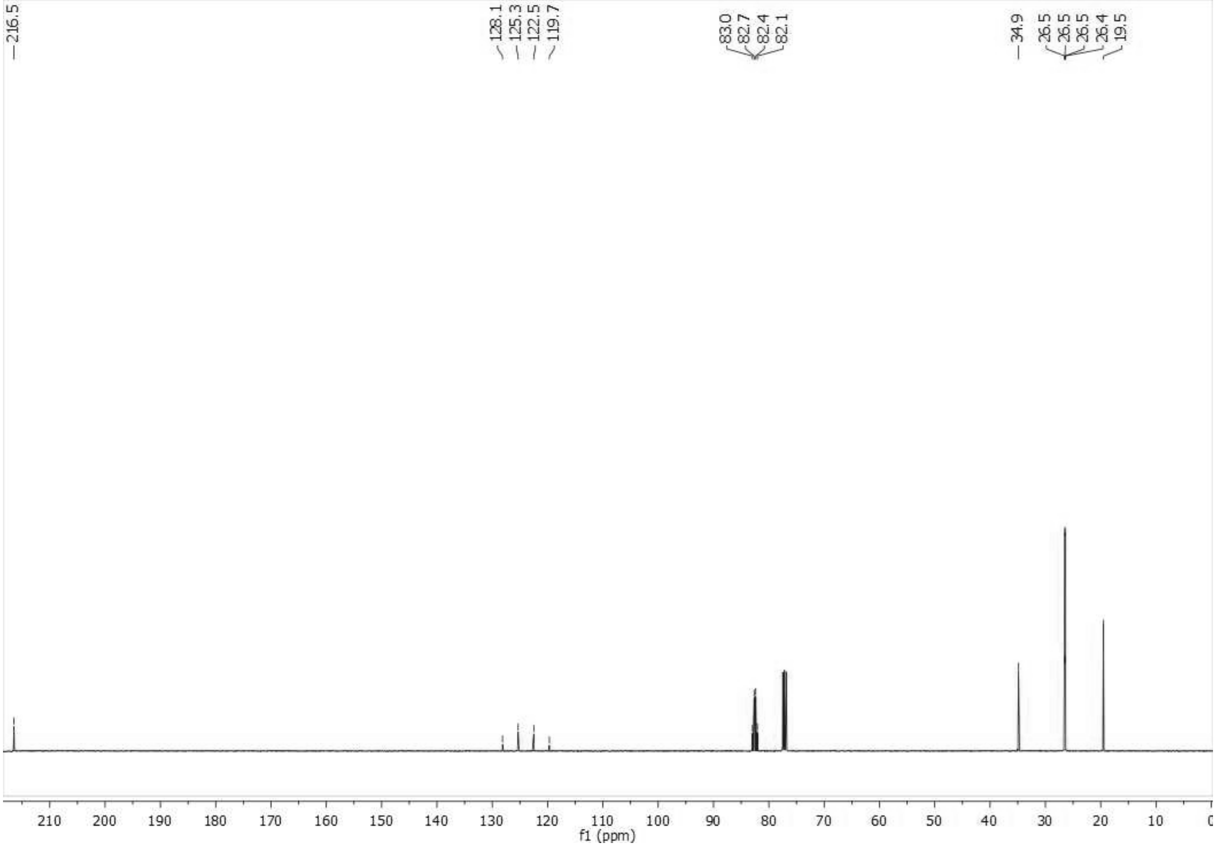

^{19}F NMR (376 MHz, $CDCl_3$)

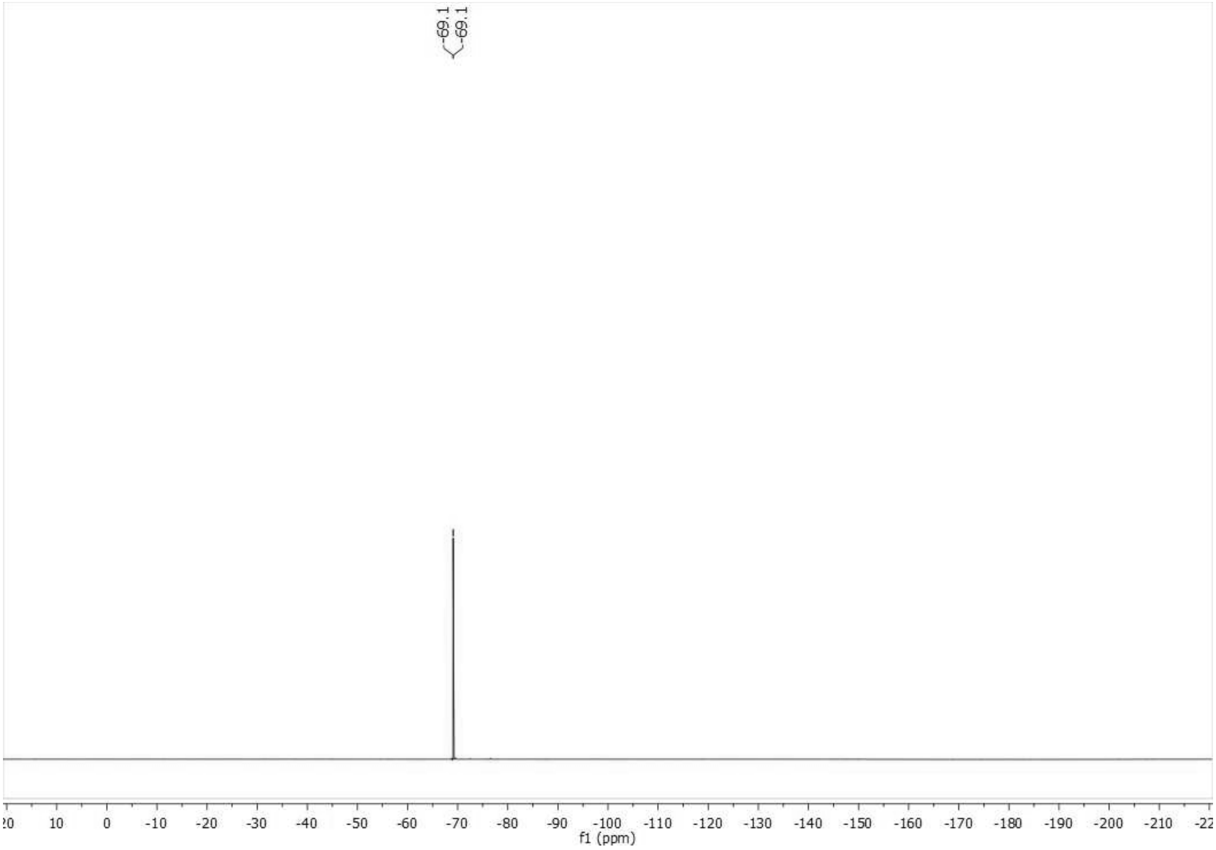

S-Methyl O-(1,1,1-trifluoroheptan-2-yl) carbonodithioate (11')

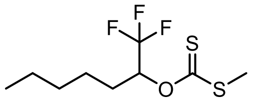

11'

^{1}H NMR (500 MHz, $CDCl_3$)

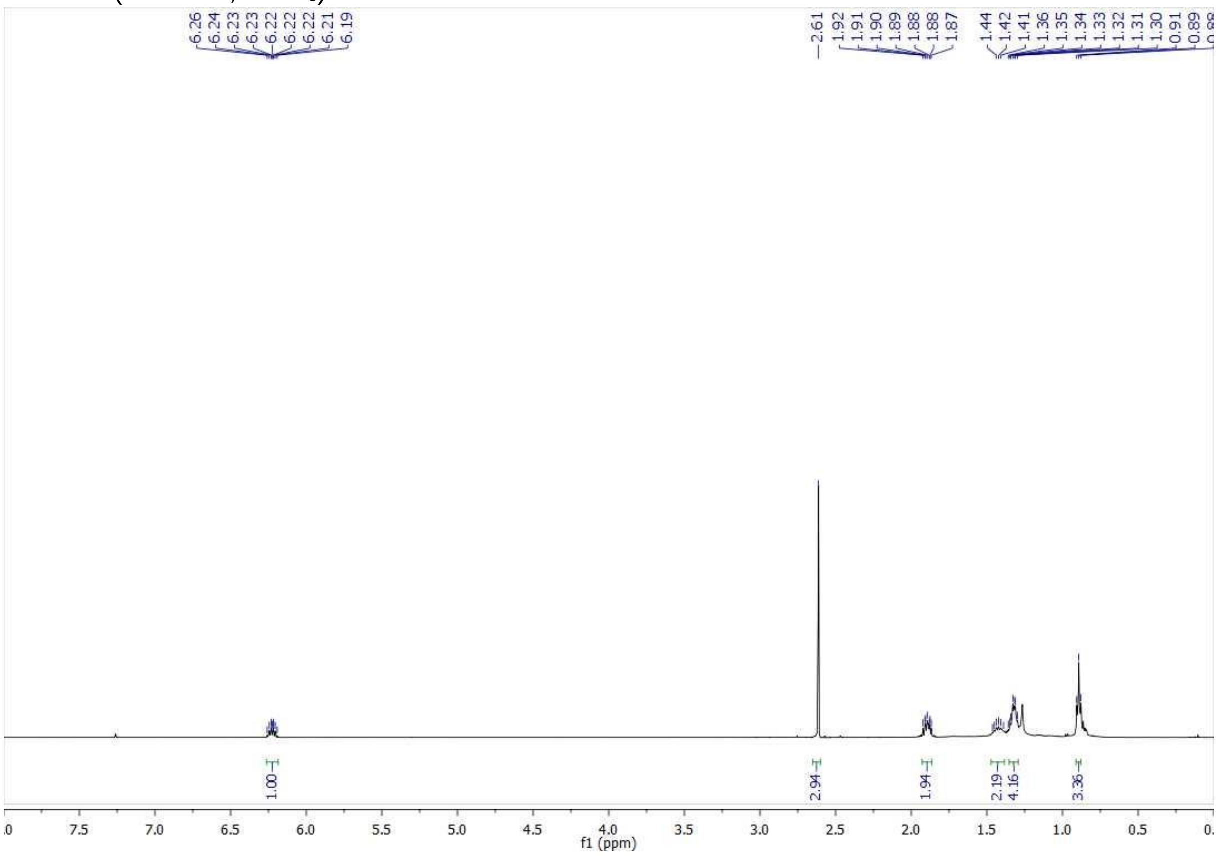

$^{13}C\{^1H\}$ NMR (126 MHz, $CDCl_3$)

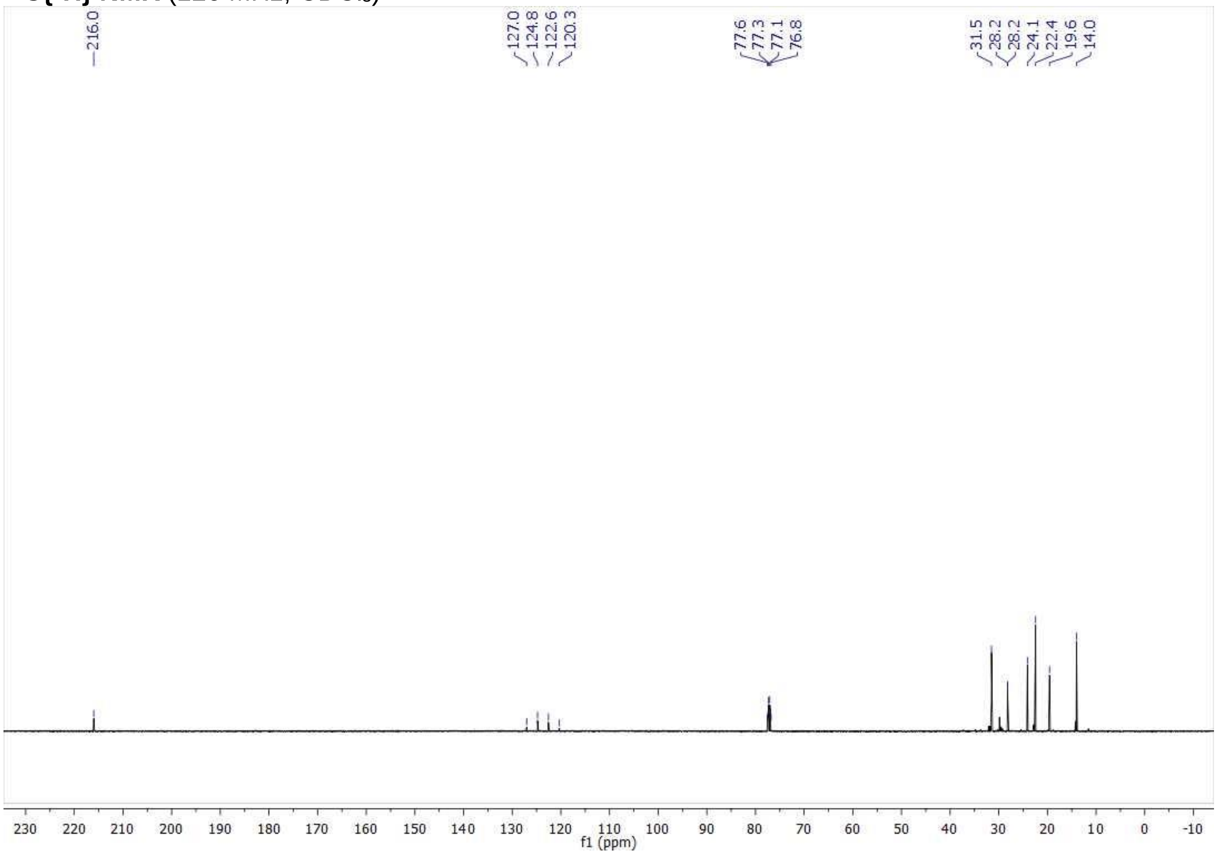

^{19}F NMR (376 MHz, $CDCl_3$)

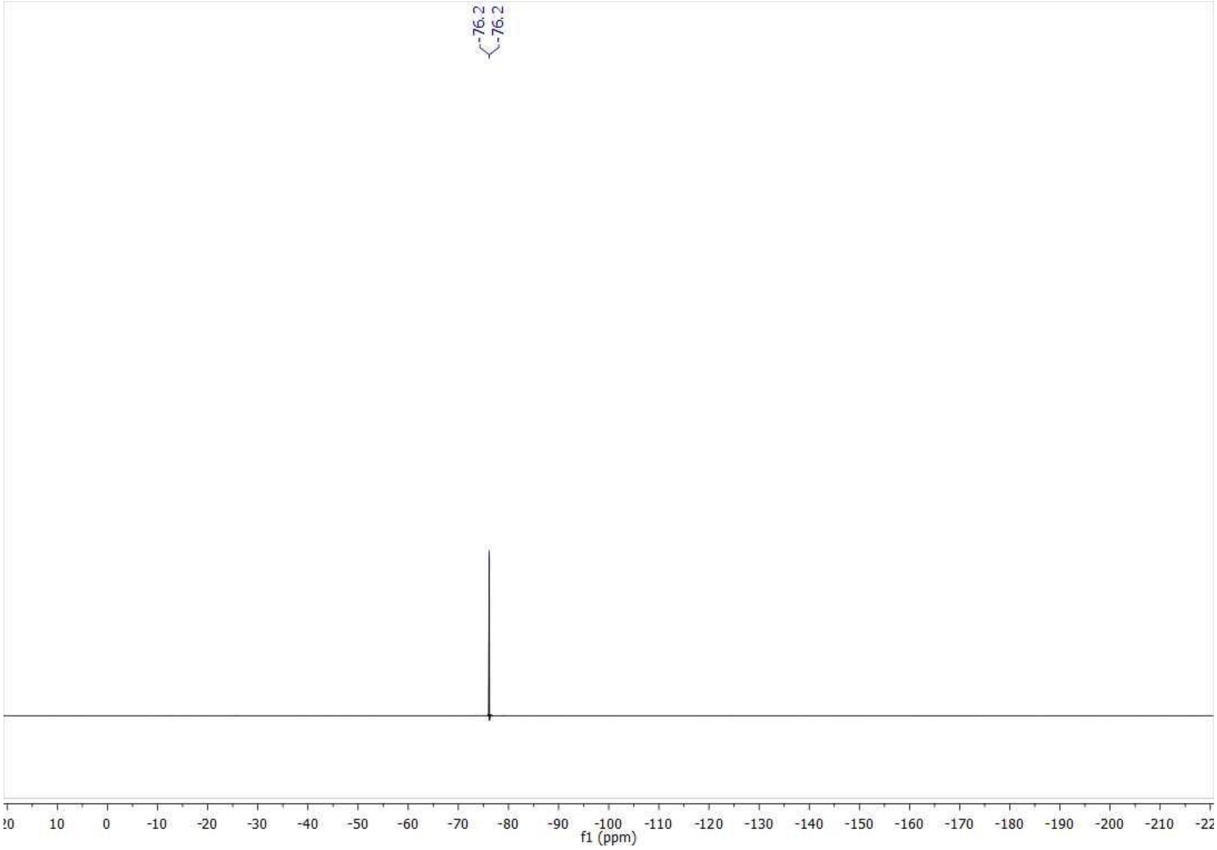

_S_-Methyl _O_-(1,1,1-trifluoro-3-methylhexan-2-yl) carbonodithioate (12’)

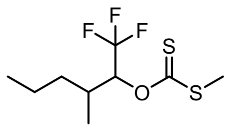

12'

^{1}H NMR (400 MHz, $CDCl_3$)

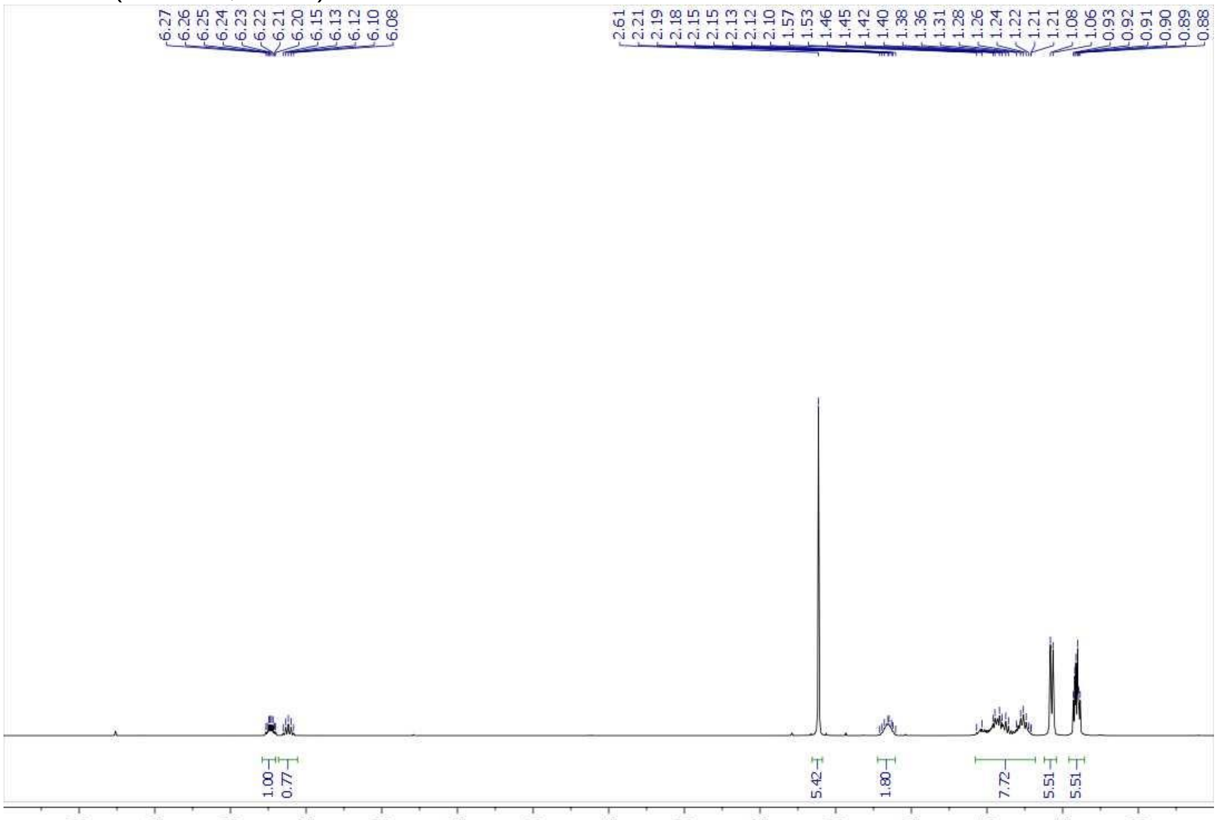

$^{13}C\{^{1}H\}$ **NMR** (101 MHz, $CDCl_3$)

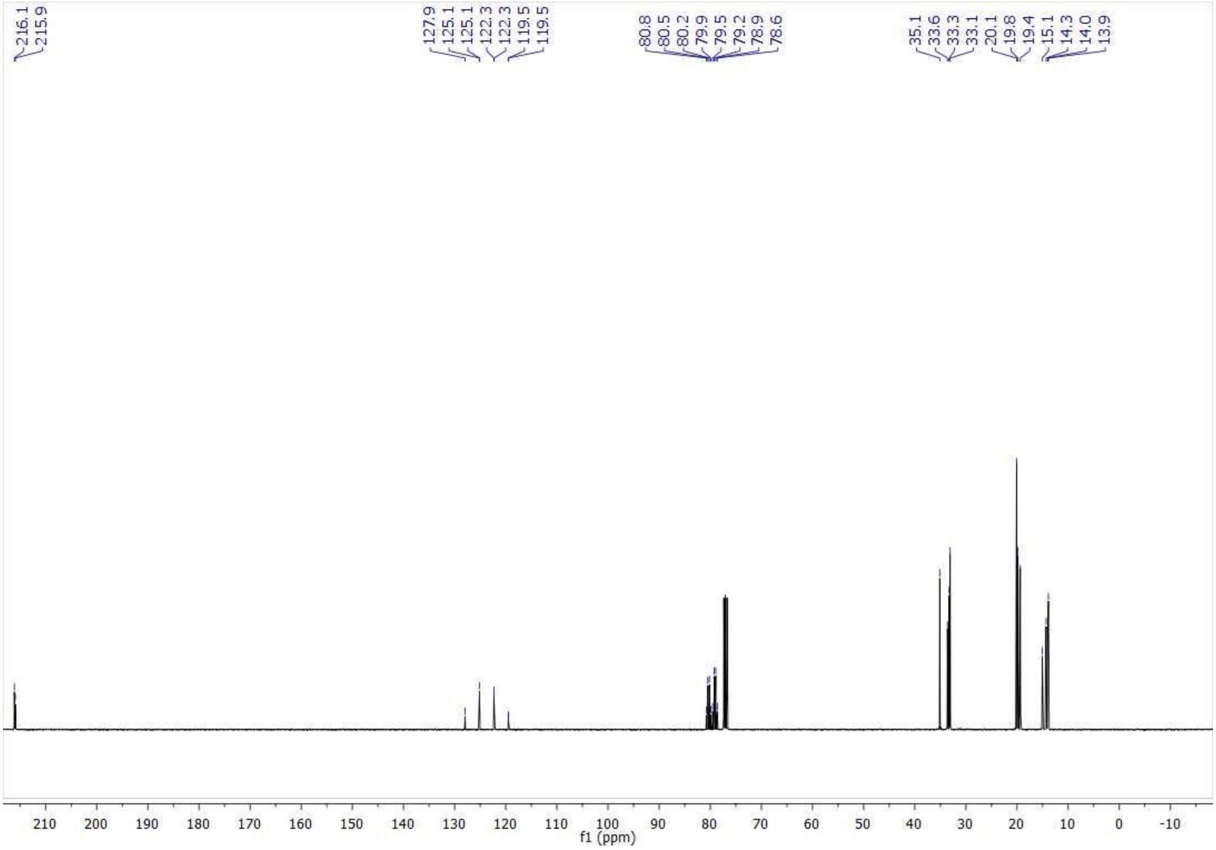

^{19}F **NMR** (282 MHz, $CDCl_3$)

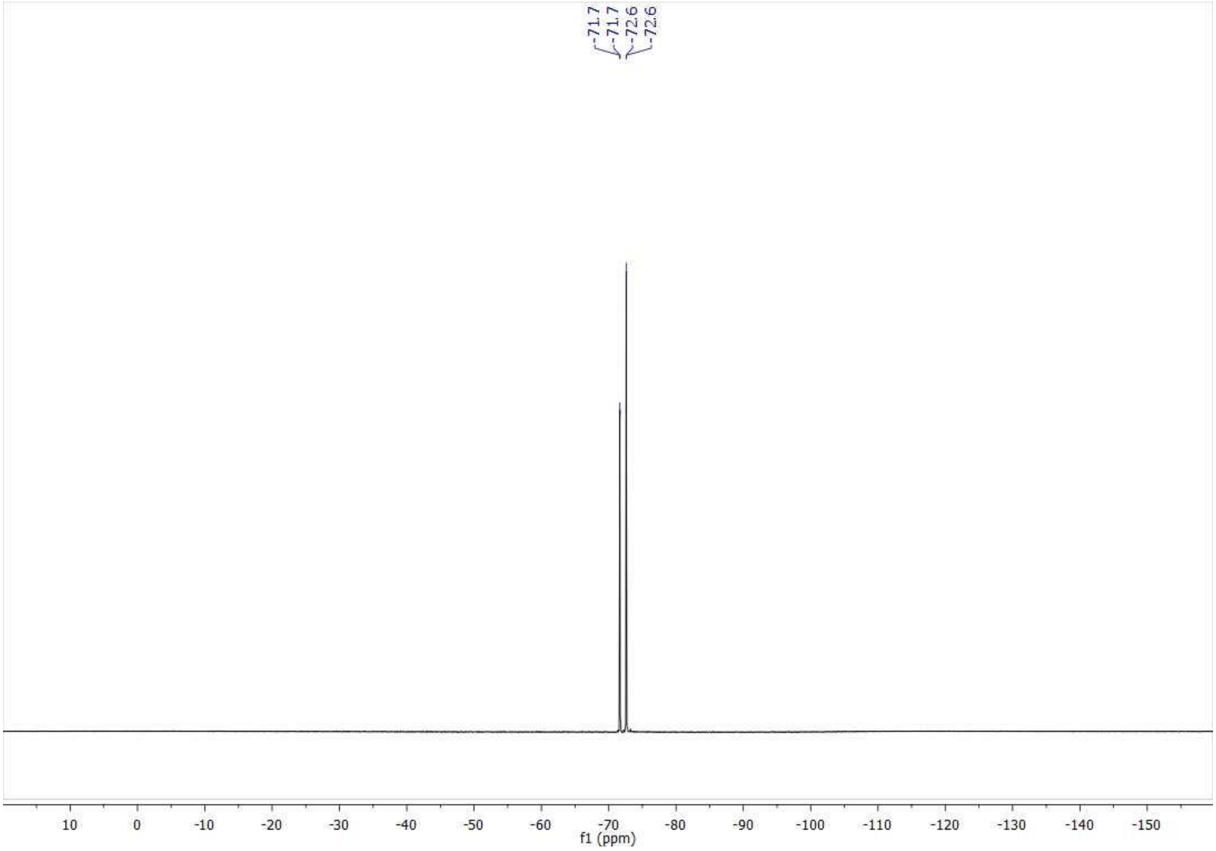

_S_-Methyl _O_-(1,1,1-trifluoro-3-ethylpentan-2-yl) carbonodithioate (13')

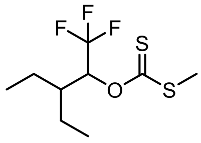

13'

^{1}H NMR (400 MHz, $CDCl_3$)

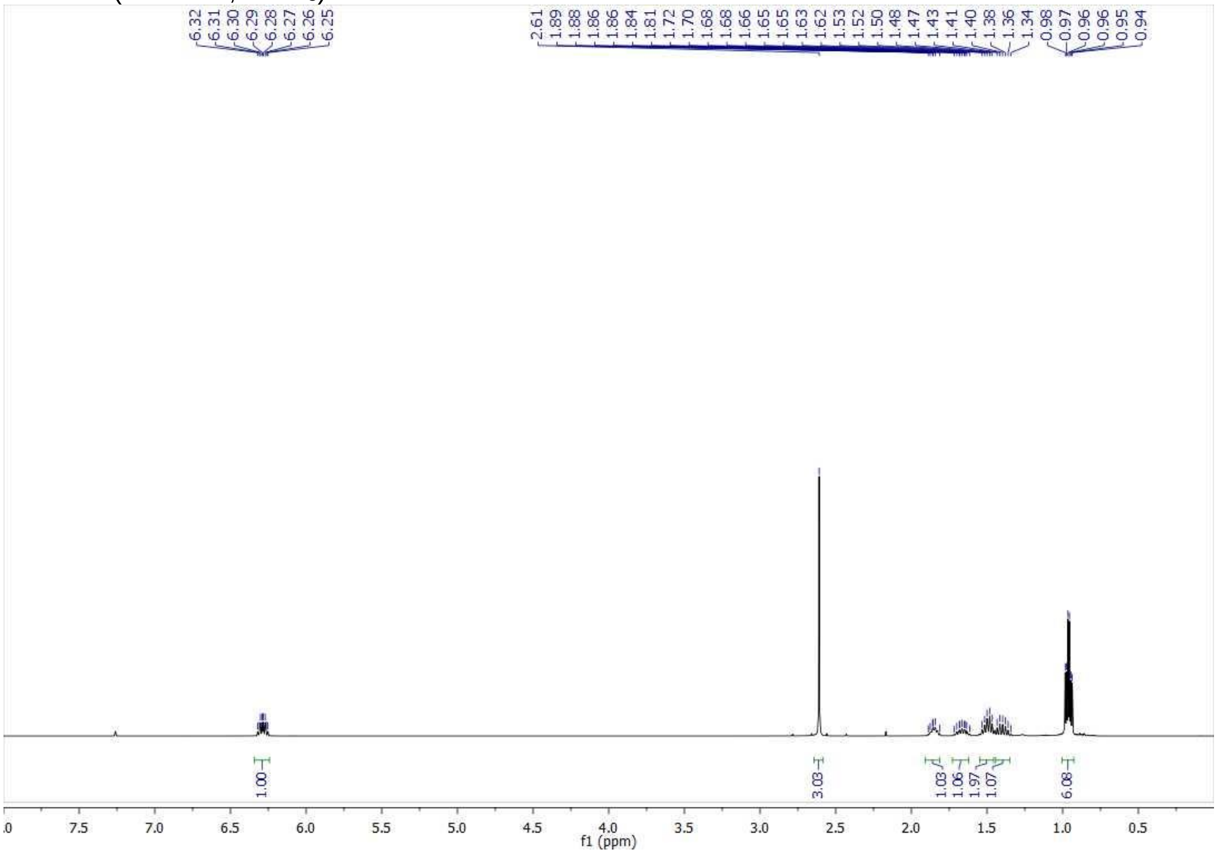

$^{13}C\{^{1}H\}$ NMR (101 MHz, $CDCl_3$)

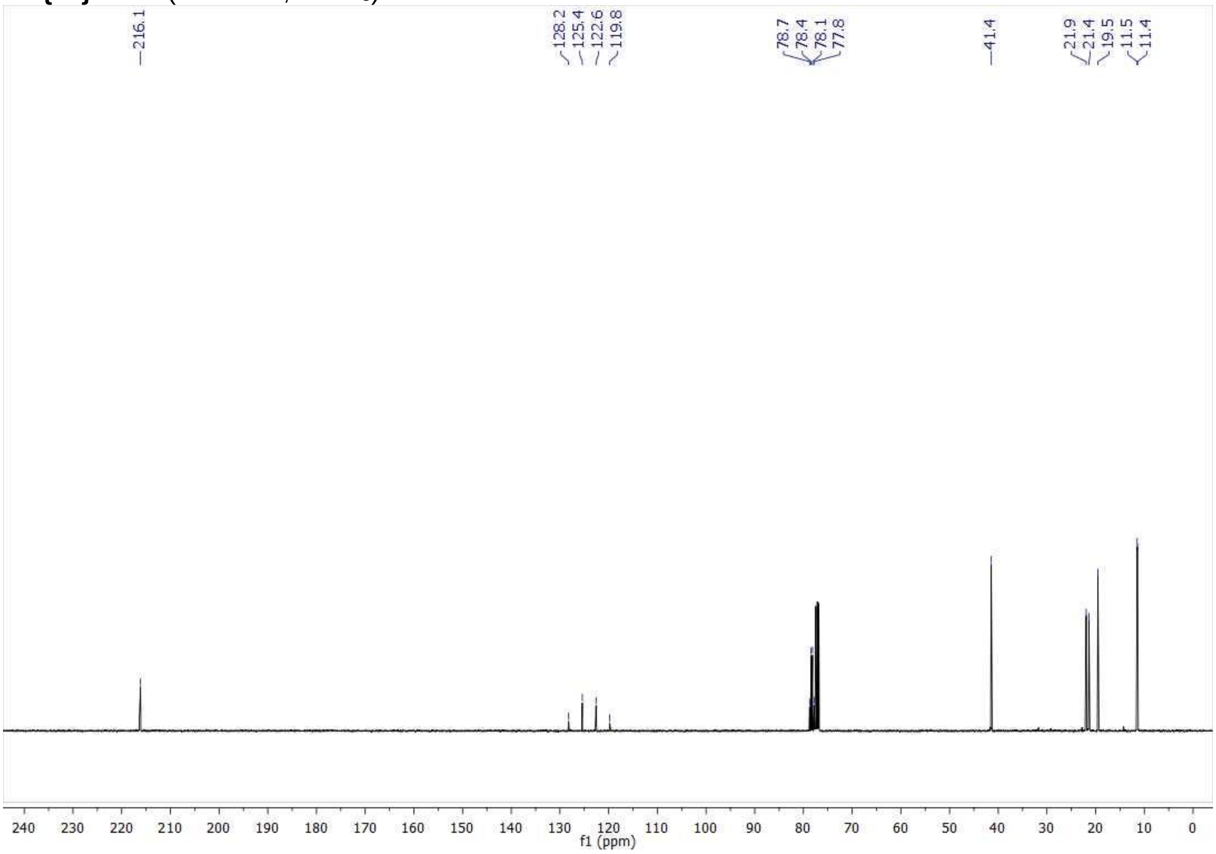

^{19}F NMR (376 MHz, $CDCl_3$)

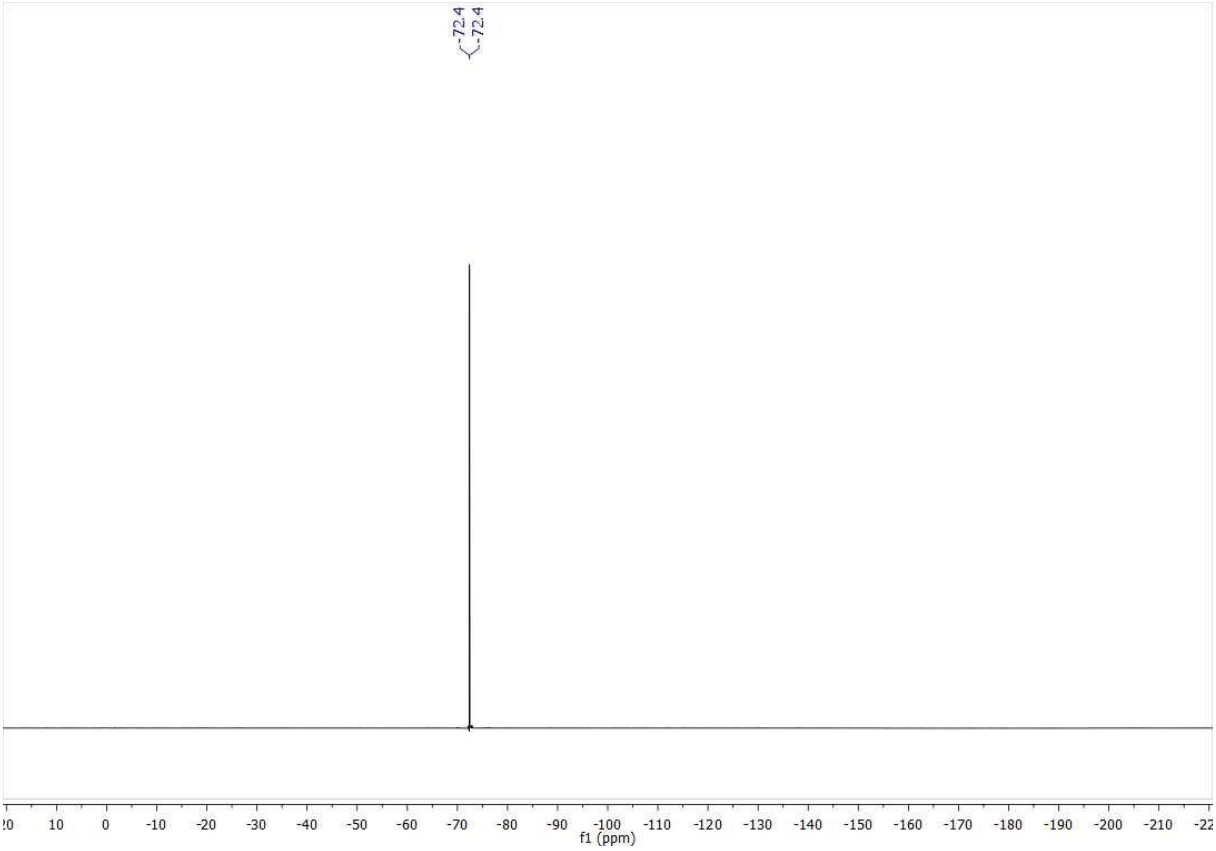

S-Methyl O-(1,1,1-trifluorooctan-2-yl) carbonodithioate (14')

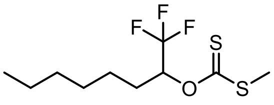

14'

^{1}H NMR (500 MHz, $CDCl_3$)

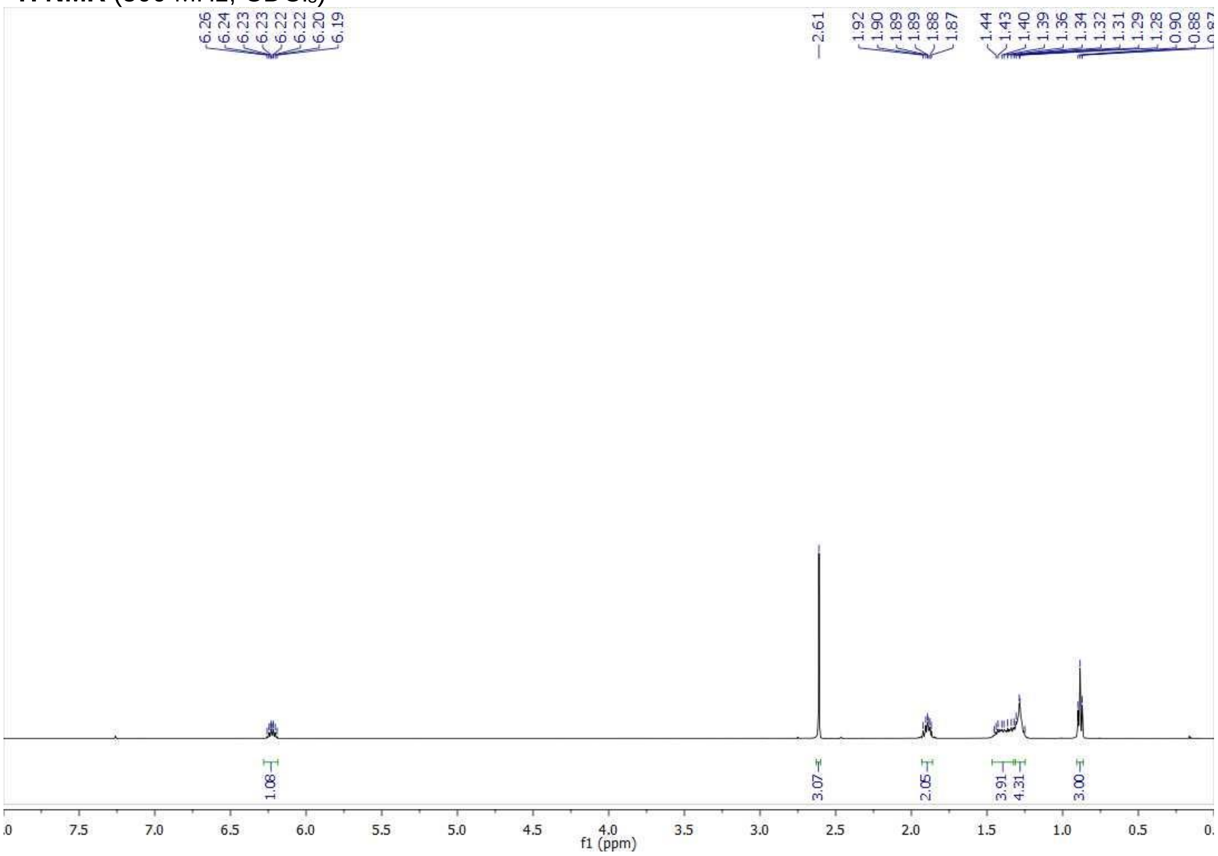

^{13}C{^{1}H} NMR (126 MHz, $CDCl_3$)

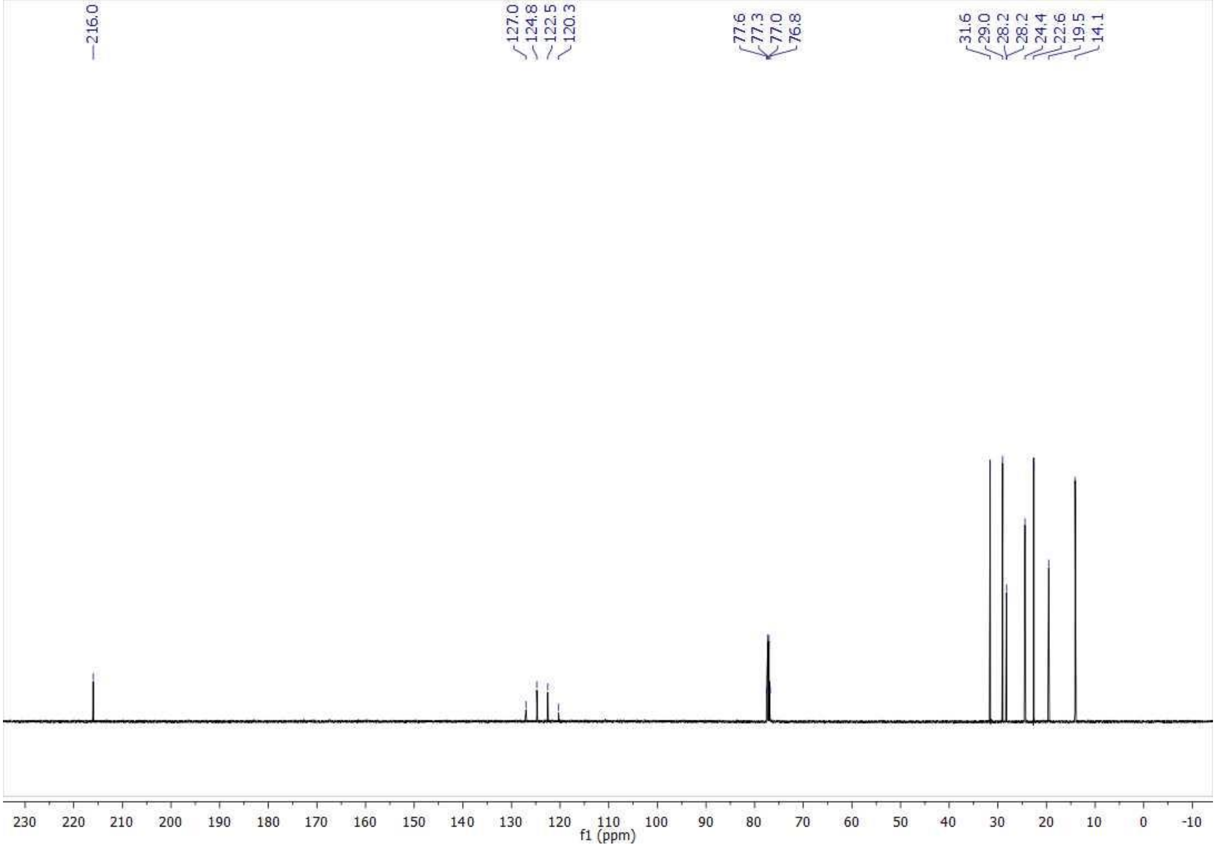

^{19}F NMR (376 MHz, $CDCl_3$)

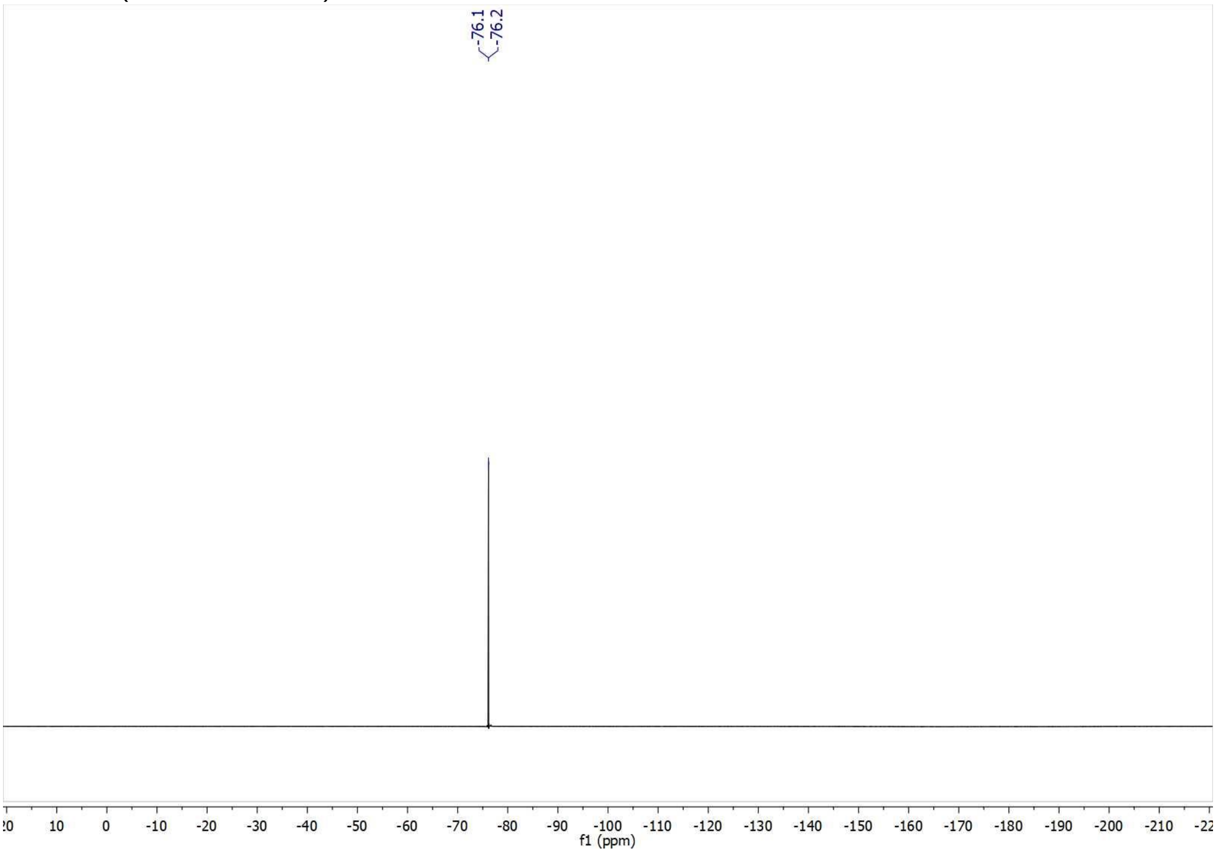

***S*-Methyl *O*-(1,1,1-trifluoro-3-methylheptan-2-yl) carbonodithioate (15')**

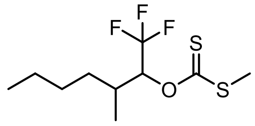

15'

^{1}H NMR (400 MHz, $CDCl_3$)

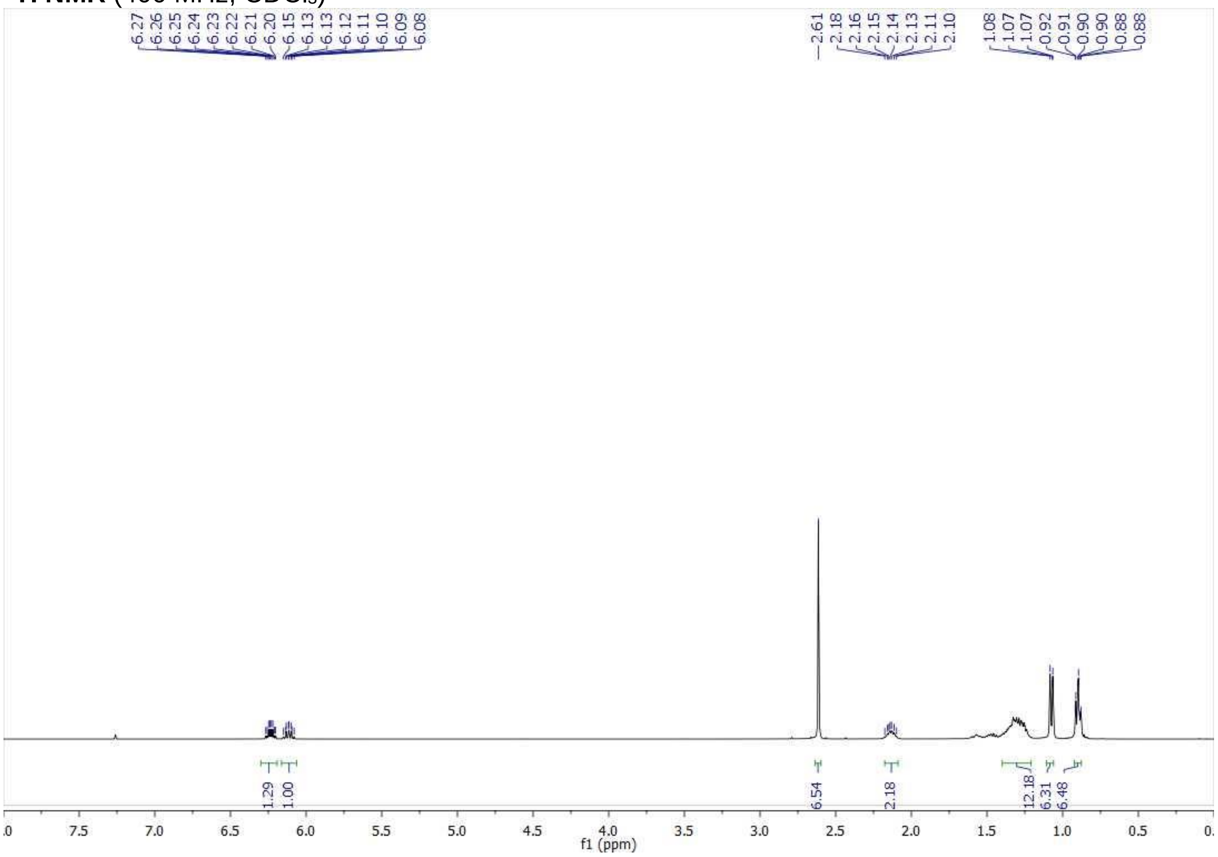

$^{13}C\{^1H\}$ NMR (101 MHz, $CDCl_3$)

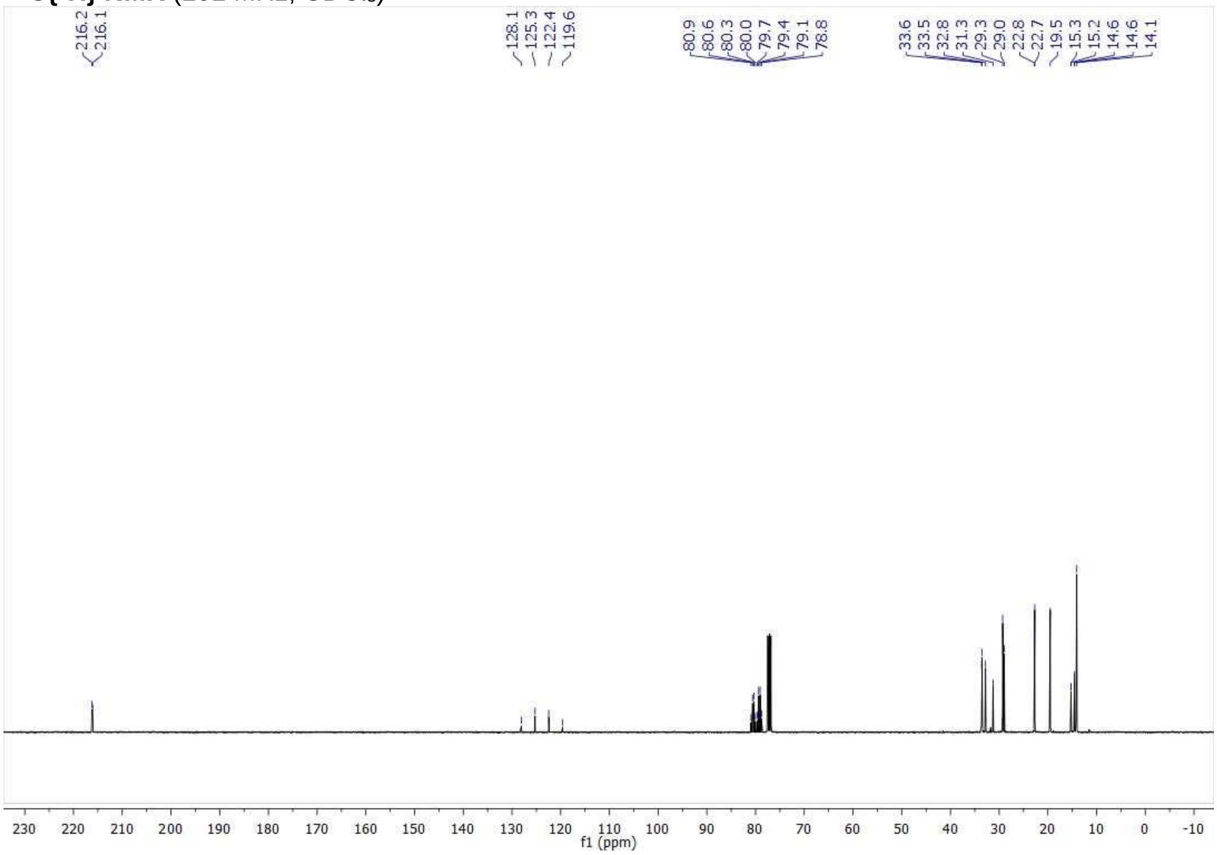

^{19}F NMR (376 MHz, $CDCl_3$)

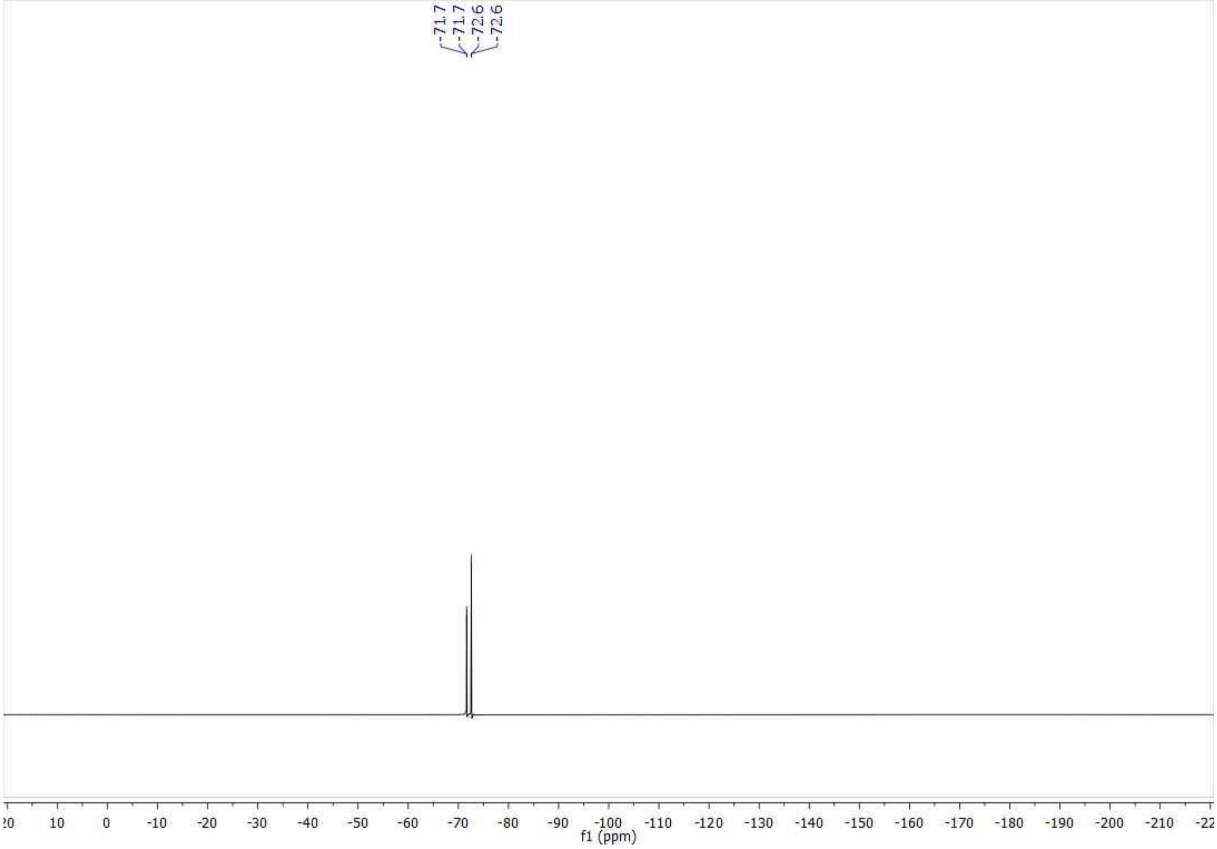

***S*-Methyl *O*-(1,1,1-trifluoro-3-ethylhexan-2-yl) carbonodithioate (16')**

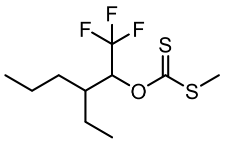

16'

^{1}H NMR (400 MHz, $CDCl_3$)

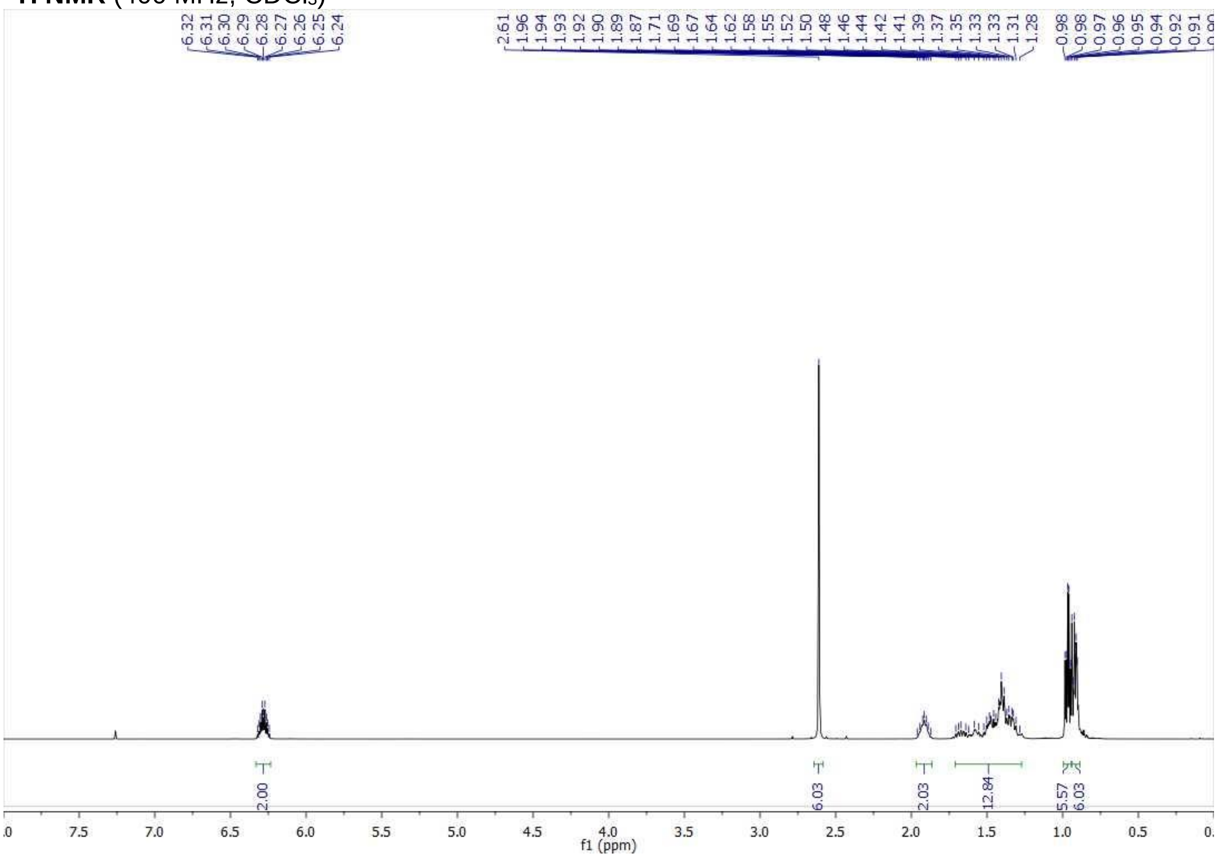

^{13}C{^{1}H} NMR (101 MHz, $CDCl_3$)

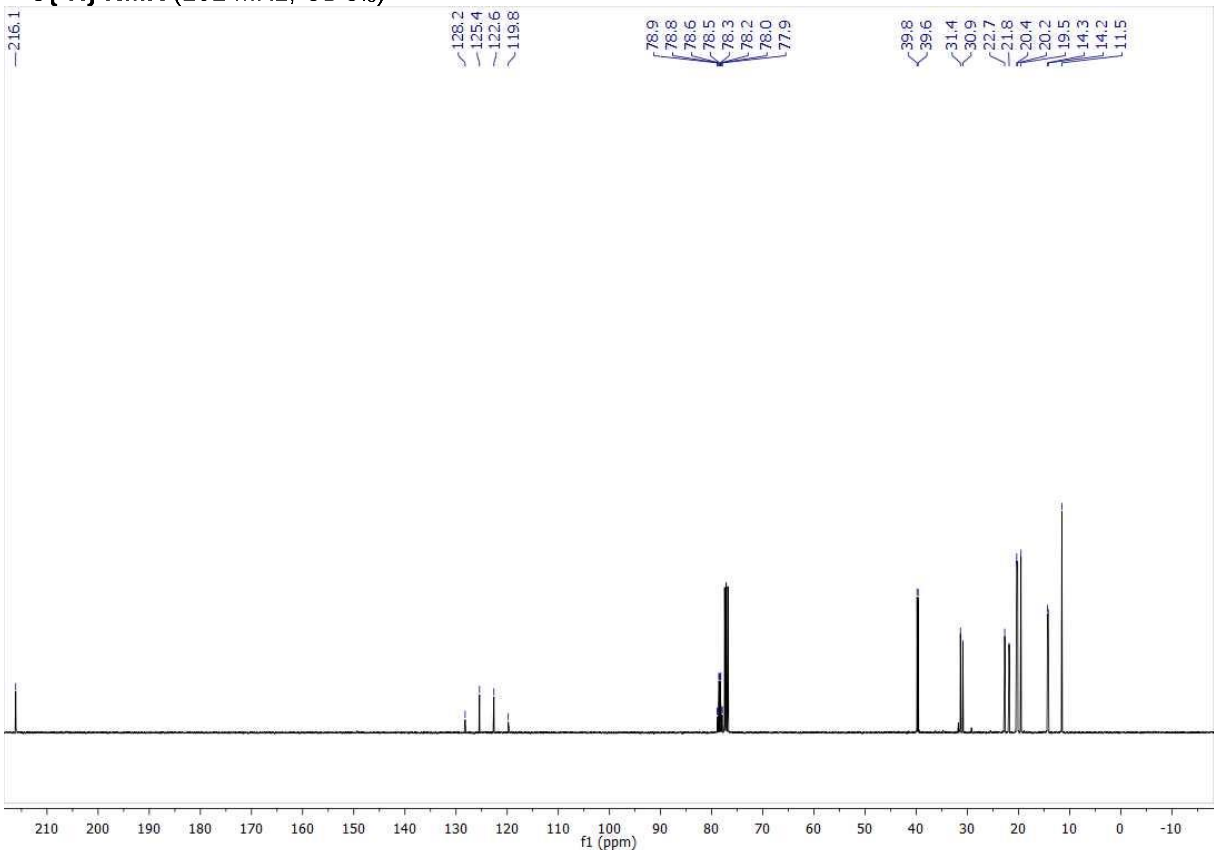

^{19}F NMR (376 MHz, $CDCl_3$)

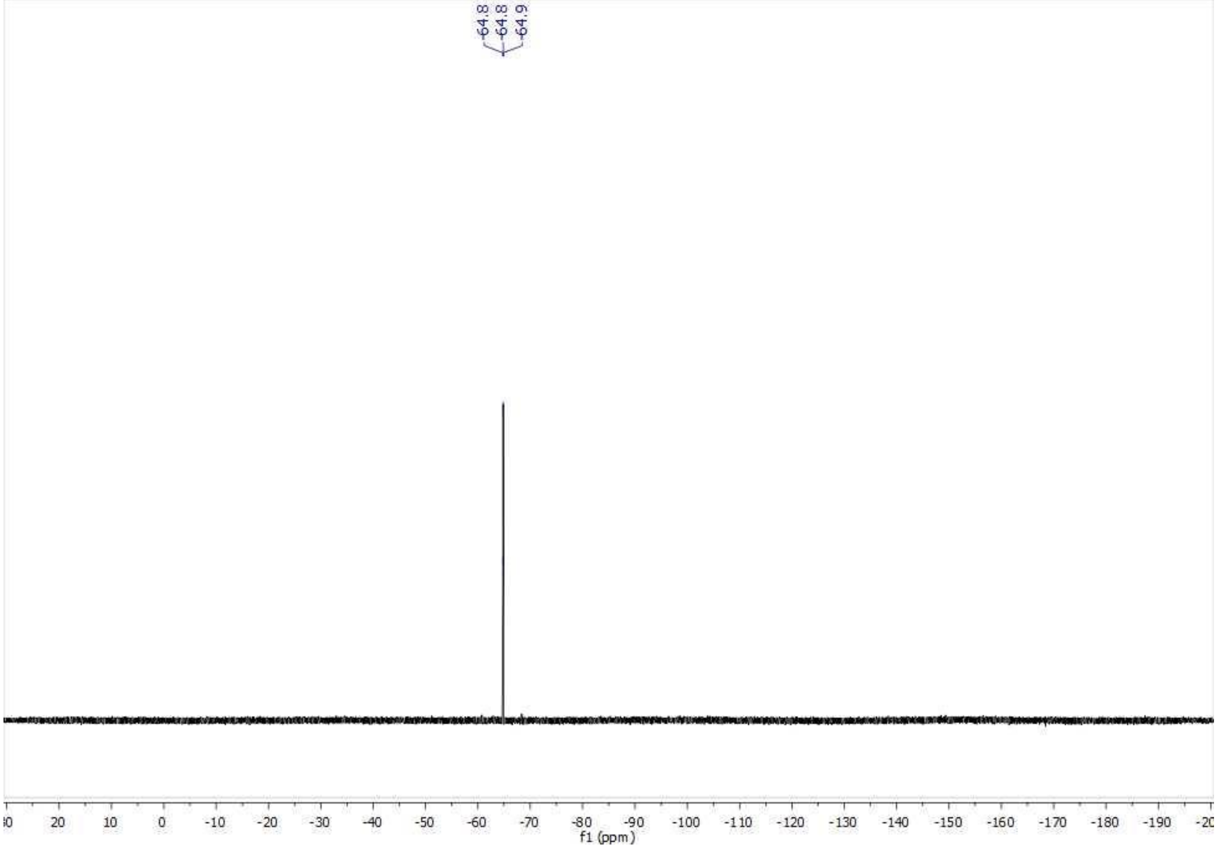

S-Methyl *O*-(1-cyclohexyl-2,2,2-trifluoroethyl) carbonodithioate (17’)

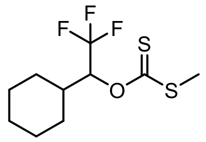

17'

^{1}H NMR (400 MHz, $CDCl_3$)

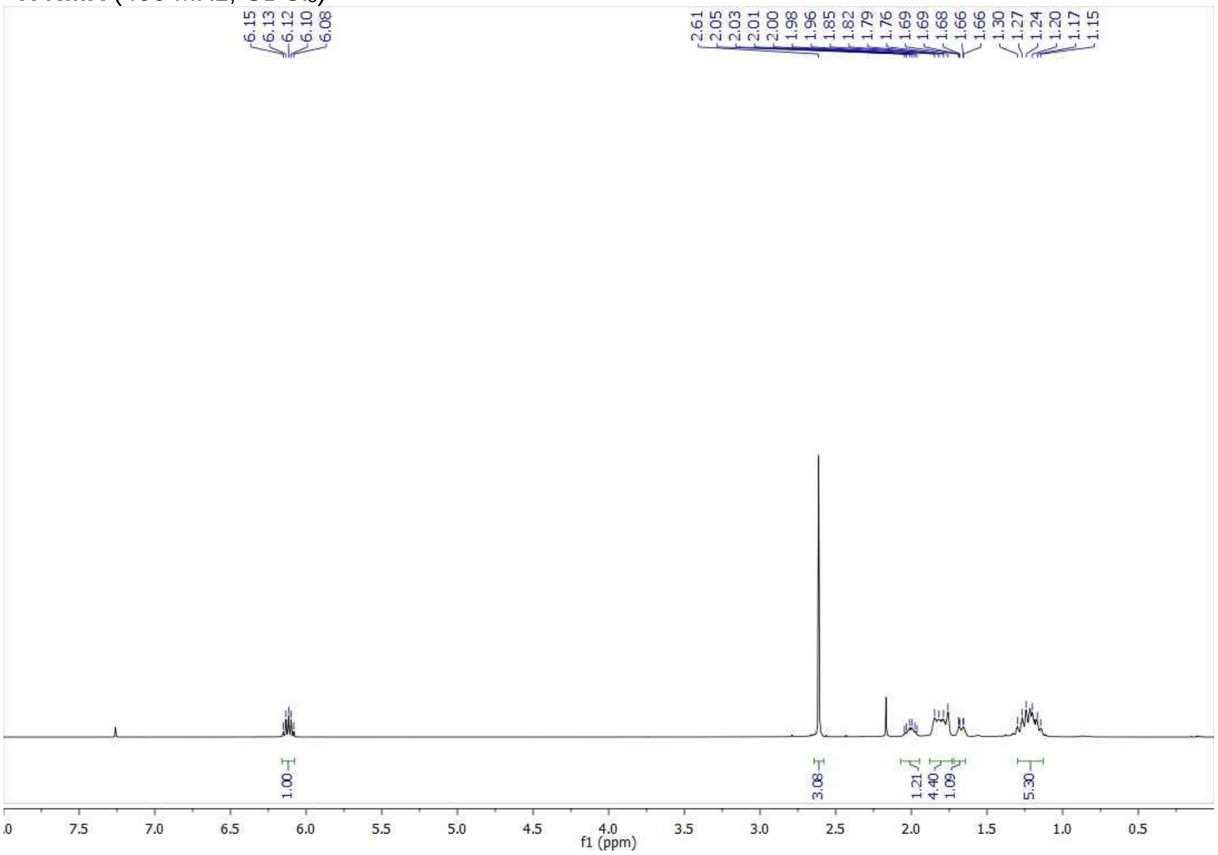

$^{13}C\{^1H\}$ NMR (101 MHz, $CDCl_3$)

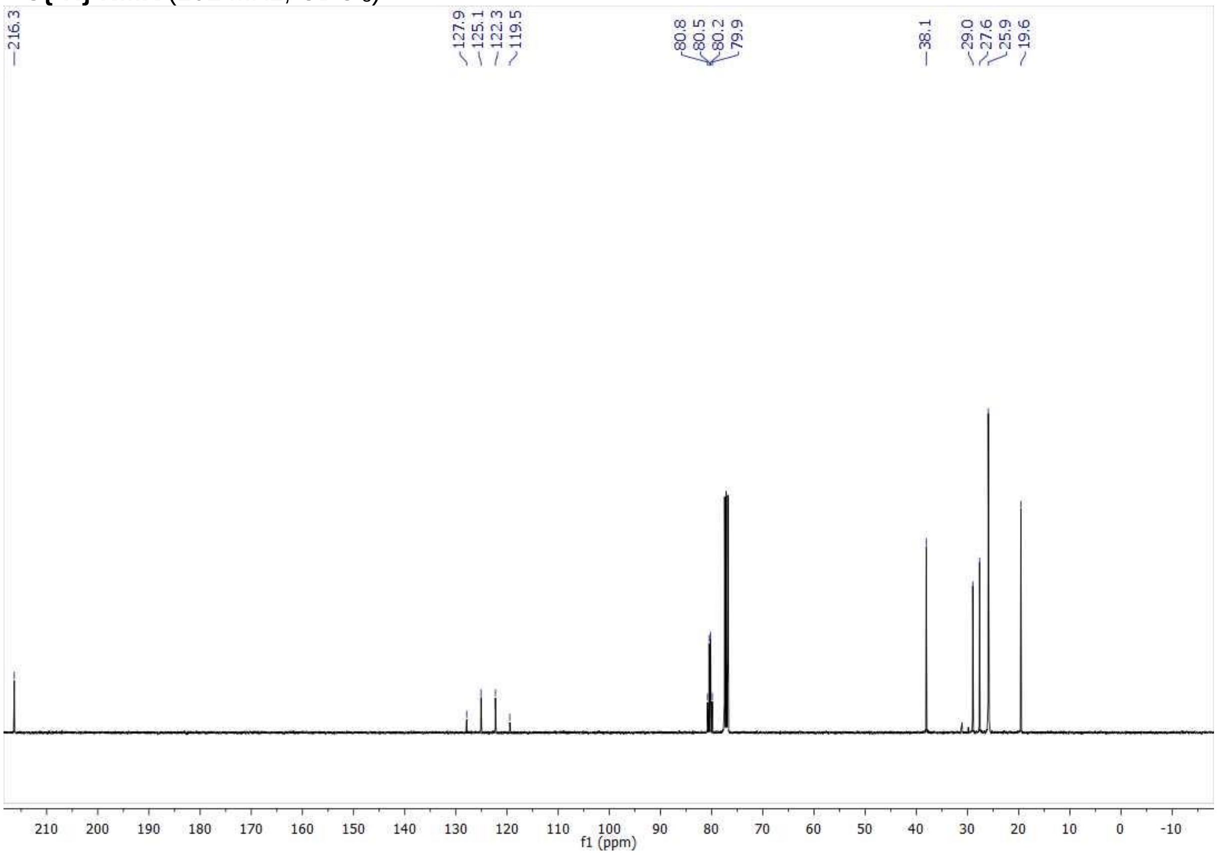

^{19}F NMR (376 MHz, $CDCl_3$)

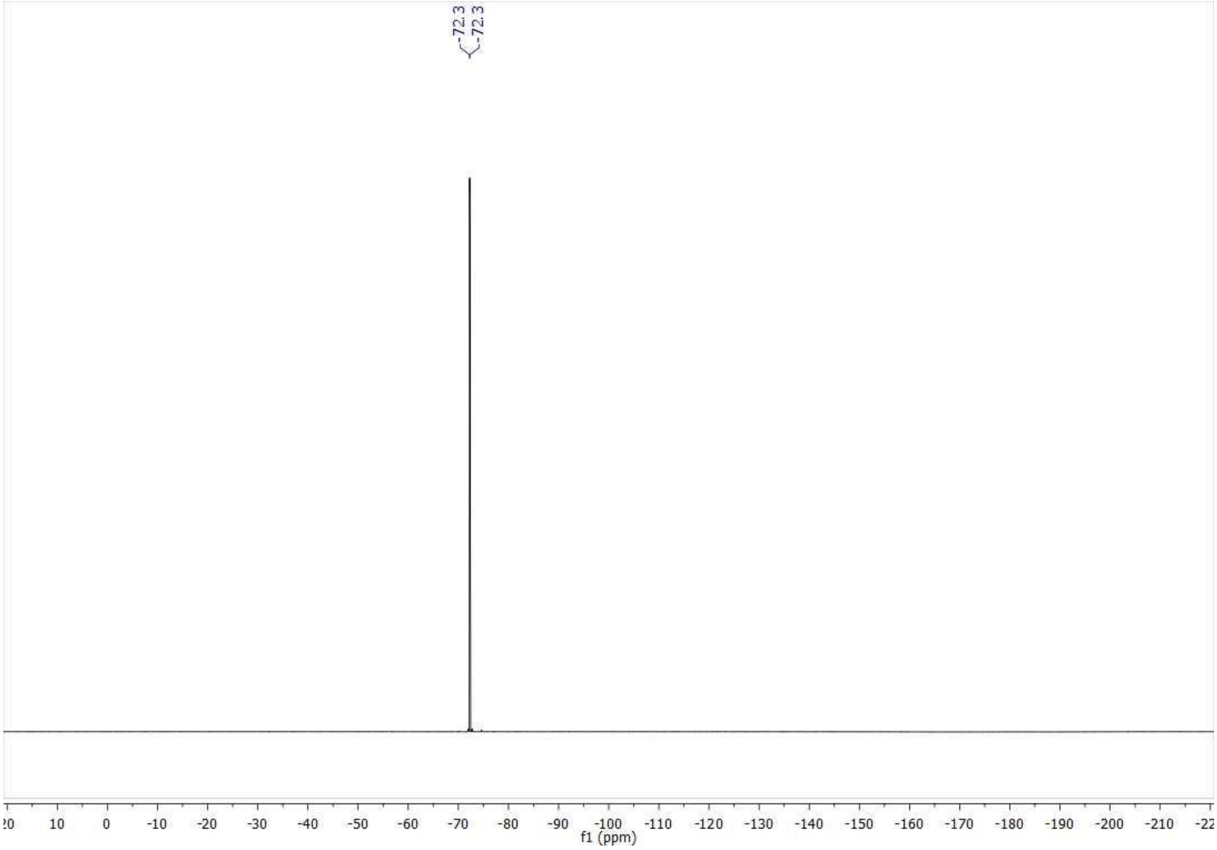

1,1,1-trifluorobutane (4)

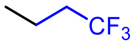

4

^{1}H NMR (400 MHz, CD_2Cl_2)

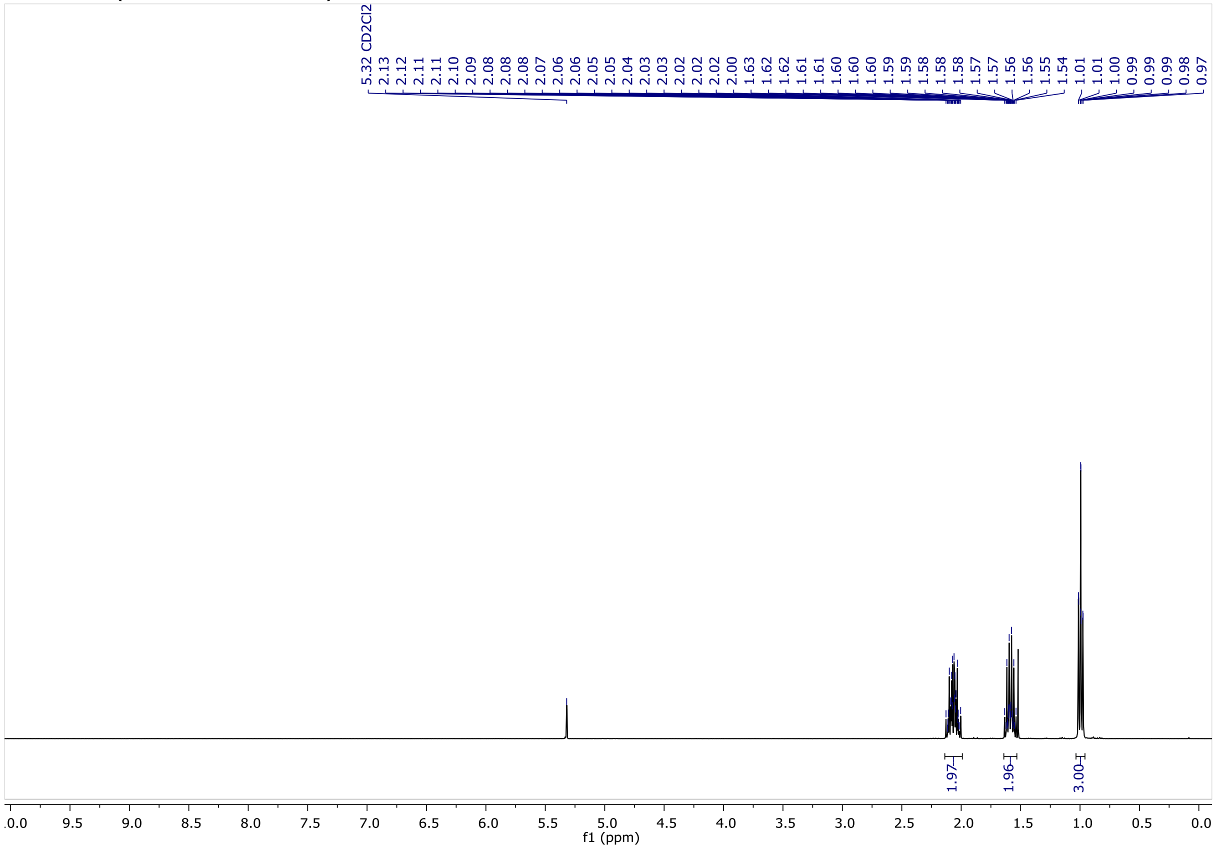

$^{13}C\{^{1}H\}$ NMR (101 MHz, CD_2Cl_2)

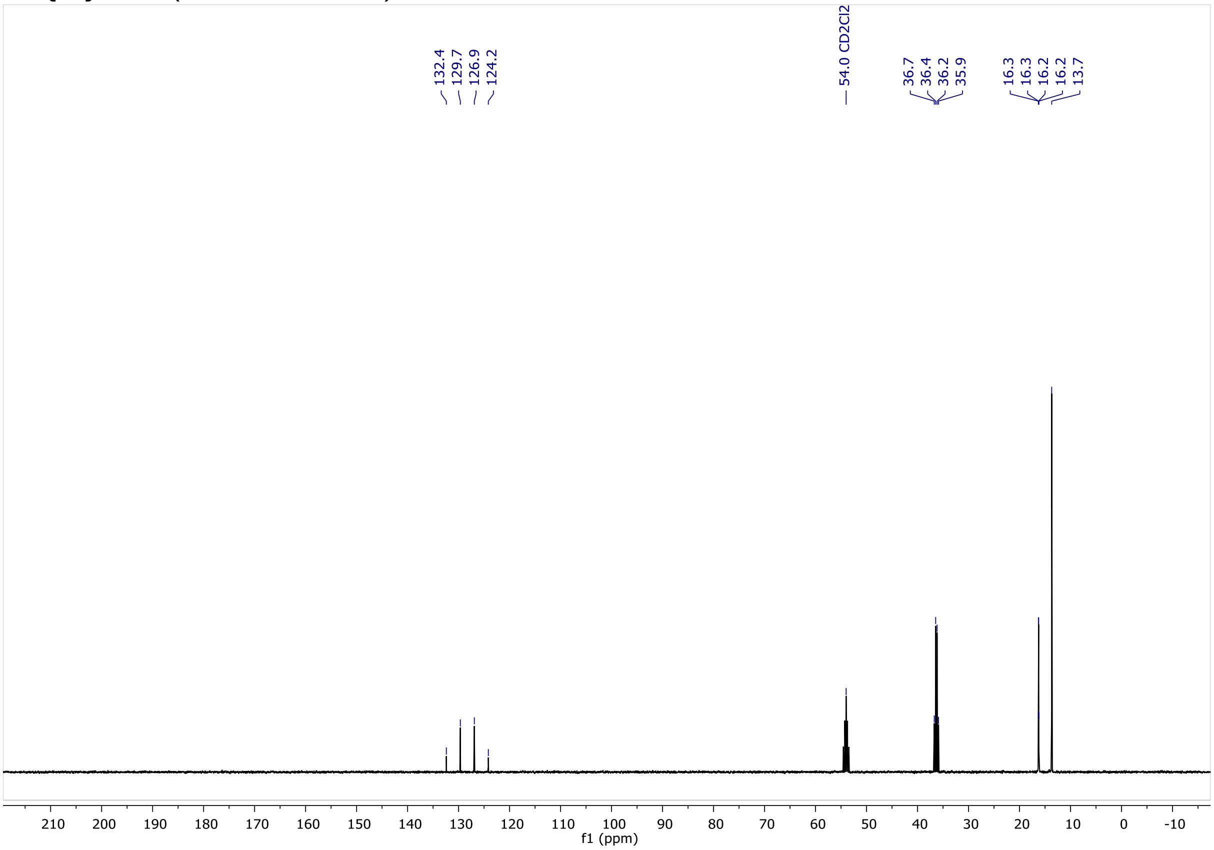

^{19}F NMR (376 MHz, CD_2Cl_2)

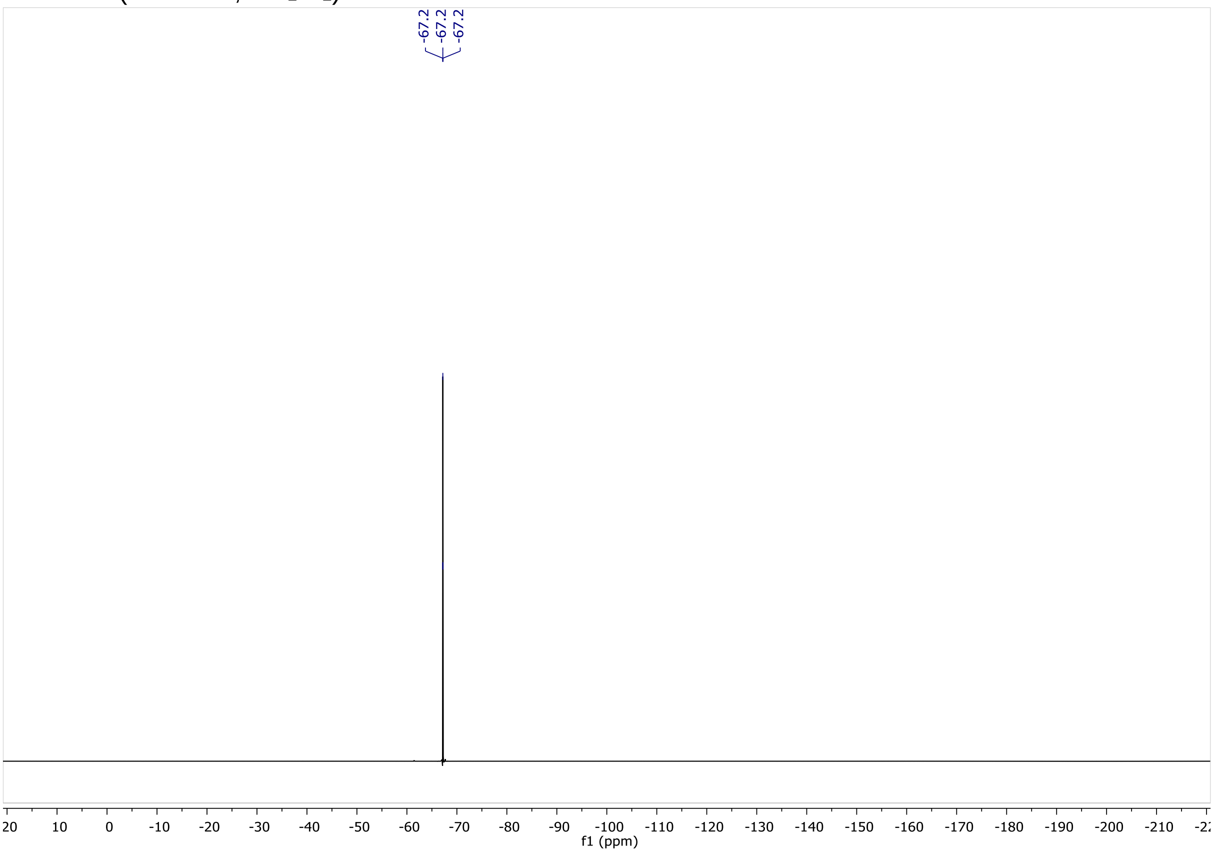

1,1,1-trifluoropentane (5)

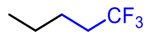

5

¹H NMR (400 MHz, $CDCl_3$)

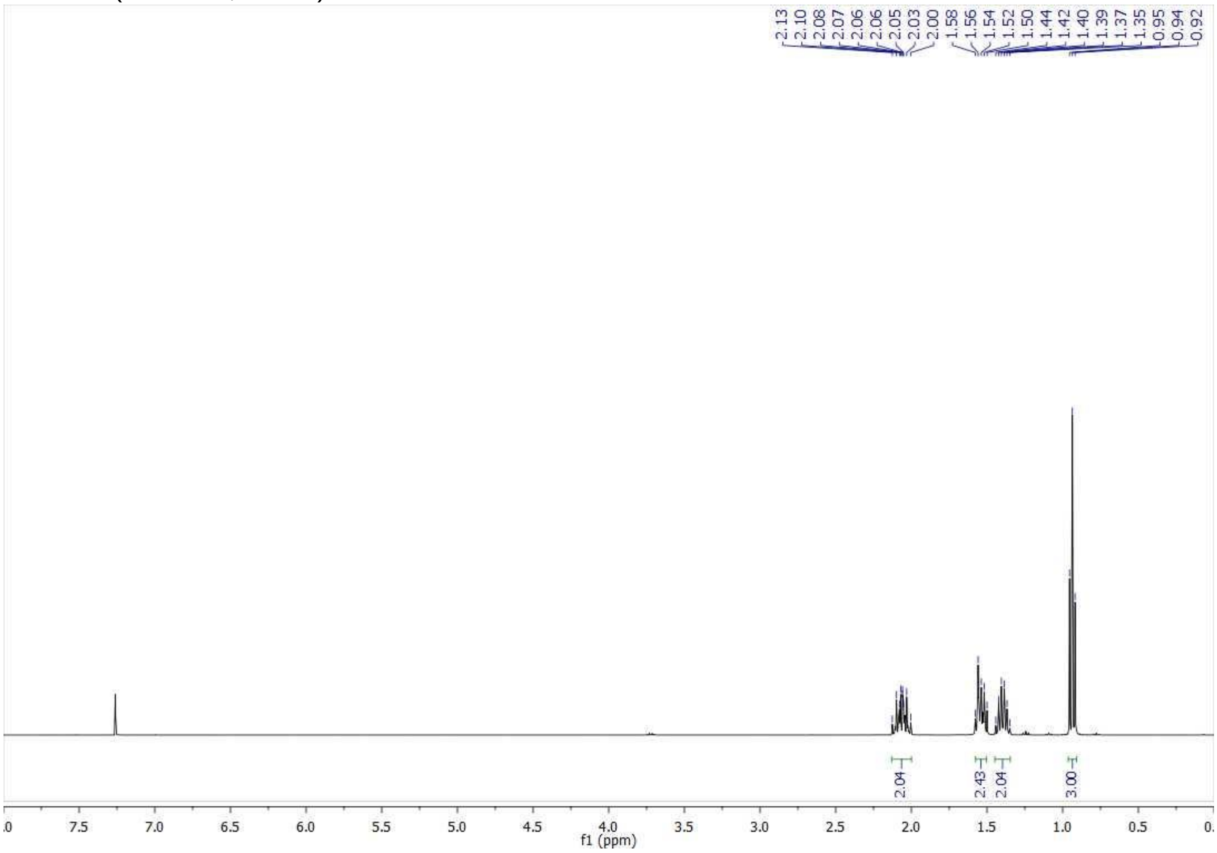

^{13}C{^{1}H} NMR (101 MHz, $CDCl_3$)

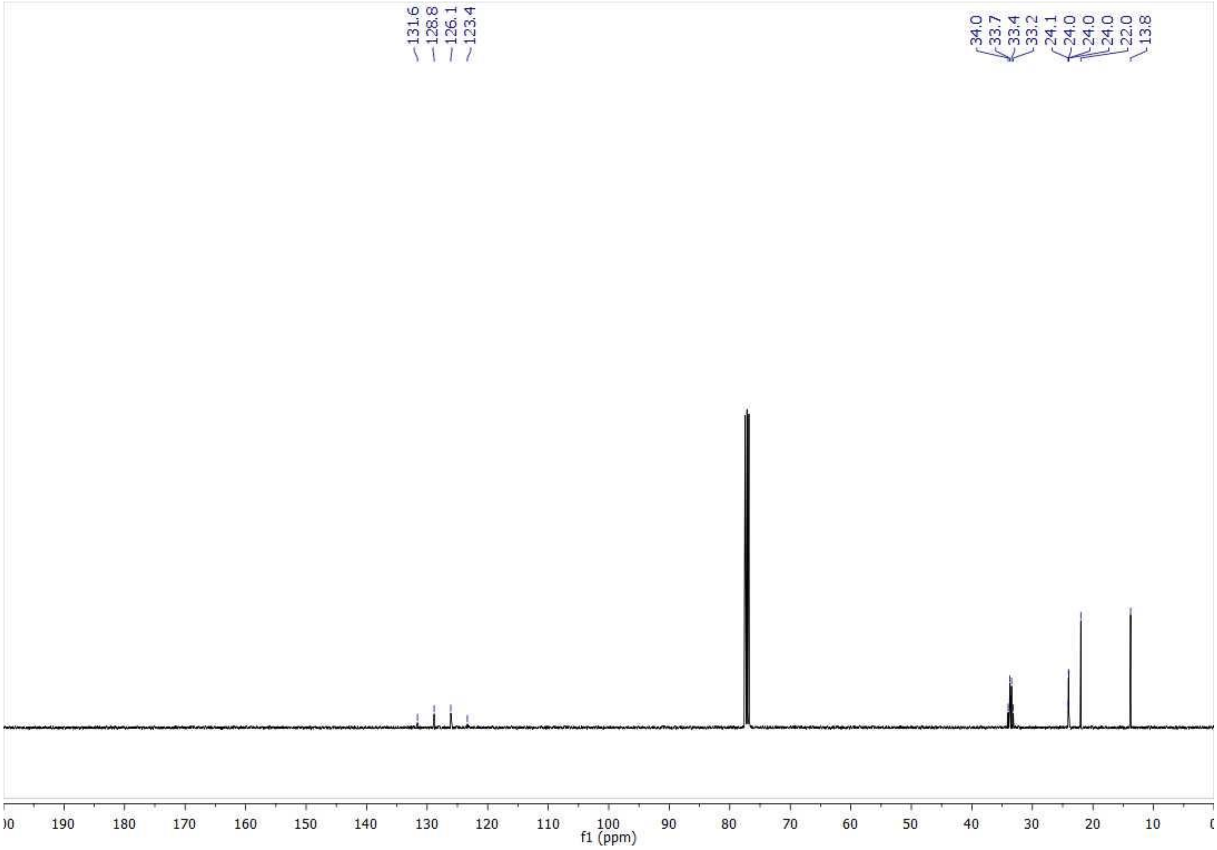

^{19}F NMR (376 MHz, $CDCl_3$)

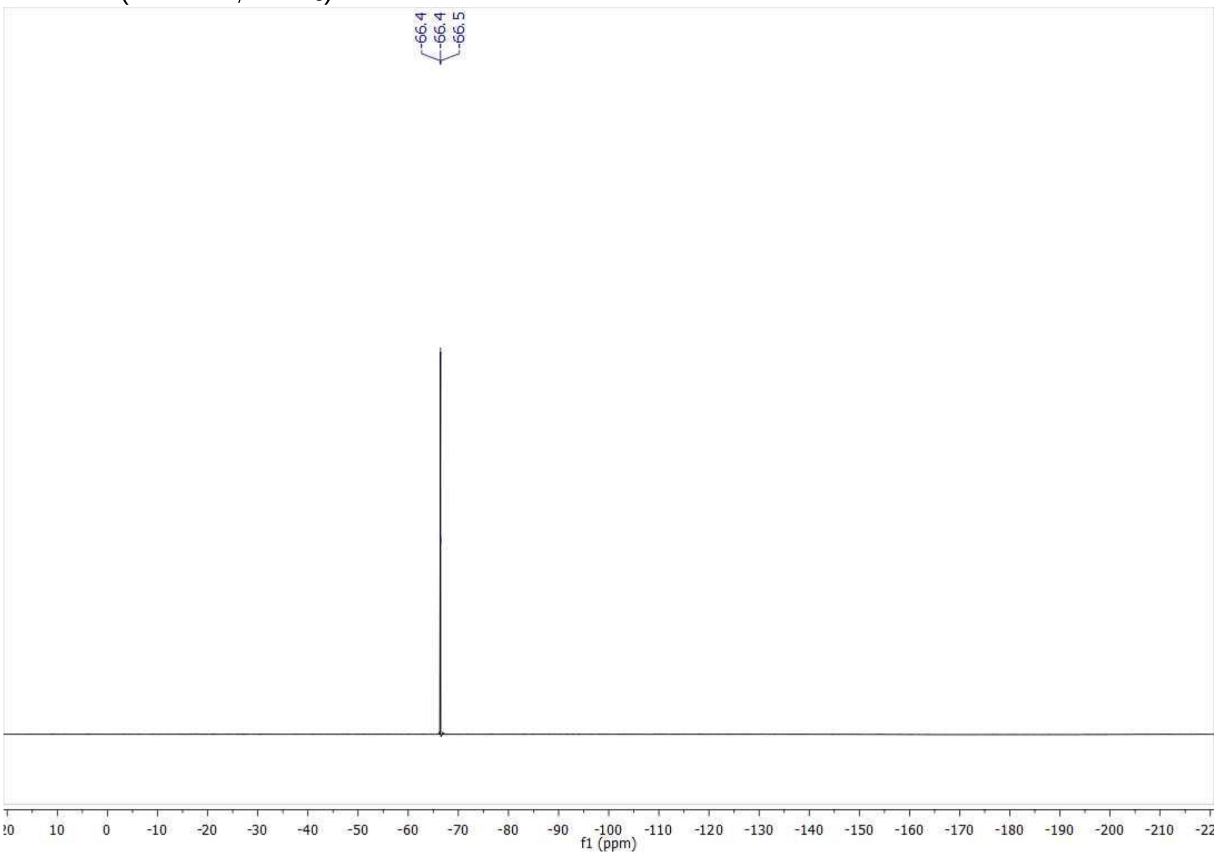

1,1,1-trifluoro-3-methylbutane (6)

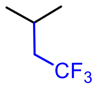

6

^{1}H NMR (400 MHz, $CDCl_3$)

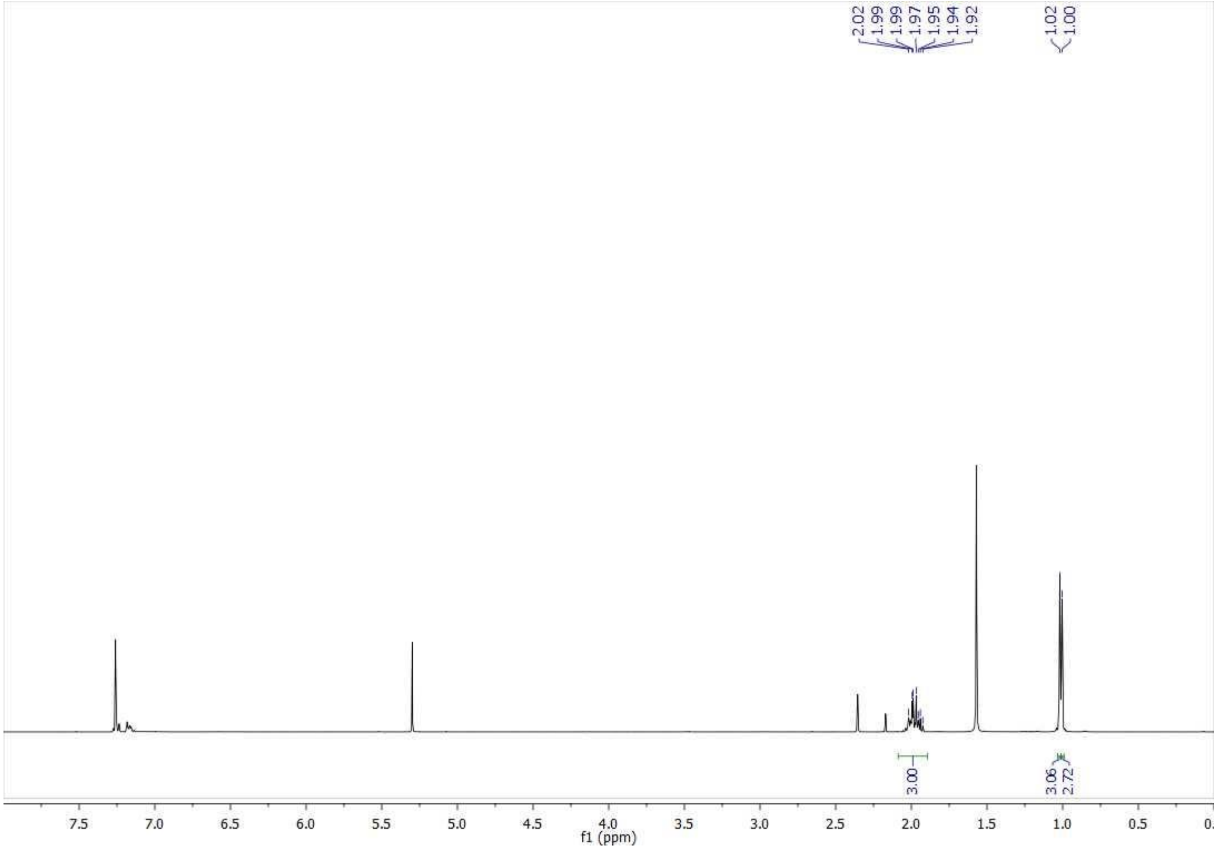

$^{13}C\{^1H\}$ NMR (101 MHz, $CDCl_3$)

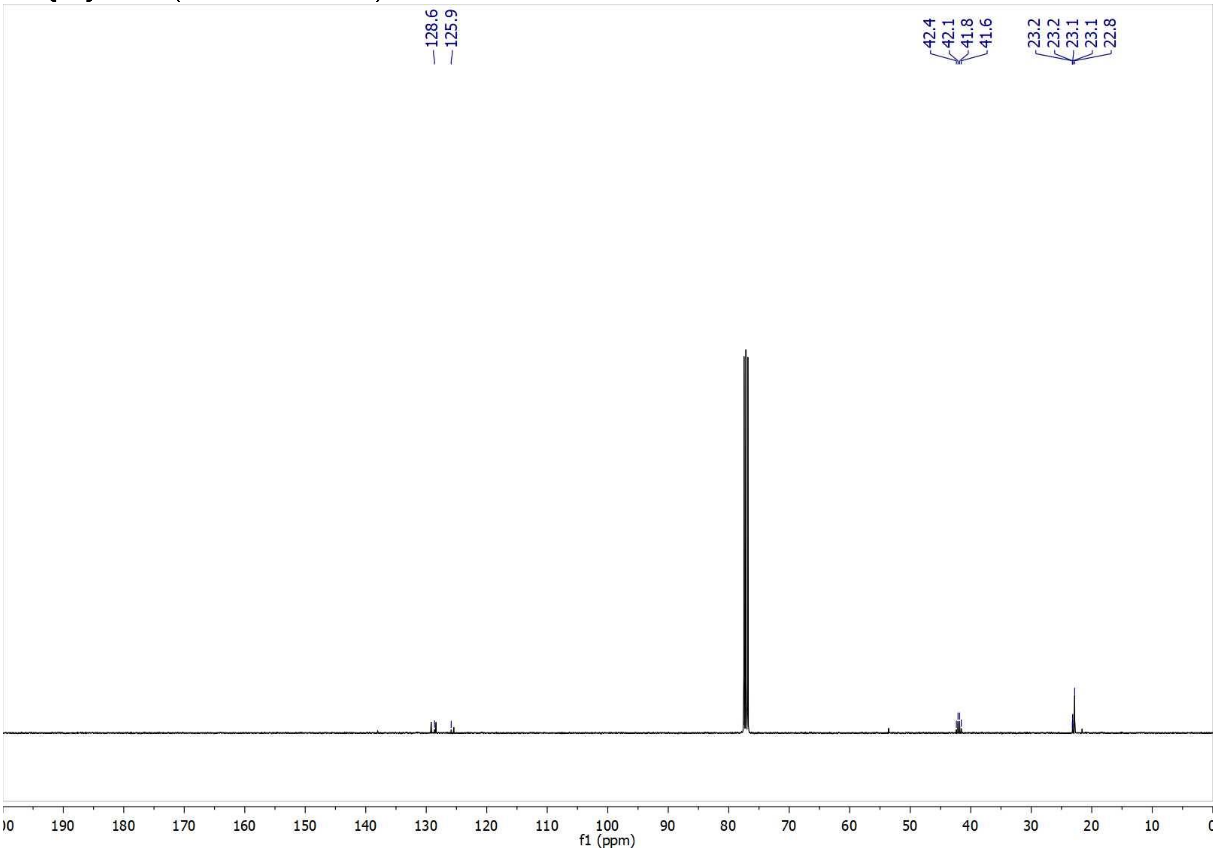

^{19}F NMR (376 MHz, $CDCl_3$)

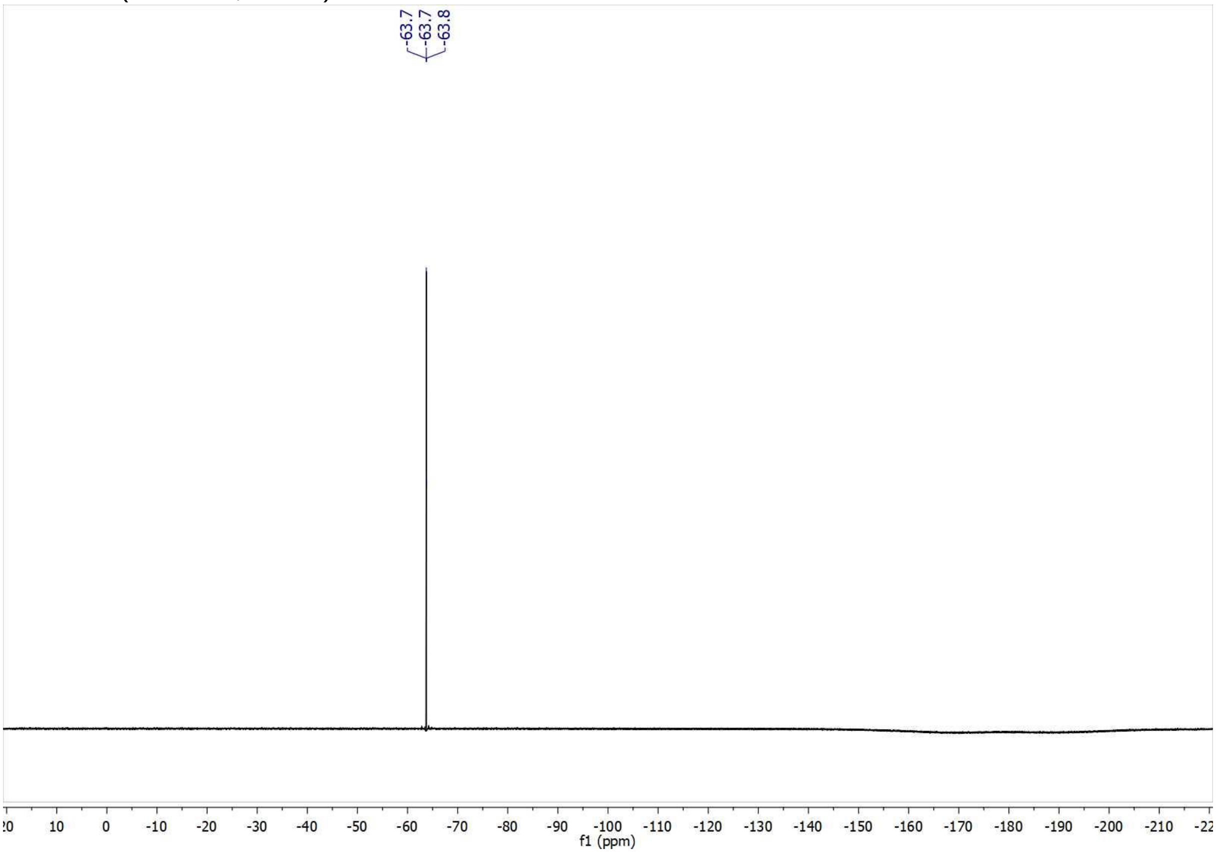

1,1,1-trifluorohexane (7)

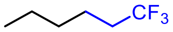

7

^{1}H NMR (400 MHz, $CDCl_3$)

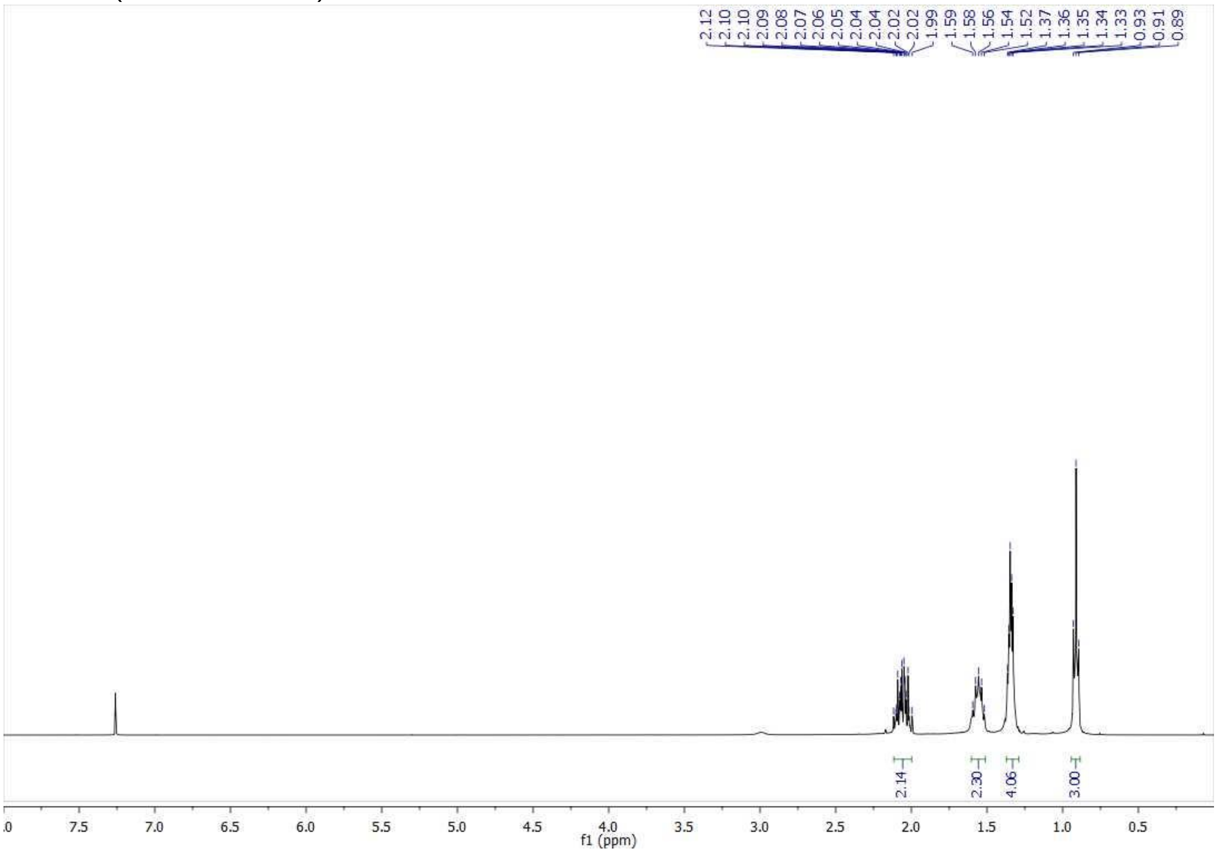

$^{13}C\{^1H\}$ NMR (101 MHz, $CDCl_3$)

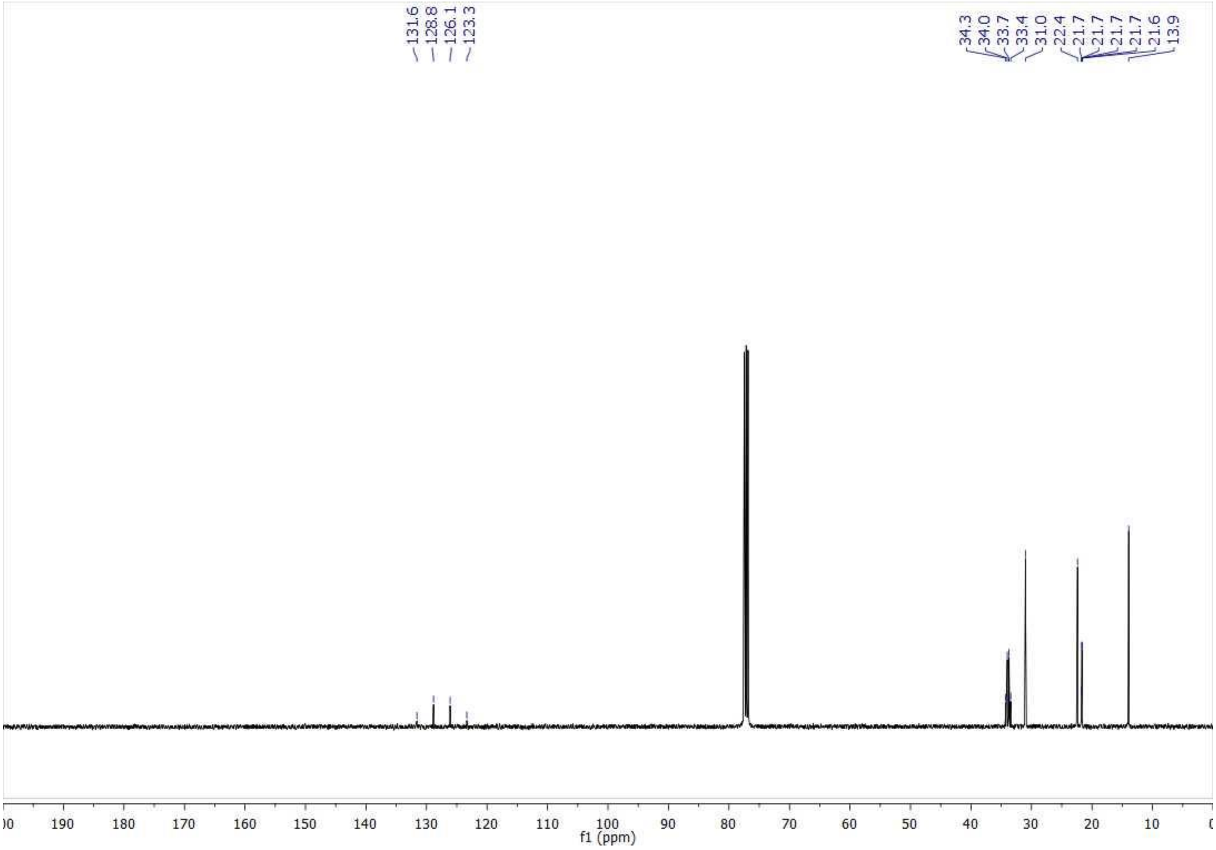

^{19}F NMR (282 MHz, $CDCl_3$)

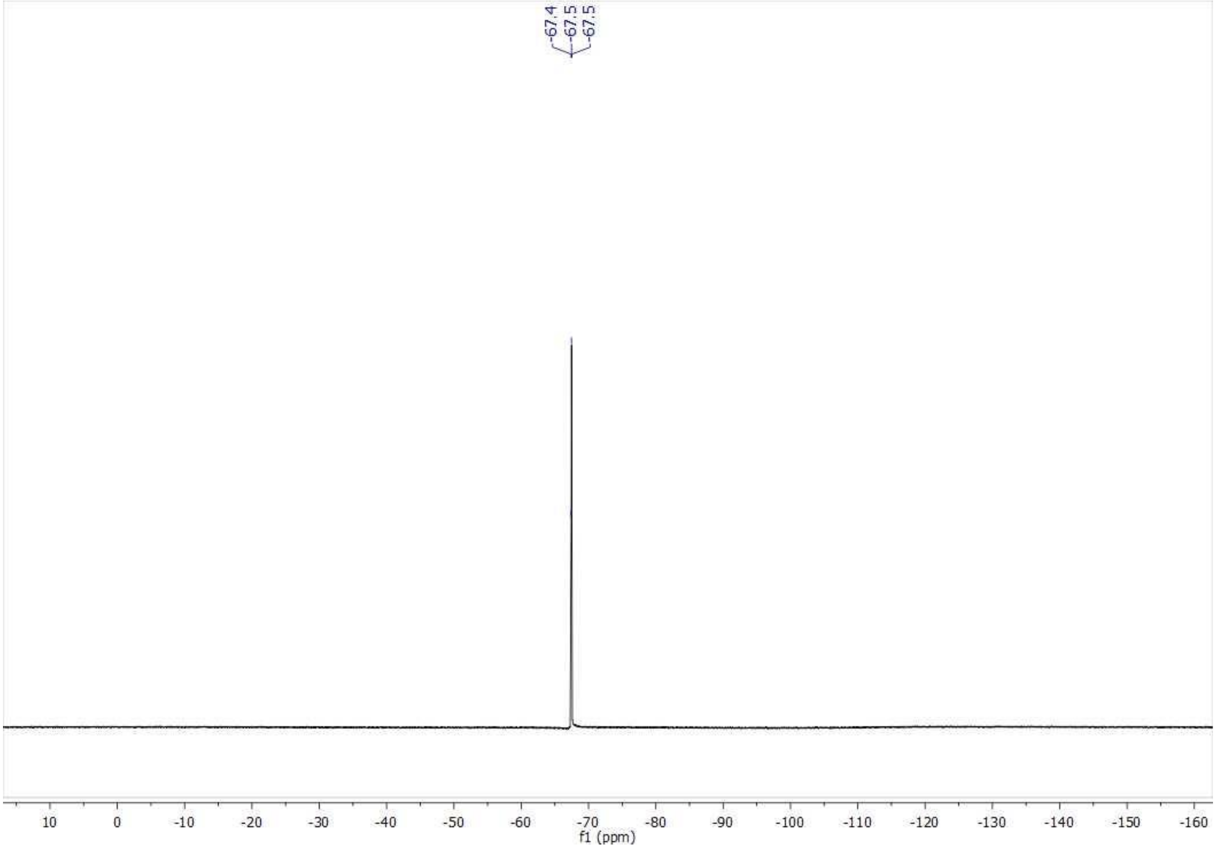

1,1,1-trifluoro3-methylpentane (8)

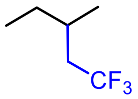

8

^{1}H NMR (500 MHz, C_6D_{12})

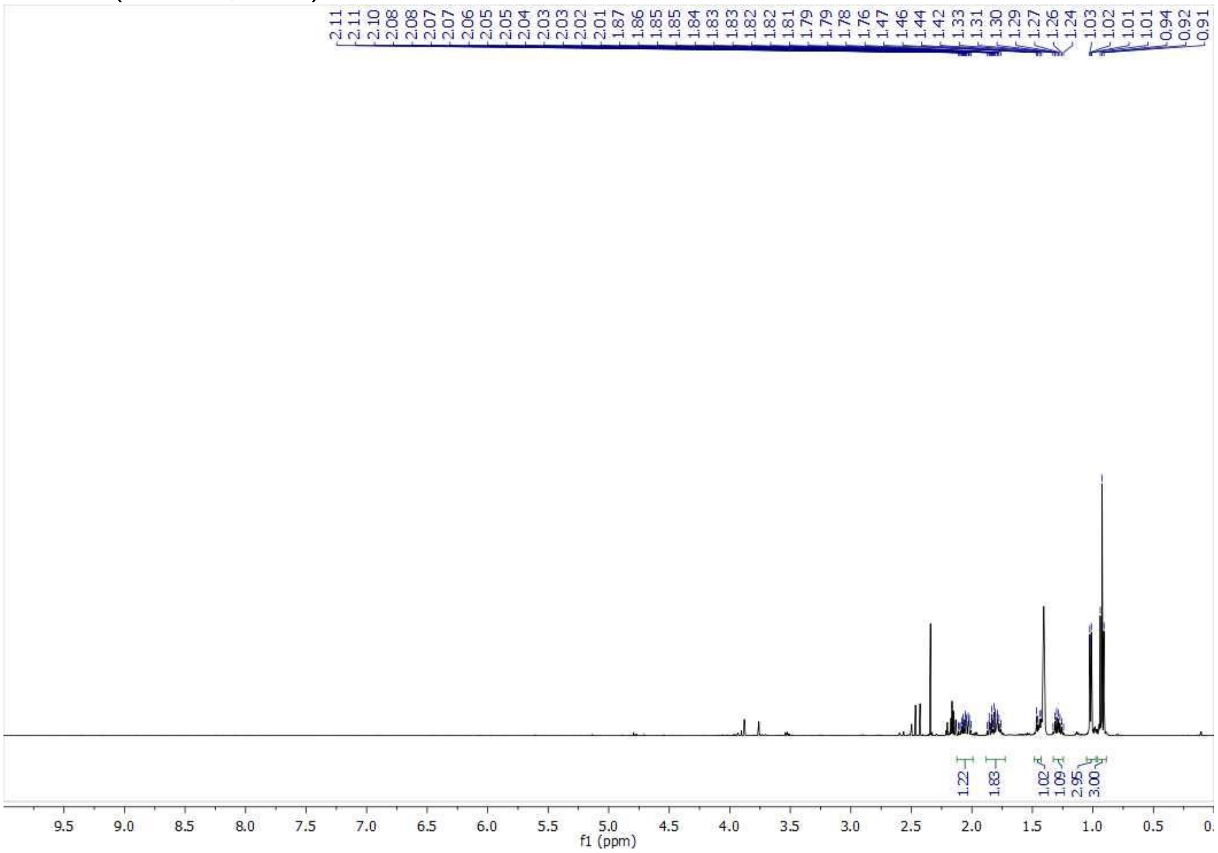

$^{13}C\{^{1}H\}$ NMR (126 MHz, C_6D_{12})

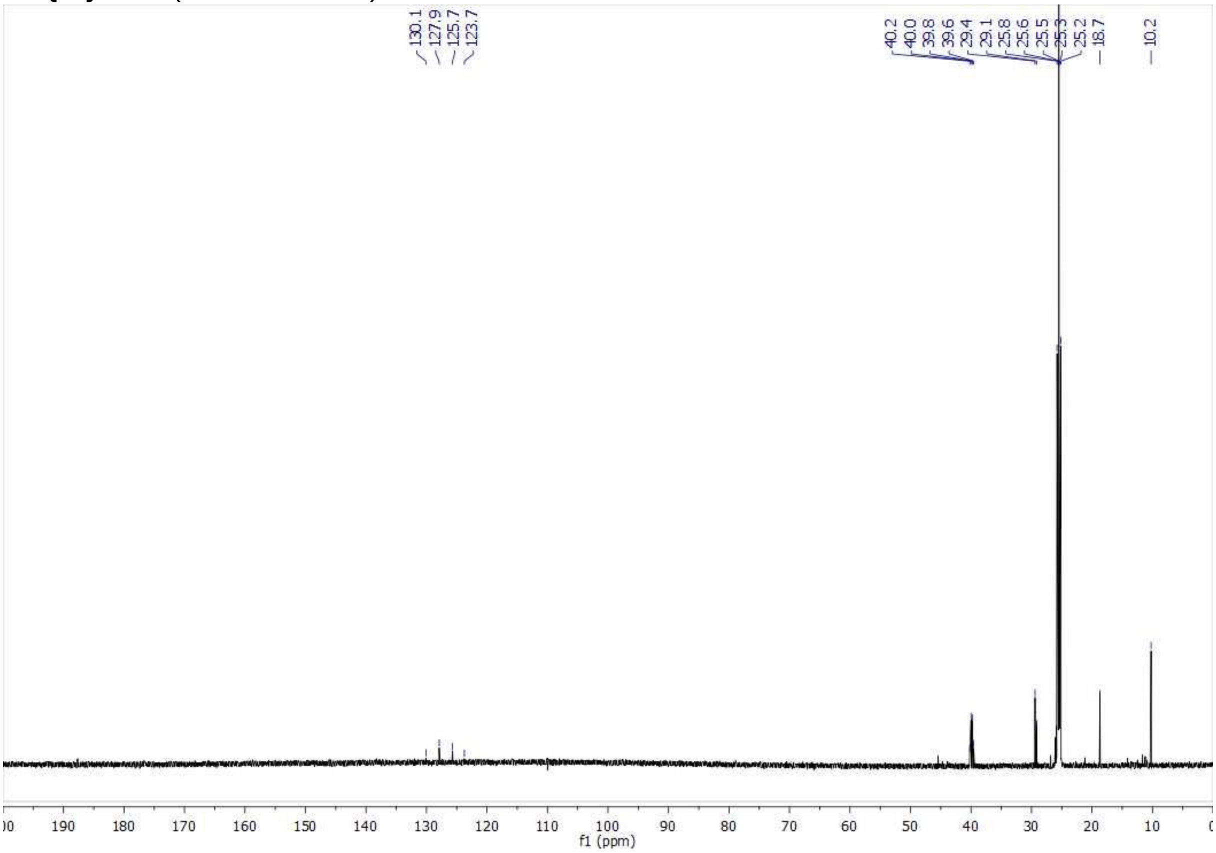

^{19}F NMR (282 MHz, C_6D_{12})

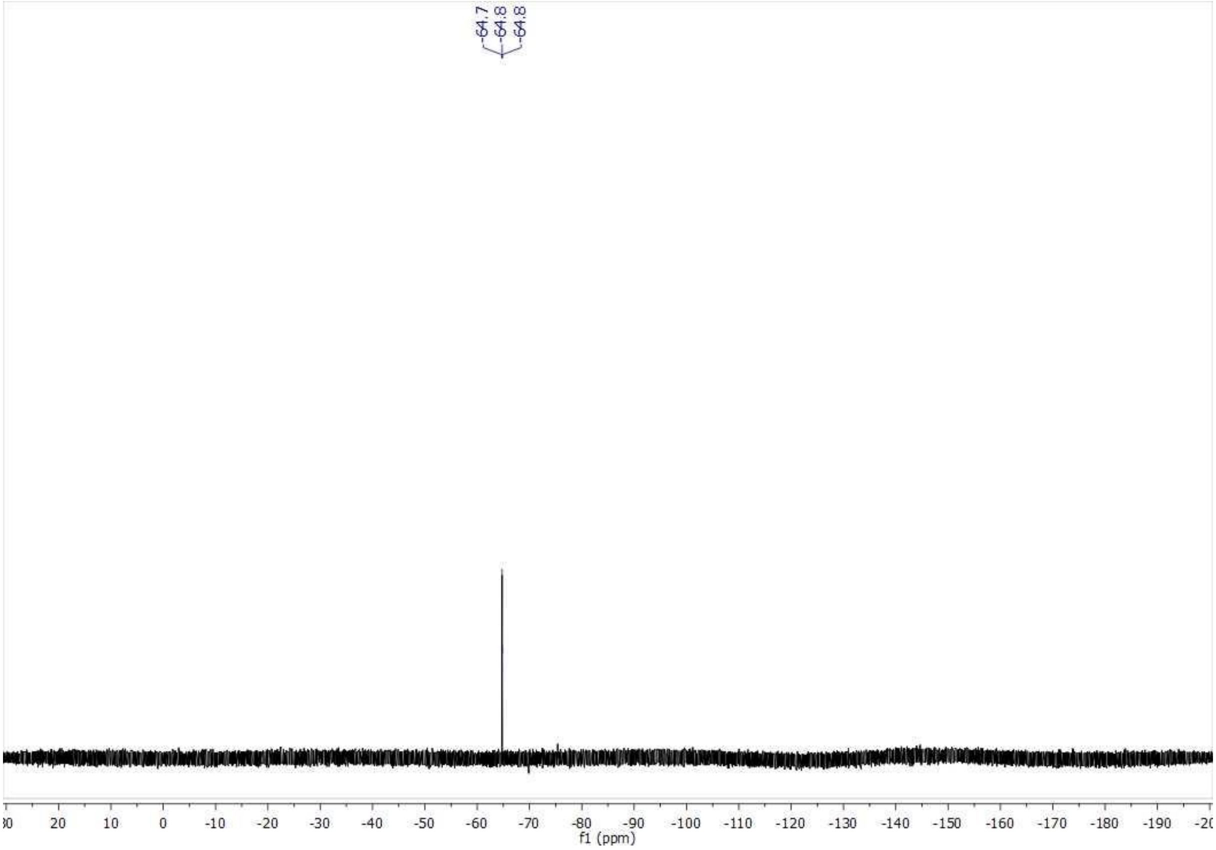

1,1,1-trifluoro-4-methylpentane (9)

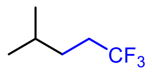

9

^{1}H NMR (500 MHz, C_6D_{12})

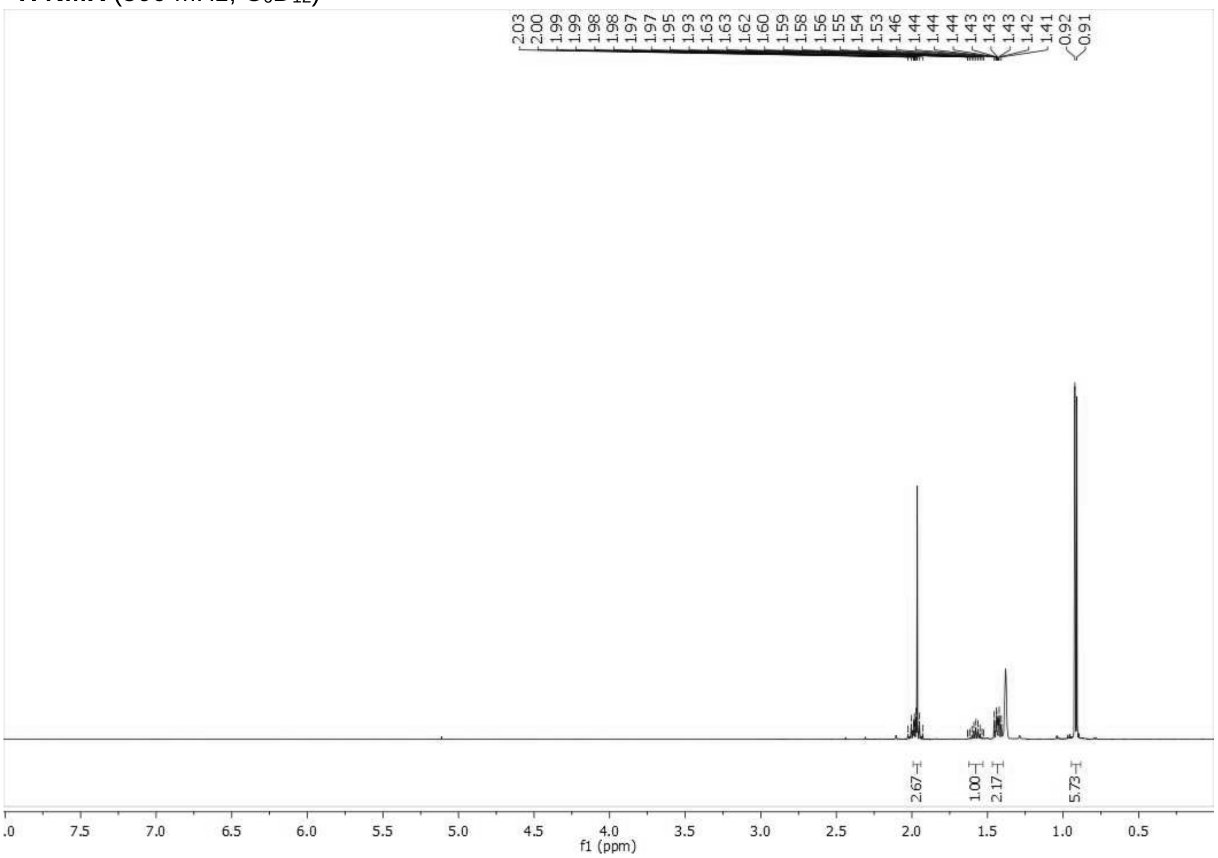

$^{13}C\{^1H\}$ NMR (126 MHz, C_6D_{12})

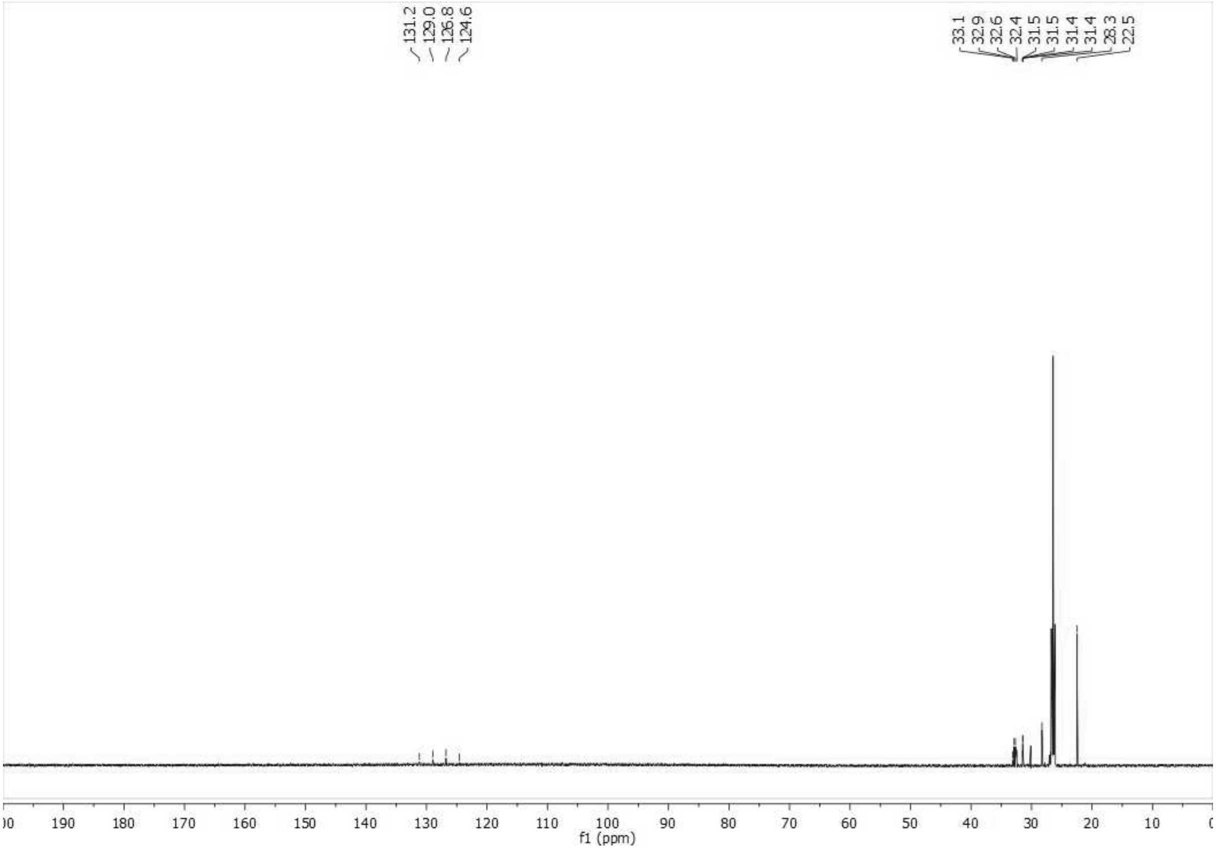

^{19}F NMR (376 MHz, $CDCl_3$)

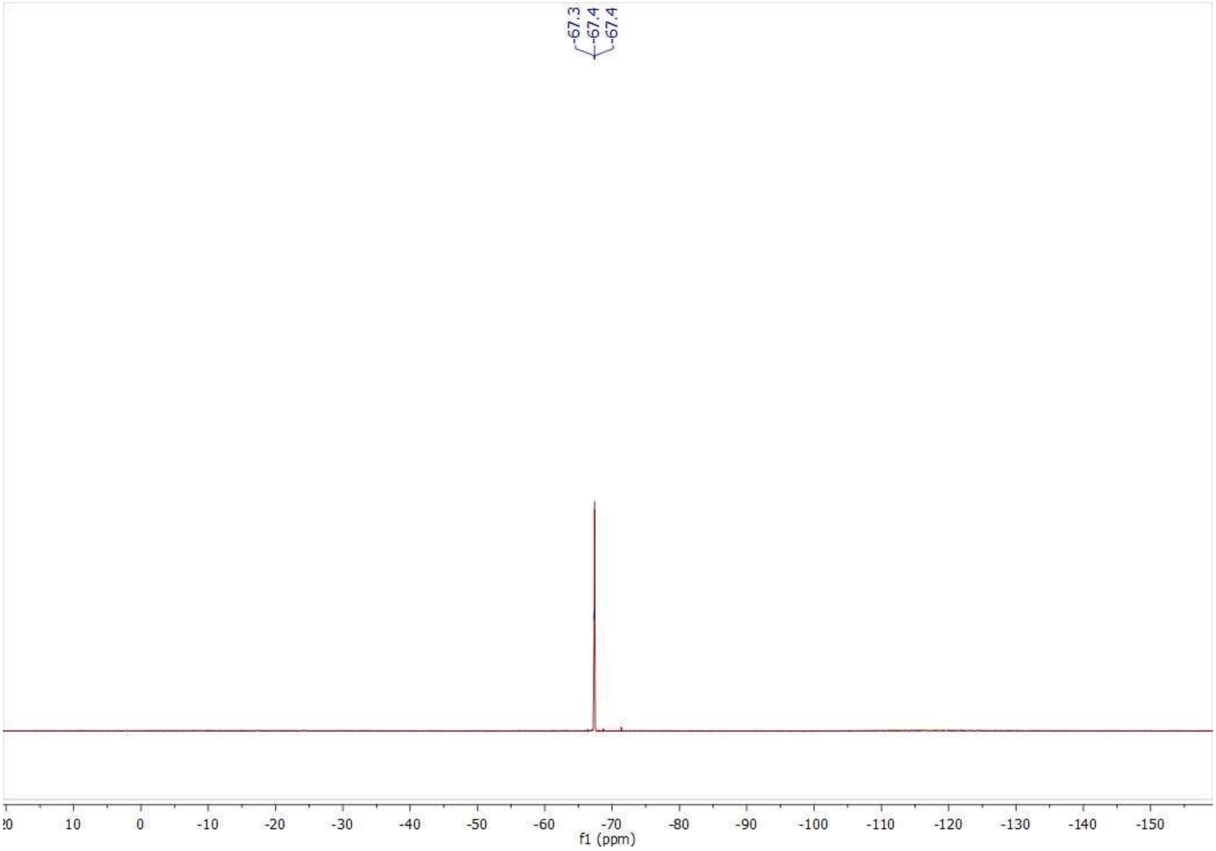

1,1,1-trifluoro-3,3-dimethylbutane (10)

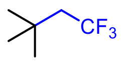

10

^{1}H NMR (500 MHz, C_6D_{12})

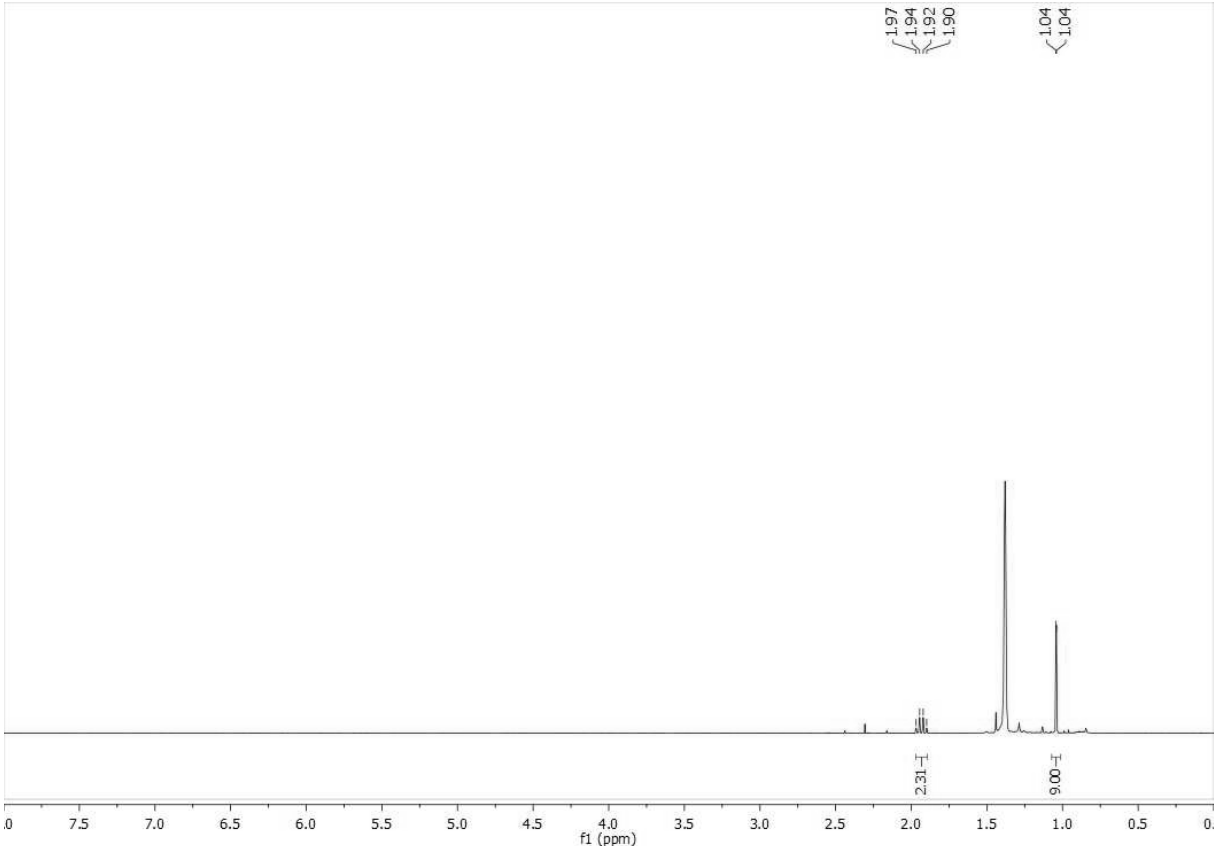

$^{13}C\{^{1}H\}$ NMR (126 MHz, C_6D_{12})

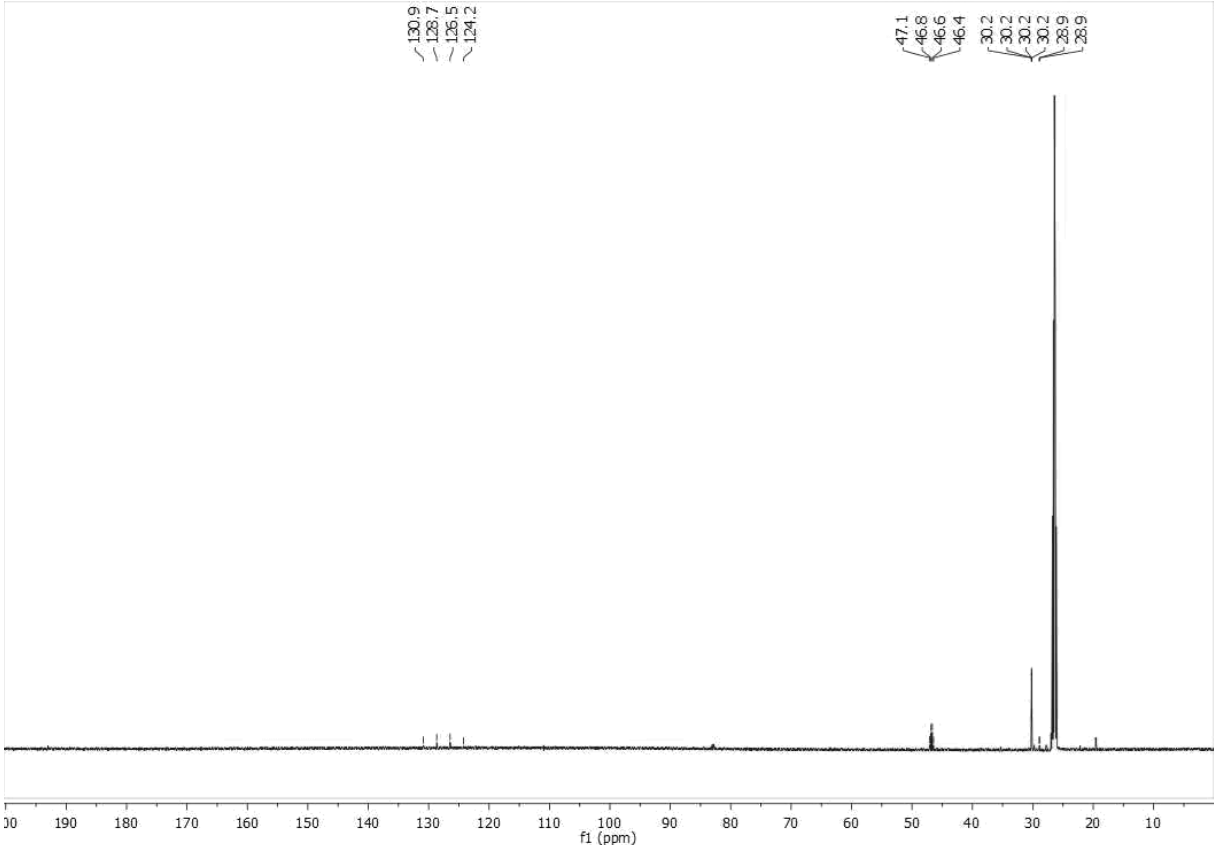

^{19}F NMR (376 MHz, $CDCl_3$)

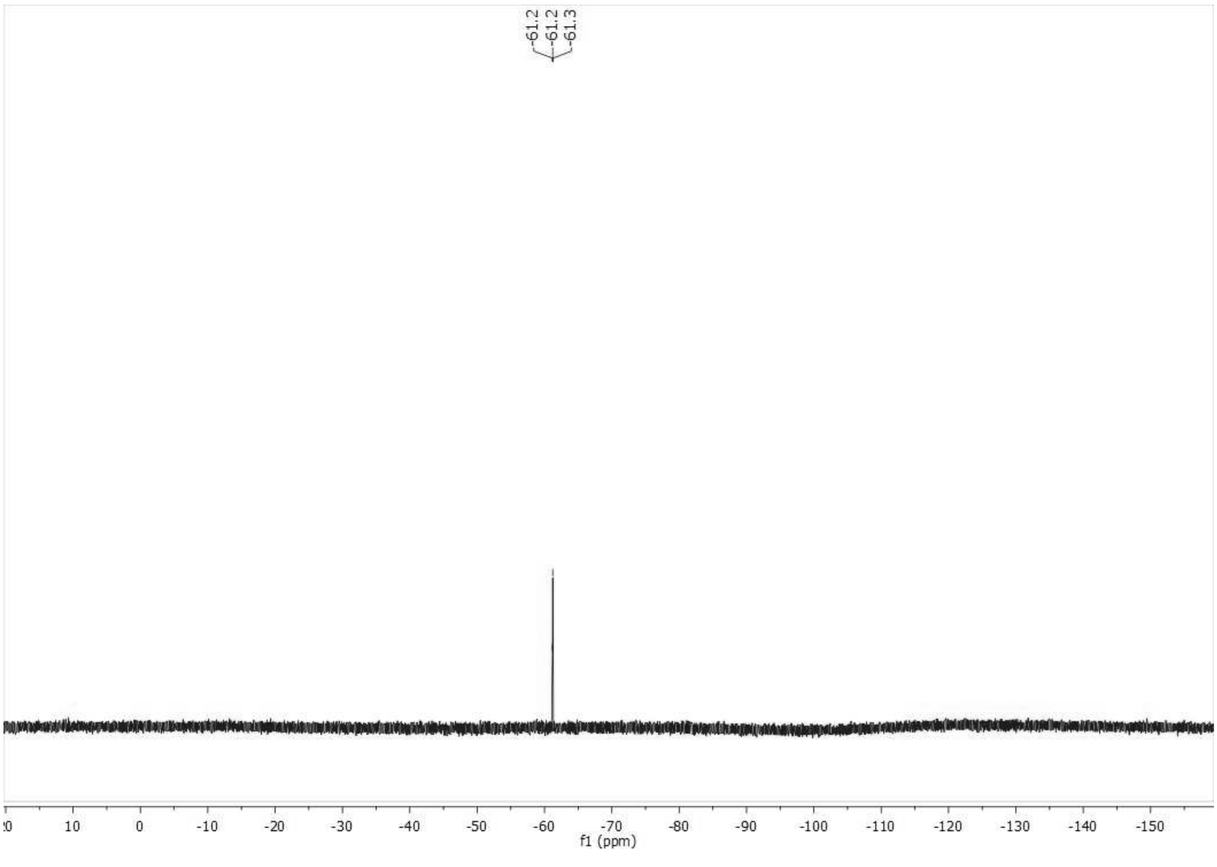

1,1,1-trifluoroheptane (11)

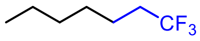

11

^{1}H NMR (500 MHz, C_6D_{12})

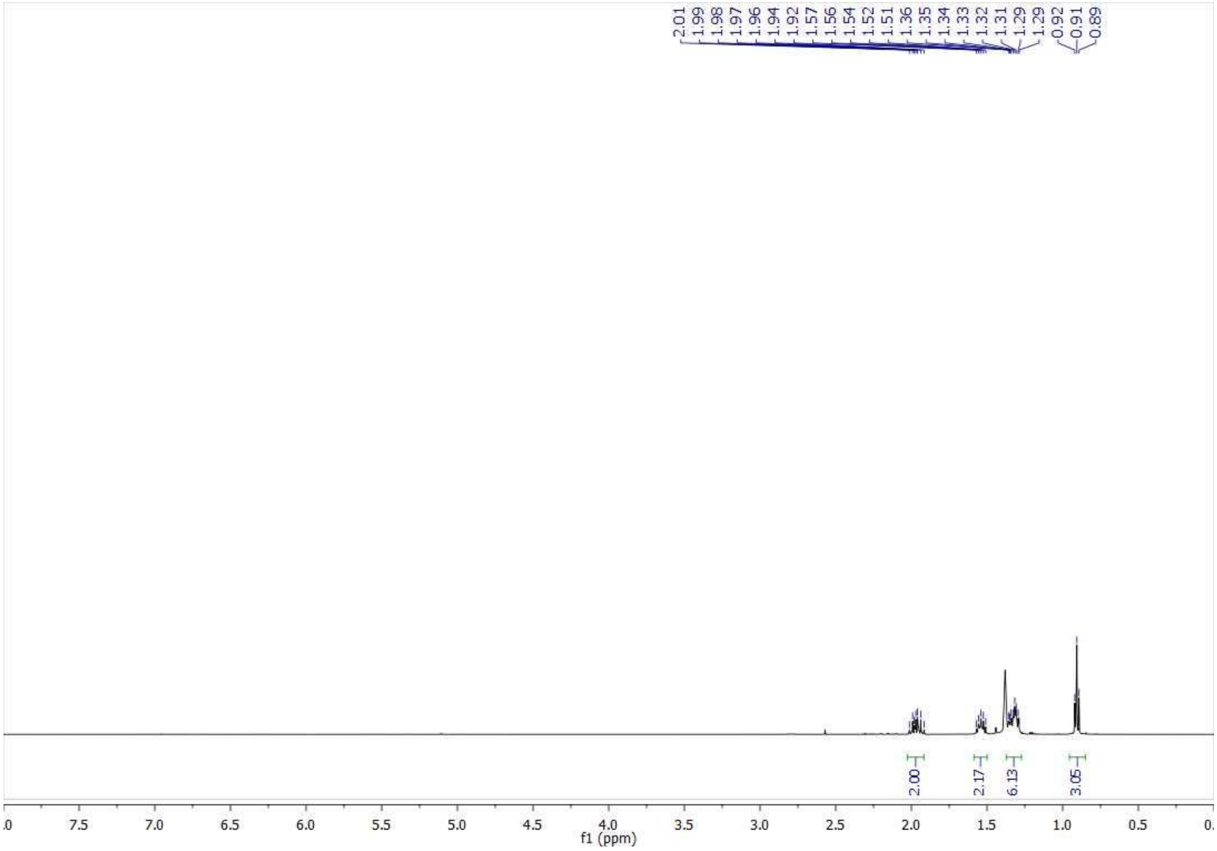

$^{13}C\{^{1}H\}$ NMR (126 MHz, C_6D_{12})

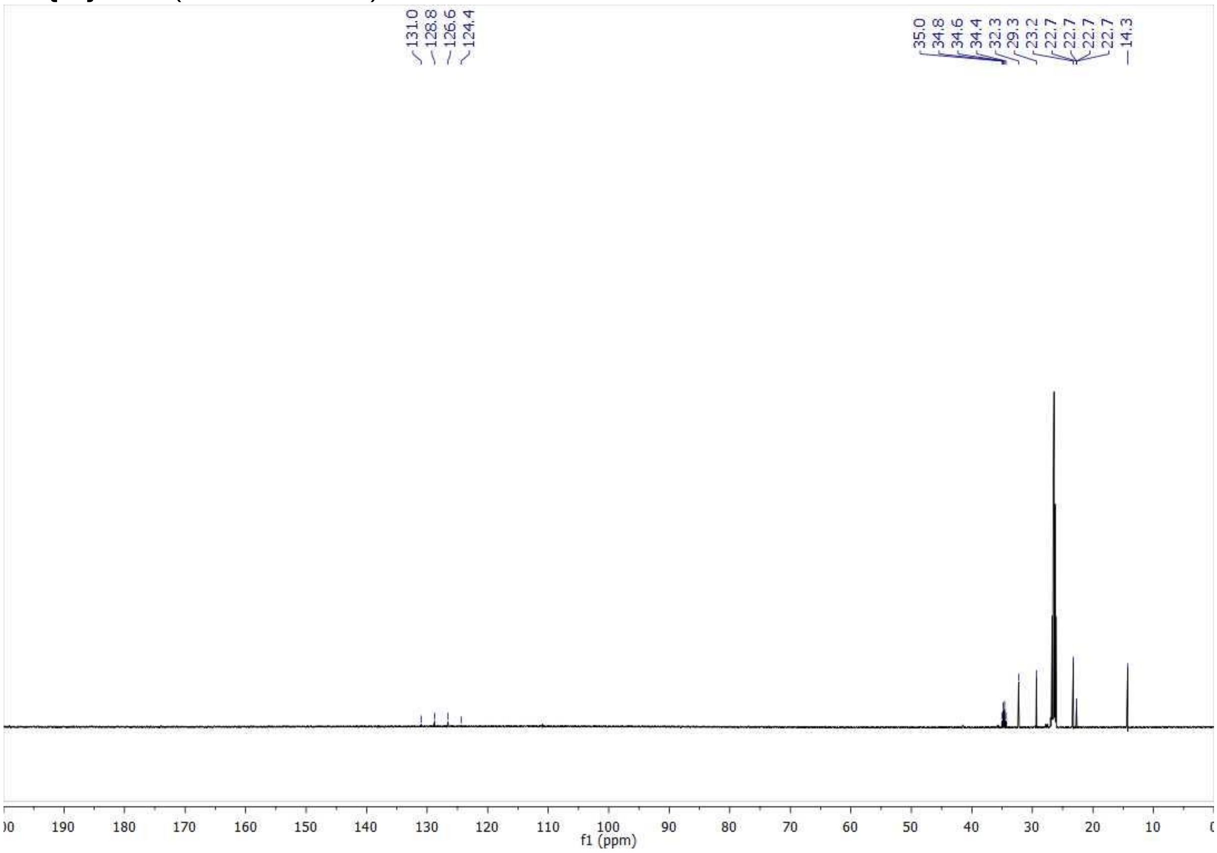

^{19}F NMR (282 MHz, $CDCl_3$)

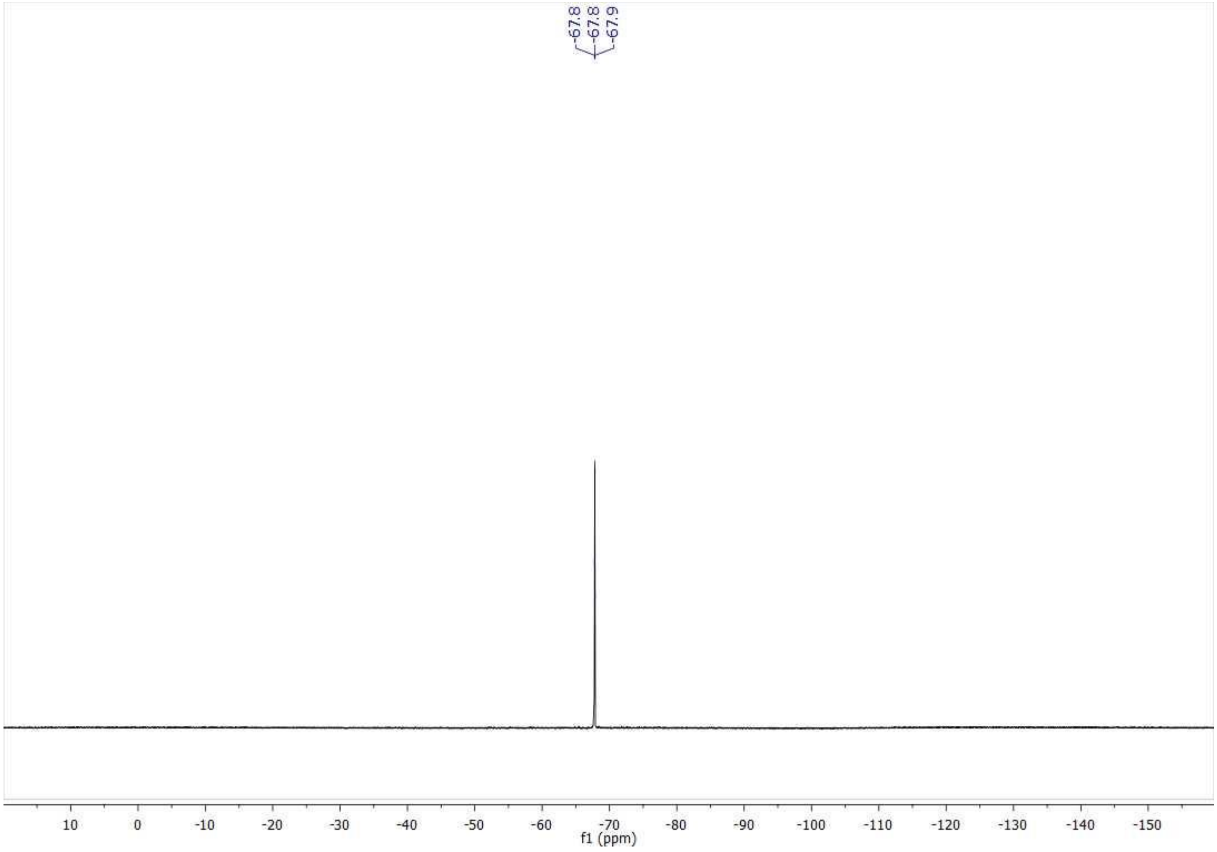

1,1,1-trifluoro-3-methylhexane (12)

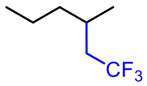

12

^{1}H NMR (500 MHz, C_6D_{12})

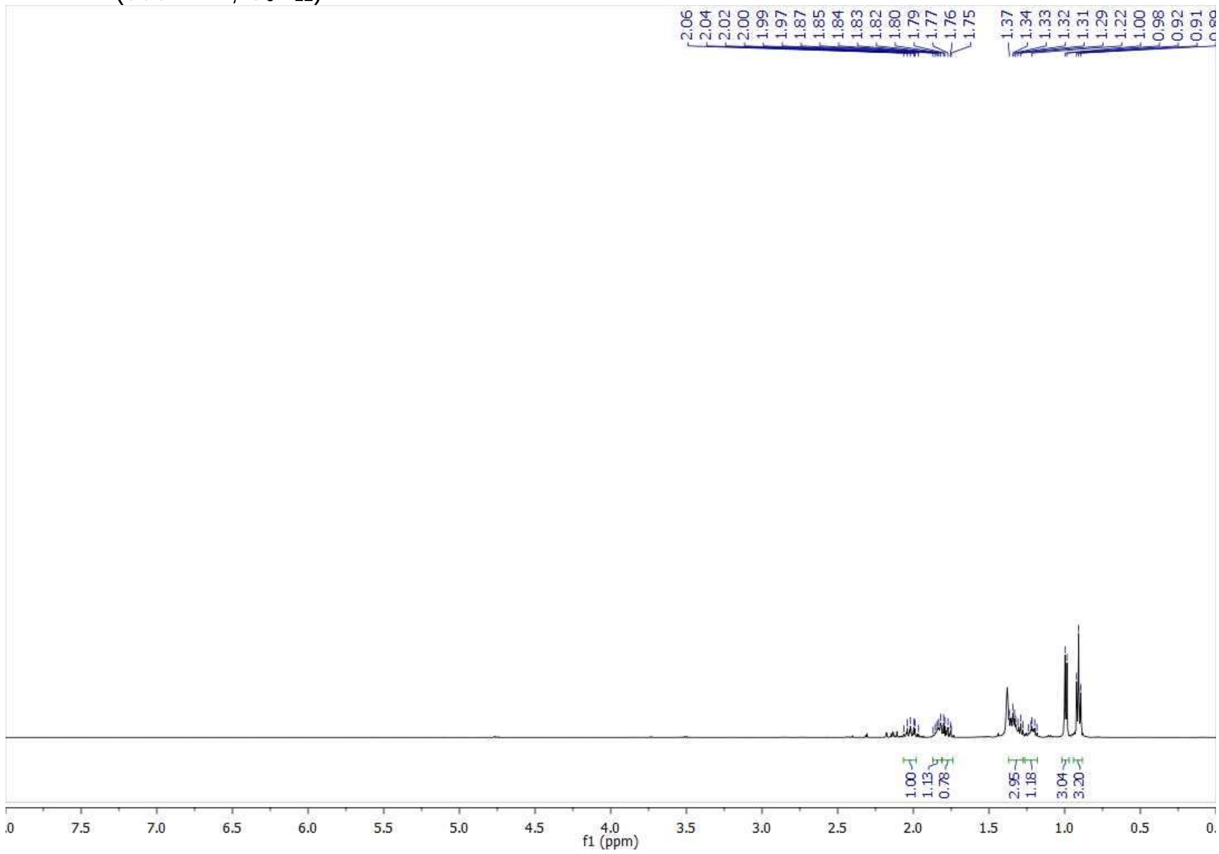

$^{13}C\{^1H\}$ NMR (126 MHz, C_6D_{12})

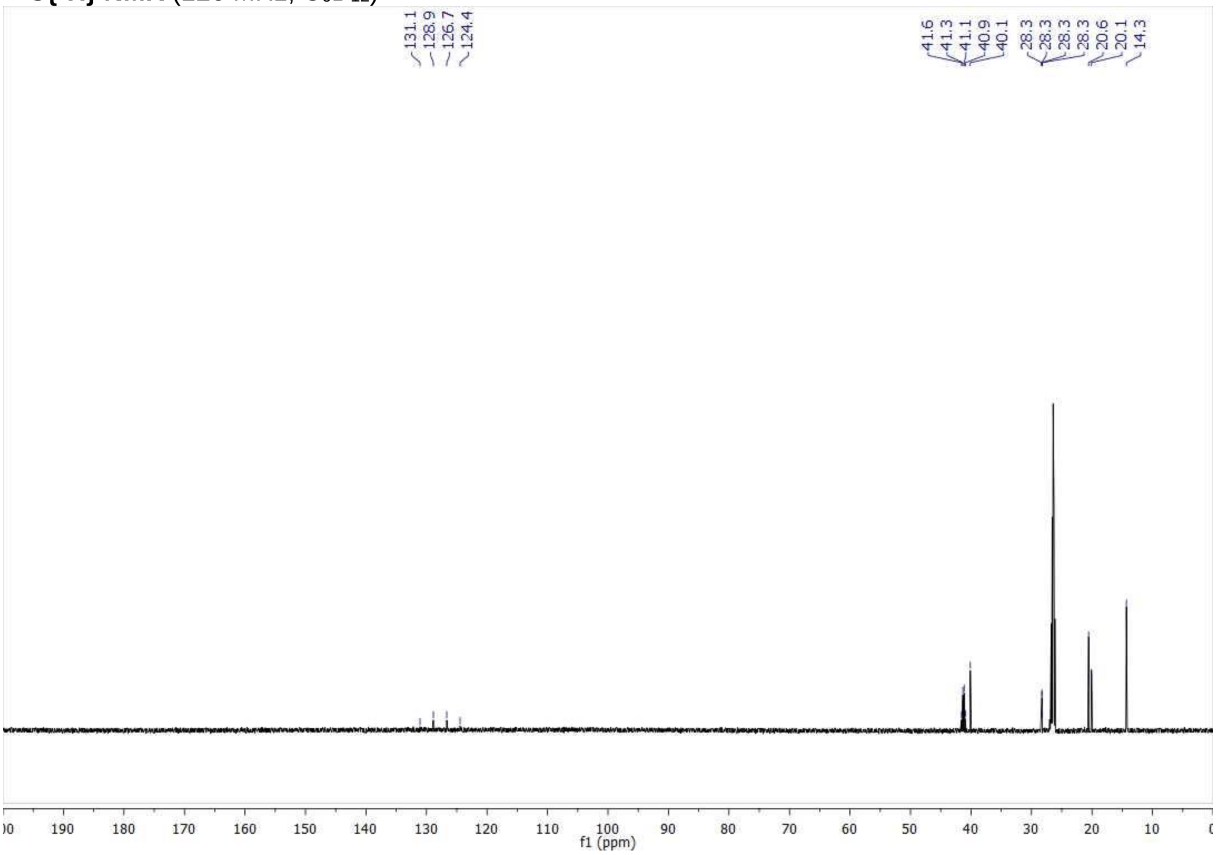

^{19}F NMR (282 MHz, C_6D_{12})

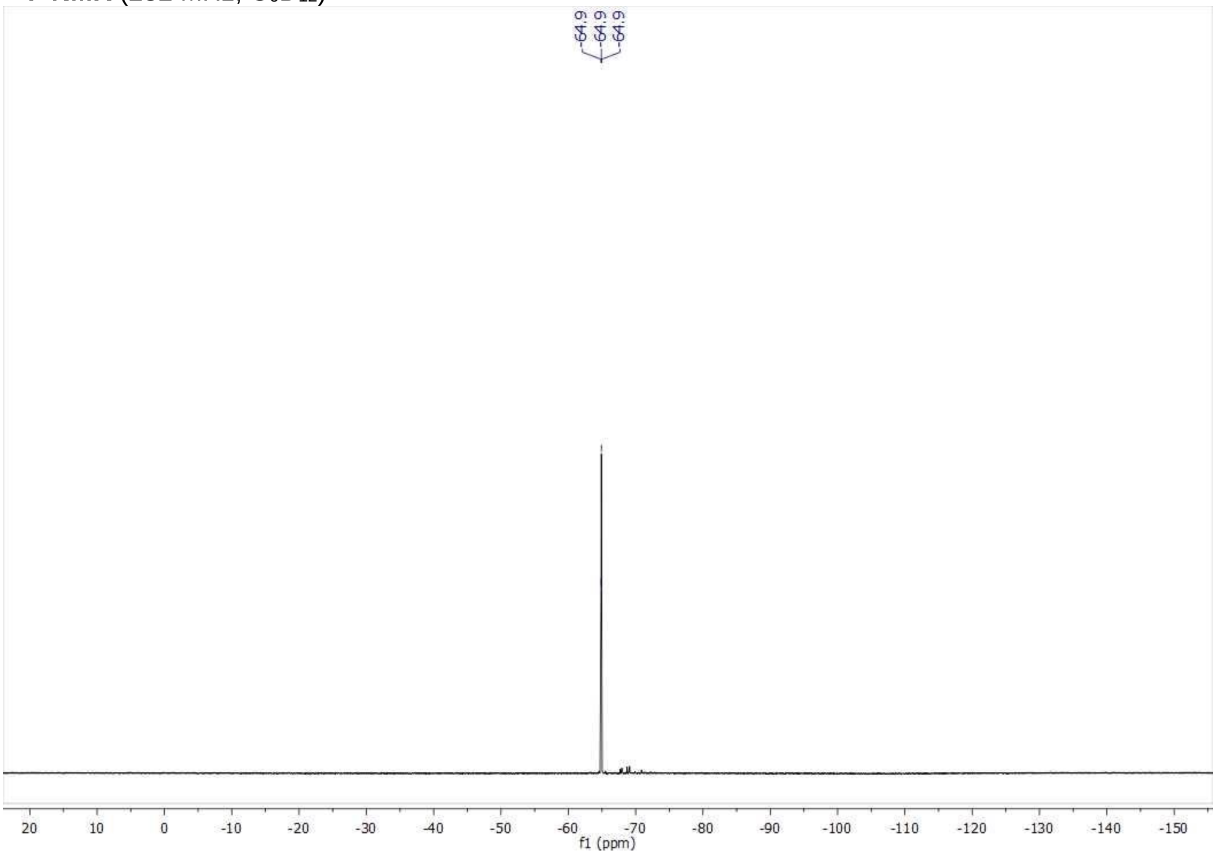

1,1,1-trifluoroethylpentane (13)

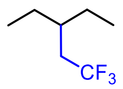

13

[1]**H NMR** (500 MHz, C_6D_{12})

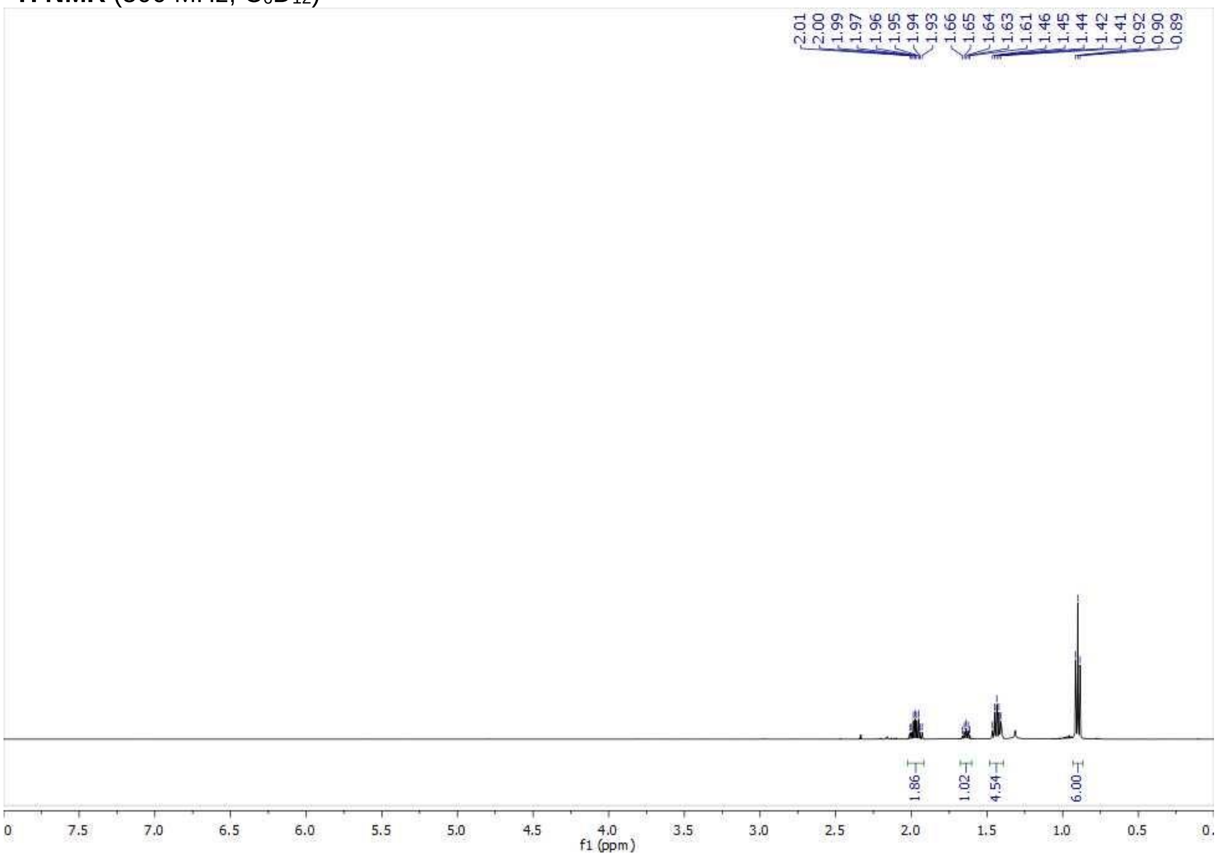

$^{13}C\{^{1}H\}$ NMR (126 MHz, C_6D_{12})

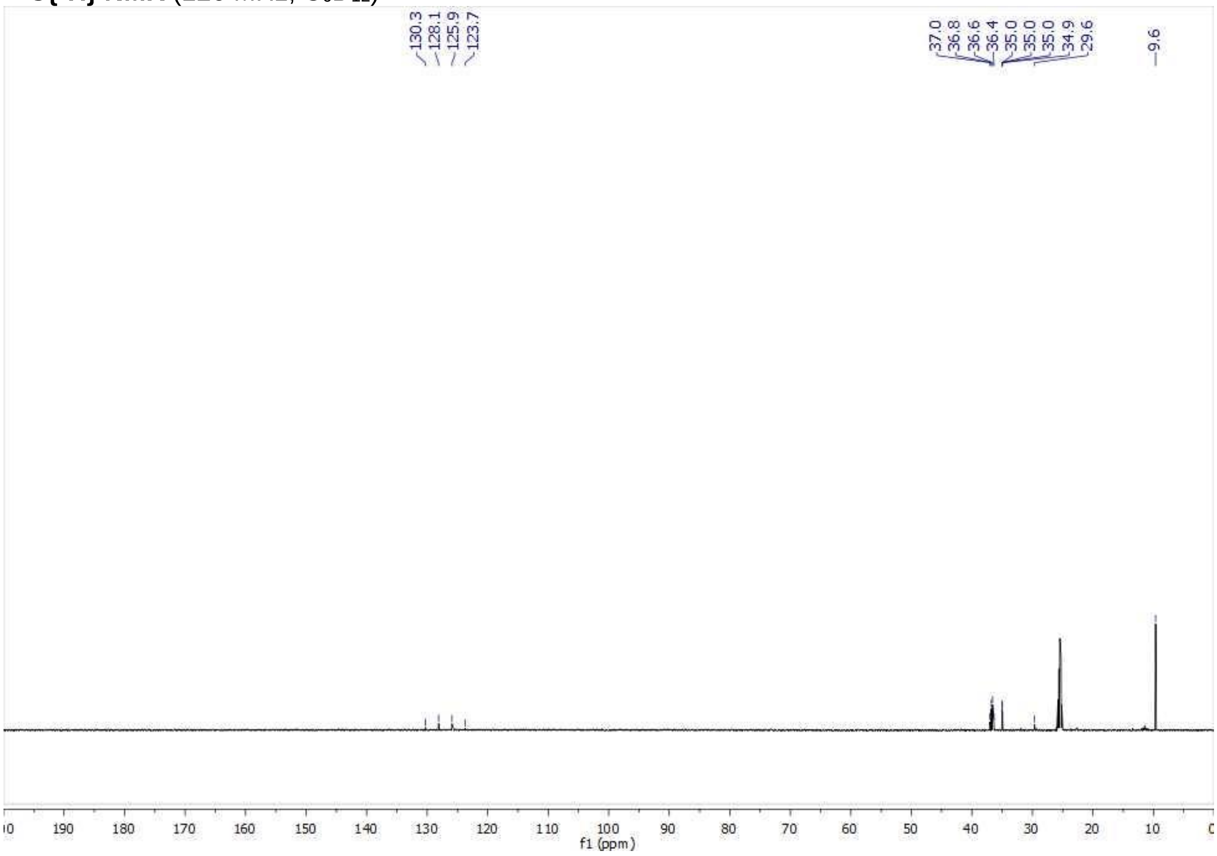

^{19}F NMR (470 MHz, C_6D_{12})

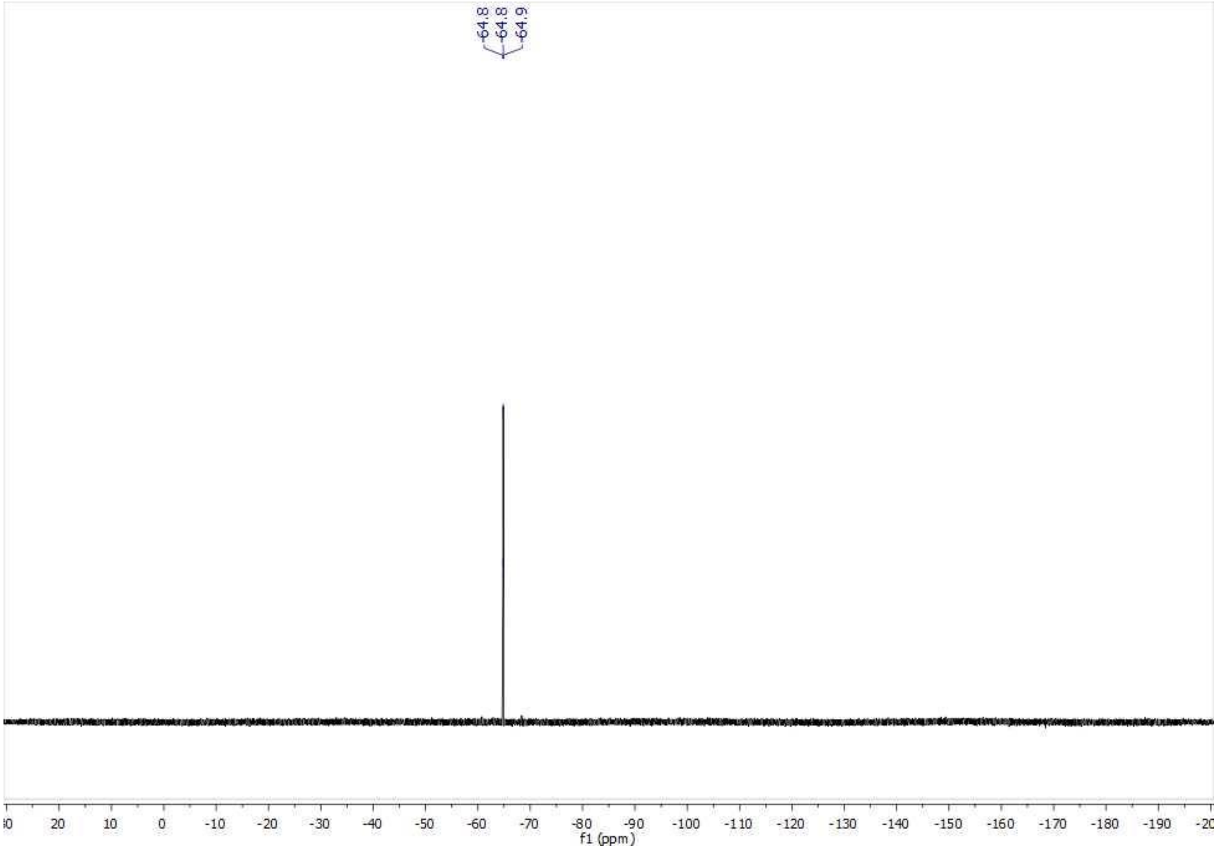

1,1,1-trifluorooctane (14)

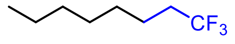

14

^{1}H NMR (500 MHz, C_6D_{12})

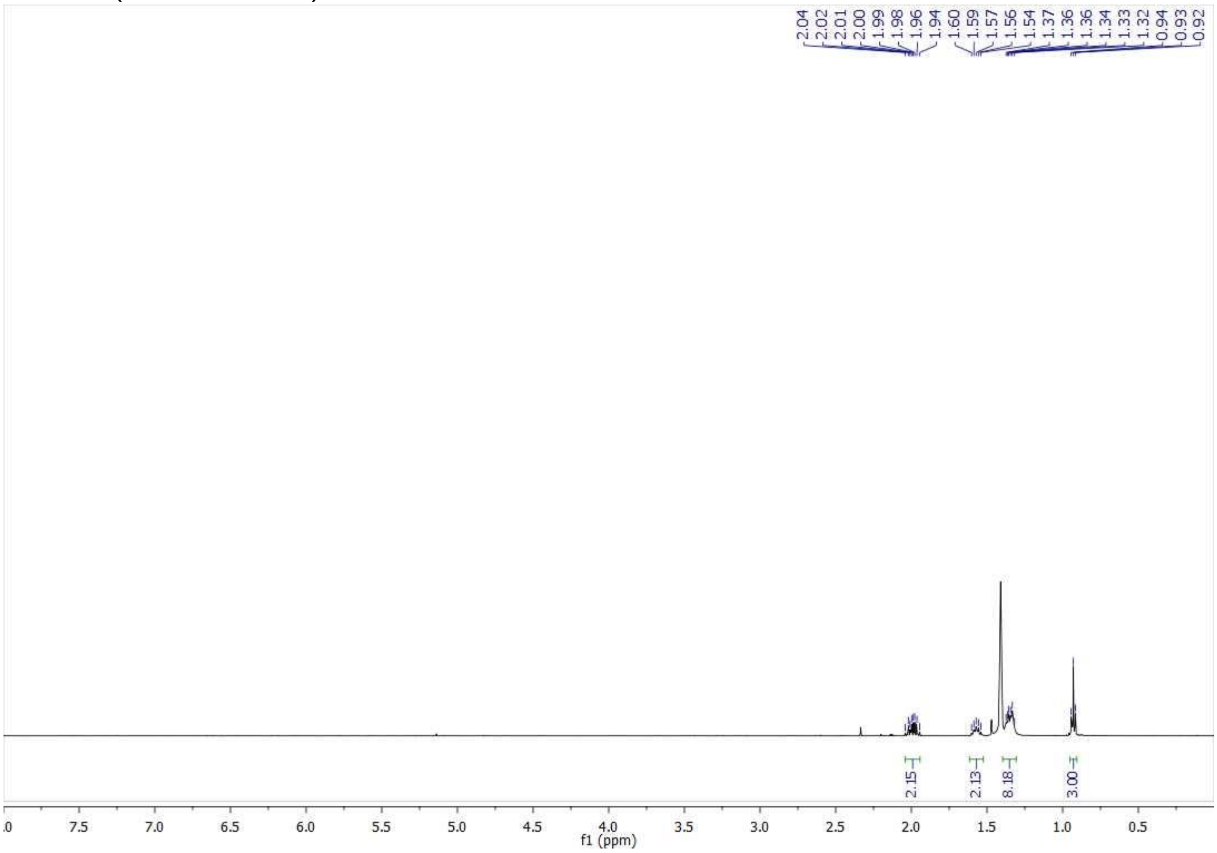

$^{13}C\{^1H\}$ NMR (126 MHz, C_6D_{12})

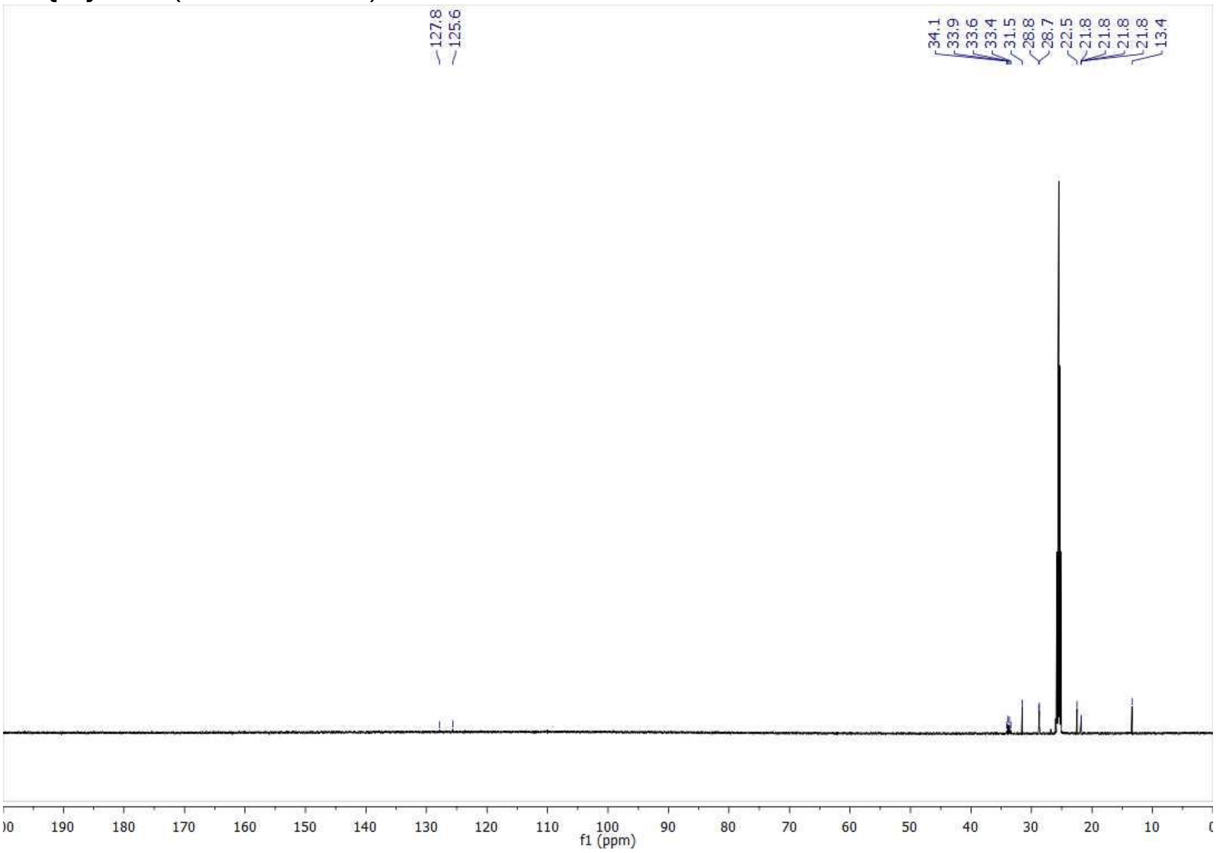

^{19}F NMR (282 MHz, $CDCl_3$)

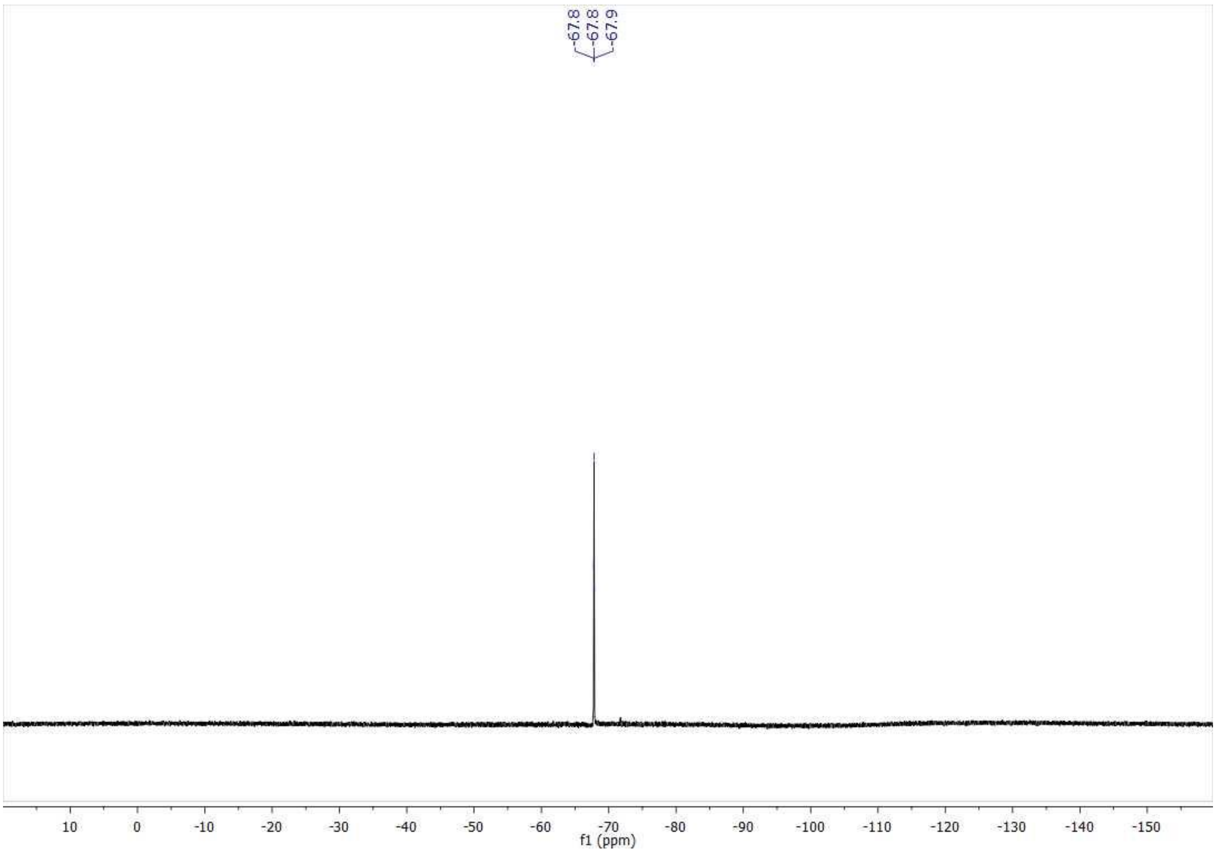

1,1,1-trifluoro-3-methylheptane (15)

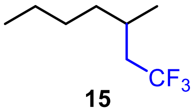

15

^{1}H NMR (500 MHz, C_6D_{12})

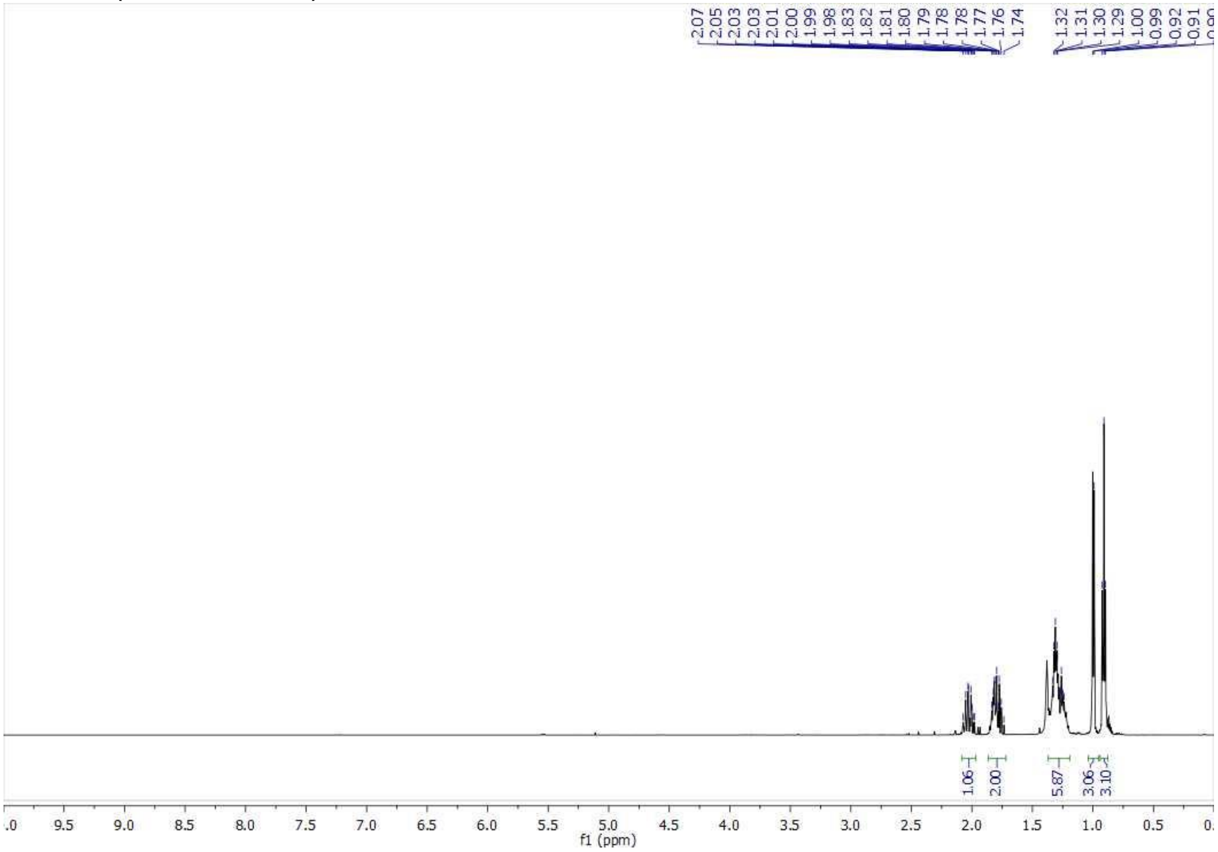

$^{13}C\{^1H\}$ NMR (126 MHz, C_6D_{12})

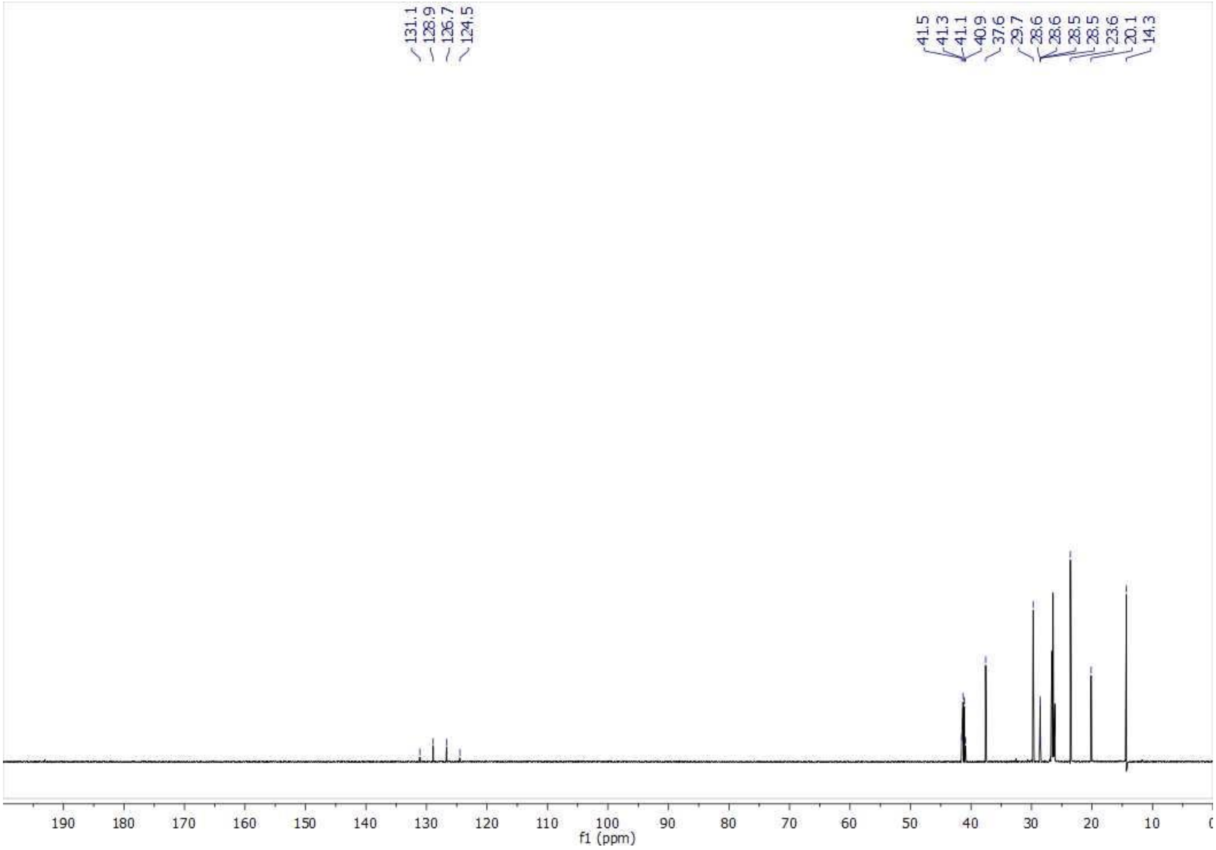

^{19}F NMR (282 MHz, C_6D_{12})

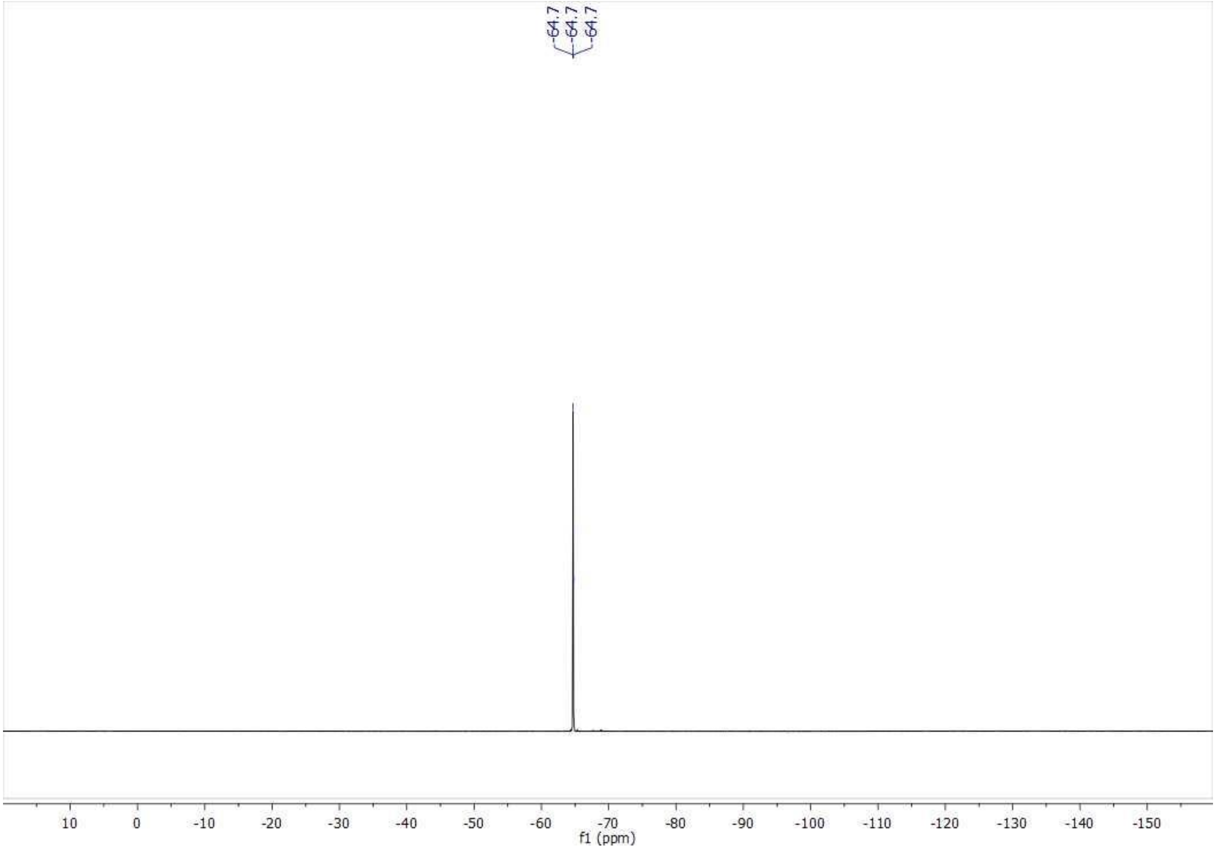

1,1,1-trifluoro-3-ethylhexane (16)

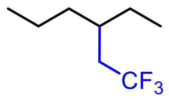

16

^{1}H NMR (500 MHz, C_6D_{12})

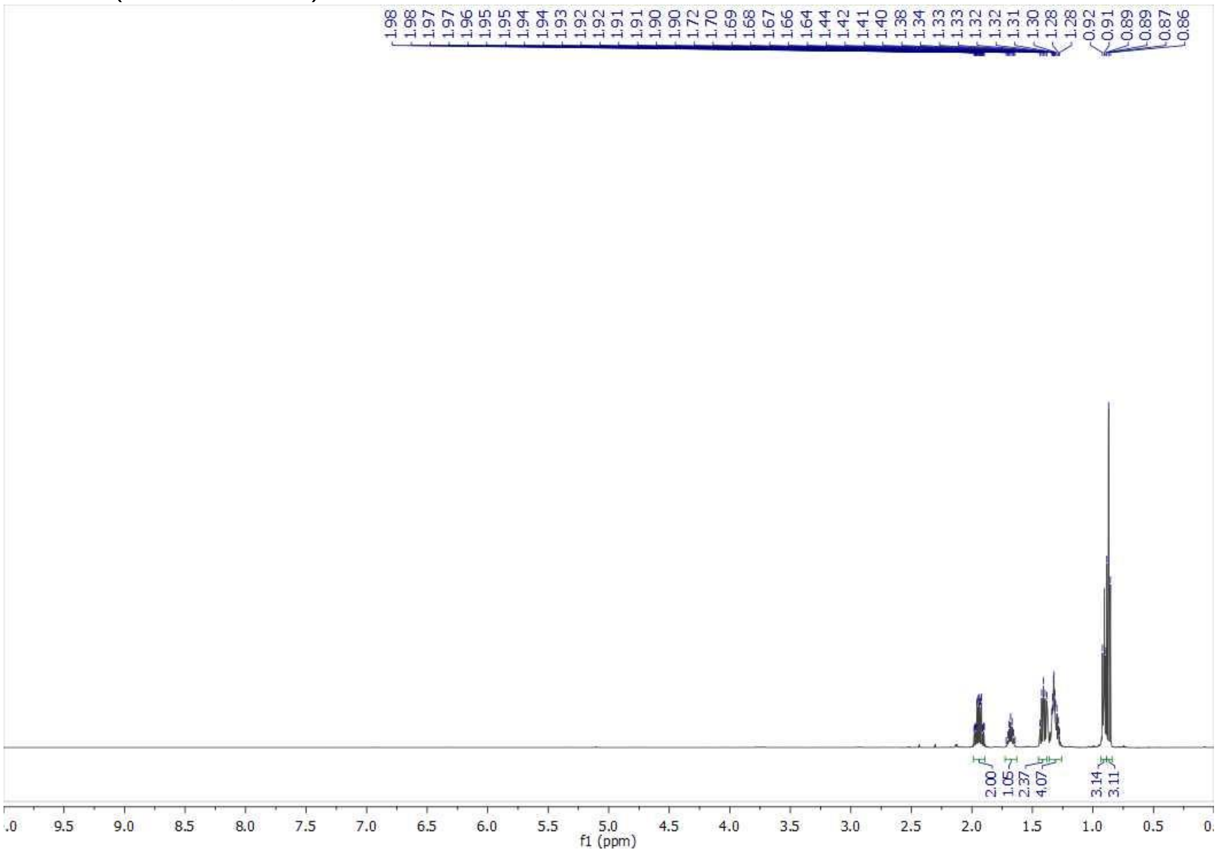

$^{13}C\{^{1}H\}$ NMR (126 MHz, C_6D_{12})

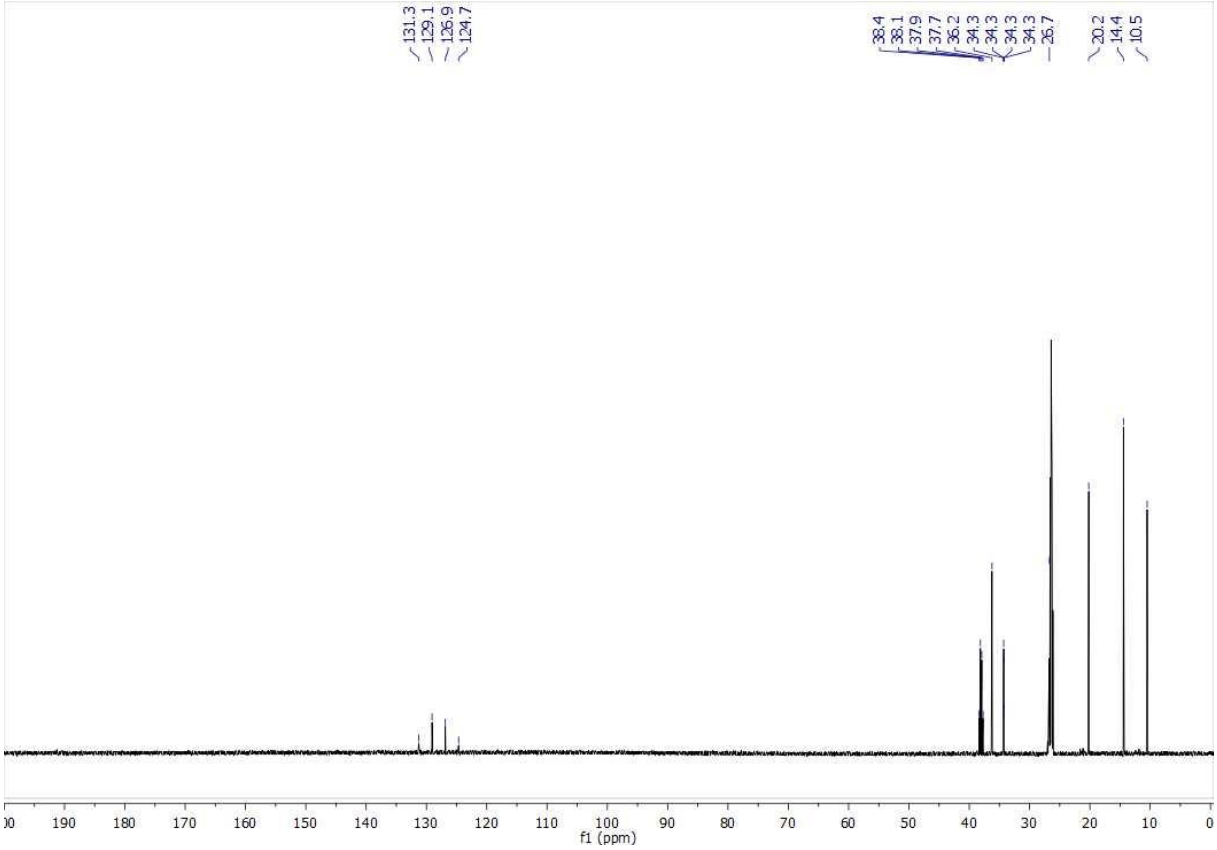

^{19}F NMR (282 MHz, C_6D_{12})

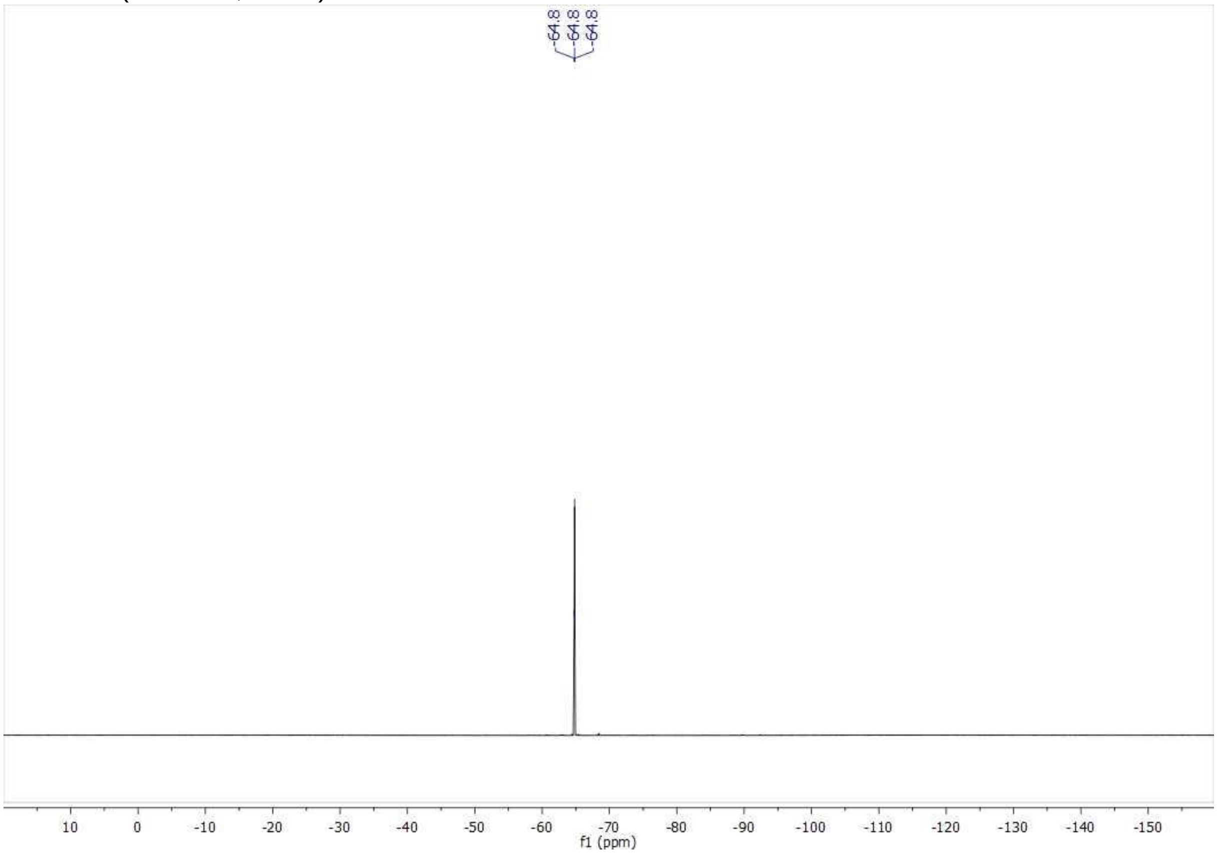

1,1,1-trifluoroethylcyclohexane (17)

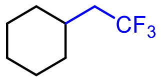

17

^{1}H NMR (500 MHz, C_6D_{12})

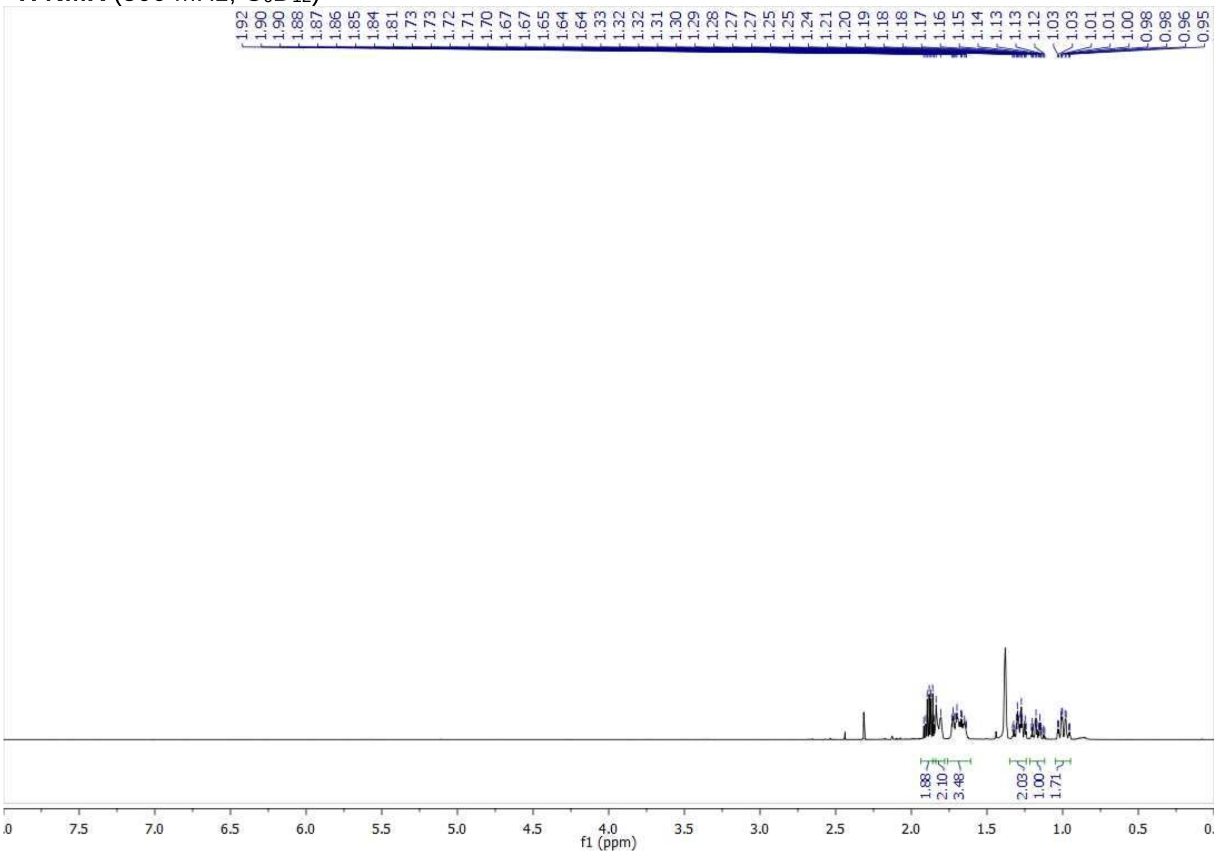

$^{13}C\{^1H\}$ NMR (126 MHz, C_6D_{12})

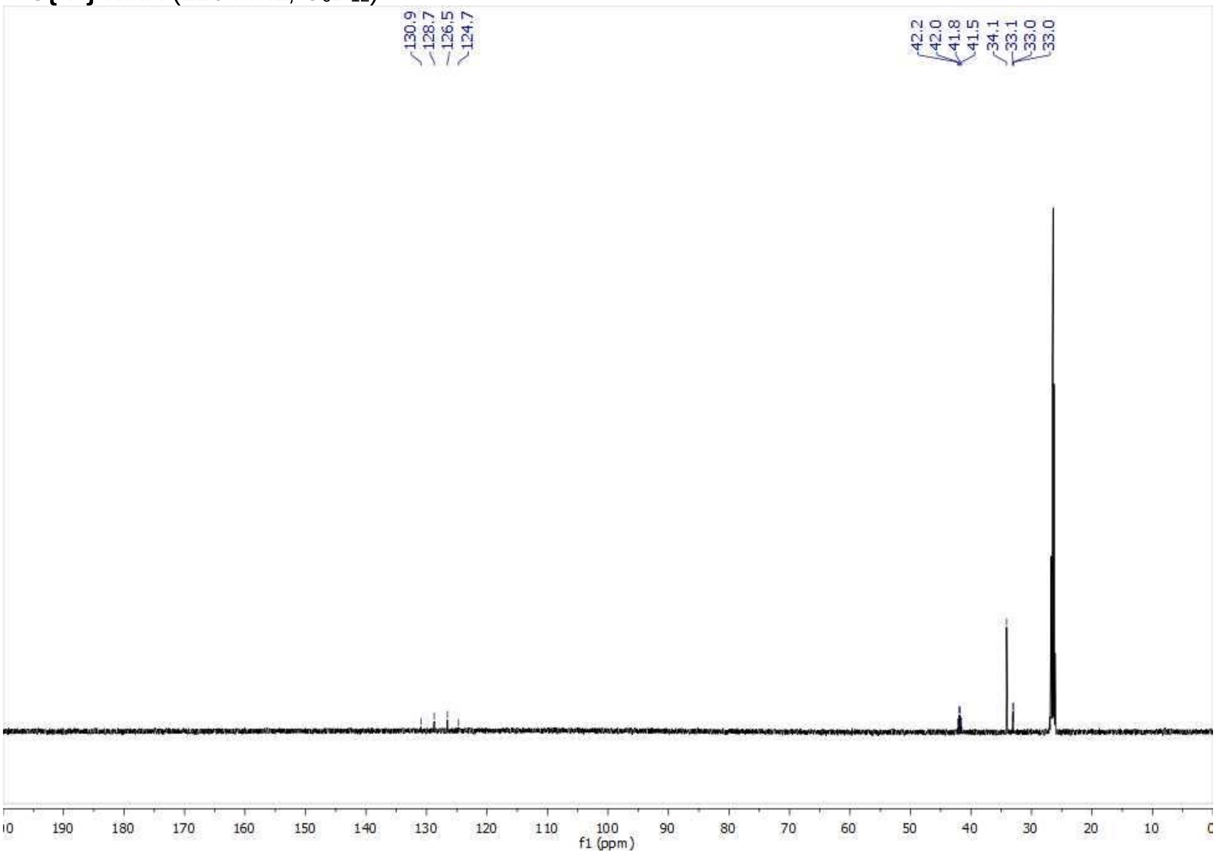

^{19}F NMR (470 MHz, C_6D_{12})

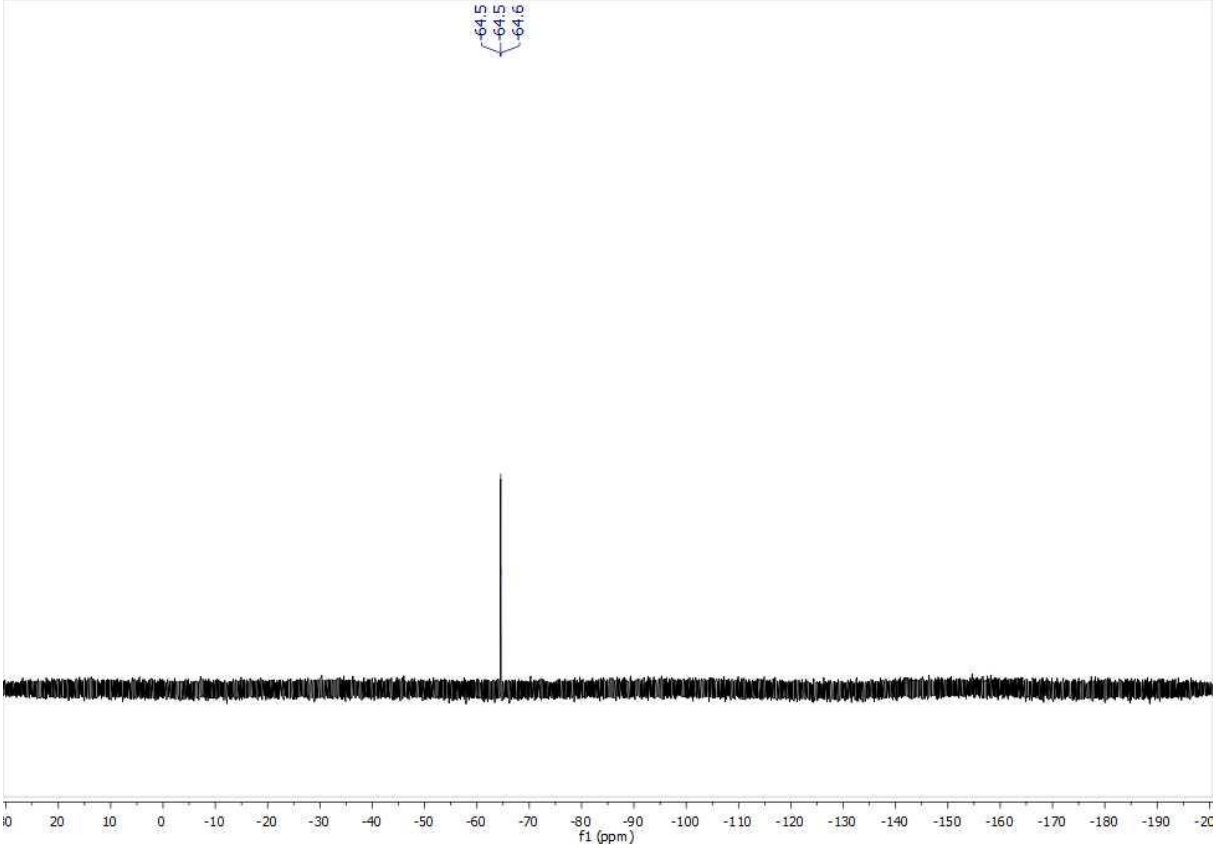

7. GC traces for methane and ethane functionalization

GC trace for methane functionalization employing TFDE and catalyst 1

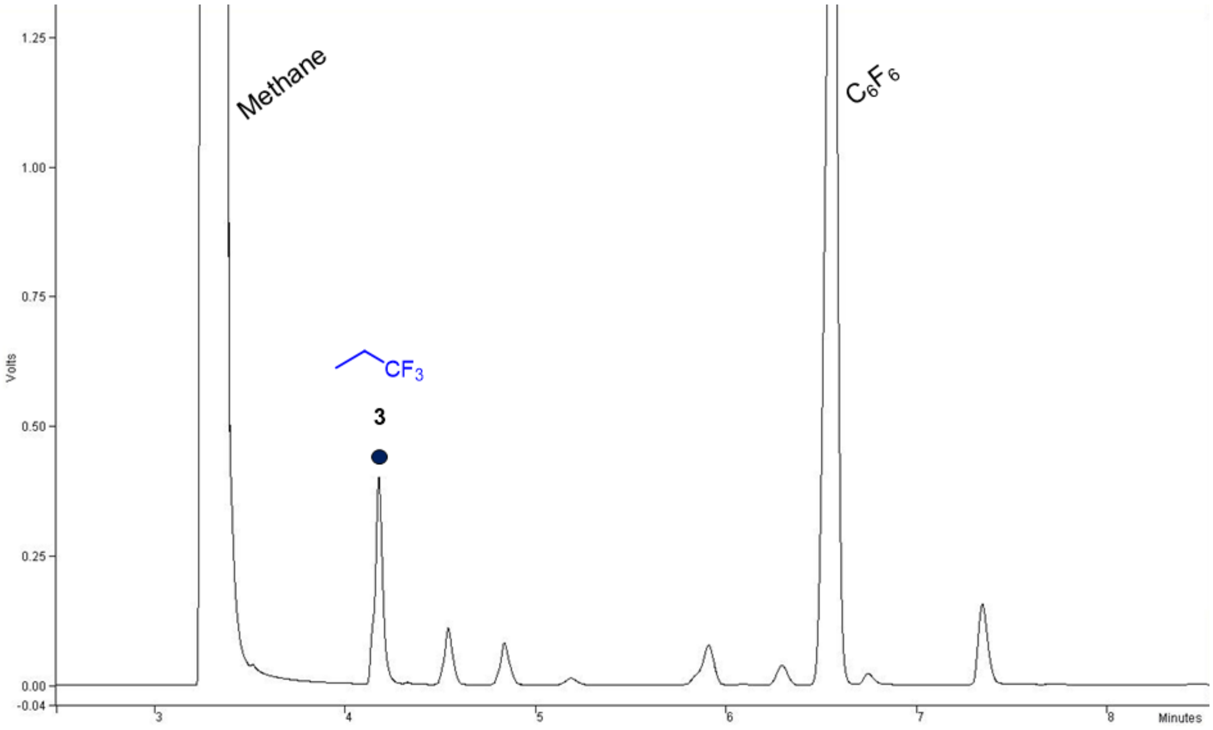

GC trace for ethane functionalization employing TFDE and catalyst 1

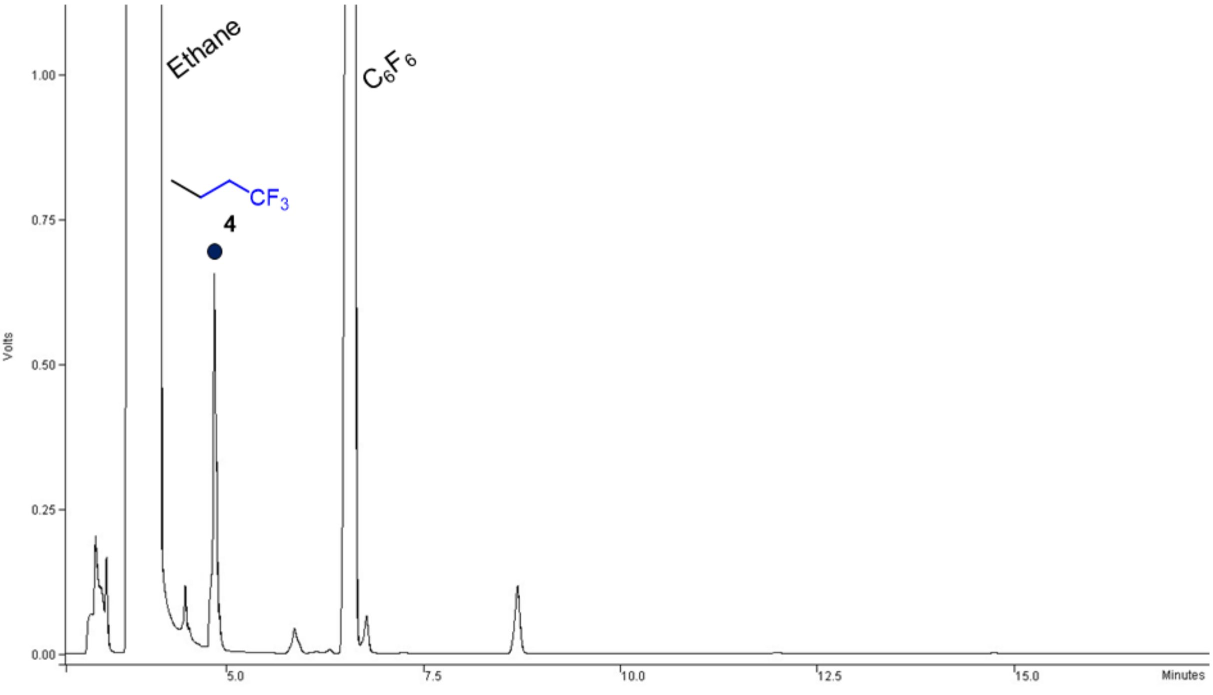

8. Computational details

Theoretical mechanistic analysis was performed at DFT level of theory with Gaussian16 software,[1] using B3LYP functional[2] combined with the Grimme's D3 correction for dispersion.[3] Structures of all the intermediates and transition states were optimized with basis set BS1 in n-hexane solvent (ε = 1.88) using the SMD continuum model.[4] BS1 set of basis functions includes the 6-31G(d,p) basis set for the main group elements[5] while LANL2DZ including pseudopotential for Ag and Br atoms.[6] For Br atoms was included an additional set of d and p polarization functions.[7] Frequency calculations, at the same level of theory, were carried out for all the stationary points in order to characterize them as either minima or transition states. Connection of transition states with the corresponding intermediates was confirmed by usual intrinsic reaction coordinate (IRC) calculations and subsequent optimization to minima. Final Gibbs energies in solvent at 298.15 K were obtained adding the thermal and entropic corrections obtained with BS1 to the electronic energy in solvent computed with single point calculations on the BS1 optimized geometries, using an extended basis set (BS2). BS2 consists in the triple triple-ζ *def2*-TZVP basis set for the main group elements and Br atoms and the quadruple-ζ *def2*-QZVP basis set for Ag.[8,9] A correction of 1.9 kcal mol^{-1} was applied to all Gibbs energy values to change the standard state from the gas phase (1 atm) to solution (1 M) at 298.15 K.[10]

The Molecular Dynamics (MD) simulations under periodic boundary conditions will be carried out initially along 200 ns via the OpenMM engine[11] through OMMProtocol.[12] The General Amber Force Field (GAFF2) was used for the organic ligands and solvent (propane), while Ag-bonding force constants and equilibrium parameters were obtained through the Seminario method, using the MCPB.py module.[13] Metal centers parametrization was based on the optimized geometries and harmonic frequencies of the [Ag]=C(H)CF_3 carbene.

MDs were carried out solvating the system in a cubic box of explicit propane molecules. The box dimension was set to fit the experimental the concentration of liquid propane corresponding to 50x50x50 Å with 837 propane molecules. The resulting systems were then subjected to a minimization followed by thermalization increasing the temperature from 0 K to 300 K (time step of 20 ps), followed by 100 ns of NPT production using the Langevin thermostat under periodic boundary conditions with electrostatic cut-off of 10.0 Å and Particle Mesh Ewald method for long range electrostatic interactions.

Analysis of the trajectories will be carried out by means of CPPTraj implemented in AmberTools16.[14]

Table SC1. B3LYP-D3 characterized transition states for CH_3–H activation over the Tp^xAg=C(R^1)R^2 and $Rh_2(Ac)_4$=C(R^1)R^2 carbenes.[a]

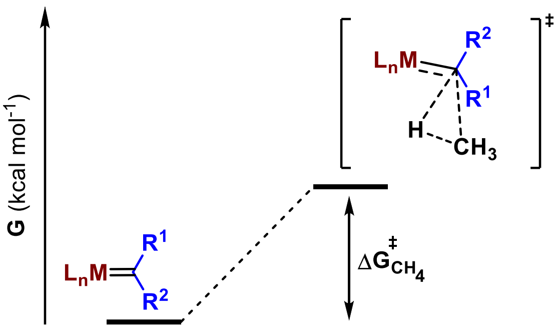

**ML**	**R^1**	**R^2**	**Species**	**ΔG_{sol} (kcal·mol^{-1})[b]**
$Tp^{Br_3}Ag$	Ph	CO_2Et	**$I^{Ethoxy,b}$**	4.7
			**$TS^{Ethoxy,b}$**	27.5
			**$I^{Oxy,c}$**	2.1
			**$TS^{Oxy,c}$**	**26.8**
$Tp^{(CF_3)_2,Br}Ag$	Ph	CO_2Et	**$I^{Ethoxy,b}$**	5.4
			**$TS^{Ethoxy,b}$**	**28.3**
			**$I^{Oxy,c}$**	6.3
			**$TS^{Oxy,c}$**	28.5
$Tp^{F27}Ag$	Ph	CO_2Et	**$I^{Ethoxy,b}$**	5.8
			**$TS^{Ethoxy,b}$**	**26.8**
			**$I^{Oxy,c}$**	6.3
			**$TS^{Oxy,c}$**	27.7
$Rh_2(OAc)_4$	Ph	CO_2Et	**$I^{Ethoxy,b}$**	7.3
			**$TS^{Ethoxy,b}$**	**32.5**
			**$I^{Oxy,c}$**	7.2
			**$TS^{Oxy,c}$**	33.9
$Tp^{Br_3}Ag$	p-C_6H_4-CF_3	$CO_2CH_2CF_3$	**$I^{Ethoxy,b}$**	7.2
			**$TS^{Ethoxy,b}$**	**24.3**
			**$I^{Oxy,c}$**	4.2
			**$TS^{Oxy,c}$**	24.3
$Tp^{(CF_3)_2,Br}Ag$	p-C_6H_4-CF_3	$CO_2CH_2CF_3$	**$I^{Ethoxy,b}$**	4.8
			**$TS^{Ethoxy,b}$**	**22.0**
			**$I^{Oxy,c}$**	7.5
			**$TS^{Oxy,c}$**	23.7
$Tp^{F27}Ag$	p-C_6H_4-CF_3	$CO_2CH_2CF_3$	**$I^{Ethoxy,b}$**	9.7
			**$TS^{Ethoxy,b}$**	**22.8**
			**$I^{Oxy,c}$**	7.8
			**$TS^{Oxy,c}$**	24.1
$Rh_2(OAc)_4$	p-C_6H_4-CF_3	$CO_2CH_2CF_3$	**$I^{Ethoxy,b}$**	3.7
			**$TS^{Ethoxy,b}$**	**26.3**
			**$I^{Oxy,c}$**	5.4
			**$TS^{Oxy,c}$**	27.8
$Tp^{Br_3}Ag$	H	CO_2Et	**$I^{Ethoxy,b}$**	7.1
			**$TS^{Ethoxy,b}$**	**10.7**
			**$I^{Oxy,c}$**	3.0
			**$TS^{Oxy,c}$**	11.3
$Tp^{(CF_3)_2,Br}Ag$	H	CO_2Et	**$I^{Ethoxy,b}$**	6.6
			**$TS^{Ethoxy,b}$**	**9.1**
			**$I^{Oxy,c}$**	6.2
			**$TS^{Oxy,c}$**	10.1
$Tp^{F27}Ag$	H	CO_2Et	**$I^{Ethoxy,b}$**	6.0
			**$TS^{Ethoxy,b}$**	**8.1**
			**$I^{Oxy,c}$**	6.6
			**$TS^{Oxy,c}$**	11.4
$Rh_2(OAc)_4$	H	CO_2Et	**$I^{Ethoxy,b}$**	5.4
			**$TS^{Ethoxy,b}$**	**15.7**
			**$I^{Oxy,c}$**	6.2
			**$TS^{Oxy,c}$**	17.3
$Tp^{Br_3}Ag$	H	CF_3	**I**	5.7
			**TS**	**6.6**
$Tp^{(CF_3)_2,Br}Ag$	H	CF_3	**TS**	**Barrierless**
$Tp^{F27}Ag$	H	CF_3	**TS**	**Barrierless**
$Rh_2(OAc)_4$	H	CF_3	**I**	5.1
			**TS**	**15.3**

[a]Continuum solvation model for n-Hexane. [b]C−H activation with the methane incoming from the ester face containing the methoxy group. [c]C−H activation with the methane incoming from the ester face containing the carbonyl oxygen.

Table SC2. B3LYP-D3 characterized transition states for 1ry and 2ry C−H activation over the $Tp^{(CF_3)_2,Br}AgC(H)CO_2Et$ carbene.

**Species**	**Orientation**	**ΔG_{sol} (Kcal mol^{-1})[a]**
$TS'_{Oxy}A$	Oxy face[b]	5.9
$TS'_{Oxy}B$	Oxy face[b]	9.8
$TS'_{Ethoxy}A$	Ethoxy face[c]	**3.7**
$TS'_{Ethoxy}B$	Ethoxy face[c]	**3.7**
1ry product		-56.6
$TS''_{Oxy}A$	Oxy face[b]	3.1
$TS''_{Oxy}B$	Oxy face[b]	**2.7**
$TS''_{EthOxy}A$	Ethoxy face[c]	3.8
$TS''_{EthOxy}B$	Ethoxy face[c]	**2.7**
2ry product		-56.3

[a]Continuum solvation model for n-Hexane. [b]C−H activation with the propane incoming from the ester face containing the carbonyl oxygen. [c]C−H activation with the propane incoming from the ester face containing the methoxy group.

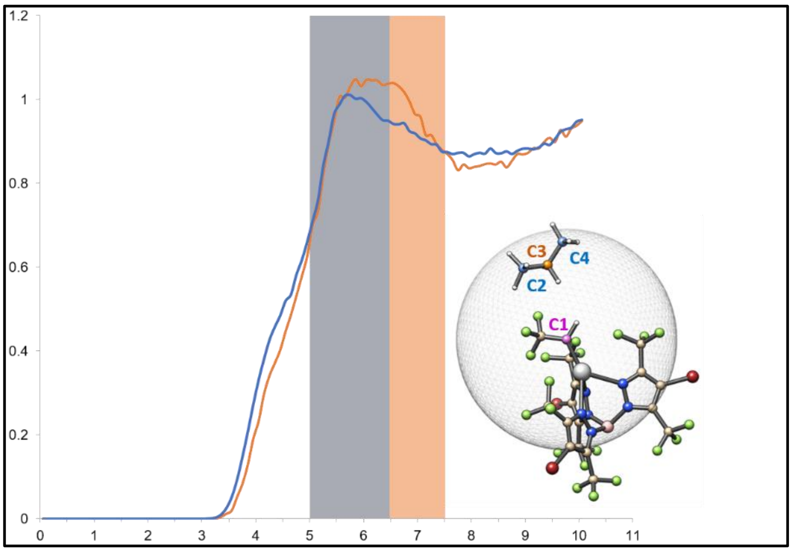

Figure SC1. Radial pair distribution function between C1-C2/C4 (blue) and C1-C3 (orange) along the whole trajectory.

We also computed the complete Gibbs energy profile of the methane C−H bond functionalization by carbene insertion from the fluorinated diazo N_2=CHCF$_3$, using both Tp$^{(CF_3)_2,Br}$Ag(THF) and Tp^{F27}Ag(THF) as catalyst precursors. The results are shown in Figures SC2 and SC3.

In line with our previous reported studies,[15] the first step of the reaction is the catalyst activation by replacing the THF ligand by the diazocompound. This leads to intermediate I, 5.7 and 5.0 kcal·mol^{-1} below the starting complex for Tp$^{(CF_3)_2,Br}$Ag(THF) and Tp^{F27}Ag(THF), respectively. The second step is the N_2 extrusion entailing the formation of the active carbene species. This is the highest barrier in the whole process. The overall reaction barriers ($\Delta G^{\neq}$) barriers for the reaction results in 20.8 and 20.5 kcal·mol^{-1}. These values are lower than those previously computed for the activation of ethyl diazoacetate by Tp$^{(CF_3)_2,Br}$Ag(THF) and Tp^{F27}Ag(THF). These results demonstrate that the rate-limiting step of the catalytic cycle remains in the nitrogen extrusion step, and that this is completely feasible, even with a lower barrier than with other diazocompounds.

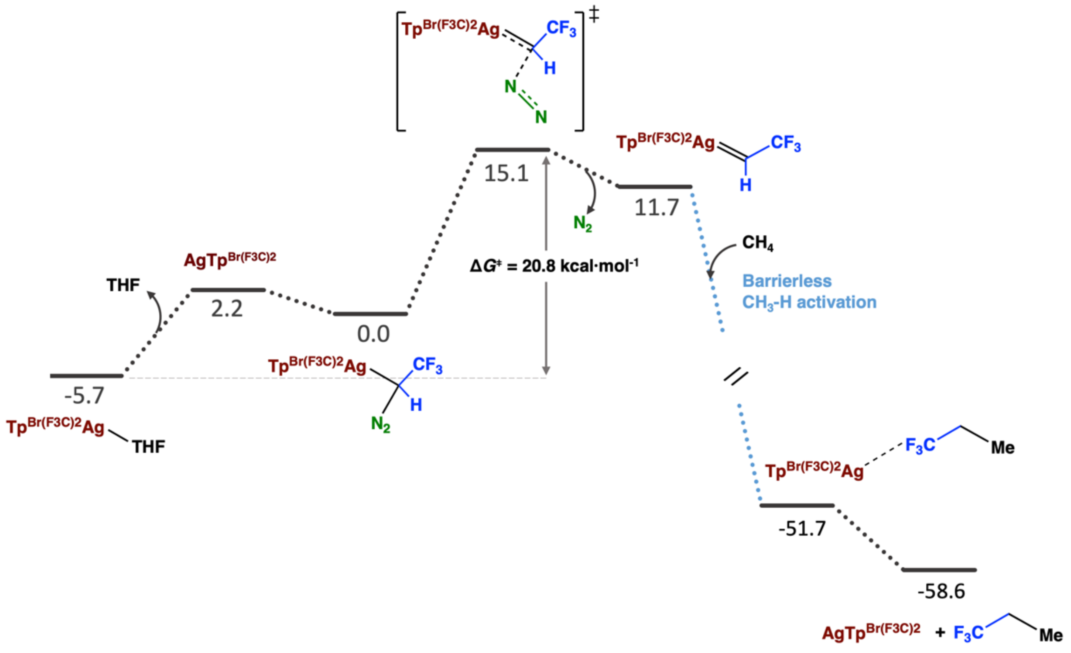

Figure SC2. Complete B3LYP-D3 (continuum solvation model for n-hexane) computed Gibbs energy profile for the Tp$^{(CF_3)_2,Br}$Ag catalyzed methane C−H activation by carbene insertion from the fluorinated diazo N_2=CHCF3. Values of calculated ΔG are given in kcal mol^{-1}.

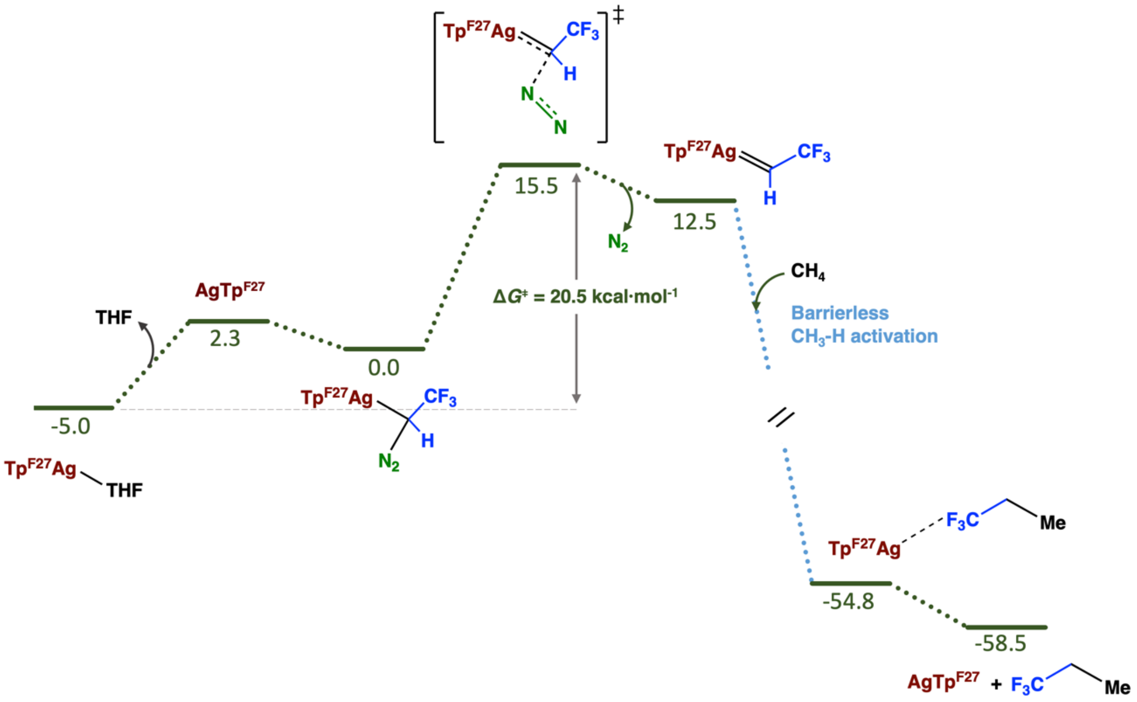

Figure SC3. Complete B3LYP-D3 (continuum solvation model for n-hexane) computed Gibbs energy profile for the Tp^{F27}Ag catalyzed methane C−H activation by carbene insertion from the fluorinated diazo N_2=CHCF$_3$. Values of calculated Δ*G* are given in kcal mol^{-1}.

9. References for experimental section

(1) Gava, R.; Olmos, A.; Noverges, B.; Varea, T.; Álvarez, E.; Belderrain, T. R.; Caballero, A.; Asensio, G.; Perez, P. J. Discovering Copper for Methane C–H Bond Functionalization. *ACS Catal.* **2015**, *5*, 3726–3730.

(2) Wang, S.; Yang, L.-J.; Zeng, J.-L.; Zheng, Y.; Ma, J.-A. Silver-catalyzed [3 + 2] cycloaddition of isocyanides with diazo compounds: new regioselective access to 1,4-disubstituted-1,2,3-triazoles. *Org. Chem. Front.* **2015**, *2*, 1468–1474.

(3) (a) Wu, L.-L.; Yang, C.-L.; Lo, F.-C.; Chiang, C.-H.; Chang, C.-W.; Ng, K. Y.; Chou, H.-H.; Hung, H.-Y.; Chan, S. I.; Yu, S. S.-F. Tuning the Regio- and Stereoselectivity of C–H Activation in n-Octanes by Cytochrome P450 BM-3 with Fluorine Substituents: Evidence for Interactions Between a C–F Bond and Aromatic π Systems. *Chem. Eur. J.* **2011**, *17*, 4774–4787. (b) Choi, G.; Lee, G. S.; Park, B.; Kim, D.; Hong, S. H. Direct C(sp^3)–H Trifluoromethylation of Unactivated Alkanes Enabled by Multifunctional Trifluoromethyl Copper Complexes. *Angew. Chem. Int. Ed.* **2021**, *60*, 5467–5474.

(4) Elleman, D. D.; Brown, L. C.; Williams, D. The nuclear magnetic resonance spectra of fluorocarbons: Part II. Halogenated n-paraffins. *Journal of Molecular Spectroscopy* **1961**, *1*, 322–340.

(5) Linclau, B.; Wang, Z.; Compain, G.; Paumelle, V.; Fontenelle, C. Q.; Wells, N.; Weymouth-Wilson, A. Investigating the Influence of (Deoxy)fluorination on the Lipophilicity of Non-UV-Active Fluorinated Alkanols and Carbohydrates by a New log *P* Determination Method. *Angew. Chem. Int. Ed.* **2016**, *55*, 674–678.

(6) Park, H. S.; Lee, H. Y.; Kim, Y. H. Facile Barton-McCombie Deoxygenation of Alcohols with Tetrabutylammonium Peroxydisulfate and Formate Ion. *Org. Lett.* **2005**, *7*, 3187–3190.

(7) Xia, A. ; Xie, X. ; Hu, X. ; Xu, W. ; Liu, Y. Dehalogenative Deuteration of Unactivated Alkyl Halides Using D_2O as the Deuterium Source. *J. Org. Chem.* **2019**, *84*, 13841–13857.

(8) Jones, K. D.; Rixson J. E.; Skelton, B. W.; Gericke, K. M.; Stewart, S. G. The Total Synthesis of Heraclemycin B through β-Ketosulfoxide and Aldehyde Annulation. *Asian J. Org. Chem.* **2015**, *4*, 936–942.

(9) Su, Y.-L.; Li, L.-L.; Zhou, X.-L.; Dai, Z.-Y.; Wang, P.-S.; Gong, L.-Z. Asymmetric α-allylation of aldehydes with alkynes by integrating chiral hydridopalladium and enamine catalysis. *Org. Lett.* **2018**, *20*, 2403–2406.

10. References for computational section

(1) R.D. Gaussian 09; Frisch, M. J.; Trucks, G. W.; Schlegel, H. B.; Scuseria, G. E.; Robb, M. A.; Cheeseman, J. R.; Scalmani, G.; Barone, V.; Petersson, G. A.; Nakatsuji, H.; Li, X.; Caricato, M.; Marenich, A.; Bloino, J.; Janesko, B. G.; Gomperts, R.; Mennucci, B.; Hratchian, H. P.; Ortiz, J. V.; Izmaylov, A. F.; Sonnenberg, J. L.; Williams-Young, D.; Ding, F.; Lipparini, F.; Egidi, F.; Goings, J.; Peng, B.; Petrone, A.; Henderson, T.; Ranasinghe, D.; Zakrzewski, V. G.; Gao, J.; Rega, N.; Zheng, G.; Liang, W.; Hada, M.; Ehara, M.; Toyota, K.; Fukuda, R.; Hasegawa, J.; Ishida, M.; Nakajima, T.; Honda, Y.; Kitao, O.; Nakai, H.; Vreven, T.; Throssell, K.; Montgomery Jr., J. A.; Peralta, J. E.; Ogliaro, F.; Bearpark, M.; Heyd, J. J.; Brothers, E.; Kudin, K. N.; Staroverov, V. N.; Keith, T.; Kobayashi, R.; Normand, J.; Raghavachari, K.; Rendell, A.; Burant, J. C.;

Iyengar, S. S.; Tomasi, J.; Cossi, M.; Millam, J. M.; Klene, M.; Adamo, C.; Cammi, R.; Ochterski, J. W.; Martin, R. L.; Morokuma, K.; Farkas, O.; Foresman, J. B.; Fox, D. J., *Gaussian, Inc.*, Wallingford CT, **2016**.

(2) Yanai, T.; Tew, D. P.; Handy, N. C. A new hybrid exchange-correlation functional using the Coulomb-attenuating method (CAM-B3LYP). *Chem. Phys. Lett.* **2004**, *393*, 51–57.

(3) Grimme, S.; Antony, J.; Ehrlich, S.; Krieg, H. A consistent and accurate ab initio parametrization of density functional dispersion correction (DFT-D) for the 94 elements H-Pu. *J. Phys. Chem.* **2010**, 132, 154104–19.

(4) Marenich, A. V.; Cramer, C. J.; Truhlar, D. G. Universal Solvation Model Based on Solute Electron Density and on a Continuum Model of the Solvent Defined by the Bulk Dielectric Constant and Atomic Surface Tensions. *J. Phys. Chem. B* **2009**, *113*, 6378–6396.

(5) Franci, M. M.; Pietro, W. J.; Hehre, W. J.; Binkley, J. S.; Gordon, M. S.; DeFrees, D. J.; Pople, J. A. Self-consistent molecular orbital methods. XXIII. A polarization-type basis set for second-row elements. *J. Phys. Chem.* **1982**, *77*, 3654–3665.

(6) Hay, P. J.; Wadt, W. R. Ab initio effective core potentials for molecular calculations. Potentials for the transition metal atoms Sc to Hg. *J. Phys. Chem.* **1985**, *82*, 270–283.

(7) Rodríguez, M. R.; Beltrán, Á.; Mudarra, Á. L.; Álvarez, E.; Maseras, F.; Díaz-Requejo, M. M.; Pérez, P. J. Catalytic Nitrene Transfer To Alkynes: A Novel and Versatile Route for the Synthesis of Sulfinamides and Isothiazoles. *Angew. Chem. Int. Ed.* **2017**, *56*, 12842–12847.

(8) Weigend, F. Accurate Coulomb-fitting basis sets for H to Rn. *Phys. Chem. Chem. Phys.* **2006**, *8*, 1057–1065.

(9) Weigend, F.; Ahlrichs, R. Balanced basis sets of split valence, triple zeta valence and quadruple zeta valence quality for H to Rn: Design and assessment of accuracy. *Phys. Chem. Chem. Phys.* **2005**, *7*, 3297–3305.

(10) Bryantsev, V. S.; Diallo, M. S.; Goddard, W.A. Calculation of solvation free energies of charged solutes using mixed cluster/continuum models. *J. Phys. Chem. B* **2008**, *112*, 9709–9719.

(11) Eastman, P.; Swails, J.; Chodera, J. D.; McGibbon, R. T.; Zhao, Y.; Beauchamp, K. A.; Wang, L.-P.; Simmonett, A. C.; Harrigan, M. P.; Stern, C. D.; Wiewiora, R. P.; Brooks, B. R.; Pande, V. S. OpenMM 7: Rapid development of high performance algorithms for molecular dynamics. *PLoS Comput. Biol.* **2017**, *13*, e1005659.

(12) Rodríguez-Guerra, J.; Alonso-Cotchico, L.; Velasco-Carneros, L.; Maréchal, J.-D. OMMProtocol: A Command Line Application to Launch Molecular Dynamics Simulations with OpenMM. *Preprint from ChemRxi*, **2018**.

(13) Li, P.; Merz, K. M. MCPB.py: A Python Based Metal Center Parameter Builder. *J. Chem. Inf. Model.* **2016**, *56*, 599–604.

(14) Case, D. A.; Betz, R. M.; Cerutti, D. S.; CheathamIII, T. E.; Darden, T. A.; Duke, R. E.; Giese, T. J.; Gohlke, H.; Goetz, A. W.; Homeyer, N.; Izadi, S.; Janowski, P.; Kaus, J.; Kovalenko, A.; Lee, T. S.; LeGrand, S.; Li, P.; Lin, C.; Luchko, T.; Luo, R.; Madej, B.; Mermelstein, D.; Merz, K. M.; Monard, G.; Nguyen, H.; Nguyen, H. T.; Omelyan, I.; Onufriev, A.; Roe, D. R.; Roitberg, A.; Sagui, C.; Simmerling, C. L.; Botello-Smith, W. M.; Swails, J.; Walker, R. C.; Wang, J.; Wolf, R. M.; Wu, X.; Xiao, L.; Kollman, P. A., *AMBER 2016*, University of California-San Francisco, **2016**.

(15) Olmos, A.; Gava, R.; Noverges, B.; Bellezza, D.; Jacbo, K.; Besora, M.; Sameera, W. M. C.; Etienne, M.; Maseras, F.; Asensio, G.; Caballero, A.; Pérez, P. J. *Angew. Chem. Int. Ed.* **2018**, *57*, 13848-13852.

11. Cartesian coordinates

00-TpBrCF3_2Ag_CHCO2Et

Charge	0
Electronic Energy, BS1 (a.u.)	-3214.563176
Thermal and entropic correction, BS1 (a.u.)	0.199299
Electronic Energy, BS2 (a.u.)	-10900.184682
Number of Imaginary Frequencies	0
Imaginary frequencies (cm-1)	None

Molecular Geometry in Cartesian Coordinates

Ag	5.612582	-1.032967	9.960475
Br	7.388619	1.988906	4.837494
Br	-0.664604	-0.378346	9.227408
Br	6.599175	3.316171	14.342364
F	6.589488	-1.158175	5.132230
F	5.942075	-2.035939	7.016789
F	7.942118	-1.200408	6.835265
F	6.373567	4.505802	6.306847
F	5.980659	4.464857	8.445692
F	4.330702	4.349464	7.030791
F	0.886222	-2.234431	11.456076
F	3.058708	-2.309938	11.576793
F	2.041181	-2.964818	9.766250
F	0.072231	2.370829	8.014801
F	2.114881	2.756031	7.373053
F	1.381232	3.599665	9.240675
F	7.087589	-0.307984	13.684094
F	8.689439	1.068593	13.167614
F	8.051843	-0.443030	11.736998
F	4.327105	4.870003	12.935192
F	2.978064	3.868612	11.553935
F	4.609597	5.091389	10.791316
C	6.159329	-3.026774	10.227467
N	5.398580	0.398217	8.024285
N	5.136577	1.701181	8.224958
N	3.274849	-0.006930	10.233624
N	3.087860	1.170073	9.614695
N	6.021892	1.134815	10.944348
N	5.146642	2.131660	10.721361
C	6.138096	0.309351	6.919989
C	6.381263	1.590090	6.386983
C	5.721307	2.455507	7.255619
C	6.652184	-1.024554	6.467609
C	5.599391	3.953610	7.253676
C	2.111784	-0.652018	10.201619
C	1.134541	0.115814	9.536292
C	1.803407	1.281482	9.175686
C	2.016061	-2.038335	10.755463
C	1.334306	2.508731	8.449427
C	6.607029	1.379041	12.115417
C	6.093970	2.560703	12.684727
C	5.158510	3.011830	11.758298
C	7.615214	0.421983	12.679247
C	4.266590	4.221140	11.762260
O	5.730078	-3.915561	12.070967
C	5.345061	-3.996509	10.900524
B	4.272720	2.159223	9.436446
H	3.866828	3.249583	9.245878

H	7.149231	-3.424877	9.980935
O	4.388161	-4.712186	10.386175
C	3.558044	-5.481527	11.332882
H	3.157179	-4.774852	12.062792
H	4.206365	-6.196908	11.845708
C	2.474937	-6.153534	10.521477
H	1.840353	-6.743187	11.190356
H	1.855021	-5.408161	10.018750
H	2.904484	-6.824619	9.772289

01-TpBrCF3_2Ag_CHCF3

Charge	0
Electronic Energy, BS1 (a.u.)	-3284.372241
Thermal and entropic correction, BS1 (a.u.)	0.134585
Electronic Energy, BS2 (a.u.)	
Number of Imaginary Frequencies	0
Imaginary frequencies (cm-1)	None

Molecular Geometry in Cartesian Coordinates

Ag	5.588064	-0.938535	10.240832
Br	7.398195	1.952582	4.841810
Br	-0.570504	-0.524770	9.403637
Br	6.821436	3.597616	14.178490
F	6.565167	-1.239842	5.246341
F	6.076405	-2.019274	7.221852
F	8.025395	-1.124025	6.852852
F	6.256051	4.497038	6.187826
F	5.953143	4.517593	8.342178
F	4.249937	4.306966	7.003992
F	1.111540	-2.662722	10.999716
F	3.082691	-2.253726	11.833549
F	2.899076	-2.978356	9.794728
F	-0.002687	2.283337	8.288381
F	1.982647	2.762638	7.541746
F	1.337060	3.509967	9.481870
F	7.023105	-0.465668	13.316687
F	8.570149	1.062238	13.457057
F	8.345752	-0.104669	11.631435
F	4.522921	5.087493	12.794849
F	3.084012	4.002409	11.578112
F	4.656428	5.170416	10.628224
C	6.191393	-2.866615	10.278050
N	5.415796	0.421260	8.060685
N	5.126790	1.723643	8.221306
N	3.400958	-0.050903	10.217582
N	3.140594	1.143427	9.663182
N	6.050150	1.211301	10.959622
N	5.185491	2.209604	10.710101
C	6.168379	0.318005	6.970841
C	6.388395	1.587637	6.397773
C	5.701668	2.464207	7.232091
C	6.708998	-1.018826	6.564490
C	5.539215	3.955417	7.183812
C	2.268152	-0.752995	10.220351
C	1.228902	0.002934	9.644361
C	1.832309	1.212955	9.301826
C	2.322740	-2.167846	10.719296
C	1.277208	2.450137	8.651162
C	6.699929	1.510123	12.085273
C	6.239277	2.736357	12.599755
C	5.268670	3.150300	11.687467
C	7.673093	0.506035	12.633016

C	4.382011	4.366394	11.672944
B	4.280158	2.182224	9.438831
H	3.828283	3.253178	9.248112
H	6.564660	-3.453659	9.431473
C	5.973439	-3.797184	11.437708
F	5.634314	-3.174599	12.574260
F	4.964024	-4.628135	11.094121
F	7.089052	-4.515608	11.677745

02-Propane

Charge	0
Electronic Energy, BS1 (a.u.)	-119.160637
Thermal and entropic correction, BS1 (a.u.)	0.078181
Electronic Energy, BS2 (a.u.)	-119.199844
Number of Imaginary Frequencies	0
Imaginary frequencies (cm-1)	None

Molecular Geometry in Cartesian Coordinates

C	3.684150	-3.416986	7.923234
H	2.847300	-3.775448	8.534808
H	3.608313	-2.324091	7.871393
C	3.650425	-4.038821	6.524252
H	3.759311	-5.128369	6.605493
H	4.515881	-3.687797	5.946878
H	4.611891	-3.661437	8.452456
C	2.362517	-3.710386	5.763378
H	2.355537	-4.163250	4.765682
H	1.480994	-4.078074	6.302366
H	2.243124	-2.627370	5.638525

03-TpBrCF3_2Ag

Charge	0
Electronic Energy, BS1 (a.u.)	-2908.132174
Thermal and entropic correction, BS1 (a.u.)	0.114634
Electronic Energy, BS2 (a.u.)	-10593.631237
Number of Imaginary Frequencies	0
Imaginary frequencies (cm-1)	None

Molecular Geometry in Cartesian Coordinates

Ag	5.621266	-0.986363	10.030139
Br	7.443919	2.049711	4.830018
Br	-0.617877	-0.447036	9.427815
Br	6.681405	3.522961	14.270784
F	6.376475	-1.352199	5.332714
F	6.651646	-1.935934	7.413346
F	8.234399	-0.878259	6.353865
F	6.258465	4.528145	6.236233
F	5.921790	4.510863	8.384950
F	4.245826	4.278029	7.015745
F	1.401002	-2.198070	11.856437
F	3.363101	-2.636816	11.018239
F	1.564835	-2.995683	9.842399
F	0.048477	2.303245	8.203550
F	2.050250	2.715842	7.460492
F	1.404832	3.534674	9.371428

F	7.352541	0.156821	13.960969
F	8.899323	1.179107	12.828611
F	7.867966	-0.531770	11.959332
F	4.361299	4.973886	12.842393
F	2.989498	3.865361	11.569630
F	4.546654	5.100339	10.681278
N	5.557731	0.406176	8.044383
N	5.201459	1.691516	8.222040
N	3.371755	-0.107694	10.176267
N	3.155845	1.097407	9.617405
N	6.086295	1.188133	10.986740
N	5.191909	2.158937	10.723373
C	6.325313	0.352596	6.960883
C	6.475287	1.634727	6.398746
C	5.744697	2.467126	7.241681
C	6.899605	-0.954896	6.506555
C	5.538842	3.955900	7.213389
C	2.213964	-0.760406	10.174054
C	1.200199	0.035964	9.608113
C	1.846847	1.217278	9.256914
C	2.128138	-2.151356	10.725617
C	1.329640	2.452177	8.573104
C	6.664372	1.484532	12.146288
C	6.144949	2.686381	12.663534
C	5.198777	3.084472	11.723707
C	7.702399	0.574387	12.729472
C	4.272560	4.268265	11.704668
B	4.317251	2.122209	9.432046
H	3.868225	3.192589	9.230699

04-CO2EtProdPrim

Charge	0
Electronic Energy, BS1 (a.u.)	-425.686594
Thermal and entropic correction, BS1 (a.u.)	0.166663
Electronic Energy, BS2 (a.u.)	-425.847282
Number of Imaginary Frequencies	0
Imaginary frequencies (cm-1)	None

Molecular Geometry in Cartesian Coordinates

C	5.132817	-2.658722	12.337974
O	4.794117	-5.018346	11.855390
C	4.857799	-3.869118	11.466483
H	5.770032	-1.964549	11.778524
O	4.658380	-3.506880	10.182672
C	4.340918	-4.578399	9.261229
H	3.432770	-5.085675	9.604215
H	5.153821	-5.312135	9.276986
C	4.162177	-3.956397	7.891110
H	3.921759	-4.733744	7.158675
H	3.348394	-3.224793	7.897784
H	5.078025	-3.450825	7.569971
H	4.170844	-2.145610	12.470051
C	5.745219	-3.022795	13.693001
H	5.731565	-2.133260	14.335398
C	7.179378	-3.556743	13.589418
H	7.811220	-2.803179	13.098587
H	7.183626	-4.440948	12.941230
C	7.773989	-3.917726	14.952710
H	8.798407	-4.293744	14.856837
H	7.179697	-4.694690	15.447665
H	7.800260	-3.046851	15.618920
H	5.111691	-3.775773	14.175922

05-CO2EtProdSec

Charge	0
Electronic Energy, BS1 (a.u.)	-425.687633
Thermal and entropic correction, BS1 (a.u.)	0.167302
Electronic Energy, BS2 (a.u.)	-425.847536
Number of Imaginary Frequencies	0
Imaginary frequencies (cm-1)	None

Molecular Geometry in Cartesian Coordinates

```
C          7.214328       -3.192675       12.453557
O          5.720623       -5.091111       12.198464
C          6.132690       -4.022472       11.791873
H          8.136280       -3.379216       11.885236
O          5.696406       -3.430837       10.659261
C          4.708252       -4.157048        9.885060
H          4.220849       -3.388601        9.279842
H          3.974218       -4.596927       10.565954
C          5.361321       -5.220682        9.018002
H          4.606052       -5.705348        8.389928
H          6.119502       -4.776730        8.365349
H          5.832903       -5.984503        9.641323
H          5.298160       -3.660671       14.416805
C          6.193733       -3.142336       14.771615
H          6.008774       -2.061657       14.720209
C          7.423825       -3.526303       13.938260
H          6.982393       -2.130603       12.313010
H          7.558348       -4.612489       14.011555
C          8.686441       -2.834370       14.466277
H          9.577638       -3.134848       13.903241
H          8.857413       -3.081272       15.519756
H          8.597895       -1.743105       14.392624
H          6.337141       -3.404630       15.825528
```

06-TpBrCF3_2Ag_CHCO2Et-C4H8

Charge	0
Electronic Energy, BS1 (a.u.)	-3333.739318
Thermal and entropic correction, BS1 (a.u.)	0.298640
Electronic Energy, BS2 (a.u.)	-11019.393068
Number of Imaginary Frequencies	0
Imaginary frequencies (cm-1)	None

Molecular Geometry in Cartesian Coordinates

```
Ag         5.706231       -1.157267        9.664162
Br         8.018871        1.973919        4.881847
Br        -0.616733       -0.451868        8.490326
Br         6.284950        3.259944       14.087413
F          7.553661       -1.178916        5.151755
F          6.219345       -2.103415        6.605717
F          8.168324       -1.380987        7.232119
F          6.743066        4.442537        6.213677
F          6.119616        4.355298        8.295553
F          4.636701        4.233558        6.706770
F          1.097190       -2.079303       11.277124
F          3.061768       -2.712682       10.594114
```

F	1.313128	-3.032217	9.336808
F	0.281674	2.245744	7.261185
F	2.374744	2.648921	6.826653
F	1.460176	3.455678	8.629596
F	7.080324	-0.021695	13.798294
F	8.638186	1.061401	12.740869
F	7.745337	-0.706733	11.839471
F	4.023095	4.686318	12.545289
F	2.820367	3.657089	11.054628
F	4.471506	4.933369	10.433627
C	6.228320	-3.156825	9.421692
N	5.635568	0.292938	7.738169
N	5.331589	1.585460	7.923936
N	3.279592	-0.199195	9.681322
N	3.150392	0.993526	9.075625
N	5.942691	0.976617	10.727569
N	5.070293	1.956826	10.419222
C	6.522826	0.228362	6.746744
C	6.818567	1.520944	6.271113
C	6.030844	2.363514	7.054533
C	7.118516	-1.107155	6.416537
C	5.881867	3.858836	7.060691
C	2.110024	-0.822268	9.561516
C	1.185723	-0.018336	8.863544
C	1.896881	1.138017	8.561517
C	1.893061	-2.160871	10.193778
C	1.495391	2.379380	7.818353
C	6.442529	1.260734	11.929446
C	5.878842	2.449173	12.429754
C	4.998732	2.860054	11.434194
C	7.479392	0.392912	12.578496
C	4.076549	4.045471	11.367450
O	5.487595	-4.743372	10.623435
C	5.284724	-4.233544	9.519001
B	4.333948	1.996400	9.045984
H	3.935694	3.086772	8.841722
H	7.229348	-3.503797	9.147281
O	4.362352	-4.504738	8.636278
C	3.365368	-5.523320	9.008370
H	2.855654	-5.168777	9.906321
H	3.896173	-6.450704	9.240143
C	2.429634	-5.665637	7.830553
H	1.658935	-6.404568	8.070786
H	1.940228	-4.713705	7.612382
H	2.967499	-6.002548	6.939856
H	5.881752	-3.649543	14.504066
C	5.268483	-2.744183	14.574224
H	4.395427	-2.979288	15.195218
C	4.845642	-2.245936	13.190381
H	4.317887	-3.041625	12.654497
H	5.745214	-2.031356	12.602030
C	3.962911	-0.995706	13.256599
H	3.721762	-0.614531	12.259397
H	4.466587	-0.193218	13.806904
H	3.017551	-1.207520	13.770104
H	5.855044	-1.982592	15.100420

07-TSPrim-OxyA

Charge	0
Electronic Energy, BS1 (a.u.)	-3333.730310
Thermal and entropic correction, BS1 (a.u.)	0.299384
Electronic Energy, BS2 (a.u.)	-11019.384343
Number of Imaginary Frequencies	2

Imaginary frequencies (cm-1) -99.0i, -8.3i

Molecular Geometry in Cartesian Coordinates

Ag	5.374329	-1.050307	10.115987
Br	8.448608	1.273255	5.284919
Br	-0.662922	-0.472155	8.033910
Br	5.460876	3.905899	13.919205
F	7.887540	-1.779875	5.998766
F	6.284047	-2.458772	7.308569
F	8.125532	-1.690586	8.162671
F	7.068183	3.929588	6.001337
F	6.186997	4.188793	7.972768
F	4.911691	3.823911	6.247485
F	0.550114	-1.600096	11.245614
F	2.594301	-2.318410	11.044278
F	1.084574	-2.876732	9.572888
F	0.485518	1.960710	6.488947
F	2.631137	2.265542	6.296067
F	1.500838	3.382619	7.783549
F	5.997698	0.138285	13.888467
F	7.658506	1.517082	13.630239
F	7.406272	-0.149329	12.252622
F	3.572550	5.190312	11.841927
F	2.550153	3.988902	10.344780
F	4.340031	5.107102	9.810330
C	5.776998	-3.085607	10.197430
N	5.647001	0.093219	8.010344
N	5.360080	1.401332	7.944684
N	3.031726	-0.031161	9.710973
N	3.030156	1.030412	8.885575
N	5.566051	1.213916	10.855493
N	4.792736	2.160719	10.294100
C	6.658251	-0.138404	7.175083
C	7.055093	1.057835	6.545595
C	6.193949	2.022070	7.066981
C	7.243707	-1.518541	7.143725
C	6.090482	3.499774	6.813405
C	1.870282	-0.655950	9.539902
C	1.081251	0.011715	8.580707
C	1.866362	1.087075	8.179720
C	1.526286	-1.863892	10.354920
C	1.613289	2.180341	7.182901
C	5.894898	1.643360	12.073248
C	5.306422	2.896515	12.328522
C	4.605948	3.191113	11.161485
C	6.745402	0.790791	12.965967
C	3.765105	4.380767	10.789438
O	4.484247	-4.940199	10.903821
C	4.734123	-4.120320	10.041527
B	4.240748	2.000382	8.845341
H	3.909974	3.050505	8.424233
H	6.774929	-3.483755	9.977634
O	4.120245	-3.980850	8.867486
C	3.079507	-4.958442	8.544968
H	2.351614	-4.959742	9.358105
H	3.545201	-5.946984	8.488329
C	2.471570	-4.528308	7.228858
H	1.685939	-5.234624	6.942247
H	2.025380	-3.534974	7.320680
H	3.225149	-4.506556	6.436204
H	6.304500	-3.519181	12.004443
C	6.429906	-3.228497	13.071062
H	5.863831	-2.304091	13.209740
C	7.906905	-3.052460	13.414339
H	8.363372	-2.342575	12.715344
H	8.432959	-4.006853	13.287988
C	8.093525	-2.539007	14.848289

H	9.155257	-2.418960	15.092018
H	7.657765	-3.232193	15.577910
H	7.603796	-1.568131	14.978329
H	5.949859	-4.029436	13.643797

08-TSPrim-OxyB

Charge	0
Electronic Energy, BS1 (a.u.)	-3333.733041
Thermal and entropic correction, BS1 (a.u.)	0.298679
Electronic Energy, BS2 (a.u.)	-11019.387117
Number of Imaginary Frequencies	1
Imaginary frequencies (cm-1)	-120.3i

Molecular Geometry in Cartesian Coordinates

Ag	5.457995	-1.034869	10.060123
Br	8.357289	1.334082	5.158503
Br	-0.636849	-0.501912	8.150524
Br	5.633220	3.925022	13.889974
F	7.836348	-1.726194	5.863173
F	6.280703	-2.425213	7.219364
F	8.146549	-1.655899	8.018941
F	6.994519	3.978348	5.950126
F	6.170960	4.214885	7.949208
F	4.846712	3.860099	6.258447
F	0.680887	-1.626195	11.348351
F	2.711681	-2.349812	11.055517
F	1.139837	-2.891637	9.644787
F	0.451987	1.952575	6.598561
F	2.590018	2.270856	6.352022
F	1.491754	3.374264	7.873322
F	6.253290	0.431171	14.038444
F	7.967979	1.565280	13.332885
F	7.367925	-0.251081	12.293991
F	3.657445	5.188370	11.877837
F	2.600169	3.982155	10.409418
F	4.363849	5.115808	9.823836
C	5.911273	-3.059270	10.154953
N	5.656532	0.117759	7.967592
N	5.361036	1.424781	7.923015
N	3.092116	-0.038914	9.744326
N	3.061655	1.028141	8.926173
N	5.647433	1.224139	10.835742
N	4.853997	2.167531	10.293454
C	6.639832	-0.102283	7.095806
C	7.008951	1.101210	6.463917
C	6.161257	2.057027	7.023182
C	7.230075	-1.479239	7.031809
C	6.043360	3.536621	6.786617
C	1.932456	-0.673288	9.596252
C	1.115201	-0.006145	8.660696
C	1.881127	1.079062	8.248740
C	1.617112	-1.886274	10.414980
C	1.595690	2.175617	7.264165
C	6.004040	1.658113	12.043755
C	5.419604	2.910765	12.309875
C	4.685837	3.199564	11.163424
C	6.903006	0.848397	12.929788
C	3.824672	4.382847	10.817805
O	4.678759	-4.908148	10.956168
C	4.879521	-4.109947	10.059942
B	4.263329	2.008366	8.860743
H	3.912348	3.057683	8.454330

H	6.908906	-3.452425	9.924349
O	4.213145	-4.010296	8.911908
C	3.160602	-4.997982	8.667738
H	2.459791	-4.959380	9.503673
H	3.621162	-5.990222	8.640323
C	2.510196	-4.623834	7.354513
H	1.713468	-5.338922	7.126077
H	2.070074	-3.625615	7.416099
H	3.237200	-4.641126	6.537326
H	6.467192	-3.248988	11.998241
C	6.345384	-3.158070	13.101770
H	6.433477	-4.174816	13.497423
C	5.004053	-2.519694	13.439671
H	4.196917	-3.139925	13.036007
H	4.929802	-1.544766	12.943149
C	4.821471	-2.325838	14.948616
H	3.854704	-1.861646	15.172502
H	5.605485	-1.679285	15.357442
H	4.863815	-3.283747	15.479935
H	7.190683	-2.553183	13.445529

09-TSPrim-EthOxyA

Charge	0
Electronic Energy, BS1 (a.u.)	-3333.736230
Thermal and entropic correction, BS1 (a.u.)	0.301214
Electronic Energy, BS2 (a.u.)	-11019.389669
Number of Imaginary Frequencies	1
Imaginary frequencies (cm-1)	-21.3i

Molecular Geometry in Cartesian Coordinates

Ag	5.718694	-1.003780	10.233582
Br	7.304969	2.053468	4.812977
Br	-0.582358	-0.481932	9.496176
Br	6.643837	3.511320	14.342628
F	5.991493	-1.465569	5.492302
F	6.825739	-1.908458	7.452113
F	8.023977	-0.859794	5.971774
F	6.260812	4.526532	6.313393
F	5.932162	4.484639	8.462261
F	4.247730	4.298644	7.096009
F	1.033539	-1.895327	12.111971
F	3.180715	-2.206809	11.942938
F	1.832341	-2.975672	10.407502
F	0.169365	2.102318	7.951206
F	2.222577	2.480745	7.339173
F	1.410703	3.466834	9.102283
F	7.243202	-0.104176	13.835239
F	8.800124	1.287393	13.225987
F	8.181268	-0.308744	11.880214
F	4.282479	4.906160	12.922767
F	2.980911	3.828314	11.554055
F	4.566522	5.101518	10.777136
C	5.990071	-3.098110	10.193114
N	5.532265	0.392012	8.097798
N	5.196848	1.680949	8.282156
N	3.356579	-0.001111	10.449457
N	3.169462	1.102130	9.706821
N	6.110368	1.183162	11.036724
N	5.203628	2.145061	10.780314
C	6.270223	0.338021	6.989384
C	6.412579	1.622047	6.424275
C	5.718986	2.458491	7.292882

C	6.782727	-0.972428	6.474185
C	5.535759	3.950377	7.284903
C	2.193749	-0.647188	10.483926
C	1.217786	0.043758	9.738287
C	1.886127	1.163858	9.254840
C	2.059359	-1.929923	11.241605
C	1.413605	2.309917	8.409480
C	6.694927	1.490909	12.193929
C	6.148192	2.675979	12.721099
C	5.192404	3.060998	11.785333
C	7.736870	0.588725	12.787486
C	4.253777	4.235343	11.760925
O	6.096444	-4.074828	12.152791
C	5.399414	-4.025774	11.143998
B	4.328681	2.116704	9.494329
H	3.884968	3.189873	9.290538
H	6.821775	-3.550863	9.642333
O	4.262048	-4.642371	10.914582
C	3.679399	-5.376428	12.042553
H	3.469210	-4.650489	12.832615
H	4.419178	-6.092728	12.409979
C	2.424874	-6.048174	11.530552
H	1.954617	-6.606584	12.345986
H	1.715310	-5.304997	11.160236
H	2.657945	-6.747937	10.722450
C	4.204188	-3.256847	7.981758
H	3.258292	-3.508284	8.466226
H	4.234197	-2.183434	7.779273
C	4.494345	-4.089436	6.737916
H	4.419800	-5.156678	6.979930
H	5.521095	-3.903783	6.406324
H	4.992050	-3.442239	8.760744
C	3.525562	-3.735727	5.601274
H	3.719227	-4.349874	4.714625
H	2.483086	-3.894852	5.900489
H	3.637480	-2.685027	5.311954

10-TSPrim-EthOxyB

Charge	0
Electronic Energy, BS1 (a.u.)	-3333.739155
Thermal and entropic correction, BS1 (a.u.)	0.303137
Electronic Energy, BS2 (a.u.)	-11019.391630
Number of Imaginary Frequencies	1
Imaginary frequencies (cm-1)	-75.5i

Molecular Geometry in Cartesian Coordinates

N	-1.421125	2.171513	-0.657442
N	-2.686118	1.979007	-0.255480
N	-1.945675	-0.805023	-0.619820
N	-2.922065	-0.483094	0.245205
N	-0.788916	0.379612	1.915733
N	-1.955251	1.043680	2.001688
B	-3.005938	0.946672	0.860603
C	-1.460698	2.996738	-1.703237
C	-2.791421	3.354287	-1.999467
C	-3.547328	2.680678	-1.042105
C	-2.112184	-2.084158	-0.952977
C	-3.221102	-2.625332	-0.274933
C	-3.711051	-1.563589	0.484281
C	-0.147564	0.572619	3.065760
C	-0.907229	1.387581	3.929582
C	-2.060539	1.671947	3.205146
H	-4.087886	1.137962	1.288797
C	-1.090000	-2.719644	-1.849224

F	-0.622871	-1.828861	-2.750225
F	-1.582475	-3.771624	-2.519856
C	-0.176644	3.273547	-2.422380
F	0.847243	3.409519	-1.556005
F	-0.225134	4.382432	-3.178411
C	1.171664	-0.087463	3.321133
F	1.687124	-0.620248	2.191280
F	1.062103	-1.080887	4.225091
F	2.080776	0.789358	3.804010
F	-0.024428	-3.148188	-1.130200
F	0.146858	2.238294	-3.246905
C	-3.280766	2.470687	3.559392
C	-4.873028	-1.477644	1.433980
C	-5.030175	2.631002	-0.805297
Br	-3.386392	4.492849	-3.388604
Br	-0.428574	1.916690	5.680657
Br	-3.872027	-4.396974	-0.391942
F	-3.094710	3.177392	4.685339
F	-3.587737	3.342548	2.572359
F	-4.354842	1.675761	3.743367
F	-5.350823	3.106284	0.414788
F	-5.699580	3.354286	-1.715808
F	-5.484520	1.359446	-0.877442
F	-4.477420	-0.985599	2.630198
F	-5.420107	-2.683258	1.650699
F	-5.839405	-0.664649	0.963099
C	2.681751	0.653768	-2.291302
O	2.644116	-0.332153	-3.024374
O	3.012626	1.884806	-2.590678
C	3.240723	2.186141	-4.009760
H	2.418769	1.751573	-4.583435
H	4.175930	1.705621	-4.310853
C	3.296052	3.692625	-4.133749
H	3.497699	3.961213	-5.175400
H	4.092672	4.107503	-3.509523
H	2.346052	4.143680	-3.837899
C	2.195272	0.250573	-0.988768
H	2.959830	-0.255382	-0.391373
H	2.568845	1.990739	-0.046211
C	2.822218	2.425353	0.940732
H	3.024974	1.606869	1.635436
C	1.673169	3.309820	1.425744
H	0.755739	2.713687	1.490810
H	1.474945	4.087718	0.681540
C	1.971410	3.955341	2.782804
H	2.140210	3.196578	3.552076
H	1.136635	4.584222	3.111177
H	2.865790	4.587365	2.732099
H	3.747538	2.993414	0.792090
Ag	0.166002	0.267308	-0.483168

11-TSSec-OxyA

Charge	0
Electronic Energy, BS1 (a.u.)	-3333.734636
Thermal and entropic correction, BS1 (a.u.)	0.298141
Electronic Energy, BS2 (a.u.)	-11019.387701
Number of Imaginary Frequencies	1
Imaginary frequencies (cm-1)	-90.5045

Molecular Geometry in Cartesian Coordinates

Ag	5.615000	-1.009946	10.066426
Br	8.339459	1.496256	5.142310

Br	-0.544883	-0.678468	8.247027
Br	5.819138	3.973662	13.856319
F	7.914332	-1.594248	5.810870
F	6.424908	-2.354362	7.208290
F	8.280055	-1.512462	7.957603
F	6.886242	4.082794	5.968894
F	6.099122	4.268876	7.988092
F	4.750367	3.892642	6.321471
F	0.902395	-1.829051	11.398386
F	2.940761	-2.483479	11.009150
F	1.334282	-3.028134	9.640645
F	0.409586	1.856672	6.742452
F	2.527844	2.253029	6.445532
F	1.436152	3.283457	8.022046
F	6.625166	0.519980	13.987786
F	8.225812	1.715093	13.132953
F	7.620627	-0.139439	12.166528
F	3.657768	5.129971	11.967075
F	2.594877	3.887609	10.532815
F	4.280648	5.103533	9.885228
C	6.167171	-3.007566	10.107508
N	5.709054	0.157372	7.962844
N	5.368907	1.454151	7.935838
N	3.199175	-0.111825	9.772905
N	3.108838	0.972985	8.983207
N	5.756010	1.253698	10.820478
N	4.898289	2.163076	10.320205
C	6.689899	-0.018673	7.078357
C	7.011483	1.203603	6.456576
C	6.137374	2.123376	7.035124
C	7.331120	-1.371308	6.995857
C	5.967829	3.601032	6.819625
C	2.061569	-0.787147	9.632353
C	1.198189	-0.128975	8.732410
C	1.912363	0.995046	8.332537
C	1.809521	-2.032262	10.423869
C	1.563288	2.103306	7.381925
C	6.164872	1.708631	12.003810
C	5.550171	2.940161	12.297548
C	4.740977	3.193289	11.194025
C	7.163465	0.949331	12.825152
C	3.816037	4.340220	10.893816
O	5.057398	-4.901262	10.900351
C	5.187130	-4.096287	9.994262
B	4.273497	1.993479	8.902882
H	3.879254	3.034316	8.514838
H	7.186964	-3.355032	9.906641
O	4.479737	-4.031783	8.873059
C	3.455134	-5.059962	8.676147
H	2.774439	-5.025139	9.528699
H	3.948118	-6.036512	8.658384
C	2.759725	-4.736372	7.372752
H	1.980449	-5.481085	7.182184
H	2.289485	-3.751383	7.423694
H	3.465249	-4.750398	6.536985
H	4.467848	-2.977992	12.867517
C	5.280082	-2.660093	13.525772
H	5.359578	-1.568808	13.479874
C	6.600630	-3.323896	13.143434
H	6.835304	-3.046633	12.091499
H	6.484920	-4.412317	13.125344
C	7.772459	-2.911221	14.032561
H	8.712156	-3.369807	13.705709
H	7.592709	-3.224021	15.068700
H	7.904434	-1.825727	14.033791
H	5.000910	-2.922751	14.553445

12-TSSec-OxyB

Charge	0
Electronic Energy, BS1 (a.u.)	-3333.734711
Thermal and entropic correction, BS1 (a.u.)	0.298595
Electronic Energy, BS2 (a.u.)	-11019.388713
Number of Imaginary Frequencies	1
Imaginary frequencies (cm-1)	-100.0i

Molecular Geometry in Cartesian Coordinates

```
Ag        5.689791     -1.076397      9.962635
Br        8.214909      1.640684      5.046327
Br       -0.510088     -0.794598      8.307508
Br        5.833121      3.722860     13.982219
F         7.861678     -1.476544      5.627506
F         6.422954     -2.304743      7.039681
F         8.290860     -1.470617      7.764107
F         6.774564      4.180039      6.031568
F         6.034828      4.279105      8.074717
F         4.650726      3.945885      6.428221
F         1.009283     -2.075118     11.331557
F         3.073906     -2.628350     10.924581
F         1.496213     -3.171293      9.521666
F         0.352269      1.824231      6.893177
F         2.454452      2.270817      6.555790
F         1.388509      3.218238      8.200572
F         6.655287      0.213052     13.897231
F         8.236469      1.538032     13.213663
F         7.735003     -0.238914     12.060895
F         3.667938      4.953320     12.150569
F         2.592197      3.750994     10.692139
F         4.252560      5.008343     10.058168
C         6.298686     -3.056968      9.975650
N         5.708436      0.168964      7.912361
N         5.351031      1.460910      7.943573
N         3.257932     -0.212428      9.764486
N         3.125998      0.903750      9.025841
N         5.803586      1.157255     10.813494
N         4.925710      2.073994     10.364425
C         6.659512      0.036813      6.988653
C         6.942973      1.284388      6.399534
C         6.079021      2.171548      7.041192
C         7.311998     -1.304720      6.836856
C         5.883971      3.653626      6.885414
C         2.131332     -0.905204      9.622614
C         1.232269     -0.227075      8.773937
C         1.912967      0.928346      8.406459
C         1.928453     -2.195228     10.354550
C         1.518555      2.066823      7.510991
C         6.214311      1.561567     12.014733
C         5.579205      2.765568     12.372794
C         4.758197      3.058328     11.287887
C         7.214890      0.766481     12.800034
C         3.814948      4.204198     11.047137
O         5.262705     -4.968576     10.792447
C         5.356396     -4.175854      9.869556
B         4.271929      1.947344      8.956378
H         3.851257      2.994371      8.615655
H         7.331000     -3.373380      9.786531
O         4.639974     -4.155791      8.754925
C         3.635780     -5.210646      8.593017
H         2.963680     -5.169263      9.452177
H         4.149651     -6.176432      8.592925
C         2.920242     -4.933238      7.290001
H         2.154239     -5.697897      7.126653
H         2.431182     -3.956688      7.322158
```

H	3.616847	-4.954091	6.446904
H	8.032125	-4.850400	13.407966
C	7.000324	-4.545018	13.614555
H	6.329640	-5.273460	13.149322
C	6.713388	-3.141456	13.089691
H	6.881187	-3.133566	11.990976
H	7.433461	-2.416842	13.486661
C	5.287049	-2.670904	13.361484
H	5.111269	-1.662608	12.974340
H	5.090129	-2.643407	14.439988
H	4.559716	-3.346415	12.901943
H	6.850972	-4.589453	14.700524

13-TSSec-EthOxyA

Charge	0
Electronic Energy, BS1 (a.u.)	-3333.739680
Thermal and entropic correction, BS1 (a.u.)	0.305125
Electronic Energy, BS2 (a.u.)	-11019.393482
Number of Imaginary Frequencies	1
Imaginary frequencies (cm-1)	-55.3i

Molecular Geometry in Cartesian Coordinates

Ag	5.707249	-0.973104	10.209791
Br	7.413174	1.944015	4.855514
Br	-0.620945	-0.276114	10.203651
Br	6.774098	3.691332	14.192681
F	6.704967	-1.370552	5.452996
F	6.819716	-1.960231	7.546726
F	8.410491	-0.773118	6.658682
F	6.028360	4.407447	6.057616
F	5.787773	4.523515	8.215247
F	4.066725	4.121519	6.943709
F	1.345591	-1.325473	12.891287
F	3.359158	-1.943274	12.339125
F	1.635328	-2.715083	11.250230
F	0.069146	2.000210	8.191146
F	2.083565	2.319508	7.432488
F	1.362615	3.473021	9.132506
F	7.379479	0.108450	13.803430
F	8.926566	1.427975	13.034034
F	8.210138	-0.231450	11.819345
F	4.394139	5.065676	12.771954
F	3.044690	3.924517	11.503664
F	4.590489	5.168831	10.609810
C	6.056516	-3.017992	10.479857
N	5.589535	0.377906	8.150864
N	5.158434	1.649927	8.242025
N	3.414817	0.118243	10.652797
N	3.184456	1.131560	9.800378
N	6.168466	1.277001	10.962533
N	5.258787	2.233260	10.698905
C	6.367158	0.308084	7.071540
C	6.445935	1.561920	6.434491
C	5.660600	2.398003	7.219716
C	7.074982	-0.953343	6.681983
C	5.380583	3.870860	7.103713
C	2.241941	-0.461018	10.893252
C	1.213441	0.178917	10.173604
C	1.862048	1.195960	9.481182
C	2.145536	-1.609168	11.846487
C	1.335714	2.253653	8.556691
C	6.774693	1.613892	12.098937
C	6.241011	2.814598	12.605170
C	5.267443	3.176503	11.678651

C	7.829103	0.727671	12.692315
C	4.323236	4.345045	11.642307
O	5.837594	-3.869955	12.418760
C	5.271772	-3.880298	11.324581
B	4.324637	2.134631	9.457957
H	3.860759	3.191629	9.218280
H	6.947970	-3.527422	10.099312
O	4.158950	-4.470268	10.977975
C	3.435202	-5.187818	12.039284
H	3.113491	-4.445956	12.773051
H	4.130734	-5.882611	12.517551
C	2.273896	-5.892921	11.377584
H	1.702229	-6.434822	12.137417
H	1.611129	-5.173094	10.892395
H	2.625485	-6.611670	10.631892
C	4.274947	-3.835351	7.890815
H	3.948199	-4.797916	8.299715
C	4.684546	-3.994914	6.427001
H	5.506803	-4.709477	6.311010
H	5.005651	-3.044073	5.994340
H	5.164444	-3.550550	8.484655
H	3.838575	-4.361208	5.832856
C	3.186006	-2.783969	8.103646
H	3.544824	-1.782871	7.836496
H	2.309888	-2.999863	7.480365
H	2.854700	-2.765060	9.142769

14-TSSec-EthOxyB

Charge	0
Electronic Energy, BS1 (a.u.)	-3333.740594
Thermal and entropic correction, BS1 (a.u.)	0.303904
Electronic Energy, BS2 (a.u.)	-11019.394049
Number of Imaginary Frequencies	1
Imaginary frequencies (cm-1)	-520.4i

Molecular Geometry in Cartesian Coordinates

Ag	5.660083	-1.002306	10.252179
Br	7.117593	1.838780	4.715521
Br	-0.653020	-0.578808	9.655663
Br	6.595702	3.706296	14.198579
F	5.885879	-1.680221	5.659548
F	6.824710	-1.969994	7.599939
F	7.924527	-1.000517	5.993384
F	6.040053	4.364252	6.100327
F	5.751015	4.433628	8.254368
F	4.048371	4.128767	6.933363
F	1.007224	-2.038357	12.108400
F	3.174802	-2.205233	12.050573
F	1.966763	-3.055686	10.447413
F	0.000491	1.999485	8.079271
F	2.018953	2.392005	7.370639
F	1.278096	3.386411	9.160974
F	7.201961	0.059974	13.837375
F	8.742145	1.439661	13.162951
F	8.123008	-0.209025	11.882538
F	4.220658	5.035996	12.741640
F	2.896803	3.890570	11.451341
F	4.468432	5.121228	10.584299
C	6.110411	-3.041888	10.432553
N	5.477643	0.324696	8.134539
N	5.099234	1.608984	8.250197
N	3.315570	-0.053404	10.447091
N	3.087754	1.044407	9.706218
N	6.026160	1.226941	11.011271

N	5.111423	2.171580	10.723892
C	6.190935	0.225172	7.013833
C	6.276117	1.476391	6.370369
C	5.573403	2.341218	7.204153
C	6.711309	-1.106228	6.562627
C	5.348144	3.824893	7.116092
C	2.159936	-0.706740	10.542241
C	1.147616	-0.030334	9.832897
C	1.784857	1.091228	9.311890
C	2.075233	-1.999912	11.291338
C	1.262163	2.223818	8.477824
C	6.626054	1.589596	12.143701
C	6.082235	2.796407	12.623259
C	5.111842	3.134816	11.684419
C	7.680382	0.717951	12.760274
C	4.172646	4.306663	11.616240
O	6.246468	-3.909909	12.370788
C	5.515658	-3.948633	11.379422
B	4.226507	2.075812	9.448026
H	3.759537	3.130339	9.204843
H	6.912776	-3.516180	9.857522
O	4.403398	-4.608750	11.200904
C	3.855255	-5.307779	12.373379
H	3.676005	-4.556186	13.146100
H	4.606983	-6.015430	12.732696
C	2.582227	-5.988023	11.924819
H	2.140570	-6.519256	12.773648
H	1.862453	-5.253629	11.557748
H	2.783574	-6.712127	11.130654
C	4.038059	-3.552986	7.910501
H	4.925118	-3.643102	7.274903
C	3.157731	-2.408153	7.405712
H	3.686959	-1.451824	7.425011
H	2.258737	-2.310221	8.021872
H	4.406430	-3.293180	8.918479
H	2.837219	-2.592371	6.373664
C	3.301199	-4.889472	7.989973
H	2.435160	-4.817152	8.655434
H	2.939325	-5.193220	7.000188
H	3.949643	-5.686442	8.368988

15-CH4

Charge	0
Electronic Energy, BS1 (a.u.)	-40.523140
Thermal and entropic correction, BS1 (a.u.)	0.025262
Electronic Energy, BS2 (a.u.)	-40.537463
Number of Imaginary Frequencies	0
Imaginary frequencies (cm-1)	None

Molecular Geometry in Cartesian Coordinates

C	3.671146	-3.403921	7.934004
H	2.839658	-3.795038	8.526120
H	3.584038	-2.316764	7.860279
H	4.616113	-3.660647	8.419312
H	3.644750	-3.841827	6.933185

16-TpBrCF3_2Ag_CHCO2Et

Charge	0
Electronic Energy, BS1 (a.u.)	-3214.563000
Thermal and entropic correction, BS1 (a.u.)	0.198183

Electronic Energy, BS2 (a.u.)	-10900.184680
Number of Imaginary Frequencies	0
Imaginary frequencies (cm-1)	None

Molecular Geometry in Cartesian Coordinates

Ag	5.614054	-1.032577	9.961373
Br	7.385740	1.988329	4.835087
Br	-0.665910	-0.376233	9.233309
Br	6.602501	3.321314	14.339880
F	6.580285	-1.160534	5.131739
F	5.955305	-2.037032	7.024373
F	7.949824	-1.193235	6.821377
F	6.369939	4.504881	6.304128
F	5.978831	4.464999	8.443322
F	4.327812	4.347260	7.029813
F	0.887265	-2.235166	11.457442
F	3.059843	-2.310623	11.576604
F	2.041252	-2.965116	9.766695
F	0.071247	2.372164	8.019386
F	2.113262	2.755127	7.374219
F	1.383298	3.600384	9.242583
F	7.087071	-0.305371	13.686476
F	8.690111	1.069189	13.168138
F	8.050437	-0.443189	11.739137
F	4.329408	4.873198	12.932342
F	2.979194	3.869534	11.553836
F	4.609647	5.091663	10.787890
C	6.158765	-3.026313	10.235661
N	5.401681	0.397371	8.025309
N	5.137753	1.700191	8.225285
N	3.275505	-0.008147	10.232920
N	3.088447	1.169204	9.614735
N	6.021820	1.134777	10.945856
N	5.146990	2.131721	10.721668
C	6.140331	0.309019	6.920383
C	6.380787	1.589715	6.386113
C	5.720408	2.454701	7.254775
C	6.655717	-1.023768	6.466168
C	5.596986	3.952728	7.251944
C	2.111921	-0.652363	10.202568
C	1.134144	0.116431	9.539131
C	1.803304	1.281734	9.177876
C	2.016517	-2.038768	10.756130
C	1.334099	2.509186	8.451952
C	6.607603	1.380455	12.116254
C	6.095575	2.563329	12.683957
C	5.159878	3.013496	11.757271
C	7.615095	0.423217	12.680913
C	4.267822	4.222767	11.760373
O	5.717554	-3.910143	12.077365
C	5.341020	-3.995967	10.904431
B	4.273389	2.158311	9.436452
H	3.867334	3.248473	9.245291
H	7.151158	-3.423760	9.998034
O	4.389708	-4.715712	10.385726
C	3.557204	-5.486491	11.329463
H	3.156803	-4.781524	12.061272
H	4.204340	-6.204357	11.840458
C	2.473886	-6.154551	10.515139
H	1.838222	-6.745541	11.181838

H	1.855111	-5.406860	10.014475
H	2.903024	-6.823737	9.764043

17-TpBr_3Ag_CHCO2Et

Charge	0
Electronic Energy, BS1 (a.u.)	-1267.777597
Thermal and entropic correction, BS1 (a.u.)	0.117943
Electronic Energy, BS2 (a.u.)	-24318.528158
Number of Imaginary Frequencies	0
Imaginary frequencies (cm-1)	None

Molecular Geometry in Cartesian Coordinates

Ag	5.397515	-0.983755	10.172756
Br	7.665390	0.880274	4.596526
Br	-0.807610	-0.078385	8.456465
Br	6.111513	4.257099	13.609831
C	5.830090	-2.988124	10.283922
N	5.814384	0.151436	8.160211
N	5.331697	1.393182	7.898265
N	3.113116	-0.046962	9.610979
N	3.043461	1.149892	8.969069
N	5.450277	1.213773	10.968683
N	4.930218	2.256206	10.261122
C	6.615768	-0.184374	7.159277
C	6.680129	0.843774	6.200519
C	5.841603	1.831207	6.719043
C	1.911168	-0.595465	9.537707
C	1.011323	0.232009	8.835865
C	1.785346	1.338068	8.491376
C	5.892767	1.701593	12.120281
C	5.672301	3.089077	12.200215
C	5.054571	3.395772	10.989423
O	4.554354	-4.620971	11.015933
C	4.874534	-4.031919	9.986609
B	4.297651	2.054260	8.856707
H	3.986352	3.106193	8.415474
H	6.835766	-3.393070	10.440995
O	4.406115	-4.193123	8.770169
C	3.357892	-5.203291	8.583342
H	2.591230	-5.046404	9.345480
H	3.808768	-6.188390	8.736146
C	2.819380	-5.022611	7.182307
H	2.036563	-5.764749	6.997048
H	2.387579	-4.025447	7.058692
H	3.608890	-5.158301	6.437545
Br	1.597251	-2.275030	10.344849
Br	1.245885	2.864157	7.526872
Br	7.480613	-1.863283	7.167896
Br	5.445317	3.509378	5.963942
Br	4.466853	5.087666	10.407132
Br	6.691093	0.556231	13.387685

18-TpF27Ag_CHCO2Et

Charge	0
Electronic Energy, BS1 (a.u.)	-4530.768216
Thermal and entropic correction, BS1 (a.u.)	0.286631

Electronic Energy, BS2 (a.u.)	-4533.963275
Number of Imaginary Frequencies	0
Imaginary frequencies (cm-1)	None

Molecular Geometry in Cartesian Coordinates

Ag	5.143395	-0.897101	9.991097
F	7.021524	-1.438265	5.334089
F	5.780809	-2.135910	7.027358
F	0.394010	-2.072910	9.957895
F	2.449293	-2.152219	10.761499
F	7.561062	1.344498	13.750342
F	7.357997	-0.179826	12.160656
C	5.942745	-2.716597	10.623800
N	5.515017	0.365959	7.895072
N	5.359503	1.704046	7.987770
N	3.044699	0.179055	9.507044
N	3.031833	1.389596	8.901684
N	5.590250	1.384290	10.866590
N	4.844441	2.392573	10.365303
C	6.464470	0.107377	7.008760
C	6.998456	1.316125	6.488109
C	6.251850	2.325605	7.155730
C	6.805821	-1.309305	6.672988
C	1.932731	-0.468382	9.191702
C	1.130123	0.320510	8.324403
C	1.883072	1.516061	8.164693
C	1.705417	-1.874443	9.655795
C	5.898180	1.669759	12.122139
C	5.328839	2.915034	12.503409
C	4.652356	3.346086	11.329948
C	6.670279	0.696062	12.952587
O	4.658826	-4.371628	10.706029
C	5.559556	-3.936523	9.980495
B	4.280589	2.317972	8.925470
H	3.986213	3.399652	8.548665
H	6.615894	-2.861544	11.473412
O	5.995956	-4.372747	8.833443
C	5.260199	-5.474161	8.188462
H	4.230268	-5.461946	8.550784
H	5.742018	-6.402962	8.505129
C	5.351789	-5.249047	6.695030
H	4.905891	-6.102264	6.174325
H	4.815846	-4.342116	6.409013
H	6.394226	-5.151631	6.380047
C	7.998681	1.663678	5.565574
C	8.236598	2.999013	5.322531
C	7.493679	4.000862	5.993118
C	6.506590	3.685561	6.905413
C	3.943027	4.560472	11.319356
C	5.301150	3.698763	13.668746
C	4.607780	4.889406	13.646868
C	3.933091	5.312966	12.476848
C	-0.101517	0.159563	7.667444
C	1.398965	2.558019	7.351827
C	0.184484	2.375919	6.720793
C	-0.565541	1.186013	6.874400
F	-0.813133	-0.967781	7.791872
F	-1.731429	1.079940	6.223830
F	-0.309979	3.336361	5.932152
F	2.076741	3.698172	7.160871

F	5.924129	3.301925	14.785709
F	4.547653	5.670499	14.733443
F	3.265876	6.471704	12.517214
F	3.272257	4.988858	10.240518
F	8.717285	0.722780	4.939129
F	9.182479	3.383085	4.455350
F	7.778776	5.280752	5.729051
F	5.841563	4.663613	7.536595
C	8.074067	-1.850769	7.372601
F	7.926192	-1.804442	8.714285
F	8.306384	-3.120117	7.017856
F	9.133657	-1.101418	7.040100
C	2.077051	-2.936023	8.587812
F	3.376527	-2.814133	8.235201
F	1.888391	-4.175267	9.055125
F	1.328556	-2.762607	7.489966
C	5.777057	-0.160668	13.881778
F	5.093903	0.631768	14.719329
F	4.897732	-0.871798	13.149741
F	6.520745	-1.014798	14.595858

19-TpF27Ag_CHCO2Et

Charge	0
Electronic Energy, BS1 (a.u.)	-4530.768250
Thermal and entropic correction, BS1 (a.u.)	0.285373
Electronic Energy, BS2 (a.u.)	-4533.963485
Number of Imaginary Frequencies	0
Imaginary frequencies (cm-1)	None

Molecular Geometry in Cartesian Coordinates

F	7.445924	-1.370340	5.300483
F	6.196856	-2.177624	6.937197
F	0.166973	-1.789507	10.282543
F	2.250588	-1.945658	11.001272
F	7.046492	0.688490	13.858367
F	6.973296	-0.695632	12.140090
N	5.593937	0.324814	7.731337
N	5.351656	1.649073	7.839858
N	2.972200	0.094918	9.433882
N	3.020100	1.247161	8.731312
N	5.429985	1.062733	10.766217
N	4.803262	2.153122	10.265787
C	6.592568	0.145533	6.880839
C	7.065479	1.395901	6.398080
C	6.229598	2.343179	7.049736
C	7.122934	-1.226166	6.613766
C	1.804427	-0.489161	9.213810
C	1.028257	0.279465	8.305680
C	1.860212	1.396272	8.018529
C	1.471941	-1.779169	9.892660
C	5.632706	1.243268	12.062706
C	5.110932	2.498811	12.474554
C	4.584624	3.053647	11.275809
C	6.237093	0.159550	12.901746
B	4.263850	2.177933	8.806914
H	3.960458	3.281159	8.508929
C	8.075325	1.823009	5.520026
C	8.233210	3.174660	5.303611

C	7.400260	4.114340	5.956921
C	6.402627	3.721036	6.826539
C	3.969755	4.319708	11.288806
C	5.015635	3.200535	13.688244
C	4.411600	4.438628	13.688964
C	3.893868	4.990243	12.493300
C	-0.235762	0.160680	7.705181
C	1.431541	2.395440	7.126753
C	0.183992	2.256630	6.551759
C	-0.649390	1.148230	6.837791
F	-1.023894	-0.892915	7.955888
F	-1.843748	1.078968	6.235304
F	-0.263338	3.176679	5.690033
F	2.199949	3.447462	6.810315
F	5.486886	2.682393	14.829228
F	4.292404	5.145677	14.820137
F	3.310986	6.192523	12.553396
F	3.449356	4.880790	10.188828
F	8.878903	0.943805	4.908054
F	9.183180	3.632976	4.477895
F	7.607208	5.414277	5.718674
F	5.651109	4.646410	7.439558
C	8.396086	-1.559485	7.427991
F	9.372352	-0.691966	7.128522
F	8.135259	-1.474961	8.747350
F	8.816481	-2.802194	7.159977
C	1.673429	-3.032323	9.008583
F	0.920942	-2.932809	7.903801
F	2.965649	-3.138364	8.633939
F	1.331251	-4.143147	9.671031
C	5.175456	-0.694439	13.642423
F	4.458259	0.086148	14.463945
F	4.327942	-1.263069	12.755828
F	5.749060	-1.668595	14.354394
C	5.575375	-3.102258	10.000557
H	6.206169	-3.719880	9.354864
C	5.118091	-3.778539	11.176763
O	6.081068	-3.708480	11.947330
O	3.914042	-4.228334	11.390160
C	3.569103	-4.581529	12.777925
H	4.099792	-5.503463	13.031679
H	3.926563	-3.776135	13.423975
C	2.066216	-4.741948	12.823283
H	1.764048	-5.005850	13.841478
H	1.736371	-5.534608	12.146526
H	1.572250	-3.810710	12.538925
Ag	5.144529	-1.080572	9.725134

20-TpBrCF3_2Ag_CHCF3

Charge	0
Electronic Energy, BS1 (a.u.)	-3284.372007
Thermal and entropic correction, BS1 (a.u.)	0.135480
Electronic Energy, BS2 (a.u.)	-10970.049261
Number of Imaginary Frequencies	0
Imaginary frequencies (cm-1)	None

Molecular Geometry in Cartesian Coordinates

Ag	5.582091	-0.941237	10.235711
Br	7.407215	1.957366	4.841949
Br	-0.573183	-0.520211	9.428696
Br	6.805826	3.588377	14.184770
F	6.593546	-1.234095	5.246333
F	6.079515	-2.016363	7.214333
F	8.033378	-1.121672	6.871371
F	6.248549	4.498651	6.177593
F	5.958270	4.520866	8.333822
F	4.247434	4.307428	7.005502
F	1.111110	-2.633754	11.067091
F	3.105580	-2.234121	11.848985
F	2.866441	-2.981042	9.823992
F	-0.000150	2.273447	8.274424
F	1.987441	2.751670	7.532854
F	1.332880	3.509719	9.465774
F	6.999818	-0.471125	13.323285
F	8.553726	1.049487	13.467952
F	8.331123	-0.118977	11.642971
F	4.514162	5.081695	12.793858
F	3.082306	4.004322	11.562235
F	4.664737	5.172054	10.628785
C	6.194123	-2.866792	10.259208
N	5.420661	0.423152	8.057109
N	5.128463	1.724962	8.216256
N	3.401550	-0.045890	10.223449
N	3.141796	1.143756	9.658802
N	6.046581	1.208155	10.958594
N	5.184851	2.208375	10.706163
C	6.175511	0.320900	6.968683
C	6.393714	1.590654	6.395063
C	5.702941	2.466150	7.227183
C	6.720819	-1.015158	6.566255
C	5.538157	3.957296	7.178190
C	2.267066	-0.745007	10.238033
C	1.227582	0.007291	9.658118
C	1.832547	1.212477	9.301312
C	2.319928	-2.154482	10.751855
C	1.277907	2.444894	8.641285
C	6.691048	1.504236	12.088001
C	6.229845	2.730342	12.602030
C	5.264543	3.147200	11.685392
C	7.657402	0.496549	12.641063
C	4.380401	4.365108	11.668366
B	4.281432	2.182594	9.433334
H	3.829754	3.253723	9.243173
H	6.565544	-3.448094	9.407883
C	5.986976	-3.801792	11.416996
F	5.669386	-3.182047	12.561178
F	4.962636	-4.617305	11.078884
F	7.095052	-4.536258	11.639258

21-TpBrCF3_2Ag_C-Ph-CO2Et

Charge	0
Electronic Energy, BS1 (a.u.)	-3445.674113
Thermal and entropic correction, BS1 (a.u.)	0.273427
Electronic Energy, BS2 (a.u.)	-11131.371479
Number of Imaginary Frequencies	0
Imaginary frequencies (cm-1)	None

Molecular Geometry in Cartesian Coordinates

Ag	5.505439	-1.113946	10.234283
Br	7.294789	1.437453	4.760079
Br	-0.744728	-0.298498	9.780966
Br	6.808352	3.604161	14.055515
F	6.196114	-1.673743	5.291535
F	5.854675	-2.352928	7.331847
F	7.793015	-1.541464	6.760732
F	6.356197	4.100470	6.035227
F	5.995953	4.234708	8.176374
F	4.320898	4.054048	6.798018
F	1.014844	-1.749379	12.298610
F	3.103150	-2.225091	11.911929
F	1.555315	-2.882705	10.526770
F	0.008100	2.221647	8.139140
F	2.029262	2.524175	7.393779
F	1.366333	3.551123	9.194819
F	7.128933	-0.451972	13.219610
F	8.639037	1.114176	13.306254
F	8.403544	-0.080214	11.499442
F	4.473923	5.048135	12.644054
F	3.066952	3.940562	11.409413
F	4.654945	5.102027	10.478415
C	6.323554	-3.050884	10.543010
N	5.268086	0.160624	8.064835
N	5.058264	1.484145	8.169445
N	3.271030	0.008705	10.434726
N	3.077024	1.119030	9.701402
N	6.086272	1.179021	10.847493
N	5.197831	2.154368	10.602815
C	6.000461	-0.030731	6.969586
C	6.286949	1.198446	6.342584
C	5.663731	2.147491	7.146429
C	6.454535	-1.404945	6.584520
C	5.583080	3.642518	7.032564
C	2.087108	-0.587088	10.555909
C	1.090449	0.144903	9.880722
C	1.769264	1.232393	9.341555
C	1.941304	-1.861319	11.328069
C	1.285727	2.389162	8.515318
C	6.729983	1.500922	11.968729
C	6.240946	2.717914	12.483368
C	5.257481	3.103758	11.573900
C	7.733887	0.526847	12.508370
C	4.362457	4.309285	11.529630
O	5.494534	-4.203102	12.399667
C	5.410223	-3.998969	11.200097
B	4.258941	2.068922	9.366404
H	3.851250	3.141857	9.094510
O	4.499902	-4.517581	10.375223
C	3.480186	-5.366876	10.982103
H	2.939088	-4.772250	11.721221
H	3.976358	-6.193081	11.500584
C	2.582540	-5.846991	9.862921
H	1.790591	-6.480404	10.275116
H	2.117954	-4.999810	9.353061
H	3.147270	-6.431600	9.130462
C	7.614220	-3.507474	10.179792
C	8.030803	-4.859942	10.372305
C	8.522322	-2.600811	9.560630
C	9.282885	-5.275339	9.957356

C	9.770129	-3.026887	9.136051
C	10.148326	-4.360329	9.336251
H	7.359955	-5.559149	10.860673
H	8.212902	-1.571868	9.422033
H	9.597438	-6.303003	10.107150
H	10.450623	-2.332805	8.654415
H	11.128799	-4.693375	9.007439

22-TpBrCF3_2Ag_C-pCF3Ph-CO2CH2CF3

Charge	0
Electronic Energy, BS1 (a.u.)	-4080.4111805
Thermal and entropic correction, BS1 (a.u.)	0.246491
Electronic Energy, BS2 (a.u.)	-11766.4174587
Number of Imaginary Frequencies	0
Imaginary frequencies (cm-1)	None

Molecular Geometry in Cartesian Coordinates

Ag	5.496227	-1.087900	9.995770
Br	7.528448	2.066405	4.871285
Br	-0.643722	-0.474491	8.889915
Br	6.459194	3.306455	14.298140
F	6.480741	-1.126816	5.048555
F	6.137672	-2.000779	7.014029
F	8.051764	-1.070117	6.549988
F	6.453274	4.556857	6.364761
F	5.961440	4.467019	8.481824
F	4.379186	4.375997	6.989653
F	0.895807	-2.665608	10.598640
F	2.836902	-2.314428	11.525601
F	2.724314	-2.967360	9.453677
F	0.059231	2.340308	7.851682
F	2.107175	2.788593	7.272365
F	1.319053	3.535428	9.158802
F	7.099208	-0.288458	13.595076
F	8.646152	1.153915	13.092207
F	8.060988	-0.362066	11.643278
F	4.130788	4.788390	12.896716
F	2.841517	3.766296	11.473858
F	4.442312	5.058644	10.762335
C	6.291568	-2.996562	10.489336
N	5.410048	0.403647	7.937459
N	5.141081	1.702539	8.157862
N	3.260904	-0.074614	10.022581
N	3.060743	1.129043	9.466794
N	5.984235	1.148832	10.868252
N	5.073505	2.114903	10.653114
C	6.197495	0.347891	6.865675
C	6.462750	1.641387	6.374092
C	5.766703	2.483975	7.234738
C	6.708795	-0.965538	6.362415
C	5.638458	3.980391	7.262228
C	2.118807	-0.752145	9.924663
C	1.138785	0.026268	9.277782
C	1.786483	1.229259	9.000865
C	2.123241	-2.180812	10.375234
C	1.308725	2.480177	8.319763
C	6.550774	1.398812	12.047331
C	5.990916	2.553172	12.628546

C	5.045495	2.981667	11.701291
C	7.593922	0.473702	12.599551
C	4.112985	4.159252	11.711582
O	5.715676	-3.432583	12.704391
C	5.536076	-3.703528	11.532309
B	4.234506	2.139422	9.344870
H	3.816201	3.225269	9.154522
O	4.648858	-4.598074	11.069324
C	3.799306	-5.189060	12.065959
H	3.246973	-4.420586	12.608414
H	4.380222	-5.790866	12.768574
C	7.501666	-3.584650	10.033177
C	7.911242	-4.894923	10.420586
C	8.320792	-2.860140	9.124480
C	9.073150	-5.446807	9.914405
C	9.483190	-3.415260	8.615142
C	9.852998	-4.704304	9.013603
H	7.301125	-5.468902	11.109998
H	8.021278	-1.862920	8.831064
H	9.381858	-6.446753	10.197570
H	10.094179	-2.865156	7.909313
C	11.148757	-5.303551	8.510806
F	12.133778	-5.114187	9.413801
F	11.030964	-6.632190	8.314120
F	11.543864	-4.745869	7.351407
C	2.824484	-6.086415	11.334250
F	2.022186	-5.393936	10.506843
F	3.460789	-7.024183	10.606578
F	2.049364	-6.714025	12.242959

23-TpF27Ag_C-pCF3Ph-CO2CH2CF3

Charge	0
Electronic Energy, BS1 (a.u.)	-5396.610776
Thermal and entropic correction, BS1 (a.u.)	0.3322250
Electronic Energy, BS2 (a.u.)	-5400.194327
Number of Imaginary Frequencies	0
Imaginary frequencies (cm-1)	None

Molecular Geometry in Cartesian Coordinates

F	6.383183	-1.808464	5.302248
F	5.928378	-2.360323	7.391787
F	0.346857	-1.778965	10.346199
F	2.472756	-2.151495	10.811294
F	7.562479	1.289444	14.011818
F	7.465236	-0.235215	12.418600
N	5.659004	0.257993	8.014741
N	5.443653	1.594985	8.009538
N	3.144113	0.211461	9.628416
N	3.168239	1.454330	9.094977
N	5.777071	1.321542	10.994033
N	5.095010	2.349649	10.443835
C	6.260215	-0.081223	6.885472
C	6.482776	1.065522	6.076362
C	5.935713	2.128907	6.844604
C	6.636044	-1.501946	6.602926
C	1.932038	-0.291476	9.456446
C	1.097314	0.631173	8.768011
C	1.942232	1.753916	8.559303

C	1.610609	-1.694267	9.853183
C	6.028312	1.619454	12.261384
C	5.475915	2.883963	12.599689
C	4.874022	3.319158	11.388474
C	6.725194	0.646634	13.153572
B	4.484923	2.273338	9.027035
H	4.243733	3.362749	8.647180
C	7.064109	1.314478	4.821393
C	7.094681	2.609674	4.353383
C	6.555339	3.667026	5.122642
C	5.980846	3.450206	6.359528
C	4.184719	4.545731	11.336606
C	5.400187	3.674782	13.758759
C	4.731975	4.877774	13.694259
C	4.127320	5.304719	12.488447
C	-0.226257	0.635839	8.297490
C	1.454501	2.898496	7.899950
C	0.146970	2.880895	7.456454
C	-0.692515	1.758642	7.649583
F	-1.024721	-0.427439	8.457968
F	-1.946076	1.812339	7.180450
F	-0.356578	3.943477	6.818371
F	2.208054	3.984813	7.679740
F	5.949266	3.273362	14.912173
F	4.627832	5.665618	14.772575
F	3.476780	6.473646	12.487508
F	3.571042	4.985001	10.228238
F	7.586808	0.324095	4.087675
F	7.641552	2.899656	3.165933
F	6.623684	4.907062	4.626704
F	5.511046	4.494815	7.054528
C	8.132003	-1.800459	6.854000
F	8.896797	-1.067491	6.034737
F	8.446780	-1.480132	8.126905
F	8.402425	-3.095597	6.664050
C	1.706695	-2.683619	8.665239
F	1.009427	-2.226629	7.616154
F	2.992180	-2.829334	8.305491
F	1.211753	-3.884348	9.010171
C	5.755740	-0.197177	14.015516
F	5.061009	0.606592	14.834060
F	4.891464	-0.858008	13.226148
F	6.432769	-1.089503	14.749348
C	5.993334	-2.839033	10.930071
C	4.901179	-3.743385	11.314443
O	4.424074	-3.727004	12.432405
O	4.495072	-4.531097	10.305514
C	3.392691	-5.400053	10.602742
H	3.577664	-5.979449	11.510065
H	2.473313	-4.827312	10.717643
Ag	5.350424	-1.002525	10.057903
C	7.310960	-3.272926	11.242426
C	8.412600	-2.438443	10.911304
C	7.577015	-4.537211	11.850124
C	9.715142	-2.846754	11.153595
C	8.874437	-4.937979	12.101991
C	9.938033	-4.091349	11.748824
H	8.213614	-1.472194	10.468723
H	6.753032	-5.184605	12.130445
H	10.553053	-2.209995	10.896447
H	9.078708	-5.894100	12.571414
C	11.347906	-4.576824	12.004463

F	12.257353	-3.594225	11.871338
F	11.463586	-5.084681	13.248755
F	11.671806	-5.559385	11.137631
C	3.270850	-6.348056	9.428428
F	3.117544	-5.700914	8.261092
F	4.361747	-7.134292	9.321579
F	2.198606	-7.143816	9.616813

24-TpF27Ag_C-Ph-CO2Et

Charge	0
Electronic Energy, BS1 (a.u.)	-4761.872626
Thermal and entropic correction, BS1 (a.u.)	0.360818
Electronic Energy, BS2 (a.u.)	-4765.145759
Number of Imaginary Frequencies	0
Imaginary frequencies (cm-1)	None

Molecular Geometry in Cartesian Coordinates

F	6.130841	-1.726618	5.070765
F	5.416985	-2.372559	7.061986
F	0.344645	-1.705027	10.543299
F	2.482768	-2.092861	10.937607
F	7.499656	1.324825	13.996452
F	7.474720	-0.188061	12.388912
N	5.500056	0.181711	7.899621
N	5.440631	1.531243	7.975295
N	3.137907	0.218785	9.652368
N	3.162009	1.441073	9.072162
N	5.793768	1.341981	10.932730
N	5.097014	2.358018	10.378049
C	6.161822	-0.155059	6.803585
C	6.596046	1.006085	6.109433
C	6.106432	2.074933	6.907396
C	6.323934	-1.590160	6.412514
C	1.914280	-0.272373	9.541399
C	1.067962	0.639571	8.852334
C	1.920434	1.741953	8.574788
C	1.591285	-1.656881	10.001400
C	6.003167	1.634152	12.208829
C	5.407586	2.880342	12.547073
C	4.824297	3.310979	11.325695
C	6.698608	0.669887	13.110591
B	4.482434	2.250398	8.966085
H	4.247799	3.329906	8.548443
C	7.323506	1.266897	4.936317
C	7.552355	2.577926	4.579595
C	7.070096	3.639860	5.381210
C	6.355218	3.410207	6.539985
C	4.101043	4.516723	11.265711
C	5.281529	3.659548	13.709369
C	4.580416	4.843747	13.637753
C	3.992750	5.264069	12.421479
C	-0.273636	0.652083	8.436450
C	1.421402	2.875734	7.905594
C	0.096587	2.866226	7.516457
C	-0.749928	1.762948	7.775236
F	-1.081667	-0.392183	8.662300
F	-2.021402	1.822796	7.356609
F	-0.417873	3.919433	6.870834

F	2.179106	3.945736	7.627940
F	5.814936	3.266564	14.873331
F	4.428998	5.619888	14.719758
F	3.309054	6.414611	12.413856
F	3.503796	4.945726	10.143749
F	7.798511	0.270293	4.178106
F	8.245582	2.879342	3.473637
F	7.338019	4.892820	4.996959
F	5.946368	4.448254	7.282621
C	7.718238	-2.178180	6.722380
F	8.678535	-1.416370	6.177422
F	7.906327	-2.212530	8.055949
F	7.828773	-3.422968	6.246995
C	1.622827	-2.686322	8.845024
F	0.867712	-2.263091	7.820944
F	2.886583	-2.847657	8.414724
F	1.154081	-3.877097	9.252420
C	5.725868	-0.199568	13.942958
F	5.001186	0.584364	14.756465
F	4.888631	-0.865527	13.130902
F	6.402885	-1.087533	14.682502
C	6.044896	-2.838369	10.881504
C	4.982909	-3.786194	11.256962
O	4.502826	-3.768485	12.377698
O	4.610874	-4.584223	10.255449
C	3.498762	-5.487451	10.530693
H	3.783863	-6.151713	11.352676
H	2.645325	-4.889237	10.855042
C	3.219599	-6.244986	9.250978
H	2.370824	-6.919480	9.401772
H	4.087080	-6.840679	8.951683
H	2.973433	-5.554118	8.441879
Ag	5.300715	-1.064431	9.961895
C	7.372323	-3.185961	11.239781
C	8.445553	-2.321673	10.878329
C	7.683954	-4.396040	11.932348
C	9.756845	-2.654705	11.175200
C	8.994135	-4.715374	12.239332
C	10.028906	-3.847302	11.856823
H	8.212087	-1.400832	10.359207
H	6.879948	-5.057269	12.238392
H	10.567849	-1.993295	10.889124
H	9.224457	-5.631954	12.772599
H	11.057003	-4.104419	12.096184

25-TpBr_3Ag_C-Ph-CO2Et

Charge	0
Electronic Energy, BS1 (a.u.)	-1498.887310
Thermal and entropic correction, BS1 (a.u.)	0.194056
Electronic Energy, BS2 (a.u.)	-24549.712182
Number of Imaginary Frequencies	0
Imaginary frequencies (cm-1)	None

Molecular Geometry in Cartesian Coordinates

Ag	5.289354	-1.170179	10.024692
Br	7.886639	1.402299	4.833950
Br	-0.756272	-0.370638	8.286071
Br	5.886383	3.827347	13.909119

C	5.905324	-3.173310	10.322966
N	5.646640	0.134232	8.008878
N	5.249850	1.433457	7.939258
N	3.072444	-0.132953	9.698602
N	2.992312	1.044837	9.023218
N	5.539209	1.075736	10.906748
N	4.865631	2.121028	10.352350
C	6.535647	-0.052651	7.042944
C	6.743634	1.129760	6.306576
C	5.898818	2.052330	6.921117
C	1.902307	-0.739430	9.558939
C	1.017914	0.030085	8.777637
C	1.764622	1.162334	8.457048
C	5.947824	1.470398	12.104131
C	5.552919	2.795578	12.369037
C	4.860887	3.166635	11.217970
O	4.294891	-4.793303	10.883278
C	4.853544	-4.155888	10.004966
B	4.213618	1.997670	8.949434
H	3.858685	3.067365	8.586628
O	4.562915	-4.183011	8.702206
C	3.449885	-5.018437	8.270686
H	2.674852	-4.997039	9.039300
H	3.816187	-6.046596	8.177395
C	2.961019	-4.461402	6.950800
H	2.130910	-5.070816	6.579190
H	2.607958	-3.433553	7.074924
H	3.758228	-4.466785	6.201481
Br	1.625508	-2.422087	10.367653
Br	1.233437	2.637838	7.412156
Br	7.344438	-1.743158	6.812306
Br	5.654812	3.865485	6.471292
Br	4.031976	4.820746	10.862622
Br	6.914139	0.284058	13.212093
C	7.197109	-3.659713	10.653317
C	8.238690	-2.725337	10.921911
C	7.509699	-5.052003	10.689800
C	9.528478	-3.156923	11.187761
C	8.796054	-5.476478	10.973514
C	9.804151	-4.530430	11.215825
H	7.999140	-1.667354	10.903832
H	6.723072	-5.777527	10.511956
H	10.318314	-2.437943	11.378622
H	9.028346	-6.536020	11.005221
H	10.813663	-4.869344	11.431022

26-TpBr_3Ag_C-p-CF3Ph-CO2CH2CF3

Charge	0
Electronic Energy, BS1 (a.u.)	-2133.629281
Thermal and entropic correction, BS1 (a.u.)	0.165948
Electronic Energy, BS2 (a.u.)	-25184.760326
Number of Imaginary Frequencies	0
Imaginary frequencies (cm-1)	None

Molecular Geometry in Cartesian Coordinates

Ag	5.270643	-1.272748	10.016533
Br	7.532954	1.057007	4.594056
Br	-0.779728	-0.695154	8.487158

Br	5.917817	3.831925	13.765421
C	5.968128	-3.249579	10.312670
N	5.540023	-0.050535	7.983503
N	5.120261	1.237747	7.871404
N	3.038000	-0.238385	9.874268
N	2.932475	0.868822	9.089162
N	5.570384	1.004426	10.834205
N	4.852168	2.018032	10.275612
C	6.362957	-0.283802	6.970034
C	6.499486	0.856873	6.155833
C	5.685706	1.803808	6.775998
C	1.876154	-0.873739	9.799874
C	0.977342	-0.201616	8.950740
C	1.702826	0.907109	8.518516
C	5.997335	1.437894	12.011261
C	5.572385	2.757004	12.258240
C	4.839626	3.082081	11.118514
O	4.217805	-4.771487	10.726863
C	4.930477	-4.208470	9.919995
B	4.134302	1.834522	8.912513
H	3.747926	2.884932	8.525287
O	4.816631	-4.288522	8.574379
C	3.544678	-4.665268	8.034454
H	2.788137	-4.727150	8.817997
H	3.628481	-5.624326	7.519367
Br	1.640219	-2.492261	10.738171
Br	1.150256	2.258949	7.328998
Br	7.180892	-1.973464	6.784273
Br	5.382693	3.584651	6.243761
Br	3.954607	4.703573	10.750391
Br	7.017954	0.302573	13.124994
C	7.258110	-3.718106	10.677247
C	8.271734	-2.770357	10.988616
C	7.583881	-5.105296	10.722027
C	9.554443	-3.183641	11.312559
C	8.858334	-5.515847	11.067643
C	9.838362	-4.553314	11.355960
H	8.022317	-1.715352	10.959436
H	6.819912	-5.843759	10.502183
H	10.328639	-2.461310	11.543056
H	9.106439	-6.570253	11.117537
C	11.240223	-5.025801	11.674317
F	11.964935	-4.075759	12.293509
F	11.218182	-6.115640	12.468185
F	11.890212	-5.365250	10.541141
C	3.151399	-3.593269	7.032211
F	3.176293	-2.364801	7.589305
F	3.976048	-3.574626	5.969187
F	1.900515	-3.832011	6.590714

27-TpBr_3Ag_CHCF3

Charge	0
Electronic Energy, BS1 (a.u.)	-1337.592478
Thermal and entropic correction, BS1 (a.u.)	0.054689
Electronic Energy, BS2 (a.u.)	-24388.397365
Number of Imaginary Frequencies	0
Imaginary frequencies (cm-1)	None

Molecular Geometry in Cartesian Coordinates

N	-2.090437	2.232148	-1.444452
N	-3.256008	1.994795	-0.785157
N	-1.739020	-0.647033	-0.563701
N	-2.944112	-0.393718	0.013902
N	-0.972296	1.646116	1.260042
N	-2.302395	1.537986	1.527498
B	-3.307600	1.045627	0.446598
C	-2.361259	3.043197	-2.460961
C	-3.731616	3.359486	-2.493049
C	-4.259779	2.666183	-1.404160
C	-1.695804	-1.946200	-0.809412
C	-2.881988	-2.587456	-0.396613
C	-3.650233	-1.549422	0.125113
C	-0.371512	2.051807	2.373393
C	-1.306031	2.217977	3.411637
C	-2.523348	1.878022	2.822576
H	-4.399009	1.061579	0.899447
Br	-4.639485	4.463445	-3.716852
Br	-0.987917	2.772482	5.181434
Br	-3.326364	-4.413237	-0.520471
C	1.679546	1.065820	-1.730396
H	2.079841	0.156880	-2.193616
Ag	-0.098957	1.292083	-0.823894
Br	-0.986020	3.613448	-3.616352
Br	-6.053977	2.622628	-0.839037
Br	1.491089	2.335520	2.403394
Br	-4.220813	1.869335	3.634717
Br	-5.376399	-1.656057	0.871661
Br	-0.165201	-2.707002	-1.616363
C	2.671519	2.170987	-1.955121
F	2.279789	3.358287	-1.469617
F	3.827404	1.832256	-1.340837
F	2.894193	2.321414	-3.279215

28-TpBr_3Ag_CHCO2Et_CH4_Ethoxy_Int-I

Charge	0
Electronic Energy, BS1 (a.u.)	-1308.311854
Thermal and entropic correction, BS1 (a.u.)	0.160827
Electronic Energy, BS2 (a.u.)	-24359.068953
Number of Imaginary Frequencies	0
Imaginary frequencies (cm-1)	None

Molecular Geometry in Cartesian Coordinates

N	-1.465006	1.288591	-1.443124
N	-2.356824	1.697995	-0.502542
N	-2.018761	-1.391810	-0.278349
N	-2.906184	-0.612108	0.401989
N	-0.120354	0.408624	1.193518
N	-1.292624	0.908074	1.661386
B	-2.578858	0.857000	0.782139
C	-1.455978	2.206743	-2.395811
C	-2.348010	3.257529	-2.102164
C	-2.901358	2.885238	-0.878849
C	-2.593844	-2.572067	-0.464285
C	-3.886330	-2.599222	0.091997
C	-4.039756	-1.324407	0.632898
C	0.815219	0.671476	2.094350
C	0.272399	1.361204	3.194297

C	-1.078862	1.488182	2.870681
H	-3.479910	1.311654	1.398367
Br	-2.701748	4.804879	-3.115822
Br	1.161007	1.974401	4.736342
Br	-5.117335	-4.023633	0.104366
C	2.610371	0.193540	-2.537369
O	2.672281	-0.193938	-3.701211
O	2.959812	1.360194	-2.043769
C	3.498459	2.350296	-2.984561
H	2.700339	2.610966	-3.684265
H	4.317412	1.885783	-3.540810
C	3.951235	3.536736	-2.163950
H	4.356785	4.302969	-2.832055
H	4.732432	3.247437	-1.454997
H	3.114692	3.967795	-1.607941
C	1.980977	-0.808736	-1.706055
H	2.673112	-1.620698	-1.457765
H	-0.202186	3.249762	0.489430
C	0.749218	3.476900	0.005246
H	1.099670	2.601948	-0.542914
H	0.608496	4.313766	-0.683960
Ag	0.101069	-0.603524	-0.908202
H	1.485688	3.751679	0.765054
Br	-0.324855	1.986142	-3.891494
Br	-4.180771	3.823326	0.136412
Br	2.600310	0.146708	1.777549
Br	-2.427632	2.310952	3.893498
Br	-5.544446	-0.646310	1.540526
Br	-1.667253	-3.939284	-1.374234

29-TpBr_3Ag_CHCO2Et_CH4_Ethoxy_TS

Charge	0
Electronic Energy, BS1 (a.u.)	-1308.304372
Thermal and entropic correction, BS1 (a.u.)	0.161845
Electronic Energy, BS2 (a.u.)	-24359.064186
Number of Imaginary Frequencies	1
Imaginary frequencies (cm-1)	-97.2i

Molecular Geometry in Cartesian Coordinates

N	-1.587099	2.160979	-0.629537
N	-2.861483	1.904054	-0.230771
N	-1.772765	-0.900313	-0.342234
N	-2.925255	-0.575284	0.304960
N	-0.862114	0.833070	1.982737
N	-2.193940	1.094096	2.078304
B	-3.144819	0.852433	0.874411
C	-1.660370	3.062926	-1.596295
C	-2.995308	3.426189	-1.860467
C	-3.727032	2.653020	-0.960822
C	-1.876433	-2.169603	-0.712084
C	-3.112901	-2.714202	-0.315469
C	-3.746759	-1.655496	0.331981
C	-0.316650	1.173105	3.140926
C	-1.282783	1.669067	4.036755
C	-2.468705	1.600235	3.307817
H	-4.266508	0.957422	1.237139
Br	-3.641042	4.666124	-3.122993
Br	-1.035951	2.275861	5.802649

Br	-3.761762	-4.459850	-0.597282
C	2.429425	0.737500	-2.342634
O	2.069914	-0.046741	-3.206195
O	2.944130	1.947332	-2.544191
C	3.040238	2.373829	-3.937885
H	2.045831	2.303507	-4.387328
H	3.704274	1.683572	-4.466731
C	3.572510	3.790300	-3.932761
H	3.661117	4.150768	-4.962130
H	4.559692	3.837531	-3.463688
H	2.901145	4.463230	-3.390069
C	2.165371	0.307100	-0.959941
H	2.757694	-0.571083	-0.694345
H	2.755121	1.307237	-0.053219
C	3.842892	1.536495	0.284156
H	4.086811	0.828120	1.073955
H	4.543485	1.507227	-0.544931
Ag	0.178605	0.465758	-0.175792
H	3.701230	2.553045	0.659139
Br	-0.073942	3.675156	-2.419189
Br	-5.598070	2.597299	-0.745907
Br	1.544686	0.971727	3.402889
Br	-4.191676	2.112414	3.871919
Br	-5.451513	-1.658073	1.134068
Br	-0.458751	-2.999089	-1.639837

30-TpBr_3Ag_CHCO2Et_CH4_Oxy_Int-I

Charge	0
Electronic Energy, BS1 (a.u.)	-1308.310203
Thermal and entropic correction, BS1 (a.u.)	0.158947
Electronic Energy, BS2 (a.u.)	-24359.073505
Number of Imaginary Frequencies	0
Imaginary frequencies (cm-1)	None

Molecular Geometry in Cartesian Coordinates

Ag	6.181512	-1.628493	9.929711
Br	6.442905	0.883033	4.163333
Br	-0.278578	-0.910867	10.653731
Br	7.385938	3.160386	13.819635
C	6.688885	-3.567416	9.487962
N	6.089112	-0.168953	8.090379
N	5.347487	0.967866	8.088467
N	3.742512	-0.891436	9.925470
N	3.448857	0.413980	9.682130
N	6.210101	0.402298	11.049098
N	5.554934	1.501757	10.578076
C	6.533042	-0.358322	6.856495
C	6.091541	0.667636	6.000373
C	5.334337	1.490125	6.835170
C	2.609478	-1.485884	10.263914
C	1.529962	-0.579664	10.246678
C	2.119837	0.625014	9.870195
C	6.882062	0.776652	12.130950
C	6.680375	2.141605	12.403334
C	5.826018	2.559854	11.384794
O	5.643483	-5.443403	9.998074
C	5.704875	-4.569230	9.137700
B	4.584389	1.420681	9.364708

H	4.118234	2.490025	9.172986
H	7.710719	-3.919041	9.311374
O	4.956191	-4.416093	8.068160
C	3.884102	-5.390955	7.841097
H	3.407218	-5.610544	8.798584
H	4.344036	-6.306872	7.457957
C	2.926194	-4.765321	6.852406
H	2.112740	-5.466539	6.641246
H	2.495364	-3.846977	7.261144
H	3.432177	-4.527485	5.912263
H	3.102234	1.579237	12.677017
C	3.359793	0.646131	13.182874
H	3.945462	0.869664	14.078309
H	2.441906	0.127706	13.471481
H	3.942517	0.009846	12.515065
Br	2.615661	-3.313561	10.740927
Br	1.281848	2.297863	9.655963
Br	7.593621	-1.870570	6.461818
Br	4.416566	3.065318	6.365666
Br	5.126310	4.288386	11.127679
Br	7.920714	-0.477838	13.078189

31-TpBr_3Ag_CHCO2Et_CH4_Oxy_TS

Charge	0
Electronic Energy, BS1 (a.u.)	-1308.301304
Thermal and entropic correction, BS1 (a.u.)	0.161333
Electronic Energy, BS2 (a.u.)	-24359.062756
Number of Imaginary Frequencies	1
Imaginary frequencies (cm-1)	-182.7i

Molecular Geometry in Cartesian Coordinates

Ag	5.611647	-0.868037	10.513197
Br	7.728401	0.466110	4.746903
Br	-0.304878	-1.415283	8.450685
Br	5.825293	4.759880	13.215298
C	5.905123	-2.906407	10.729347
N	5.737274	-0.116737	8.264363
N	5.257171	1.107286	7.921130
N	3.146689	-0.070127	10.188798
N	3.064765	0.771544	9.121934
N	5.612573	1.403798	10.899609
N	4.848593	2.252433	10.154271
C	6.587977	-0.485963	7.316353
C	6.682448	0.496107	6.312373
C	5.813675	1.497460	6.748055
C	2.092452	-0.870755	10.122558
C	1.292273	-0.579772	8.998667
C	1.962666	0.478895	8.387643
C	6.021338	2.077273	11.967268
C	5.534182	3.396763	11.950311
C	4.789416	3.458441	10.773668
O	4.417276	-4.785031	11.029666
C	4.814883	-3.853993	10.360896
B	4.192289	1.795761	8.823892
H	3.750405	2.736223	8.253751
H	6.889997	-3.357669	10.564480
O	4.342383	-3.467481	9.170579
C	3.284106	-4.271270	8.565990

H	2.440189	-4.294922	9.260217
H	3.655542	-5.292326	8.434393
C	2.932838	-3.605187	7.253561
H	2.122978	-4.157625	6.767000
H	2.597824	-2.578107	7.420984
H	3.795228	-3.586406	6.580648
H	6.033616	-3.146999	12.495387
C	6.373866	-3.920415	13.229123
H	6.626039	-4.851352	12.724340
H	7.238455	-3.499871	13.748399
H	5.531165	-4.074934	13.905866
Br	1.838544	-2.201588	11.438035
Br	1.506811	1.385124	6.800508
Br	7.493087	-2.137461	7.449027
Br	5.438444	3.140911	5.911409
Br	3.837760	4.937516	10.102484
Br	7.099007	1.228120	13.261125

32-TpBrCF3_2Ag_CHCO2Et_CH4_Ethoxy_Int-I

Charge	0
Electronic Energy, BS1 (a.u.)	-3255.095906
Thermal and entropic correction, BS1 (a.u.)	0.241208
Electronic Energy, BS2 (a.u.)	-10940.726413
Number of Imaginary Frequencies	0
Imaginary frequencies (cm-1)	None

Molecular Geometry in Cartesian Coordinates

N	-1.410115	2.148146	-0.605505
N	-2.689516	1.927016	-0.269439
N	-1.952244	-0.867502	-0.571067
N	-2.947344	-0.531065	0.265195
N	-0.780279	0.471559	1.904034
N	-1.996251	1.040571	1.999741
B	-3.035367	0.909204	0.851902
C	-1.415763	2.991112	-1.635982
C	-2.736528	3.328006	-1.994300
C	-3.525307	2.624911	-1.086684
C	-2.119927	-2.148287	-0.897800
C	-3.251493	-2.674749	-0.245497
C	-3.753432	-1.602472	0.490840
C	-0.143314	0.721258	3.045720
C	-0.956155	1.478371	3.912210
C	-2.135817	1.666798	3.200014
H	-4.122621	1.107991	1.262337
C	-1.079480	-2.795674	-1.763626
F	-0.611722	-1.927146	-2.683797
F	-1.548462	-3.874817	-2.407101
C	-0.106800	3.311882	-2.284643
F	0.897377	3.299495	-1.386007
F	-0.099803	4.508625	-2.895441
C	1.240909	0.203450	3.292792
F	1.710973	-0.485810	2.227028
F	1.272390	-0.625035	4.354722
F	2.107041	1.207437	3.541318
F	-0.015507	-3.188798	-1.018689
F	0.202378	2.378748	-3.229980
C	-3.402107	2.390207	3.560493
C	-4.940397	-1.499655	1.407247

C	-5.014981	2.551030	-0.914028
Br	-3.275011	4.476018	-3.397946
Br	-0.494858	2.054860	5.652320
Br	-3.913716	-4.442513	-0.362201
F	-3.258502	3.087725	4.698182
F	-3.752313	3.260375	2.586671
F	-4.431304	1.535595	3.727859
F	-5.396017	3.034219	0.285915
F	-5.656953	3.251397	-1.861467
F	-5.442603	1.270320	-0.988737
F	-4.569440	-1.020583	2.616169
F	-5.515432	-2.695955	1.602644
F	-5.878415	-0.667351	0.913296
C	2.520123	0.606933	-2.431057
O	2.214086	-0.248773	-3.266213
O	3.037867	1.788916	-2.604119
C	3.197903	2.257623	-3.992364
H	2.304775	1.971196	-4.550594
H	4.066338	1.744165	-4.414067
C	3.381189	3.756932	-3.928102
H	3.538419	4.142429	-4.940266
H	4.250478	4.020337	-3.318926
H	2.495841	4.239448	-3.507705
C	2.101698	0.013697	-1.191538
H	2.853760	-0.681273	-0.802589
H	2.007158	2.719622	0.942244
C	3.028796	2.355179	0.821623
H	3.050985	1.274538	0.982392
H	3.383798	2.600093	-0.181019
Ag	0.184521	0.185659	-0.378905
H	3.675336	2.826995	1.566255

33-TpBrCF3_2Ag_CHCO2Et_CH4_Ethoxy_TS

Charge	0
Electronic Energy, BS1 (a.u.)	-3255.093679
Thermal and entropic correction, BS1 (a.u.)	0.242377
Electronic Energy, BS2 (a.u.)	-10940.723535
Number of Imaginary Frequencies	1
Imaginary frequencies (cm-1)	-141.2i

Molecular Geometry in Cartesian Coordinates

N	-1.486677	2.177418	-0.530645
N	-2.770096	1.918925	-0.239716
N	-1.974263	-0.862995	-0.549279
N	-2.987630	-0.550326	0.274970
N	-0.871383	0.520974	1.943779
N	-2.107757	1.041024	2.032571
B	-3.121939	0.884011	0.866383
C	-1.479144	3.041763	-1.542503
C	-2.795690	3.355116	-1.936045
C	-3.595919	2.613797	-1.069424
C	-2.111351	-2.145609	-0.883149
C	-3.240669	-2.697795	-0.248698
C	-3.774640	-1.639061	0.484360
C	-0.224842	0.863544	3.054833
C	-1.053517	1.634432	3.894775
C	-2.254938	1.726387	3.199834
H	-4.222522	1.058740	1.252134

C	-1.046196	-2.768015	-1.737310
F	-0.585715	-1.887367	-2.649211
F	-1.483511	-3.855484	-2.388933
C	-0.157873	3.401108	-2.146165
F	0.828968	3.345768	-1.227201
F	-0.144656	4.629844	-2.690362
C	1.207347	0.475046	3.254148
F	1.626840	-0.383152	2.296360
F	1.407322	-0.114127	4.446363
F	2.019872	1.557748	3.199510
F	0.016232	-3.139891	-0.979246
F	0.172548	2.522338	-3.131837
C	-3.549347	2.404911	3.544902
C	-4.981475	-1.562069	1.377133
C	-5.087769	2.502407	-0.943952
Br	-3.314183	4.521504	-3.331876
Br	-0.579006	2.350878	5.578991
Br	-3.864576	-4.478365	-0.380497
F	-3.442619	3.117055	4.677525
F	-3.926327	3.252570	2.560877
F	-4.545455	1.511511	3.711204
F	-5.518684	2.974252	0.243662
F	-5.716802	3.187679	-1.911039
F	-5.480653	1.211390	-1.032200
F	-4.647735	-1.069197	2.591253
F	-5.530802	-2.771480	1.566197
F	-5.929411	-0.755235	0.859890
C	2.505404	0.665510	-2.371513
O	2.234879	-0.149855	-3.243886
O	2.991277	1.880879	-2.524506
C	3.142921	2.362101	-3.899864
H	2.252055	2.077951	-4.463208
H	4.011409	1.860842	-4.337675
C	3.319108	3.862899	-3.823335
H	3.469817	4.261758	-4.831349
H	4.189095	4.125812	-3.214476
H	2.433067	4.336433	-3.393533
C	2.134010	0.166380	-1.048272
H	2.825027	-0.604360	-0.696012
H	2.635728	1.330984	0.001453
C	3.604936	1.668829	0.474778
H	3.767838	1.052333	1.356637
H	4.425165	1.602345	-0.236614
Ag	0.127135	0.236356	-0.360396
H	3.399515	2.708693	0.738673

34-TpBrCF3_2Ag_CHCO2Et_CH4_Oxy_Int-I

Charge	0
Electronic Energy, BS1 (a.u.)	-3255.092858
Thermal and entropic correction, BS1 (a.u.)	0.239259
Electronic Energy, BS2 (a.u.)	-10940.725000
Number of Imaginary Frequencies	0
Imaginary frequencies (cm-1)	None

Molecular Geometry in Cartesian Coordinates

Ag	5.258537	-0.990524	9.949817
Br	8.658666	1.575374	5.484330
Br	-0.582801	-0.100558	7.419718

Br	5.166817	3.612287	14.138037
F	7.882623	-1.505213	5.868361
F	6.220870	-2.206852	7.091641
F	8.073698	-1.585471	8.036985
F	7.357664	4.228560	6.362611
F	6.357421	4.358948	8.289727
F	5.186627	4.203044	6.461409
F	0.256817	-1.654026	10.410295
F	2.356624	-2.197656	10.597982
F	1.244525	-2.722607	8.799063
F	0.676805	2.488771	6.259212
F	2.836295	2.753496	6.229967
F	1.637105	3.745315	7.751699
F	5.551213	-0.599693	13.234068
F	6.819178	0.933441	14.122624
F	7.423664	-0.051994	12.275749
F	3.528935	5.161268	12.050284
F	2.559272	4.131969	10.398801
F	4.429675	5.197111	10.073137
C	5.626323	-3.033306	9.980048
N	5.648501	0.305461	7.932765
N	5.424263	1.627183	7.967541
N	2.960483	0.129120	9.430070
N	3.032001	1.268042	8.719526
N	5.407421	1.184965	10.861454
N	4.725533	2.214395	10.333032
C	6.697001	0.098270	7.137938
C	7.183897	1.323396	6.641116
C	6.336100	2.280195	7.197619
C	7.224338	-1.299761	7.016760
C	6.311073	3.777086	7.070190
C	1.811918	-0.456532	9.106476
C	1.107172	0.314270	8.159221
C	1.928353	1.413522	7.933813
C	1.415317	-1.756184	9.732331
C	1.762448	2.607183	7.039233
C	5.651662	1.478721	12.138415
C	5.102761	2.733345	12.464588
C	4.515885	3.169629	11.276948
C	6.369137	0.447452	12.959004
C	3.757449	4.426804	10.951333
O	4.632934	-4.382015	11.284984
C	4.610071	-4.041063	10.100761
B	4.269395	2.197058	8.843031
H	4.001959	3.289652	8.490588
H	6.626706	-3.464925	9.883408
O	3.810006	-4.420322	9.142032
C	2.731459	-5.354278	9.509468
H	2.052260	-4.816661	10.174118
H	3.174866	-6.192867	10.052668
C	2.064863	-5.783265	8.223202
H	1.244099	-6.468974	8.455413
H	1.656246	-4.919883	7.693419
H	2.772299	-6.299663	7.568188
H	6.828213	-2.776697	12.742310
C	7.845260	-3.098656	12.516790
H	7.861324	-4.179484	12.352942
H	8.205576	-2.579635	11.625465
H	8.498602	-2.851277	13.357684

35-TpBrCF3_2Ag_CHCO2Et_CH4_Oxy_TS

Charge	0
Electronic Energy, BS1 (a.u.)	-3255.088058
Thermal and entropic correction, BS1 (a.u.)	0.240522
Electronic Energy, BS2 (a.u.)	-10940.720080
Number of Imaginary Frequencies	1
Imaginary frequencies (cm-1)	-149.1i

Molecular Geometry in Cartesian Coordinates

Ag	5.265210	-0.986531	9.971501
Br	8.491101	1.441895	5.302748
Br	-0.687995	-0.152984	7.770547
Br	5.417763	3.709503	14.032766
F	7.762347	-1.625865	5.830963
F	6.148263	-2.294365	7.133841
F	8.026676	-1.627391	7.993744
F	7.201698	4.116169	6.130699
F	6.292079	4.310261	8.096921
F	5.037838	4.082710	6.332532
F	0.355534	-1.622228	10.754458
F	2.450873	-2.212090	10.771415
F	1.174994	-2.745801	9.086990
F	0.498509	2.400872	6.471164
F	2.651105	2.665610	6.299150
F	1.549226	3.688785	7.872961
F	5.734212	-0.464103	13.289259
F	7.143492	1.060226	13.950370
F	7.524341	-0.068098	12.125277
F	3.679987	5.218157	11.990580
F	2.615675	4.140262	10.430403
F	4.460957	5.196186	9.962705
C	5.759636	-2.983729	10.238430
N	5.588719	0.241999	7.911130
N	5.357622	1.563493	7.909636
N	2.983452	0.100805	9.530936
N	3.009225	1.227422	8.796384
N	5.500413	1.215518	10.800657
N	4.785977	2.228576	10.286175
C	6.606295	0.012690	7.083056
C	7.064702	1.222262	6.524807
C	6.233486	2.193459	7.081074
C	7.139719	-1.385564	6.992782
C	6.192642	3.684823	6.902186
C	1.814325	-0.487579	9.296801
C	1.049271	0.268427	8.385522
C	1.855463	1.360746	8.084602
C	1.448661	-1.767229	9.982268
C	1.631186	2.536136	7.177905
C	5.811816	1.538797	12.055290
C	5.273764	2.798386	12.381818
C	4.622776	3.205389	11.217287
C	6.561308	0.525641	12.869595
C	3.843613	4.452127	10.901730
O	4.495644	-4.540440	11.534433
C	4.754501	-4.048971	10.455706
B	4.248425	2.162062	8.824054
H	3.958283	3.242206	8.451988
H	6.727533	-3.377493	9.910364
O	4.172570	-4.319220	9.286636

C	3.044186	-5.253417	9.308142
H	2.289776	-4.853476	9.988706
H	3.400150	-6.210168	9.700627
C	2.537412	-5.360925	7.887685
H	1.682838	-6.044490	7.858191
H	2.211868	-4.384580	7.521096
H	3.313773	-5.748543	7.221610
H	6.434522	-2.791225	11.983094
C	7.200771	-3.271564	12.626705
H	7.374089	-4.302753	12.319605
H	8.112509	-2.676633	12.554842
H	6.787123	-3.244536	13.637024

36-TpF27_Ag_CHCO2Et_CH4_Ethoxy_Int-I

Charge	0
Electronic Energy, BS1 (a.u.)	-4571.29961952
Thermal and entropic correction, BS1 (a.u.)	0.3279885199999626
Electronic Energy, BS2 (a.u.)	-4574.505669
Number of Imaginary Frequencies	0
Imaginary frequencies (cm-1)	None

Molecular Geometry in Cartesian Coordinates

F	7.241396	-1.421242	5.301805
F	6.041930	-2.179595	6.998649
F	0.258445	-1.860550	10.301701
F	2.342237	-1.974254	11.023269
F	6.957317	0.872974	14.038218
F	6.945059	-0.556175	12.357570
N	5.588720	0.327983	7.826547
N	5.382285	1.658374	7.930325
N	3.017196	0.134370	9.515730
N	3.048084	1.294967	8.825537
N	5.436727	1.165097	10.883148
N	4.825058	2.243437	10.339724
C	6.545535	0.116024	6.935541
C	7.027589	1.349190	6.420791
C	6.243038	2.322648	7.097450
C	6.997573	-1.275683	6.632084
C	1.858062	-0.462793	9.289156
C	1.070192	0.303881	8.388879
C	1.885104	1.436375	8.114790
C	1.562734	-1.786147	9.917882
C	5.611454	1.382358	12.178228
C	5.084516	2.650454	12.543649
C	4.587628	3.173229	11.318570
C	6.182143	0.320232	13.066426
B	4.294642	2.222404	8.880219
H	3.998260	3.316163	8.543789
C	8.009643	1.743064	5.496834
C	8.191115	3.088350	5.260727
C	7.410114	4.054409	5.939875
C	6.440851	3.693848	6.854526
C	3.978692	4.441814	11.281265
C	4.962056	3.385915	13.734997
C	4.363653	4.625840	13.686679
C	3.877318	5.146136	12.464336
C	-0.190979	0.170809	7.785915
C	1.439268	2.438890	7.235015

C	0.194088	2.286360	6.657979
C	-0.620814	1.161164	6.929640
F	-0.961277	-0.899141	8.023230
F	-1.813651	1.078974	6.325538
F	-0.268293	3.210187	5.808056
F	2.187477	3.509122	6.931285
F	5.401690	2.899335	14.902088
F	4.220142	5.364098	14.795008
F	3.299271	6.352276	12.476816
F	3.487976	4.975268	10.154111
F	8.764684	0.837686	4.861485
F	9.116778	3.515928	4.392183
F	7.640262	5.346511	5.681727
F	5.740861	4.642169	7.492733
C	8.294656	-1.686132	7.368746
F	9.305714	-0.890796	6.998575
F	8.127136	-1.577159	8.702085
F	8.612570	-2.958962	7.089984
C	1.815102	-2.988498	8.977433
F	1.063285	-2.866415	7.874466
F	3.112351	-3.020126	8.608441
F	1.516888	-4.144294	9.581386
C	5.086875	-0.506139	13.789128
F	4.340244	0.306850	14.552931
F	4.273274	-1.097220	12.886208
F	5.624431	-1.454964	14.558659
H	6.498331	-4.569341	7.570418
H	5.463280	-5.916857	7.046024
C	5.720968	-5.267133	7.886628
H	6.081223	-5.883771	8.714748
H	4.827701	-4.716576	8.192452
C	5.775396	-2.985184	10.307224
H	6.597923	-3.537132	9.848568
C	5.181132	-3.669162	11.414809
O	5.984478	-3.458016	12.330620
O	4.007230	-4.231751	11.457857
C	3.481483	-4.542265	12.800195
H	4.073070	-5.365651	13.209921
H	3.627940	-3.655948	13.422662
C	2.022635	-4.900610	12.632149
H	1.603618	-5.154451	13.610836
H	1.904257	-5.762892	11.970483
H	1.463681	-4.059911	12.217772
Ag	5.231435	-1.015744	9.861050

37-TpF27Ag_CHCO2Et_CH4_Ethoxy_TS

Charge	0
Electronic Energy, BS1 (a.u.)	-4571.298194
Thermal and entropic correction, BS1 (a.u.)	0.328898
Electronic Energy, BS2 (a.u.)	-4574.503337
Number of Imaginary Frequencies	1
Imaginary frequencies (cm-1)	-135.7i

Molecular Geometry in Cartesian Coordinates

F	7.260040	-1.478318	5.269981
F	6.037880	-2.234377	6.950495
F	0.310792	-1.983774	10.250003
F	2.427783	-2.145292	10.851406

F	6.921526	0.891578	14.032937
F	6.897362	-0.572954	12.383831
N	5.593771	0.265774	7.789037
N	5.389504	1.595815	7.895104
N	3.038050	0.058018	9.434355
N	3.047139	1.248463	8.795743
N	5.419196	1.136250	10.863522
N	4.829310	2.213549	10.294581
C	6.558559	0.055528	6.906116
C	7.049827	1.289074	6.401250
C	6.261414	2.261823	7.074476
C	7.004184	-1.336624	6.598877
C	1.857696	-0.511405	9.254268
C	1.029040	0.306436	8.439684
C	1.843244	1.440386	8.168563
C	1.592136	-1.875054	9.805097
C	5.597700	1.382197	12.152705
C	5.095851	2.669058	12.487489
C	4.610696	3.172291	11.249752
C	6.143342	0.330631	13.068043
B	4.298968	2.168668	8.836716
H	4.004008	3.257389	8.485667
C	8.041973	1.683658	5.488631
C	8.230783	3.029301	5.260543
C	7.447091	3.994874	5.937064
C	6.467604	3.633488	6.840520
C	4.028684	4.452316	11.180849
C	4.985717	3.434653	13.660965
C	4.413023	4.685151	13.581989
C	3.939945	5.186400	12.346702
C	-0.268537	0.216093	7.910417
C	1.355587	2.490545	7.369286
C	0.074580	2.379884	6.865630
C	-0.737138	1.251471	7.131424
F	-1.037443	-0.855927	8.142811
F	-1.965951	1.211593	6.599448
F	-0.427414	3.351040	6.094546
F	2.092997	3.569551	7.071496
F	5.412096	2.967474	14.840736
F	4.282354	5.452081	14.672510
F	3.387539	6.404623	12.329164
F	3.551547	4.970194	10.040445
F	8.799627	0.778177	4.855979
F	9.166245	3.457412	4.402688
F	7.684941	5.287473	5.688007
F	5.766017	4.582241	7.476579
C	8.292486	-1.760666	7.343844
F	9.321290	-0.999910	6.952899
F	8.133037	-1.615233	8.675255
F	8.578905	-3.048259	7.097108
C	1.799858	-2.996725	8.760397
F	0.951355	-2.824053	7.736766
F	3.059111	-2.951704	8.283332
F	1.592077	-4.205196	9.297539
C	5.025403	-0.456817	13.798435
F	4.272891	0.396057	14.514294
F	4.224533	-1.069538	12.900339
F	5.535477	-1.376572	14.617981
H	6.524507	-4.627595	7.946263
H	4.773241	-4.941597	7.696501
C	5.564825	-4.794009	8.434209
H	5.606019	-5.632423	9.128965

H	5.264949	-3.861941	8.970676
C	5.938085	-2.998821	10.383285
H	6.946773	-3.350475	10.163420
C	5.336412	-3.682156	11.516121
O	5.936469	-3.399528	12.548634
O	4.207144	-4.348037	11.396001
C	3.532104	-4.697070	12.651126
H	4.127202	-5.464487	13.155260
H	3.512243	-3.802952	13.279769
C	2.145187	-5.182657	12.293436
H	1.625290	-5.487622	13.207066
H	2.192362	-6.042594	11.619248
H	1.570699	-4.390357	11.811092
Ag	5.226043	-1.078382	9.849665

38-TpF27_Ag_CHCO2Et_CH4_Oxy_Int-I

Charge	0
Electronic Energy, BS1 (a.u.)	-4571.298336
Thermal and entropic correction, BS1 (a.u.)	0.327445
Electronic Energy, BS2 (a.u.)	-4574.504153
Number of Imaginary Frequencies	0
Imaginary frequencies (cm-1)	None

Molecular Geometry in Cartesian Coordinates

Ag	4.920583	-0.975664	9.854019
F	6.956382	-1.417859	5.210131
F	5.605464	-2.130757	6.809640
F	0.125061	-1.931524	9.570374
F	2.144427	-2.133765	10.441978
F	6.993076	1.040709	13.944391
F	6.855672	-0.459382	12.327404
C	5.672892	-2.861562	10.324288
N	5.440917	0.333494	7.801829
N	5.351891	1.671912	7.953101
N	2.890303	0.205225	9.296182
N	2.963127	1.430566	8.726058
N	5.337303	1.253198	10.866062
N	4.719917	2.323680	10.318197
C	6.413871	0.066134	6.943604
C	7.032642	1.267401	6.505583
C	6.310993	2.283119	7.190675
C	6.689201	-1.348312	6.544539
C	1.764374	-0.377728	8.913391
C	1.040392	0.472633	8.035074
C	1.856536	1.633322	7.942801
C	1.454102	-1.784871	9.321219
C	5.550361	1.501069	12.149136
C	5.046016	2.783172	12.499721
C	4.516217	3.279682	11.278433
C	6.150229	0.465538	13.045672
O	4.339621	-4.487435	10.377210
C	5.224594	-4.037617	9.642401
B	4.245886	2.303107	8.845513
H	4.018027	3.404290	8.480727
H	6.395180	-3.073781	11.115447
O	5.622555	-4.435818	8.467732
C	4.846709	-5.487530	7.791032
H	3.818098	-5.448107	8.155040

H	5.291892	-6.444656	8.075614
C	4.948502	-5.214222	6.306272
H	4.459665	-6.023355	5.754790
H	4.459950	-4.270480	6.055584
H	5.994715	-5.158328	5.993350
H	5.350904	-3.689787	13.292673
C	6.388102	-3.829949	13.604869
H	6.818556	-4.686892	13.079159
H	6.962786	-2.926431	13.390793
H	6.417267	-4.019828	14.680536
C	8.088391	1.604485	5.642900
C	8.405223	2.935267	5.476742
C	7.686945	3.942845	6.165498
C	6.646559	3.637798	7.020200
C	3.908400	4.547583	11.227445
C	4.965791	3.549184	13.674581
C	4.372757	4.791339	13.613400
C	3.848095	5.282801	12.394449
C	-0.167555	0.390462	7.322287
C	1.460160	2.718434	7.138870
C	0.267302	2.613378	6.451407
C	-0.546009	1.458955	6.539448
F	-0.937981	-0.702827	7.385615
F	-1.686110	1.428063	5.837269
F	-0.144207	3.617308	5.669276
F	2.200392	3.827919	7.008134
F	5.441027	3.087459	14.838217
F	4.268807	5.558767	14.706316
F	3.273023	6.490548	12.395832
F	3.377867	5.046426	10.102122
F	8.784421	0.657607	5.000160
F	9.406881	3.308629	4.669523
F	8.049637	5.216537	5.977794
F	6.006905	4.618138	7.673929
C	7.895030	-1.998484	7.261402
F	7.698171	-2.007449	8.599280
F	8.067469	-3.260565	6.853379
F	9.008360	-1.298190	7.007908
C	1.817219	-2.826350	8.231144
F	3.132394	-2.748579	7.928138
F	1.560118	-4.070877	8.650227
F	1.116511	-2.586528	7.114660
C	5.087101	-0.316036	13.857250
F	4.287173	0.539934	14.510897
F	4.333669	-1.058355	13.023977
F	5.668017	-1.127000	14.749106

39-TpF27_Ag_CHCO2Et_CH4_Oxy_TS

Charge	0
Electronic Energy, BS1 (a.u.)	-4571.292875
Thermal and entropic correction, BS1 (a.u.)	0.329533
Electronic Energy, BS2 (a.u.)	-4574.498608
Number of Imaginary Frequencies	1
Imaginary frequencies (cm-1)	-154.1i

Molecular Geometry in Cartesian Coordinates

Ag	5.089544	-1.032512	9.891290
F	7.025836	-1.450922	5.290467

F	5.800753	-2.177677	6.981508
F	0.241899	-2.052298	9.679667
F	2.293283	-2.232421	10.481187
F	6.977658	0.984745	14.048010
F	6.776242	-0.537878	12.461312
C	5.773247	-2.958860	10.321460
N	5.552883	0.309132	7.907869
N	5.386617	1.645281	8.009763
N	2.962834	0.129585	9.357248
N	3.005131	1.362663	8.802277
N	5.327272	1.202655	10.956224
N	4.758919	2.285768	10.381056
C	6.483252	0.063424	6.996785
C	6.994270	1.278736	6.471235
C	6.253352	2.278382	7.159363
C	6.822503	-1.346128	6.632574
C	1.834545	-0.460130	8.996866
C	1.075782	0.392824	8.149952
C	1.874661	1.564774	8.051890
C	1.559370	-1.877660	9.390118
C	5.576352	1.482725	12.226034
C	5.145287	2.799921	12.540684
C	4.624323	3.284030	11.310348
C	6.114942	0.439546	13.150442
O	4.181150	-4.729990	10.400810
C	5.093628	-4.174139	9.825724
B	4.273604	2.251227	8.912831
H	4.027036	3.346483	8.546673
H	6.791641	-3.157694	10.669041
O	5.614892	-4.487473	8.638108
C	4.909643	-5.477161	7.820495
H	3.845027	-5.430182	8.057133
H	5.289441	-6.460915	8.113407
C	5.197255	-5.157183	6.369356
H	4.785557	-5.949449	5.735850
H	4.740633	-4.207443	6.084764
H	6.274198	-5.091131	6.190708
H	5.238527	-3.058281	12.207455
C	5.676622	-3.669039	13.020878
H	6.004211	-4.638680	12.646339
H	6.505413	-3.114402	13.460596
H	4.871402	-3.806868	13.746030
C	7.974098	1.637546	5.530696
C	8.194280	2.975408	5.286738
C	7.455788	3.968101	5.975827
C	6.491552	3.642068	6.908471
C	4.090005	4.583527	11.225188
C	5.121838	3.605536	13.691620
C	4.597171	4.875813	13.597384
C	4.085856	5.356920	12.368828
C	-0.150238	0.304977	7.470252
C	1.442241	2.655655	7.275136
C	0.231701	2.545180	6.619754
C	-0.564180	1.379405	6.713535
F	-0.904766	-0.798968	7.540370
F	-1.723537	1.343862	6.043491
F	-0.214361	3.555508	5.864818
F	2.163899	3.777434	7.140931
F	5.585160	3.155519	14.864524
F	4.549307	5.681665	14.666108
F	3.581380	6.595253	12.336716
F	3.577914	5.080913	10.091103

F	8.691076	0.705321	4.890206
F	9.119869	3.371098	4.403527
F	7.724739	5.250998	5.710988
F	5.836691	4.614686	7.557762
C	8.094842	-1.895958	7.315371
F	7.954445	-1.854218	8.658384
F	8.319806	-3.164294	6.954259
F	9.153840	-1.148111	6.978625
C	1.907377	-2.888851	8.268547
F	3.218040	-2.793957	7.951024
F	1.659108	-4.144273	8.655332
F	1.189984	-2.620307	7.168187
C	4.996933	-0.260952	13.959260
F	4.293575	0.647036	14.650677
F	4.163553	-0.901321	13.116693
F	5.510355	-1.152904	14.816052

40-TpBr_3Ag_C-Ph-CO2Et_CH4_Ethoxy_Int-I

Charge	0
Electronic Energy, BS1 (a.u.)	-1539.416524
Thermal and entropic correction, BS1 (a.u.)	0.233645
Electronic Energy, BS2 (a.u.)	-24590.253526
Number of Imaginary Frequencies	0
Imaginary frequencies (cm-1)	None

Molecular Geometry in Cartesian Coordinates

N	-2.387476	2.006629	-1.070874
N	-3.404823	1.484282	-0.326701
N	-2.142554	-1.000026	-0.895604
N	-2.633011	-0.837076	0.363705
N	-0.674147	0.914715	1.406215
N	-1.973672	1.162260	1.728298
B	-3.114088	0.543604	0.876227
C	-2.936079	2.822692	-1.965766
C	-4.334506	2.855995	-1.829018
C	-4.583731	1.983034	-0.770445
C	-1.714507	-2.254952	-0.970194
C	-1.904326	-2.941064	0.247431
C	-2.495447	-1.984252	1.070600
C	0.063329	1.666421	2.214724
C	-0.738941	2.424880	3.090984
C	-2.037994	2.068371	2.733153
H	-4.096173	0.430152	1.536783
Br	-5.577974	3.850669	-2.832874
Br	-0.193750	3.627619	4.435018
Br	-1.472868	-4.726766	0.669889
C	2.281141	0.947853	-2.737449
O	2.212026	0.579567	-3.897584
O	2.939492	2.023632	-2.293587
C	3.616106	2.840730	-3.293530
H	2.862408	3.237666	-3.980798
H	4.296357	2.202774	-3.866053
C	4.346880	3.935223	-2.545937
H	4.867017	4.582673	-3.258977
H	5.085612	3.511760	-1.858832
H	3.646713	4.546703	-1.969328
C	1.557633	0.249505	-1.664919
H	4.320021	1.387274	-0.283224

C	5.109000	1.080643	0.404988
H	4.762456	0.248069	1.022247
H	5.988331	0.762958	-0.162614
Ag	-0.371041	1.001237	-1.238136
H	5.383262	1.922362	1.047166
Br	-1.850608	3.736198	-3.208104
Br	-6.255830	1.526642	-0.037884
Br	1.948472	1.646698	2.064215
Br	-3.662335	2.702224	3.445608
Br	-3.012743	-2.140701	2.875217
Br	-0.921269	-2.893711	-2.561541
C	2.162596	-0.873942	-1.057645
C	1.508897	-1.507736	0.041216
C	3.426171	-1.385161	-1.486787
C	2.094856	-2.586965	0.682243
C	3.992215	-2.476953	-0.855245
C	3.328491	-3.073167	0.230078
H	0.561264	-1.110246	0.386255
H	3.928795	-0.922620	-2.329982
H	1.598021	-3.056676	1.524129
H	4.946266	-2.870678	-1.189857
H	3.781792	-3.925835	0.727796

41-TpBr_3Ag_C-Ph-CO2Et_CH4_Ethoxy_TS

Charge	0
Electronic Energy, BS1 (a.u.)	-1539.387639
Thermal and entropic correction, BS1 (a.u.)	0.239003
Electronic Energy, BS2 (a.u.)	-24590.222561
Number of Imaginary Frequencies	1
Imaginary frequencies (cm-1)	-554.2i

Molecular Geometry in Cartesian Coordinates

N	-1.660170	2.260810	-0.784021
N	-2.841582	1.731645	-0.358337
N	-1.505745	-0.755453	-0.855228
N	-2.343340	-0.694775	0.217721
N	-0.591880	0.877210	1.854770
N	-1.931625	1.116634	1.914684
B	-2.866504	0.655803	0.762296
C	-1.949906	3.173316	-1.703992
C	-3.338732	3.270584	-1.902341
C	-3.863848	2.326721	-1.019822
C	-1.185152	-2.035815	-1.013912
C	-1.796690	-2.842908	-0.033880
C	-2.524353	-1.934346	0.731951
C	-0.061739	1.464331	2.920006
C	-1.039975	2.103373	3.706475
C	-2.222874	1.854133	3.013411
H	-3.975286	0.535995	1.171556
Br	-4.270524	4.411533	-3.074728
Br	-0.814850	3.055408	5.316405
Br	-1.664431	-4.708527	0.194729
C	2.705274	0.850357	-2.327108
O	2.548768	0.155890	-3.315459
O	3.045233	2.156264	-2.371019
C	3.107080	2.749954	-3.693641

H	2.136948	2.613642	-4.181881
H	3.859202	2.220367	-4.286820
C	3.452828	4.212763	-3.509316
H	3.499662	4.707236	-4.484583
H	4.423825	4.327882	-3.017912
H	2.697772	4.719596	-2.900107
C	2.406986	0.368267	-0.955120
H	2.705260	1.243872	-0.100136
C	3.941710	1.409126	0.169645
H	4.033123	0.825208	1.082135
H	4.688870	1.164637	-0.575114
Ag	0.284752	1.034047	-0.618108
H	3.841411	2.485587	0.320246
Br	-0.559851	4.120247	-2.560355
Br	-5.675993	1.899568	-0.744735
Br	1.804762	1.386829	3.227881
Br	-3.964698	2.425939	3.444744
Br	-3.578715	-2.265900	2.257984
Br	-0.029918	-2.548222	-2.411266
C	2.707909	-1.020690	-0.557808
C	2.044967	-1.582740	0.550950
C	3.632168	-1.811793	-1.266504
C	2.293628	-2.897235	0.936138
C	3.865926	-3.131483	-0.889273
C	3.200601	-3.676484	0.212983
H	1.320831	-0.988773	1.100250
H	4.137366	-1.400404	-2.132903
H	1.766879	-3.317328	1.787335
H	4.568222	-3.736809	-1.454645
H	3.384353	-4.706712	0.503665

42-TpBr_3Ag_C-Ph-CO2Et_CH4_Oxy_Int-I

Charge	0
Electronic Energy, BS1 (a.u.)	-1539.419921
Thermal and entropic correction, BS1 (a.u.)	0.233777
Electronic Energy, BS2 (a.u.)	-24590.257706
Number of Imaginary Frequencies	0
Imaginary frequencies (cm-1)	None

Molecular Geometry in Cartesian Coordinates

Ag	5.765562	-1.675738	9.917774
Br	6.645539	1.014928	4.197562
Br	-0.544926	-0.583856	10.505277
Br	7.250185	3.250307	13.708637
C	6.472825	-3.666607	9.986876
N	5.848084	-0.185224	8.020313
N	5.201484	1.011221	8.008625
N	3.490565	-0.739897	9.890944
N	3.255116	0.564174	9.587640
N	6.013091	0.491422	10.961153
N	5.387846	1.600881	10.480847
C	6.391112	-0.348646	6.822885
C	6.111257	0.747633	5.984196
C	5.346777	1.590358	6.788480
C	2.327058	-1.280909	10.219277
C	1.285273	-0.336372	10.136074
C	1.930634	0.829728	9.729209
C	6.688235	0.860886	12.039922

C	6.522217	2.234179	12.300259
C	5.680887	2.662679	11.275354
O	4.989494	-5.388353	10.599660
C	5.416632	-4.659159	9.718778
B	4.432485	1.518616	9.256977
H	4.004137	2.597454	9.031778
O	4.961828	-4.588811	8.465490
C	3.825848	-5.428737	8.111311
H	3.120454	-5.435678	8.945078
H	4.190584	-6.450534	7.960004
C	3.217565	-4.842966	6.855136
H	2.362387	-5.450614	6.542207
H	2.869892	-3.821920	7.037972
H	3.945235	-4.821152	6.038368
H	2.924588	1.744717	12.546468
C	3.166027	0.828663	13.090050
H	3.755261	0.165135	12.455415
H	3.738206	1.080565	13.986715
H	2.239500	0.327240	13.381332
Br	2.263872	-3.088267	10.755393
Br	1.158706	2.520382	9.421359
Br	7.387639	-1.905508	6.438847
Br	4.609211	3.259707	6.318879
Br	5.020659	4.404241	10.994383
Br	7.680163	-0.416025	13.014602
C	7.808326	-4.129074	10.120595
C	8.847167	-3.181145	10.349540
C	8.162470	-5.506403	9.998600
C	10.170737	-3.583867	10.429670
C	9.484803	-5.903445	10.095886
C	10.486767	-4.943319	10.305638
H	8.576889	-2.135309	10.451886
H	7.381786	-6.244435	9.847284
H	10.956529	-2.853784	10.593233
H	9.749307	-6.952186	10.007495
H	11.523474	-5.260462	10.375667

43-TpBr_3Ag_C-Ph-CO2Et_CH4_Oxy_TS

Charge	0
Electronic Energy, BS1 (a.u.)	-1539.389086
Thermal and entropic correction, BS1 (a.u.)	0.238650
Electronic Energy, BS2 (a.u.)	-24590.223243
Number of Imaginary Frequencies	1
Imaginary frequencies (cm-1)	-460.6i

Molecular Geometry in Cartesian Coordinates

Ag	5.205542	-1.260187	10.348148
Br	7.949761	0.280973	4.815758
Br	-0.830594	-0.413689	8.660730
Br	6.109740	4.279595	13.296489
C	5.920994	-3.283341	11.058073
N	5.562820	-0.393590	8.066169
N	5.273204	0.914698	7.820726
N	3.067814	-0.202574	9.869130
N	3.028774	0.847408	9.004697
N	5.673052	1.090298	10.775956
N	4.993094	2.041506	10.079042
C	6.449832	-0.770204	7.154637

C	6.764378	0.289388	6.280564
C	5.987955	1.345883	6.750557
C	1.855574	-0.741752	9.881067
C	0.984967	-0.055676	9.012615
C	1.784037	0.952030	8.474717
C	6.111001	1.666157	11.886094
C	5.731938	3.020252	11.947731
C	5.014939	3.210809	10.767632
O	3.942399	-4.615518	11.465339
C	4.787589	-4.182363	10.704236
B	4.296203	1.700578	8.731964
H	3.980608	2.706839	8.193077
O	4.792178	-4.353814	9.365223
C	3.560170	-4.764716	8.711064
H	2.786697	-4.907407	9.467779
H	3.768566	-5.726606	8.232836
C	3.188680	-3.697478	7.698117
H	2.261156	-3.976651	7.186932
H	3.035270	-2.733850	8.191573
H	3.977860	-3.575232	6.951151
H	5.762649	-2.906534	12.250539
C	6.006025	-3.712673	13.190176
H	6.041272	-4.773706	12.977627
H	6.952143	-3.302937	13.541904
H	5.133579	-3.439681	13.785609
Br	1.531282	-2.260807	10.949238
Br	1.291178	2.261925	7.213437
Br	7.132262	-2.527815	7.171002
Br	5.903484	3.102566	6.072681
Br	4.185850	4.794304	10.172687
Br	7.094062	0.659338	13.149314
C	7.313563	-3.597439	10.685146
C	8.311917	-2.612845	10.817991
C	7.671959	-4.855980	10.167099
C	9.624910	-2.874273	10.439766
C	8.981369	-5.106666	9.761106
C	9.961289	-4.119991	9.900789
H	8.042601	-1.635135	11.207412
H	6.916998	-5.626715	10.053552
H	10.380897	-2.102142	10.545245
H	9.239758	-6.075663	9.344416
H	10.982205	-4.319691	9.588951

44-TpBrCF3_2Ag_C-Ph-CO2Et_CH4_Ethoxy_Int-I

Charge	0
Electronic Energy, BS1 (a.u.)	-3486.204745
Thermal and entropic correction, BS1 (a.u.)	0.313927
Electronic Energy, BS2 (a.u.)	-11171.912587
Number of Imaginary Frequencies	0
Imaginary frequencies (cm-1)	None

Molecular Geometry in Cartesian Coordinates

N	-1.582934	2.111971	-0.779442
N	-2.843538	1.771435	-0.461876
N	-1.928589	-0.991445	-0.432604
N	-2.935443	-0.622163	0.376072
N	-0.863171	0.748011	1.873341
N	-2.120377	1.226326	1.893681

B	-3.130839	0.868004	0.767336
C	-1.652558	2.906235	-1.845178
C	-2.991599	3.087604	-2.243522
C	-3.725466	2.338190	-1.329666
C	-1.985274	-2.317107	-0.548088
C	-3.050521	-2.834262	0.215957
C	-3.636832	-1.711475	0.792789
C	-0.248438	1.227448	2.953017
C	-1.118332	2.048805	3.696191
C	-2.310006	2.024939	2.978584
H	-4.233893	1.041655	1.147687
C	-0.941051	-3.046464	-1.337419
F	-0.302930	-2.223163	-2.190265
F	-1.465688	-4.058121	-2.050615
C	-0.405804	3.463068	-2.458260
F	0.678845	3.182343	-1.698129
F	-0.468611	4.801828	-2.592919
C	1.184301	0.894954	3.235518
F	1.655512	-0.029635	2.366329
F	1.341874	0.397539	4.477661
F	1.972862	1.985009	3.135172
F	0.000734	-3.580340	-0.516184
F	-0.178630	2.946762	-3.685961
C	-3.630072	2.695598	3.232412
C	-4.816691	-1.571638	1.711309
C	-5.203797	2.122506	-1.185229
Br	-3.592488	4.138716	-3.695404
Br	-0.700996	2.944595	5.308142
Br	-3.529988	-4.655801	0.390483
F	-3.549781	3.560068	4.256254
F	-4.036279	3.386456	2.143314
F	-4.593861	1.799382	3.524730
F	-5.682996	2.716817	-0.072954
F	-5.876774	2.615861	-2.236523
F	-5.491628	0.804428	-1.094369
F	-4.473997	-0.899248	2.833997
F	-5.293366	-2.769509	2.084790
F	-5.823066	-0.892339	1.125420
C	2.282792	0.716335	-2.496414
O	1.720037	0.418235	-3.536867
O	3.100574	1.751487	-2.308926
C	3.228547	2.705926	-3.407390
H	2.264262	2.792146	-3.910853
H	3.956142	2.301765	-4.118843
C	3.682012	4.018142	-2.804405
H	3.829884	4.755863	-3.599661
H	4.624877	3.898127	-2.262702
H	2.926199	4.399076	-2.112829
C	2.025400	-0.087496	-1.291049
H	3.221281	2.148951	0.395614
C	4.105001	1.748409	0.895448
H	3.864176	0.784361	1.345270
H	4.903572	1.625706	0.159838
Ag	0.160788	0.348145	-0.359149
H	4.429195	2.439614	1.677306
C	2.924644	-1.140741	-0.986651
C	4.136656	-1.339826	-1.713588
C	2.634003	-2.015902	0.099033
C	5.008212	-2.355814	-1.363920
C	3.509384	-3.033482	0.441478
C	4.693373	-3.200708	-0.288327
H	4.376203	-0.680700	-2.541791

H	1.712766	-1.869985	0.650335
H	5.930700	-2.502001	-1.916257
H	3.280287	-3.697518	1.268229
H	5.379983	-3.997970	-0.017910

45-TpBrCF3_2Ag_C-Ph-CO2Et_CH4_Ethoxy_TS

Charge	0
Electronic Energy, BS1 (a.u.)	-3486.178072
Thermal and entropic correction, BS1 (a.u.)	0.320014
Electronic Energy, BS2 (a.u.)	-11171.882205
Number of Imaginary Frequencies	1
Imaginary frequencies (cm-1)	-483.3i

Molecular Geometry in Cartesian Coordinates

N	-1.541360	2.212125	-0.725868
N	-2.760234	1.735278	-0.417789
N	-1.558102	-0.857926	-0.506869
N	-2.556960	-0.654906	0.371925
N	-0.729117	1.018080	1.939912
N	-2.045071	1.274364	1.955318
B	-2.959952	0.782220	0.797466
C	-1.682804	2.977419	-1.805890
C	-3.026765	2.998182	-2.225962
C	-3.685208	2.183718	-1.309055
C	-1.496748	-2.167955	-0.732313
C	-2.476463	-2.845544	0.020114
C	-3.133393	-1.838025	0.718739
C	-0.187857	1.677868	2.963336
C	-1.176634	2.391204	3.671013
C	-2.357249	2.101584	2.990073
H	-4.088241	0.800846	1.141215
C	-0.507532	-2.732520	-1.707714
F	0.267548	-1.765344	-2.234292
F	-1.134527	-3.356993	-2.726905
C	-0.489221	3.643392	-2.418192
F	0.599391	3.520300	-1.621937
F	-0.696256	4.957203	-2.621761
C	1.302377	1.669348	3.087871
F	1.814998	0.442753	2.816780
F	1.732135	2.033502	4.301384
F	1.865243	2.517231	2.181573
F	0.300725	-3.639913	-1.121333
F	-0.171892	3.095226	-3.612753
C	-3.773320	2.544340	3.226935
C	-4.289454	-1.887653	1.674348
C	-5.130533	1.791380	-1.192960
Br	-3.718369	3.948428	-3.706586
Br	-0.930965	3.482849	5.197019
Br	-2.794551	-4.709242	0.015451
F	-3.857739	3.396059	4.260414
F	-4.267962	3.173114	2.136611
F	-4.579464	1.496340	3.489929
F	-5.701778	2.325185	-0.094408
F	-5.834735	2.197732	-2.260724
F	-5.258762	0.448212	-1.102655
F	-4.018053	-1.181224	2.795707
F	-4.566908	-3.147921	2.044192
F	-5.405404	-1.362628	1.128228

C	2.762424	0.987224	-2.048105
O	2.461156	0.504059	-3.126736
O	3.228408	2.238280	-1.885319
C	3.278206	3.060951	-3.083656
H	2.317712	2.988975	-3.597828
H	4.052090	2.661186	-3.747103
C	3.579769	4.475896	-2.637218
H	3.649419	5.130362	-3.511883
H	4.528666	4.525145	-2.094120
H	2.782879	4.849392	-1.988600
C	2.520368	0.262999	-0.776484
H	2.825615	0.981044	0.212560
C	4.047407	1.132394	0.523271
H	4.043029	0.539674	1.436167
H	4.811764	0.833426	-0.183171
Ag	0.363911	0.717589	-0.287401
H	4.000646	2.209931	0.671018
C	2.876205	-1.163405	-0.628834
C	3.586567	-1.864899	-1.620781
C	2.484012	-1.854444	0.535365
C	3.870239	-3.219370	-1.458845
C	2.797796	-3.198307	0.706805
C	3.485205	-3.888718	-0.295376
H	3.878344	-1.358299	-2.532656
H	1.929892	-1.326930	1.304495
H	4.397580	-3.752847	-2.244144
H	2.486451	-3.713295	1.610660
H	3.711595	-4.943796	-0.173055

46-TpBrCF3_2Ag_C-Ph-CO2Et_CH4_Oxy_Int-I

Charge	0
Electronic Energy, BS1 (a.u.)	-3486.205996
Thermal and entropic correction, BS1 (a.u.)	0.315626
Electronic Energy, BS2 (a.u.)	-11171.912850
Number of Imaginary Frequencies	0
Imaginary frequencies (cm-1)	None

Molecular Geometry in Cartesian Coordinates

Ag	5.105413	-1.100341	9.399488
Br	8.451974	2.149584	5.132577
Br	-0.669347	0.186660	7.283848
Br	5.427803	2.890208	14.162838
F	7.170073	-0.940279	4.833142
F	6.312202	-1.891185	6.594295
F	8.339431	-1.097415	6.659140
F	7.243649	4.642204	6.515390
F	6.299665	4.477123	8.468194
F	5.075746	4.612617	6.673207
F	0.200211	-1.963583	9.562390
F	2.251083	-2.198129	10.251788
F	1.732201	-2.670264	8.190655
F	0.526365	2.918549	6.467865
F	2.681438	3.192466	6.359535
F	1.570367	3.951071	8.070816
F	5.879726	-0.892795	12.977895
F	7.474819	0.511442	13.444505
F	7.483070	-0.555309	11.545003
F	3.600456	4.686001	12.429715

F	2.571889	3.875543	10.692594
F	4.378121	5.062406	10.434688
C	5.723562	-3.095668	9.795090
N	5.560468	0.518337	7.506632
N	5.350538	1.821917	7.760016
N	2.969627	0.138224	9.135201
N	3.007041	1.366640	8.593637
N	5.577773	1.044439	10.523075
N	4.808478	2.099952	10.211581
C	6.567450	0.449922	6.638416
C	7.037946	1.739346	6.319497
C	6.229906	2.593042	7.063157
C	7.090944	-0.873801	6.174327
C	6.212751	4.090573	7.175123
C	1.797367	-0.399304	8.804209
C	1.048910	0.493952	8.012144
C	1.862832	1.617623	7.901134
C	1.486824	-1.807697	9.206609
C	1.653152	2.927491	7.197000
C	5.890593	1.151428	11.813270
C	5.299314	2.303080	12.369396
C	4.613315	2.884355	11.305235
C	6.687849	0.055862	12.455667
C	3.789476	4.137048	11.219952
O	4.456752	-4.113944	11.472138
C	4.655910	-3.987341	10.274066
B	4.257181	2.271851	8.769000
H	3.969440	3.399402	8.576986
O	3.947531	-4.552934	9.298282
C	2.791956	-5.343972	9.712040
H	2.112564	-4.688910	10.262667
H	3.133939	-6.131359	10.390578
C	2.160499	-5.903573	8.456175
H	1.294137	-6.515902	8.726245
H	1.824394	-5.096140	7.801984
H	2.869134	-6.531042	7.907315
H	6.580275	-3.644673	12.870664
C	7.588233	-3.461184	13.247343
H	8.117512	-4.410081	13.371094
H	8.131371	-2.827624	12.543780
H	7.527403	-2.952878	14.212912
C	7.040983	-3.609998	9.731194
C	8.099982	-2.756350	9.308102
C	7.353034	-4.961752	10.069523
C	9.397459	-3.230728	9.210703
C	8.653209	-5.425406	9.983682
C	9.672071	-4.561239	9.552080
H	7.869234	-1.728617	9.056781
H	6.563406	-5.620211	10.416246
H	10.195395	-2.576297	8.876369
H	8.888309	-6.450877	10.249112
H	10.691195	-4.931373	9.484053

47-TpBrCF3_2Ag_C-Ph-CO2Et_CH4_Oxy_TS

	Value
Charge	0
Electronic Energy, BS1 (a.u.)	-3486.178836
Thermal and entropic correction, BS1 (a.u.)	0.321165
Electronic Energy, BS2 (a.u.)	-11171.883006

	Value
Number of Imaginary Frequencies	1
Imaginary frequencies (cm-1)	-403.1i

Molecular Geometry in Cartesian Coordinates

Ag	5.094505	-1.170295	10.075659
Br	8.197070	0.900799	5.021157
Br	-0.677655	-0.085510	7.881037
Br	5.382115	3.611136	14.091840
F	6.255902	-1.997755	5.274103
F	5.837059	-2.604658	7.322993
F	7.871685	-2.101819	6.722000
F	7.475616	3.693582	6.203983
F	6.553278	3.934475	8.160176
F	5.317644	3.908599	6.368543
F	0.294500	-1.547211	10.913934
F	2.359208	-2.238121	10.915502
F	1.048785	-2.711489	9.241605
F	0.635085	2.363430	6.513617
F	2.798508	2.524739	6.354640
F	1.729562	3.656653	7.875584
F	5.589991	-0.555668	13.362428
F	7.013389	0.905938	14.125424
F	7.482227	-0.255091	12.344035
F	3.857768	5.188673	11.937622
F	2.754921	4.092700	10.417415
F	4.637698	5.062843	9.912920
C	6.009485	-3.130207	10.689375
N	5.426616	-0.025830	7.867697
N	5.388342	1.316943	7.946905
N	2.984876	0.058680	9.671194
N	3.065179	1.165278	8.909620
N	5.605532	1.112994	10.862975
N	4.926325	2.132747	10.319261
C	6.324676	-0.336687	6.936418
C	6.895995	0.830260	6.391295
C	6.270310	1.870624	7.070774
C	6.573913	-1.767893	6.566439
C	6.406776	3.359634	6.944439
C	1.793679	-0.487741	9.438435
C	1.071522	0.276098	8.500579
C	1.926942	1.325363	8.180596
C	1.373129	-1.746275	10.133419
C	1.766161	2.475314	7.227495
C	5.850655	1.433841	12.133053
C	5.313403	2.701732	12.434118
C	4.730231	3.116985	11.238468
C	6.492072	0.398588	13.002505
C	3.993971	4.376319	10.878458
O	4.322599	-4.599151	11.628927
C	4.881639	-4.106874	10.667653
B	4.362118	2.025050	8.874253
H	4.119434	3.101954	8.458481
O	4.504363	-4.289227	9.386230
C	3.280257	-5.038143	9.159589
H	2.517602	-4.684953	9.856230
H	3.475656	-6.095185	9.370028
C	2.888172	-4.800654	7.716435
H	1.978465	-5.363451	7.483928
H	2.689238	-3.739867	7.543974

H	3.681525	-5.123291	7.035902
H	6.122777	-2.703592	11.873991
C	6.627594	-3.418956	12.773325
H	6.759695	-4.475111	12.576399
H	7.560085	-2.871261	12.901251
H	5.868132	-3.224321	13.529327
C	7.290475	-3.420249	10.016537
C	8.291916	-2.429443	9.962689
C	7.538636	-4.662194	9.402133
C	9.500512	-2.673789	9.320104
C	8.736738	-4.891089	8.729229
C	9.722908	-3.903005	8.692198
H	8.109584	-1.466969	10.428945
H	6.779626	-5.436632	9.424907
H	10.260548	-1.898943	9.288364
H	8.904071	-5.845892	8.239726
H	10.658096	-4.086824	8.171685

48-TpF27_Ag_C-Ph-CO2Et_CH4_Ethoxy_Int-I

Charge	0
Electronic Energy, BS1 (a.u.)	-4802.406483
Thermal and entropic correction, BS1 (a.u.)	0.403833
Electronic Energy, BS2 (a.u.)	-4805.688704
Number of Imaginary Frequencies	0
Imaginary frequencies (cm-1)	None

Molecular Geometry in Cartesian Coordinates

F	6.952406	-0.696724	4.390520
F	4.771633	-0.857127	4.729952
F	0.381156	-2.096132	10.114727
F	2.485213	-2.189938	10.766103
F	6.670011	1.538827	14.051657
F	6.851871	-0.005720	12.484586
N	4.963366	0.646984	6.955912
N	5.311247	1.530793	7.918762
N	3.043931	0.028259	9.270513
N	2.952094	1.241972	8.680023
N	5.239438	1.429167	10.846368
N	4.575957	2.405119	10.190537
C	6.067206	0.211826	6.375223
C	7.219317	0.804023	6.965394
C	6.677862	1.676938	7.951895
C	5.985490	-0.861300	5.338079
C	1.891351	-0.608766	9.115231
C	0.989065	0.183828	8.359725
C	1.720111	1.376950	8.106085
C	1.667219	-1.972370	9.691479
C	5.352400	1.773542	12.119732
C	4.718709	3.024180	12.351900
C	4.231657	3.397398	11.069190
C	5.984942	0.841820	13.105167
B	4.159735	2.220678	8.714713
H	3.848648	3.267753	8.249351
C	8.608948	0.661001	6.833533
C	9.436225	1.381875	7.665997
C	8.898697	2.282662	8.612566
C	7.535103	2.449031	8.758006
C	3.515711	4.596614	10.899466

C	4.488903	3.846495	13.467657
C	3.793518	5.022487	13.288816
C	3.311248	5.390323	12.010540
C	-0.313686	0.024277	7.855923
C	1.135684	2.428941	7.374990
C	-0.149117	2.251035	6.903969
C	-0.873286	1.057332	7.138094
F	-1.000848	-1.108504	8.049721
F	-2.109943	0.954647	6.634039
F	-0.742187	3.218994	6.196943
F	1.781291	3.572900	7.114780
F	4.917425	3.499281	14.687471
F	3.545296	5.838863	14.321582
F	2.629616	6.536016	11.898326
F	3.014325	4.975624	9.715117
F	9.143396	-0.217812	5.969953
F	10.767570	1.220646	7.608653
F	9.745157	2.981448	9.378495
F	7.081110	3.335844	9.655958
C	6.177606	-2.276389	5.932756
F	7.421301	-2.394554	6.440085
F	5.295647	-2.473609	6.927151
F	6.003586	-3.226112	5.008233
C	1.927425	-3.119778	8.688275
F	1.210801	-2.915821	7.572998
F	3.229936	-3.159262	8.368670
F	1.578277	-4.303060	9.212749
C	4.949643	-0.030788	13.858078
F	4.078824	0.765166	14.501291
F	4.254438	-0.789891	12.981774
F	5.542622	-0.831865	14.742316
H	6.785528	-4.542632	7.898009
H	5.367842	-5.403521	7.252122
C	5.951808	-5.198431	8.152493
H	6.337254	-6.138488	8.557347
H	5.313074	-4.716418	8.894801
C	6.392964	-2.449675	10.546334
C	5.848048	-3.195450	11.696284
O	6.299249	-2.988820	12.811724
O	4.799775	-3.967688	11.411783
C	4.109619	-4.549178	12.559629
H	4.812901	-5.188778	13.101976
H	3.812781	-3.730969	13.222548
C	2.922399	-5.319977	12.027207
H	2.396298	-5.797257	12.860062
H	3.243060	-6.098649	11.328712
H	2.229622	-4.654107	11.511647
Ag	5.083046	-0.880069	9.990240
C	7.698215	-2.760053	10.102489
C	8.333655	-1.895942	9.167182
C	8.402806	-3.922605	10.541408
C	9.605570	-2.169999	8.692113
C	9.665070	-4.201521	10.051197
C	10.264414	-3.325343	9.130884
H	7.797717	-1.013508	8.840619
H	7.937138	-4.587861	11.260510
H	10.079915	-1.507067	7.977571
H	10.195639	-5.090244	10.376643
H	11.258075	-3.548431	8.752773

49-k2-TpF27Ag_C-Ph-CO2Et_CH4_Ethoxy_TS

Charge	0
Electronic Energy, BS1 (a.u.)	-4802.381171
Thermal and entropic correction, BS1 (a.u.)	0.408709
Electronic Energy, BS2 (a.u.)	-4805.660120
Number of Imaginary Frequencies	1
Imaginary frequencies (cm-1)	-529.5713

Molecular Geometry in Cartesian Coordinates

F	6.813206	-0.744872	4.262050
F	4.641497	-0.931596	4.645388
F	0.231782	-2.122259	10.002139
F	2.338751	-2.265393	10.643436
F	6.944892	1.068799	13.829785
F	6.905257	-0.425031	12.203810
N	4.871167	0.547084	6.894512
N	5.237945	1.443880	7.837132
N	2.938549	-0.037801	9.220398
N	2.889465	1.186976	8.651389
N	5.287061	1.167487	10.738235
N	4.614736	2.194147	10.171781
C	5.963660	0.135295	6.273057
C	7.123975	0.755714	6.815584
C	6.600429	1.620113	7.816744
C	5.868681	-0.926549	5.227554
C	1.772153	-0.638769	9.035184
C	0.902727	0.190985	8.280862
C	1.671236	1.366427	8.058302
C	1.518661	-2.008063	9.579628
C	5.502173	1.453943	12.013072
C	4.937398	2.715872	12.339485
C	4.375602	3.160255	11.111722
C	6.147135	0.451471	12.917146
B	4.131285	2.117030	8.705470
H	3.845854	3.203192	8.318798
C	8.510766	0.638621	6.636579
C	9.347850	1.397209	7.424396
C	8.825679	2.288820	8.387817
C	7.465509	2.418917	8.586265
C	3.704814	4.394987	11.041066
C	4.828481	3.501375	13.499295
C	4.173702	4.710857	13.417803
C	3.616693	5.150049	12.193542
C	-0.396331	0.076977	7.757636
C	1.130779	2.442961	7.330109
C	-0.151131	2.309233	6.836315
C	-0.914370	1.135106	7.044714
F	-1.117670	-1.038556	7.929873
F	-2.145874	1.075916	6.521440
F	-0.703109	3.302106	6.130460
F	1.817140	3.567813	7.091981
F	5.333598	3.088845	14.668265
F	4.038701	5.494447	14.495966
F	2.981285	6.327237	12.174963
F	3.139907	4.845933	9.911785
F	9.030825	-0.239590	5.767099
F	10.680289	1.259669	7.329593
F	9.683375	3.011426	9.117670
F	7.019066	3.292013	9.502210

C	6.085509	-2.346749	5.796468
F	7.308095	-2.449478	6.352220
F	5.170506	-2.591814	6.759740
F	5.965959	-3.282757	4.851405
C	1.754102	-3.137349	8.550418
F	1.018140	-2.916034	7.454042
F	3.055074	-3.169215	8.197451
F	1.426503	-4.332527	9.058513
C	5.109976	-0.376248	13.717652
F	4.343125	0.452184	14.447210
F	4.301836	-1.049989	12.866735
F	5.702298	-1.247461	14.531074
H	6.659717	-4.402284	7.996791
H	4.938290	-4.777330	8.522468
C	5.967267	-4.572252	8.817731
H	6.338653	-5.268183	9.559266
H	5.724978	-3.364701	9.148425
C	6.301208	-2.906658	10.165415
C	5.719596	-3.475468	11.412273
O	6.213043	-3.279242	12.507701
O	4.553929	-4.122525	11.216280
C	3.846097	-4.508116	12.427413
H	4.515616	-5.112029	13.047461
H	3.603347	-3.597549	12.983119
C	2.609645	-5.273866	12.009320
H	2.067464	-5.603556	12.901268
H	2.877410	-6.158842	11.423652
H	1.946911	-4.647512	11.411430
Ag	5.010168	-1.078636	9.805784
C	7.771045	-2.795857	10.047951
C	8.330624	-1.857350	9.163146
C	8.638135	-3.626844	10.784876
C	9.711400	-1.751121	9.010373
C	10.017603	-3.508226	10.646879
C	10.556818	-2.575006	9.756565
H	7.671469	-1.212393	8.593357
H	8.227971	-4.346572	11.483771
H	10.130809	-1.029167	8.318308
H	10.674227	-4.145728	11.231320
H	11.633764	-2.487449	9.646673

50-TpF27_Ag_C-Ph-CO2Et_CH4_Oxy_Int-I

Charge	0
Electronic Energy, BS1 (a.u.)	-4802.405320
Thermal and entropic correction, BS1 (a.u.)	0.402550
Electronic Energy, BS2 (a.u.)	-4805.686664
Number of Imaginary Frequencies	0
Imaginary frequencies (cm-1)	None

Molecular Geometry in Cartesian Coordinates

Ag	5.316376	-1.058530	9.781135
F	6.792344	-1.336220	4.922832
F	5.780207	-2.113601	6.729088
F	0.437187	-1.938766	9.893235
F	2.532419	-2.166784	10.556297
F	6.501566	0.938964	14.309661
F	6.633747	-0.541458	12.680342
C	6.180294	-2.972802	10.150832

N	5.692941	0.341059	7.754845
N	5.591393	1.675299	7.935515
N	3.193999	0.154628	9.345890
N	3.227118	1.384533	8.782843
N	5.451416	1.231573	10.963761
N	4.967533	2.330070	10.336386
C	6.522940	0.109930	6.749577
C	7.028892	1.332859	6.231440
C	6.396529	2.321120	7.034005
C	6.768887	-1.289807	6.285249
C	2.022832	-0.401017	9.082186
C	1.224520	0.469997	8.292147
C	2.046643	1.618315	8.125528
C	1.737172	-1.804839	9.513461
C	5.467305	1.470398	12.266236
C	4.961467	2.770065	12.547655
C	4.652812	3.292617	11.263903
C	5.833979	0.407920	13.250226
O	4.456690	-4.565394	10.354890
C	5.344914	-4.086977	9.670352
B	4.499692	2.275500	8.862336
H	4.239461	3.365887	8.491134
O	5.668359	-4.440844	8.424873
C	4.823687	-5.412669	7.739790
H	3.807728	-5.339153	8.130569
H	5.215251	-6.407957	7.975476
C	4.898025	-5.106139	6.258172
H	4.364797	-5.879824	5.696311
H	4.441744	-4.137777	6.043581
H	5.937433	-5.080049	5.918320
H	5.032047	-4.015212	12.795280
C	5.696160	-3.913957	13.656251
H	6.359104	-4.782115	13.706304
H	6.291062	-3.005249	13.556041
H	5.103984	-3.857420	14.572318
C	7.916605	1.708587	5.209894
C	8.161158	3.049772	5.008091
C	7.535004	4.029808	5.815174
C	6.658885	3.686912	6.825714
C	4.118003	4.588715	11.134607
C	4.717114	3.527759	13.705211
C	4.191320	4.793750	13.567434
C	3.896540	5.316294	12.287250
C	-0.052919	0.414997	7.710679
C	1.583267	2.720315	7.382395
C	0.321830	2.642784	6.825996
C	-0.495670	1.499465	6.985313
F	-0.829781	-0.667861	7.842370
F	-1.704840	1.495070	6.407891
F	-0.154889	3.664888	6.105629
F	2.321387	3.822449	7.186425
F	4.971448	3.039573	14.926190
F	3.940114	5.555145	14.640915
F	3.384463	6.550176	12.210359
F	3.810044	5.133862	9.949740
F	8.524708	0.790268	4.447446
F	9.005124	3.458662	4.051027
F	7.823094	5.315887	5.584618
F	6.107516	4.644820	7.584567
C	8.104981	-1.895227	6.768612
F	8.165659	-1.859229	8.113978
F	8.225096	-3.167631	6.370799

F	9.134138	-1.186314	6.281548
C	1.980397	-2.833041	8.381743
F	3.262517	-2.760043	7.962406
F	1.741980	-4.080771	8.799875
F	1.182651	-2.563924	7.337322
C	4.595571	-0.334352	13.811915
F	3.705278	0.552603	14.285853
F	4.015206	-1.049089	12.833493
F	4.945915	-1.159841	14.805219
C	7.424133	-3.306211	10.743058
C	8.330256	-2.267215	11.098707
C	7.818322	-4.658101	10.975760
C	9.567888	-2.563189	11.645709
C	9.046491	-4.946177	11.544118
C	9.920884	-3.899524	11.874175
H	8.031586	-1.240994	10.923751
H	7.133931	-5.462882	10.728332
H	10.256688	-1.766101	11.905098
H	9.335651	-5.974963	11.732261
H	10.886306	-4.130074	12.315866

51-TpF27_Ag_C-Ph-CO2Et_CH4_Oxy_TS

Charge	0
Electronic Energy, BS1 (a.u.)	-4802.379675
Thermal and entropic correction, BS1 (a.u.)	0.408103
Electronic Energy, BS2 (a.u.)	-4805.658072
Number of Imaginary Frequencies	1
Imaginary frequencies (cm-1)	-441.0i

Molecular Geometry in Cartesian Coordinates

Ag	5.257779	-1.110223	9.873718
F	6.672982	-1.506146	5.069036
F	5.705889	-2.227302	6.919531
F	0.342617	-1.860963	9.995108
F	2.430788	-2.126387	10.665273
F	6.369338	0.851992	14.463353
F	6.465320	-0.623365	12.824337
C	5.975134	-3.120976	10.679455
N	5.672995	0.262434	7.895198
N	5.585577	1.604215	8.041680
N	3.143266	0.182567	9.469210
N	3.214301	1.409200	8.904100
N	5.427226	1.203831	11.087699
N	4.992459	2.311013	10.443464
C	6.449428	-0.002027	6.856352
C	6.937190	1.201240	6.280073
C	6.349309	2.216668	7.081869
C	6.684915	-1.415414	6.428110
C	1.956709	-0.337938	9.205780
C	1.184980	0.554993	8.413358
C	2.041215	1.678033	8.245287
C	1.644465	-1.740722	9.620014
C	5.425255	1.452364	12.388520
C	4.958016	2.770168	12.651679
C	4.693258	3.292553	11.357848
C	5.707751	0.372823	13.378378
O	4.101825	-4.623044	10.352232
C	5.155684	-4.147820	9.970206

B	4.520469	2.251120	8.969320
H	4.297464	3.344653	8.585013
O	5.732406	-4.432785	8.787753
C	5.007673	-5.349973	7.925115
H	4.017577	-4.934799	7.726112
H	4.873972	-6.296454	8.459524
C	5.826775	-5.517186	6.662531
H	5.327066	-6.226255	5.994618
H	5.936823	-4.563905	6.143176
H	6.824389	-5.902576	6.893171
H	5.357147	-2.777297	11.716951
C	5.288769	-3.588081	12.684140
H	5.547676	-4.626457	12.526466
H	5.950975	-3.066801	13.371732
H	4.224426	-3.445903	12.863851
C	7.781104	1.537980	5.208804
C	8.023843	2.870268	4.954224
C	7.441110	3.878531	5.758751
C	6.611157	3.573724	6.819296
C	4.207738	4.605753	11.209232
C	4.719996	3.544930	13.798623
C	4.240804	4.827174	13.642047
C	3.989677	5.349582	12.352450
C	-0.092318	0.535763	7.830098
C	1.611147	2.791351	7.499340
C	0.348537	2.749577	6.941285
C	-0.502402	1.631178	7.101813
F	-0.899885	-0.524180	7.963645
F	-1.710526	1.661094	6.522976
F	-0.097259	3.784044	6.218724
F	2.381129	3.871443	7.302814
F	4.937291	3.055780	15.026970
F	3.996994	5.605374	14.704831
F	3.524328	6.600386	12.256748
F	3.945381	5.153329	10.014910
F	8.350551	0.592416	4.450612
F	8.825766	3.243539	3.948043
F	7.726158	5.154733	5.476473
F	6.105946	4.560970	7.572482
C	8.034445	-1.993801	6.905078
F	8.098616	-1.944007	8.245626
F	8.180438	-3.264662	6.511806
F	9.043960	-1.265178	6.401876
C	1.881541	-2.754578	8.474309
F	3.170898	-2.694353	8.074795
F	1.609376	-4.002374	8.857541
F	1.103698	-2.440440	7.425131
C	4.417033	-0.309065	13.893603
F	3.608419	0.592002	14.462695
F	3.768266	-0.884972	12.860438
F	4.707726	-1.262729	14.791466
C	7.426712	-3.350911	10.867304
C	8.295871	-2.273680	11.116010
C	7.970986	-4.648829	10.799375
C	9.662111	-2.483919	11.285498
C	9.338030	-4.857995	10.954427
C	10.187208	-3.775399	11.201416
H	7.895254	-1.268085	11.161655
H	7.319105	-5.495371	10.607128
H	10.318146	-1.638707	11.470740
H	9.741899	-5.863753	10.886748
H	11.254005	-3.938544	11.324361

52-TpBr_3Ag_C-pC6H4-CF3-CO2CH2CF3_CH4_Ethoxy_Int-I

Charge	0
Electronic Energy, BS1 (a.u.)	-2174.159446
Thermal and entropic correction, BS1 (a.u.)	0.208249
Electronic Energy, BS2 (a.u.)	-25225.300351
Number of Imaginary Frequencies	0
Imaginary frequencies (cm-1)	None

Molecular Geometry in Cartesian Coordinates

N	-2.077919	2.132087	-1.047318
N	-3.105593	1.559124	-0.353049
N	-1.889790	-0.894959	-1.082252
N	-2.341426	-0.802343	0.198087
N	-0.427541	0.983782	1.358824
N	-1.733532	1.109412	1.718538
B	-2.841171	0.542341	0.791531
C	-2.616223	3.007007	-1.892015
C	-4.016360	3.028354	-1.772470
C	-4.278925	2.086599	-0.778877
C	-1.485961	-2.149079	-1.245763
C	-1.660348	-2.909648	-0.069718
C	-2.210414	-1.994899	0.827752
C	0.280056	1.677457	2.242106
C	-0.550655	2.272880	3.211548
C	-1.833244	1.883082	2.826232
H	-3.838450	0.377892	1.416637
Br	-5.247127	4.085209	-2.725298
Br	-0.052841	3.336953	4.683762
Br	-1.241015	-4.721396	0.229367
C	2.530107	0.609437	-2.740549
O	2.318877	0.386702	-3.913718
O	3.472048	1.455416	-2.247063
C	3.948651	2.487052	-3.115468
H	3.869980	2.199033	-4.165607
H	4.985829	2.691151	-2.848821
C	1.732057	0.055515	-1.646002
H	4.596701	2.757139	-0.043854
C	5.294569	2.031303	0.375676
H	4.838961	1.038534	0.362736
H	6.211494	2.012523	-0.220929
Ag	-0.047084	1.135895	-1.213844
H	5.548138	2.310885	1.402125
Br	-1.513523	4.005510	-3.047348
Br	-5.961753	1.583639	-0.102851
Br	2.156715	1.793648	2.050326
Br	-3.475819	2.323739	3.633813
Br	-2.682336	-2.252478	2.632755
Br	-0.734805	-2.696052	-2.890470
C	2.117835	-1.158560	-1.030618
C	1.425975	-1.595046	0.136137
C	3.159410	-1.979984	-1.558781
C	1.757280	-2.796108	0.742483
C	3.455423	-3.195858	-0.974146
C	2.748844	-3.599052	0.171585
H	0.652084	-0.962762	0.557075
H	3.690946	-1.664894	-2.450812
H	1.238653	-3.125279	1.634085
H	4.226527	-3.837659	-1.385472
C	3.106950	3.727990	-2.867450

F	1.798733	3.468734	-3.093895
F	3.215726	4.164581	-1.596816
F	3.490789	4.721069	-3.689422
C	3.035377	-4.966876	0.750479
F	2.609763	-5.078614	2.021832
F	2.412428	-5.919490	0.023265
F	4.355680	-5.241786	0.727262

53-TpBr_3Ag_C-pC6H4-CF3-CO2CH2CF3_CH4_Ethoxy_TS

Charge	0
Electronic Energy, BS1 (a.u.)	-2174.134821
Thermal and entropic correction, BS1 (a.u.)	0.212774
Electronic Energy, BS2 (a.u.)	-25225.277601
Number of Imaginary Frequencies	1
Imaginary frequencies (cm-1)	-443.2i

Molecular Geometry in Cartesian Coordinates

N	-1.684476	2.213682	-0.828802
N	-2.836982	1.627955	-0.397886
N	-1.438027	-0.791953	-0.962732
N	-2.188171	-0.765235	0.174804
N	-0.546834	0.928206	1.803405
N	-1.900847	1.059973	1.876894
B	-2.802609	0.548562	0.718133
C	-2.020841	3.133192	-1.726503
C	-3.414828	3.177308	-1.904101
C	-3.890358	2.195559	-1.034326
C	-1.010186	-2.043735	-1.099489
C	-1.456509	-2.862006	-0.042275
C	-2.206386	-1.992273	0.747587
C	-0.055387	1.537378	2.874882
C	-1.073781	2.081942	3.680452
C	-2.239503	1.751994	2.991402
H	-3.903202	0.359520	1.124238
Br	-4.406244	4.303035	-3.041228
Br	-0.909567	3.018375	5.306208
Br	-1.099555	-4.687317	0.251749
C	2.738141	0.822258	-2.361810
O	2.594377	0.119958	-3.343145
O	3.096231	2.131986	-2.432804
C	3.179168	2.665809	-3.755682
H	2.238783	2.530667	-4.295478
H	3.988047	2.200703	-4.324981
C	2.403649	0.388998	-0.986804
H	2.777384	1.281994	-0.146309
C	3.990317	1.442900	0.138481
H	4.052925	0.900447	1.078956
H	4.731786	1.131601	-0.587423
Ag	0.309975	1.121135	-0.668526
H	3.925192	2.528475	0.232117
Br	-0.671218	4.147251	-2.566843
Br	-5.680145	1.692610	-0.744868
Br	1.814025	1.607609	3.164362
Br	-4.016297	2.171412	3.450735
Br	-3.098288	-2.347787	2.368077
Br	0.075435	-2.513182	-2.567143
C	2.663065	-0.996119	-0.553179
C	1.966268	-1.507501	0.558868

C	3.550127	-1.842649	-1.247216
C	2.120845	-2.833674	0.946644
C	3.694115	-3.171858	-0.870298
C	2.972845	-3.667781	0.220874
H	1.277468	-0.870334	1.103948
H	4.090808	-1.469693	-2.108905
H	1.566858	-3.224036	1.791899
H	4.361183	-3.827364	-1.419982
C	3.465180	4.144401	-3.603905
F	2.485492	4.778949	-2.924634
F	4.620633	4.363769	-2.947719
F	3.557279	4.707168	-4.823589
C	3.068346	-5.133111	0.555590
F	2.649258	-5.399056	1.810267
F	2.311039	-5.875534	-0.285858
F	4.337258	-5.587602	0.442408

54-TpBr_3Ag_C-pC6H4-CF3-CO2CH2CF3_CH4_Oxy_Int-I

Charge	0
Electronic Energy, BS1 (a.u.)	-2174.162902
Thermal and entropic correction, BS1 (a.u.)	0.209848
Electronic Energy, BS2 (a.u.)	-25225.306659
Number of Imaginary Frequencies	0
Imaginary frequencies (cm-1)	None

Molecular Geometry in Cartesian Coordinates

Ag	5.970075	-1.621857	10.334855
Br	5.850389	-0.374304	4.173577
Br	-0.486627	-1.036446	11.051662
Br	7.143652	3.849610	13.157698
C	6.552533	-3.635454	10.083520
N	6.014845	-0.356398	8.256887
N	5.005896	0.532662	8.052685
N	3.494270	-1.037987	10.123607
N	3.163773	0.231438	9.767614
N	5.869511	0.557498	11.106793
N	5.246079	1.534557	10.390387
C	6.416092	-0.768189	7.060903
C	5.674527	-0.155071	6.034342
C	4.778967	0.662283	6.721619
C	2.388742	-1.608882	10.576419
C	1.294253	-0.723098	10.525358
C	1.843520	0.445165	10.002215
C	6.557603	1.154006	12.071086
C	6.400534	2.550326	12.016512
C	5.554425	2.743947	10.925517
O	4.990645	-5.412238	9.960974
C	5.479926	-4.422184	9.450697
B	4.254716	1.195407	9.238902
H	3.744375	2.196485	8.866318
O	5.061728	-3.844618	8.308972
C	3.862113	-4.345605	7.712014
H	3.090969	-4.515032	8.465501
H	4.052617	-5.270334	7.161237
H	3.253914	1.803767	12.603628
C	3.216144	0.901235	13.217576
H	3.635387	0.060822	12.662503
H	3.796674	1.060815	14.130187

H	2.177601	0.687039	13.481783
Br	2.440136	-3.391774	11.194284
Br	0.970988	2.081968	9.677694
Br	7.822596	-2.017747	6.913565
Br	3.431202	1.758215	5.996885
Br	4.909834	4.384567	10.264583
Br	7.559460	0.121457	13.291026
C	7.862225	-4.186687	10.125117
C	8.904998	-3.420913	10.715941
C	8.189167	-5.447333	9.543561
C	10.216930	-3.867611	10.691886
C	9.496051	-5.898413	9.534649
C	10.504929	-5.101693	10.099022
H	8.652935	-2.470919	11.175510
H	7.403358	-6.057544	9.112006
H	11.013014	-3.278725	11.132009
H	9.748573	-6.856693	9.094415
C	11.936886	-5.580258	10.008896
F	12.732228	-4.967010	10.905061
F	12.022824	-6.909557	10.217247
F	12.435555	-5.331381	8.779268
C	3.398472	-3.276266	6.741925
F	3.154309	-2.106640	7.362921
F	4.316073	-3.046853	5.782832
F	2.260373	-3.684323	6.146766

55-TpBr_3Ag_C-pC6H4-CF3-CO2CH2CF3_CH4_Oxy_TS

Charge	0
Electronic Energy, BS1 (a.u.)	-2174.137761
Thermal and entropic correction, BS1 (a.u.)	0.212323
Electronic Energy, BS2 (a.u.)	-25225.277093
Number of Imaginary Frequencies	1
Imaginary frequencies (cm-1)	-350.6i

Molecular Geometry in Cartesian Coordinates

Ag	5.184052	-1.265844	10.327613
Br	8.061746	0.212926	4.850765
Br	-0.646687	-0.592869	8.356470
Br	6.065512	4.211281	13.375439
C	5.858933	-3.269289	11.107048
N	5.657800	-0.401513	8.099052
N	5.372006	0.903048	7.830616
N	3.087164	-0.104339	9.947274
N	3.112269	0.848371	8.974831
N	5.763434	1.078579	10.764773
N	5.060980	2.026782	10.085457
C	6.548992	-0.795035	7.198794
C	6.868663	0.249078	6.308137
C	6.091100	1.314161	6.755847
C	1.892510	-0.679940	9.889416
C	1.103493	-0.122586	8.867251
C	1.932011	0.850376	8.309076
C	6.168827	1.638912	11.894716
C	5.744280	2.977359	11.989344
C	5.034743	3.176198	10.805917
O	3.857737	-4.528361	11.625128
C	4.708681	-4.164576	10.841137
B	4.387320	1.699181	8.724739

H	4.088720	2.708955	8.180774
O	4.683605	-4.437685	9.502794
C	3.423837	-4.796251	8.932134
H	2.612929	-4.659874	9.648723
H	3.450456	-5.835004	8.594908
H	5.743945	-2.873971	12.331937
C	5.971615	-3.591125	13.316206
H	5.985386	-4.661979	13.153654
H	6.930146	-3.183814	13.635928
H	5.112333	-3.269465	13.906336
Br	1.489499	-2.097823	11.066248
Br	1.560985	1.995949	6.861376
Br	7.235515	-2.550565	7.241203
Br	6.006496	3.056604	6.043801
Br	4.158608	4.745353	10.242663
Br	7.175016	0.631980	13.141213
C	7.237037	-3.624712	10.730940
C	8.251511	-2.650859	10.821874
C	7.561655	-4.895216	10.218814
C	9.542516	-2.925238	10.391158
C	8.846165	-5.162474	9.756613
C	9.834127	-4.178030	9.843272
H	8.010457	-1.666320	11.210741
H	6.799162	-5.662321	10.146005
H	10.314897	-2.166957	10.454614
H	9.087344	-6.134860	9.341156
C	11.198930	-4.444790	9.265161
F	12.156277	-3.710611	9.870355
F	11.546575	-5.745656	9.378463
F	11.235783	-4.139558	7.946842
C	3.196666	-3.894513	7.731597
F	3.273480	-2.590146	8.070649
F	4.098899	-4.115880	6.758648
F	1.968116	-4.125715	7.226143

56-TpBrCF3_2Ag_C-pC6H4-CF3-CO2CH2CF3_CH4_Ethoxy_Int-I

Charge	0
Electronic Energy, BS1 (a.u.)	-4120.944777
Thermal and entropic correction, BS1 (a.u.)	0.288541
Electronic Energy, BS2 (a.u.)	-11806.961037
Number of Imaginary Frequencies	0
Imaginary frequencies (cm-1)	None

Molecular Geometry in Cartesian Coordinates

N	-1.486649	2.099928	-0.733148
N	-2.757559	1.792553	-0.423127
N	-1.887155	-0.994750	-0.427697
N	-2.904707	-0.618277	0.363674
N	-0.811015	0.693974	1.909345
N	-2.065630	1.182256	1.929798
B	-3.071882	0.869100	0.784123
C	-1.529905	2.922837	-1.778015
C	-2.862050	3.161171	-2.168500
C	-3.620444	2.412325	-1.274467
C	-1.973294	-2.315441	-0.578496
C	-3.070688	-2.823244	0.144880
C	-3.643992	-1.699356	0.733786
C	-0.210651	1.119110	3.019674

C	-1.087912	1.909720	3.786590
C	-2.266297	1.931423	3.047679
H	-4.174066	1.055518	1.159224
C	-0.918059	-3.045473	-1.352620
F	-0.283416	-2.225730	-2.213920
F	-1.424697	-4.072251	-2.054155
C	-0.265324	3.466518	-2.364500
F	0.812276	3.052864	-1.658887
F	-0.254274	4.811184	-2.376173
C	1.200013	0.732367	3.342336
F	1.750629	-0.012108	2.351942
F	1.261724	-0.000282	4.472367
F	1.982324	1.813282	3.519102
F	0.028287	-3.553975	-0.519400
F	-0.085034	3.052890	-3.640874
C	-3.580436	2.612397	3.308869
C	-4.841608	-1.553833	1.629222
C	-5.105377	2.237476	-1.140411
Br	-3.423249	4.276042	-3.587800
Br	-0.696067	2.709501	5.453771
Br	-3.599470	-4.635071	0.266588
F	-3.501094	3.434226	4.366946
F	-3.957623	3.352047	2.242014
F	-4.562581	1.721920	3.551854
F	-5.570138	2.804505	-0.008464
F	-5.758811	2.789279	-2.174615
F	-5.432401	0.926078	-1.099081
F	-4.504036	-0.930636	2.781129
F	-5.362369	-2.748127	1.950488
F	-5.812534	-0.824865	1.044231
C	2.224592	0.577423	-2.530533
O	1.569020	0.354116	-3.530179
O	3.151029	1.542710	-2.405599
C	3.265680	2.454762	-3.508845
H	2.287116	2.698662	-3.923677
H	3.901465	2.029118	-4.289244
C	2.012991	-0.201775	-1.302848
H	2.268100	2.846674	0.367154
C	3.259804	2.582044	0.739340
H	3.285311	1.520678	0.992425
H	3.999998	2.796316	-0.032301
Ag	0.204980	0.290442	-0.298000
H	3.479557	3.166693	1.635770
C	2.874691	-1.304192	-1.055057
C	3.952513	-1.643303	-1.925009
C	2.664705	-2.098567	0.106596
C	4.772999	-2.719785	-1.643997
C	3.493374	-3.171940	0.390880
C	4.540618	-3.476830	-0.485418
H	4.126214	-1.056743	-2.821207
H	1.850551	-1.842853	0.773720
H	5.587036	-2.985825	-2.308685
H	3.331625	-3.777918	1.274561
C	5.486835	-4.610753	-0.155003
F	6.580218	-4.137192	0.479085
F	4.912515	-5.525334	0.648716
F	5.907188	-5.242090	-1.269213
C	3.919963	3.712957	-2.976333
F	4.166051	4.544597	-4.009556
F	3.138662	4.359734	-2.095084
F	5.092314	3.443447	-2.365999

57-TpBrCF3_2Ag_C-pC6H4-CF3-CO2CH2CF3_CH4_Ethoxy_TS

Charge	0
Electronic Energy, BS1 (a.u.)	-4120.921913
Thermal and entropic correction, BS1 (a.u.)	0.291597
Electronic Energy, BS2 (a.u.)	-11806.936687
Number of Imaginary Frequencies	1
Imaginary frequencies (cm-1)	-363.1i

Molecular Geometry in Cartesian Coordinates

N	-1.550910	2.191082	-0.729757
N	-2.753186	1.674051	-0.422104
N	-1.464842	-0.871559	-0.534420
N	-2.462710	-0.711693	0.354070
N	-0.692940	1.018008	1.928146
N	-2.017755	1.223835	1.948033
B	-2.917864	0.707787	0.787408
C	-1.718285	2.959244	-1.804045
C	-3.063501	2.942728	-2.219673
C	-3.693823	2.100748	-1.307622
C	-1.344423	-2.178733	-0.753760
C	-2.280908	-2.898531	0.014064
C	-2.978637	-1.918864	0.713127
C	-0.175779	1.676087	2.965747
C	-1.190701	2.334374	3.689223
C	-2.359673	2.016169	2.999908
H	-4.044932	0.683641	1.134224
C	-0.339892	-2.699059	-1.736860
F	0.365521	-1.693892	-2.293039
F	-0.939694	-3.380610	-2.733512
C	-0.549637	3.680755	-2.401280
F	0.556666	3.531258	-1.635827
F	-0.784401	4.997898	-2.529009
C	1.314284	1.699040	3.094164
F	1.845278	0.459138	2.940767
F	1.736154	2.181722	4.266475
F	1.868890	2.464028	2.110681
F	0.536741	-3.541545	-1.148817
F	-0.242873	3.205035	-3.632244
C	-3.791026	2.406275	3.241604
C	-4.123617	-2.016224	1.678547
C	-5.126392	1.664055	-1.189623
Br	-3.786801	3.886887	-3.688503
Br	-0.993541	3.392545	5.245233
Br	-2.497471	-4.776075	0.031694
F	-3.905131	3.242120	4.284649
F	-4.305625	3.030422	2.158000
F	-4.560227	1.328070	3.491208
F	-5.709908	2.176392	-0.087599
F	-5.844712	2.052747	-2.254294
F	-5.212943	0.317267	-1.103860
F	-3.868993	-1.303731	2.800016
F	-4.350164	-3.287049	2.045189
F	-5.262329	-1.530848	1.143146
C	2.737791	1.066015	-2.123627
O	2.407524	0.594090	-3.195944
O	3.212775	2.324476	-1.978072
C	3.217551	3.115888	-3.170840
H	2.221846	3.164924	-3.613611
H	3.924077	2.719420	-3.904125

C	2.529968	0.352422	-0.846706
H	2.907592	1.096072	0.134562
C	4.095598	1.300263	0.449694
H	4.096612	0.759856	1.395150
H	4.856777	0.962052	-0.242783
Ag	0.398282	0.779287	-0.283561
H	4.042842	2.384149	0.533698
C	2.930101	-1.058472	-0.688762
C	3.566228	-1.778858	-1.715798
C	2.630773	-1.722461	0.519366
C	3.870562	-3.126771	-1.547058
C	2.964154	-3.056536	0.699515
C	3.576749	-3.763089	-0.341066
H	3.781218	-1.297828	-2.661554
H	2.128832	-1.181492	1.313695
H	4.334472	-3.683895	-2.352998
H	2.722517	-3.560926	1.628996
C	3.941155	-5.207474	-0.126128
F	5.064379	-5.322761	0.623390
F	2.965483	-5.873096	0.530870
F	4.169812	-5.856281	-1.286919
C	3.659477	4.502654	-2.755570
F	3.718364	5.292109	-3.846588
F	2.809020	5.063831	-1.876759
F	4.881899	4.482234	-2.185214

58-TpBrCF3_2Ag_C-pC6H4-CF3-CO2CH2CF3_CH4_Oxy_Int-I

Charge	0
Electronic Energy, BS1 (a.u.)	-4120.946013
Thermal and entropic correction, BS1 (a.u.)	0.290055
Electronic Energy, BS2 (a.u.)	-11806.958272
Number of Imaginary Frequencies	0
Imaginary frequencies (cm-1)	None

Molecular Geometry in Cartesian Coordinates

Ag	5.192375	-1.237379	9.645887
Br	7.788816	1.306784	4.567260
Br	-0.923653	-0.148780	8.499646
Br	5.812517	3.341081	13.873670
F	6.696011	-1.744063	4.920624
F	5.775351	-2.456724	6.759397
F	7.844031	-1.781906	6.768409
F	6.795321	3.983656	5.753711
F	6.150885	4.111797	7.827272
F	4.674596	4.000034	6.231064
F	0.601552	-1.544015	11.426058
F	2.582441	-2.319821	10.960931
F	0.901225	-2.750881	9.646014
F	0.177230	2.271102	6.890527
F	2.298467	2.495372	6.460878
F	1.402300	3.577323	8.123220
F	6.030943	-0.619369	13.139326
F	7.669682	0.781525	13.436944
F	7.626503	-0.487489	11.667717
F	3.974959	4.970155	12.016027
F	2.733349	3.923759	10.569432
F	4.514133	4.988720	9.910724
C	5.963011	-3.212347	9.833851

N	5.313063	0.070443	7.567760
N	5.141933	1.398910	7.667018
N	2.983987	-0.018007	9.662032
N	2.942113	1.084749	8.891613
N	5.683838	1.013832	10.518381
N	4.922267	2.042870	10.112415
C	6.165998	-0.142504	6.567101
C	6.576337	1.080904	6.001028
C	5.893852	2.046247	6.736036
C	6.614074	-1.535839	6.245222
C	5.878705	3.544494	6.630268
C	1.766268	-0.553680	9.637607
C	0.903030	0.208461	8.827432
C	1.699458	1.249551	8.361818
C	1.463680	-1.792784	10.421512
C	1.386859	2.405283	7.456234
C	6.080568	1.284372	11.761327
C	5.556583	2.520453	12.188750
C	4.818941	2.974744	11.097857
C	6.858047	0.247155	12.512434
C	4.009142	4.225312	10.900639
O	4.317001	-4.591655	10.837513
C	4.863495	-4.178742	9.836743
B	4.191129	1.997068	8.740517
H	3.861532	3.083816	8.422935
O	4.490937	-4.451768	8.561046
C	3.105858	-4.718020	8.310910
H	2.508723	-4.603232	9.216150
H	2.989536	-5.726795	7.909706
H	6.062940	-3.720601	12.768383
C	7.032273	-3.588488	13.254926
H	7.575735	-4.537502	13.245104
H	7.608837	-2.827810	12.727248
H	6.878560	-3.270613	14.288947
C	7.306400	-3.657495	9.816313
C	8.349426	-2.698072	9.697898
C	7.651943	-5.037403	9.913770
C	9.675846	-3.096928	9.664481
C	8.976886	-5.430323	9.903288
C	9.981439	-4.457755	9.774730
H	8.086592	-1.649479	9.632663
H	6.868236	-5.780217	10.020713
H	10.471855	-2.367910	9.570737
H	9.246163	-6.476531	9.995703
C	11.422240	-4.917080	9.713323
F	12.284331	-3.914843	9.965062
F	11.657355	-5.901256	10.604831
F	11.706619	-5.406232	8.488205
C	2.654045	-3.710574	7.267850
F	2.868540	-2.441767	7.687396
F	3.314658	-3.864768	6.106920
F	1.338125	-3.858501	7.030113

59-TpBrCF3_2Ag_C-pC6H4-CF3-CO2CH2CF3_CH4_Oxy_TS

Charge	0
Electronic Energy, BS1 (a.u.)	-4120.927595
Thermal and entropic correction, BS1 (a.u.)	0.293668
Electronic Energy, BS2 (a.u.)	-11806.936068
Number of Imaginary Frequencies	1

Imaginary frequencies (cm-1)	-301.5i

Molecular Geometry in Cartesian Coordinates

Ag	5.048542	-1.303813	10.100678
Br	8.301139	0.636274	5.020969
Br	-0.693585	-0.135682	7.960937
Br	5.282953	3.569428	14.001870
F	6.325795	-2.302882	5.417513
F	6.104282	-2.819427	7.519256
F	8.056192	-2.258490	6.729636
F	7.510678	3.457391	6.061048
F	6.598328	3.767241	8.011801
F	5.351799	3.649273	6.231142
F	0.339599	-1.359003	11.186738
F	2.324505	-2.243112	11.047493
F	0.819763	-2.693167	9.541601
F	0.724475	2.124461	6.379920
F	2.892502	2.245558	6.248160
F	1.815985	3.483895	7.678228
F	5.479477	-0.593695	13.400964
F	6.914261	0.863135	14.151611
F	7.403533	-0.368832	12.424100
F	3.866170	5.110562	11.751346
F	2.794708	3.978682	10.235039
F	4.698217	4.919022	9.752841
C	5.975608	-3.239626	10.785275
N	5.534844	-0.222320	7.893749
N	5.464171	1.121638	7.908986
N	2.997147	-0.031254	9.690568
N	3.115744	1.014173	8.851306
N	5.617586	0.995959	10.842072
N	4.964964	2.006279	10.249187
C	6.448180	-0.553776	6.983543
C	6.994078	0.600081	6.386549
C	6.338889	1.655213	7.012295
C	6.733879	-1.990255	6.664031
C	6.451470	3.140019	6.822131
C	1.777959	-0.535959	9.515539
C	1.076795	0.190854	8.535387
C	1.974331	1.173468	8.128430
C	1.313344	-1.707946	10.324272
C	1.845670	2.264363	7.103978
C	5.815650	1.346633	12.112534
C	5.274333	2.623670	12.363348
C	4.739797	3.013003	11.136874
C	6.410400	0.329306	13.033451
C	4.023429	4.266598	10.720305
O	4.120072	-4.539868	11.637722
C	4.841093	-4.187572	10.729953
B	4.424820	1.856909	8.797495
H	4.193520	2.921622	8.346229
O	4.635898	-4.513558	9.416842
C	3.300278	-4.827171	9.019534
H	2.579281	-4.560500	9.792825
H	3.214618	-5.890992	8.785048
H	6.051090	-2.839806	12.031578
C	6.463295	-3.490384	12.983314
H	6.608293	-4.549783	12.807696
H	7.387376	-2.949037	13.182112
H	5.649329	-3.284140	13.676850
C	7.288845	-3.528043	10.192337

C	8.243752	-2.494142	10.100822
C	7.595056	-4.784660	9.635090
C	9.445858	-2.695087	9.438871
C	8.785572	-4.977638	8.942606
C	9.705340	-3.932184	8.840975
H	8.022061	-1.525506	10.533742
H	6.879387	-5.596196	9.700321
H	10.169282	-1.891501	9.357593
H	9.002789	-5.935627	8.482878
C	10.939076	-4.100448	7.995016
F	11.965067	-3.345137	8.445052
F	11.359256	-5.384448	7.963569
F	10.701197	-3.726858	6.716897
C	3.020218	-4.021465	7.763818
F	3.167568	-2.693280	7.987459
F	3.848582	-4.353036	6.758654
F	1.755212	-4.238053	7.358128

60-TpF27_Ag_C-pC6H4-CF3-CO2CH2CF3_CH4_Ethoxy_Int-I

Charge	0
Electronic Energy, BS1 (a.u.)	-5437.143571
Thermal and entropic correction, BS1 (a.u.)	0.379601
Electronic Energy, BS2 (a.u.)	-5440.735414
Number of Imaginary Frequencies	2
Imaginary frequencies (cm-1)	-17.9i, -7.8i

Molecular Geometry in Cartesian Coordinates

F	6.979576	-0.812776	4.588991
F	4.881858	-1.228105	5.160101
F	0.445461	-2.268090	9.849572
F	2.542538	-2.350989	10.523660
F	6.757013	1.647241	14.096990
F	6.893221	0.066742	12.560664
N	5.030368	0.548560	7.143673
N	5.327555	1.539415	8.015138
N	3.045842	0.035935	9.280454
N	2.952104	1.279629	8.752682
N	5.261137	1.479082	10.925663
N	4.582216	2.440566	10.265668
C	6.154291	0.142630	6.578526
C	7.268235	0.873923	7.081903
C	6.679242	1.783499	8.005217
C	6.133984	-1.021251	5.639314
C	1.907985	-0.607555	9.060274
C	1.010053	0.216385	8.333272
C	1.727616	1.432332	8.165686
C	1.728448	-2.036630	9.471131
C	5.395510	1.847355	12.191737
C	4.762399	3.099826	12.411769
C	4.250261	3.446962	11.131557
C	6.045309	0.935193	13.183076
B	4.167644	2.242034	8.788921
H	3.876094	3.288632	8.310967
C	8.661817	0.840550	6.919062
C	9.443076	1.690382	7.671654
C	8.853822	2.613896	8.564356
C	7.484273	2.679328	8.731834
C	3.527906	4.640727	10.950306

C	4.553394	3.944886	13.514831
C	3.852234	5.115514	13.325011
C	3.343539	5.456312	12.048776
C	-0.282040	0.070479	7.798279
C	1.136644	2.522372	7.498107
C	-0.137261	2.355949	6.994228
C	-0.845456	1.138788	7.137044
F	-0.957404	-1.079234	7.912204
F	-2.072514	1.051239	6.608199
F	-0.735790	3.360761	6.346267
F	1.761528	3.695472	7.334088
F	5.008133	3.623134	14.732087
F	3.622541	5.952629	14.344851
F	2.657026	6.597645	11.927596
F	3.001980	4.991107	9.768161
F	9.250564	-0.043591	6.099125
F	10.781074	1.632020	7.590716
F	9.655452	3.432000	9.255965
F	6.970664	3.585680	9.577018
C	6.579033	-2.349128	6.305653
F	7.897677	-2.316053	6.569114
F	5.926897	-2.523653	7.471366
F	6.326995	-3.398224	5.515753
C	2.061064	-3.021606	8.324159
F	1.304896	-2.733132	7.253136
F	3.355368	-2.895780	7.987338
F	1.828671	-4.287347	8.686842
C	5.024758	0.083042	13.977243
F	4.178187	0.892931	14.632662
F	4.301642	-0.683056	13.130381
F	5.637720	-0.711112	14.854849
H	5.305832	-5.272411	7.240945
H	4.021156	-5.640615	8.425984
C	5.094527	-5.509032	8.286097
H	5.613671	-6.428821	8.567619
H	5.432556	-4.686006	8.914981
C	6.392543	-2.349035	10.700673
C	5.862212	-3.162675	11.805794
O	6.218364	-2.963075	12.952495
O	4.906305	-4.027975	11.432954
C	4.271826	-4.741362	12.503613
H	5.001451	-5.308869	13.086198
H	3.735933	-4.054085	13.162214
Ag	5.033622	-0.837483	10.113533
C	7.732962	-2.564192	10.294957
C	8.352838	-1.616099	9.436418
C	8.488783	-3.701306	10.713115
C	9.662565	-1.783285	9.014471
C	9.785837	-3.878620	10.274730
C	10.365903	-2.916532	9.430368
H	7.781931	-0.749223	9.125705
H	8.037438	-4.431742	11.375720
H	10.133245	-1.067101	8.352759
H	10.361429	-4.746267	10.578838
C	3.288307	-5.699805	11.866391
F	2.324993	-5.055489	11.187864
F	3.900691	-6.547308	11.017438
F	2.702829	-6.428960	12.838983
C	11.790859	-3.146563	8.976552
F	12.241735	-2.159281	8.184245
F	12.616434	-3.237594	10.040137
F	11.885693	-4.306342	8.293998

61-k2-TpF27Ag_C-pC6H4-CF3-CO2CH2CF3_CH4_Ethoxy_TS

Charge	0
Electronic Energy, BS1 (a.u.)	-5437.124345
Thermal and entropic correction, BS1 (a.u.)	0.376680
Electronic Energy, BS2 (a.u.)	-5440.711612
Number of Imaginary Frequencies	1
Imaginary frequencies (cm-1)	-402.8i

Molecular Geometry in Cartesian Coordinates

Ag	5.087820	-1.107801	10.067954
F	7.625310	-1.600665	5.885761
F	6.986886	-2.128849	7.930237
F	0.158750	-1.753661	9.458118
F	2.130806	-2.122703	10.379826
F	6.374413	0.773551	14.567482
F	6.430403	-0.696574	12.921261
C	5.728912	-3.204913	10.751736
N	6.083338	0.395038	8.304078
N	5.675941	1.684033	8.211755
N	3.087473	0.182332	9.326544
N	3.241732	1.431782	8.836896
N	5.301629	1.116215	11.237806
N	4.873582	2.229617	10.602991
C	6.899086	0.139167	7.293444
C	7.058375	1.289618	6.472818
C	6.243757	2.268186	7.104985
C	7.604183	-1.175170	7.177893
C	1.917676	-0.287198	8.918440
C	1.258176	0.660869	8.092227
C	2.155213	1.763611	8.068958
C	1.500745	-1.681099	9.251792
C	5.334540	1.361080	12.538582
C	4.889948	2.682180	12.813692
C	4.604119	3.212278	11.526341
C	5.675133	0.283890	13.512799
O	4.884668	-4.682572	9.104937
C	4.756278	-4.167912	10.196764
B	4.537549	2.255813	9.091487
H	4.341565	3.371889	8.774179
O	3.663963	-4.340658	10.996438
C	2.792897	-5.418701	10.666940
H	2.867070	-5.689420	9.613643
H	1.775281	-5.113947	10.911205
H	5.381927	-2.874251	11.938736
C	5.503717	-3.657477	12.914529
H	5.665363	-4.707153	12.701890
H	6.344670	-3.175333	13.408640
H	4.529642	-3.460652	13.358083
C	7.766151	1.596165	5.297685
C	7.660370	2.866131	4.774940
C	6.855155	3.839910	5.408528
C	6.148290	3.565525	6.562811
C	4.122123	4.530134	11.398891
C	4.684493	3.451644	13.970579
C	4.215267	4.739130	13.832668
C	3.938748	5.269255	12.551323
C	0.056486	0.700633	7.364947
C	1.845385	2.915100	7.319263
C	0.654303	2.931602	6.621168

C	-0.238968	1.834233	6.640586
F	-0.784218	-0.341865	7.355319
F	-1.369350	1.920627	5.927360
F	0.323420	4.001356	5.889605
F	2.666186	3.971596	7.241888
F	4.921915	2.949287	15.189714
F	4.001002	5.513075	14.904227
F	3.477242	6.522223	12.472601
F	3.823297	5.087969	10.217877
F	8.529444	0.683805	4.683775
F	8.314961	3.207410	3.657249
F	6.790853	5.059457	4.862349
F	5.414432	4.539362	7.118115
C	9.077577	-1.102985	7.657019
F	9.127193	-0.557968	8.888392
F	9.629291	-2.320306	7.700889
F	9.794320	-0.330028	6.828844
C	1.844958	-2.684458	8.127294
F	3.172939	-2.695469	7.921006
F	1.451540	-3.926029	8.457578
F	1.232127	-2.328094	6.990717
C	4.425271	-0.426967	14.083456
F	3.621293	0.455484	14.685016
F	3.745944	-1.020716	13.078743
F	4.776306	-1.373636	14.966709
C	7.172167	-3.512468	10.642332
C	8.112564	-2.511266	10.947811
C	7.639751	-4.782000	10.249189
C	9.474984	-2.760851	10.850246
C	9.002818	-5.022238	10.115458
C	9.918698	-4.011604	10.415532
H	7.766810	-1.528753	11.247098
H	6.935900	-5.572760	10.020559
H	10.191424	-1.978833	11.073375
H	9.357065	-5.993198	9.787173
C	11.387505	-4.225252	10.171420
F	12.148944	-3.549381	11.061509
F	11.733023	-5.528983	10.246632
F	11.741887	-3.789346	8.939849
C	3.195450	-6.602061	11.527487
F	4.499190	-6.918654	11.332806
F	3.047682	-6.334454	12.841418
F	2.449841	-7.680054	11.229590

62-TpF27_Ag_C-pC6H4-CF3-CO2CH2CF3_CH4_Oxy_Int-I

Charge	0
Electronic Energy, BS1 (a.u.)	-5437.144727
Thermal and entropic correction, BS1 (a.u.)	0.376088
Electronic Energy, BS2 (a.u.)	-5440.735023
Number of Imaginary Frequencies	0
Imaginary frequencies (cm-1)	None

Molecular Geometry in Cartesian Coordinates

Ag	5.247343	-0.966334	9.812098
F	6.531624	-1.113241	4.677617
F	5.385122	-1.966615	6.362271
F	0.421747	-1.983601	9.986011
F	2.544051	-2.194871	10.560760

F	6.548600	0.978376	14.281886
F	6.613465	-0.507263	12.653496
C	6.179966	-2.870469	10.026375
N	5.465464	0.373969	7.628932
N	5.457060	1.693299	7.922166
N	3.123405	0.175790	9.400551
N	3.125431	1.417317	8.862401
N	5.402840	1.271221	10.962211
N	4.934731	2.376226	10.335675
C	6.310043	0.170913	6.627983
C	6.921849	1.392684	6.234373
C	6.337499	2.351564	7.105528
C	6.459171	-1.195177	6.036900
C	1.948567	-0.386637	9.170325
C	1.113510	0.493011	8.429736
C	1.918143	1.652522	8.255828
C	1.701678	-1.809689	9.561991
C	5.480817	1.527505	12.259700
C	5.036159	2.849162	12.538356
C	4.700267	3.365059	11.258310
C	5.846009	0.461706	13.240574
O	4.506030	-4.545370	10.012191
C	5.368870	-3.934059	9.417692
B	4.400006	2.305291	8.883211
H	4.126497	3.393961	8.515076
O	5.697586	-4.069977	8.115637
C	4.953643	-4.996702	7.322454
H	4.803393	-4.535216	6.346595
H	3.998754	-5.246895	7.785938
H	4.942361	-3.964041	12.509498
C	5.600500	-3.853952	13.374139
H	6.302326	-4.692322	13.401953
H	6.152338	-2.916300	13.303584
H	5.003868	-3.851494	14.289070
C	7.866797	1.792810	5.274938
C	8.212185	3.125418	5.203720
C	7.632344	4.074350	6.080472
C	6.701629	3.707184	7.031678
C	4.227612	4.684656	11.124733
C	4.865998	3.633801	13.691617
C	4.393924	4.920335	13.550744
C	4.080835	5.438284	12.272489
C	-0.184780	0.436586	7.896445
C	1.415614	2.765187	7.555276
C	0.134749	2.685695	7.045312
C	-0.665186	1.530862	7.210990
F	-0.945262	-0.656571	8.036397
F	-1.895104	1.526003	6.680379
F	-0.379073	3.717840	6.366838
F	2.132815	3.880395	7.357824
F	5.140440	3.151803	14.910394
F	4.214740	5.707843	14.619084
F	3.629158	6.694754	12.193284
F	3.913866	5.227439	9.941029
F	8.433464	0.904808	4.447793
F	9.113791	3.554440	4.310378
F	8.019867	5.350463	5.976152
F	6.190874	4.627071	7.863888
C	7.725431	-1.958282	6.492891
F	7.807273	-1.964215	7.839579
F	7.702102	-3.224874	6.062969
F	8.820022	-1.350663	6.013542

C	1.923903	-2.789461	8.383011
F	3.176784	-2.640442	7.896920
F	1.770156	-4.060804	8.766105
F	1.057623	-2.522077	7.395392
C	4.606095	-0.255706	13.829334
F	3.761592	0.648877	14.348943
F	3.971731	-0.932992	12.856234
F	4.964557	-1.113308	14.791395
C	7.447901	-3.246028	10.540727
C	8.330013	-2.237655	11.015209
C	7.887038	-4.603526	10.558744
C	9.591420	-2.564714	11.485402
C	9.137928	-4.928654	11.049500
C	9.983765	-3.907717	11.509040
H	7.998626	-1.207393	10.997916
H	7.236956	-5.388754	10.187395
H	10.266289	-1.797026	11.845021
H	9.470320	-5.960200	11.079573
C	11.370773	-4.279488	11.986150
F	11.909851	-3.322065	12.764325
F	11.353048	-5.427829	12.691814
F	12.195367	-4.464394	10.933668
C	5.793254	-6.249583	7.169271
F	6.961421	-5.990838	6.552821
F	6.083929	-6.791334	8.376208
F	5.127394	-7.168752	6.447017

63-TpF27_Ag_C-pC6H4-CF3-CO2CH2CF3_CH4_Oxy_TS

Charge	0
Electronic Energy, BS1 (a.u.)	-5437.126126
Thermal and entropic correction, BS1 (a.u.)	0.380978
Electronic Energy, BS2 (a.u.)	-5440.713843
Number of Imaginary Frequencies	1
Imaginary frequencies (cm-1)	-316.4i

Molecular Geometry in Cartesian Coordinates

Ag	5.211287	-1.029725	9.910368
F	6.562593	-1.366390	4.966456
F	5.435873	-2.105851	6.716868
F	0.305837	-1.798986	9.960813
F	2.384116	-2.075925	10.659252
F	6.325961	0.856219	14.491098
F	6.454371	-0.604934	12.841520
C	5.974837	-3.032211	10.634150
N	5.591404	0.289529	7.863893
N	5.588266	1.624850	8.081854
N	3.117302	0.233196	9.479007
N	3.204229	1.456919	8.909079
N	5.407206	1.228884	11.112253
N	4.967577	2.338048	10.474907
C	6.395726	0.027007	6.845221
C	6.990946	1.222306	6.360342
C	6.436310	2.231751	7.193073
C	6.517334	-1.371373	6.327604
C	1.939670	-0.291315	9.183867
C	1.190465	0.597191	8.365548
C	2.050696	1.719646	8.214917
C	1.612512	-1.690147	9.601489

C	5.387812	1.461821	12.416385
C	4.902451	2.770894	12.687871
C	4.646115	3.304521	11.396880
C	5.678120	0.377048	13.398807
O	4.144540	-4.591856	10.288677
C	5.144864	-4.026083	9.904816
B	4.516860	2.288551	8.992615
H	4.313502	3.386486	8.607017
O	5.663139	-4.130470	8.647772
C	5.018418	-5.013472	7.734869
H	5.015761	-4.527678	6.759871
H	4.003015	-5.250983	8.053925
H	5.390765	-2.798539	11.758041
C	5.327133	-3.612026	12.687883
H	5.608508	-4.634295	12.470220
H	5.989408	-3.110129	13.390575
H	4.263360	-3.501354	12.892693
C	7.904743	1.558754	5.347885
C	8.245070	2.883776	5.180581
C	7.692945	3.885168	6.015671
C	6.796750	3.579648	7.020584
C	4.143547	4.612091	11.256315
C	4.641382	3.530080	13.840432
C	4.147073	4.807429	13.691765
C	3.902839	5.340448	12.404815
C	-0.071404	0.575572	7.749679
C	1.641690	2.828803	7.451518
C	0.394712	2.783990	6.859533
C	-0.461097	1.666782	7.004161
F	-0.882932	-0.482821	7.870405
F	-1.653368	1.694243	6.394131
F	-0.030951	3.813824	6.119252
F	2.417940	3.906902	7.271394
F	4.850815	3.029806	15.065408
F	3.880496	5.570498	14.759464
F	3.420178	6.584800	12.317689
F	3.884267	5.166124	10.064530
F	8.446597	0.617993	4.564261
F	9.116303	3.255693	4.233687
F	8.074018	5.152003	5.818981
F	6.318934	4.552263	7.810335
C	7.775067	-2.125690	6.813617
F	7.861402	-2.055434	8.153686
F	7.727492	-3.414208	6.453764
F	8.872443	-1.561901	6.288203
C	1.857671	-2.718985	8.470501
F	3.152718	-2.670408	8.086914
F	1.580115	-3.962033	8.870712
F	1.094864	-2.420442	7.408350
C	4.396233	-0.331418	13.900368
F	3.568211	0.550524	14.470037
F	3.764530	-0.912263	12.858978
F	4.699372	-1.286737	14.792192
C	7.426067	-3.281041	10.765614
C	8.298815	-2.222523	11.076251
C	7.968920	-4.563053	10.543987
C	9.670647	-2.432235	11.158266
C	9.340913	-4.772751	10.609337
C	10.190178	-3.707031	10.920576
H	7.897101	-1.230089	11.236921
H	7.320522	-5.395612	10.293893
H	10.337627	-1.611364	11.397162

H	9.753172	-5.759320	10.428353
C	11.681066	-3.923127	10.932212
F	12.309115	-3.055417	11.755306
F	12.003748	-5.172669	11.332267
F	12.210409	-3.754009	9.698866
C	5.837331	-6.286986	7.658994
F	7.090260	-6.051202	7.227143
F	5.937061	-6.877938	8.874896
F	5.257144	-7.162295	6.816572

64-TpBr_3Ag_CHCF3_CH4_Int-I

Charge	0
Electronic Energy, BS1 (a.u.)	-1378.124091
Thermal and entropic correction, BS1 (a.u.)	0.097973
Electronic Energy, BS2 (a.u.)	-24428.940738
Number of Imaginary Frequencies	0
Imaginary frequencies (cm-1)	None

Molecular Geometry in Cartesian Coordinates

N	-2.157295	2.279148	-1.595574
N	-3.196033	1.956447	-0.775432
N	-1.379302	-0.525377	-0.720607
N	-2.557550	-0.392773	-0.054276
N	-0.718543	1.952189	0.981511
N	-1.955357	1.588156	1.415472
B	-3.013311	1.000320	0.440163
C	-2.631872	3.072303	-2.550105
C	-4.010839	3.288429	-2.378984
C	-4.323911	2.556046	-1.234534
C	-1.264328	-1.802564	-1.048994
C	-2.371501	-2.550300	-0.597763
C	-3.173130	-1.601046	0.032224
C	-0.038748	2.396577	2.032966
C	-0.824361	2.326759	3.198098
C	-2.038089	1.804306	2.752723
H	-4.045371	0.900369	1.007066
Br	-5.162756	4.325829	-3.445460
Br	-0.349341	2.827954	4.948577
Br	-2.693918	-4.394947	-0.796933
C	1.830788	1.307658	-1.879791
H	2.273028	0.333462	-2.116009
H	0.905939	-0.537993	0.854779
C	1.965896	-0.659634	1.084757
H	2.128376	-0.501399	2.153986
H	2.284655	-1.668736	0.812341
Ag	-0.032047	1.601614	-1.193858
H	2.558121	0.072708	0.529261
Br	-1.485595	3.748672	-3.882901
Br	-6.010959	2.386728	-0.418271
Br	1.726443	3.015812	1.830083
Br	-3.568164	1.430632	3.782300
Br	-4.833911	-1.874385	0.877668
Br	0.245227	-2.403605	-2.013763
C	2.940525	2.318814	-1.855663
F	2.525876	3.572333	-1.619219
F	3.814262	1.982202	-0.875921
F	3.580206	2.312617	-3.044042

65-TpBr_3Ag_CHCF3_CH4_TS

Charge	0
Electronic Energy, BS1 (a.u.)	-1378.122703
Thermal and entropic correction, BS1 (a.u.)	0.097759
Electronic Energy, BS2 (a.u.)	-24428.939126
Number of Imaginary Frequencies	1
Imaginary frequencies (cm-1)	-39.8i

Molecular Geometry in Cartesian Coordinates

N	-2.079039	2.228863	-1.444063
N	-3.247271	1.997660	-0.787147
N	-1.688492	-0.647613	-0.488183
N	-2.924919	-0.391313	0.016963
N	-0.992943	1.700426	1.283650
N	-2.321807	1.546829	1.537808
B	-3.306171	1.046446	0.441959
C	-2.340668	3.056447	-2.449462
C	-3.707230	3.389042	-2.477522
C	-4.243305	2.688692	-1.397186
C	-1.637011	-1.945384	-0.739680
C	-2.849588	-2.582611	-0.405102
C	-3.642837	-1.543612	0.075961
C	-0.415000	2.099937	2.411045
C	-1.362457	2.216882	3.444372
C	-2.563705	1.852762	2.837826
H	-4.404180	1.051969	0.878823
Br	-4.602094	4.517991	-3.688198
Br	-1.075324	2.744171	5.227933
Br	-3.294632	-4.405332	-0.567162
C	1.705235	1.025338	-1.679718
H	2.084286	0.090525	-2.106525
H	2.496895	-0.216688	0.401462
C	3.572450	-0.217759	0.607112
H	3.719727	-0.582739	1.626904
H	4.086197	-0.879392	-0.094922
Ag	-0.081254	1.312795	-0.792859
H	3.975906	0.792407	0.519227
Br	-0.955654	3.626423	-3.592689
Br	-6.037940	2.660868	-0.831668
Br	1.438648	2.430833	2.466051
Br	-4.266551	1.775480	3.635792
Br	-5.412934	-1.645874	0.712303
Br	-0.061642	-2.711235	-1.451186
C	2.673498	2.122592	-2.023592
F	2.458647	3.263448	-1.353760
F	3.961442	1.776015	-1.837649
F	2.468695	2.359924	-3.344996

66-Rh2-Ac4-CHCO2Et

Charge	0
Electronic Energy, BS1 (a.u.)	-1439.626603
Thermal and entropic correction, BS1 (a.u.)	0.241071
Electronic Energy, BS2 (a.u.)	-1442.36012886
Number of Imaginary Frequencies	0
Imaginary frequencies (cm-1)	None

Molecular Geometry in Cartesian Coordinates

C	2.284586	1.180530	1.405012
C	-1.193477	2.455385	1.742816
C	-2.518500	-0.995463	1.269441
C	0.970098	-2.304230	1.072650
O	-0.930073	1.781677	2.779613
O	-0.986675	2.090156	0.532875
O	-1.957587	-0.931067	2.401438
O	-2.033240	-0.593288	0.154387
O	0.777400	-1.931019	2.265169
O	0.696614	-1.638056	0.015045
O	1.805979	0.794456	2.510489
O	1.723234	1.085883	0.256552
Rh	-0.154079	0.252478	0.140629
Rh	-0.072045	-0.082590	2.570623
C	-0.120990	0.396812	-1.777986
C	-1.213750	-0.068444	-2.634203
O	-1.918500	0.796457	-3.132704
O	-1.379965	-1.379508	-2.722651
C	-2.590335	-1.833331	-3.396261
H	-3.447572	-1.350859	-2.917610
H	-2.553563	-1.510929	-4.441676
H	0.733088	0.854675	-2.288308
C	-2.630891	-3.339999	-3.260192
H	-2.648298	-3.631090	-2.205819
H	-3.531877	-3.729347	-3.744800
H	-1.757782	-3.799446	-3.732850
C	3.643509	1.837677	1.412823
H	4.114710	1.732045	2.390339
H	4.273381	1.395472	0.636556
H	3.525004	2.900583	1.177652
C	1.584277	-3.663279	0.840878
H	1.759126	-4.171697	1.789371
H	0.917742	-4.260089	0.211495
H	2.528784	-3.545196	0.301238
C	-3.908126	-1.581206	1.198320
H	-3.971072	-2.287779	0.366783
H	-4.166459	-2.073011	2.136513
H	-4.621832	-0.773886	1.002762
C	-1.803725	3.824278	1.912524
H	-2.733611	3.882423	1.339817
H	-1.996040	4.029628	2.965868
H	-1.119499	4.575495	1.506008

67-Rh2-Ac4-C-Ph-CO2Et

Charge	0
Electronic Energy, BS1 (a.u.)	-1670.727900
Thermal and entropic correction, BS1 (a.u.)	0.317460
Electronic Energy, BS2 (a.u.)	-1673.53807394
Number of Imaginary Frequencies	0
Imaginary frequencies (cm-1)	None

Molecular Geometry in Cartesian Coordinates

C	2.266046	0.875983	1.368550
C	-1.071434	2.481103	1.864208
C	-2.717395	-0.841246	1.511159
C	0.572631	-2.456652	1.005301
O	-0.791787	1.785830	2.882190
O	-0.979115	2.111627	0.641592

O	-2.090159	-0.820273	2.611029
O	-2.265583	-0.482460	0.370534
O	0.506215	-2.080444	2.211494
O	0.299550	-1.755963	-0.028843
O	1.816767	0.519265	2.494583
O	1.630178	0.862674	0.256821
Rh	-0.332160	0.200586	0.203733
Rh	-0.133323	-0.160910	2.631253
C	-0.479100	0.495870	-1.770308
C	-0.944041	-0.699395	-2.512234
O	-2.121101	-0.945568	-2.698834
O	0.083066	-1.466843	-2.892422
C	-0.248133	-2.798068	-3.367195
H	-0.946207	-3.253622	-2.658739
H	-0.753647	-2.713491	-4.335188
C	1.050962	-3.569307	-3.464843
H	1.535392	-3.622356	-2.485982
H	0.853352	-4.587654	-3.815097
H	1.739563	-3.089670	-4.167072
C	3.697880	1.350584	1.293809
H	4.104892	1.503779	2.293730
H	4.294318	0.596276	0.769867
H	3.752875	2.275459	0.713625
C	1.037021	-3.868229	0.730857
H	1.112745	-4.435743	1.658847
H	0.341786	-4.358619	0.044316
H	2.017276	-3.832805	0.244431
C	-4.145720	-1.330727	1.517125
H	-4.244732	-2.167379	0.819428
H	-4.442990	-1.639945	2.519576
H	-4.802121	-0.530301	1.162544
C	-1.593936	3.880580	2.085588
H	-2.679233	3.877232	1.936713
H	-1.375150	4.215266	3.100293
H	-1.160048	4.564633	1.352420
C	-0.235417	1.692689	-2.510270
C	0.413893	2.804075	-1.905019
C	-0.628931	1.799629	-3.875472
C	0.661992	3.955966	-2.638664
C	-0.400420	2.964493	-4.589550
C	0.250321	4.041962	-3.973007
H	0.732611	2.726350	-0.875845
H	-1.141011	0.968016	-4.348518
H	1.171816	4.794027	-2.173602
H	-0.719265	3.040646	-5.624338
H	0.438174	4.950902	-4.537789

68-Rh2-Ac4-C-pCF3Ph-CO2CH2CF3

Charge	0
Electronic Energy, BS1 (a.u.)	-2305.475303
Thermal and entropic correction, BS1 (a.u.)	0.294284
Electronic Energy, BS2 (a.u.)	-2308.59279753
Number of Imaginary Frequencies	0
Imaginary frequencies (cm-1)	None

Molecular Geometry in Cartesian Coordinates

C	2.171338	1.297904	1.425935
C	-1.423952	2.110164	1.973897

C	-2.310222	-1.467802	1.109354
C	1.262624	-2.219253	0.611801
O	-1.046990	1.346009	2.906901
O	-1.219292	1.934116	0.720823
O	-1.738950	-1.434308	2.237260
O	-1.937987	-0.846471	0.053851
O	1.062728	-2.038516	1.849674
O	0.887455	-1.447566	-0.335220
O	1.776545	0.724823	2.481195
O	1.579012	1.289973	0.289655
Rh	-0.198174	0.256245	0.082943
Rh	0.019856	-0.367279	2.446618
C	-0.422901	0.726949	-1.842746
C	-1.245786	-0.261785	-2.575419
O	-2.403921	-0.025325	-2.854539
O	-0.616431	-1.433745	-2.794177
C	-1.434976	-2.555518	-3.123897
H	-2.486856	-2.353587	-2.915514
H	-1.310847	-2.813049	-4.178853
C	3.478498	2.051292	1.475184
H	3.783040	2.224102	2.507913
H	4.246235	1.456319	0.968803
H	3.385490	2.998744	0.938696
C	1.985987	-3.475602	0.194732
H	2.604693	-3.844584	1.013960
H	1.239146	-4.233297	-0.058767
H	2.589632	-3.289213	-0.695088
C	-3.536767	-2.332126	0.955335
H	-3.258892	-3.218718	0.376759
H	-3.916726	-2.639122	1.930177
H	-4.305013	-1.793159	0.395545
C	-2.207016	3.347840	2.337864
H	-3.239339	3.228026	1.993199
H	-2.198858	3.504145	3.416879
H	-1.786909	4.216661	1.824090
C	0.001112	1.904521	-2.536558
C	0.439423	3.046948	-1.816050
C	-0.003910	1.958311	-3.957457
C	0.858347	4.184008	-2.491071
C	0.438670	3.087099	-4.626644
C	0.866917	4.198620	-3.889714
H	0.419422	3.025851	-0.736098
H	-0.349879	1.101548	-4.526343
H	1.175455	5.063111	-1.941233
H	0.440700	3.122617	-5.710133
C	1.396844	5.412179	-4.613464
F	1.151988	6.549474	-3.929432
F	2.736265	5.326201	-4.778580
F	0.851065	5.545374	-5.840425
C	-0.976444	-3.733817	-2.286773
F	0.324359	-4.019301	-2.481730
F	-1.155884	-3.528974	-0.965268
F	-1.698673	-4.821502	-2.635466

69-Rh2-Ac4-CHCF3

Charge	0
Electronic Energy, BS1 (a.u.)	-1509.447695
Thermal and entropic correction, BS1 (a.u.)	0.179366
Electronic Energy, BS2 (a.u.)	-1512.23334363

Number of Imaginary Frequencies	0
Imaginary frequencies (cm-1)	None

Molecular Geometry in Cartesian Coordinates

C	2.219877	1.230694	1.342284
C	-1.245085	2.454457	1.717073
C	-2.534890	-1.046041	1.418103
C	0.922924	-2.308833	1.083773
O	-0.925876	1.808388	2.755565
O	-1.102564	2.055198	0.506686
O	-1.924068	-0.940258	2.521043
O	-2.113890	-0.641425	0.277173
O	0.799540	-1.903538	2.274967
O	0.592668	-1.666393	0.026076
O	1.801216	0.837540	2.468460
O	1.609018	1.112813	0.221497
Rh	-0.259988	0.221579	0.150760
Rh	-0.055502	-0.057623	2.577442
C	-0.078782	0.313164	-1.739091
C	3.562024	1.916637	1.280489
H	4.040520	1.910767	2.259987
H	4.196870	1.412595	0.546434
H	3.421893	2.948241	0.942566
C	1.503137	-3.680940	0.850133
H	1.821698	-4.127829	1.792116
H	0.744785	-4.312216	0.376539
H	2.347998	-3.607865	0.159785
C	-3.890598	-1.706019	1.410922
H	-3.857561	-2.587788	0.763898
H	-4.181926	-1.994598	2.421008
H	-4.627440	-1.016323	0.989097
C	-1.847208	3.826719	1.881890
H	-2.796230	3.876775	1.340935
H	-2.001702	4.052035	2.937306
H	-1.175185	4.568359	1.438756
H	0.573215	1.072488	-2.184740
C	-0.387780	-0.778685	-2.721295
F	-1.025295	-0.210644	-3.776364
F	0.749773	-1.353436	-3.176531
F	-1.173239	-1.749991	-2.243229

70-Rh2-Ac4-CHCO2Et-CH4_Ethoxy_Int-I

Charge	0
Electronic Energy, BS1 (a.u.)	-1480.156344
Thermal and entropic correction, BS1 (a.u.)	0.282540
Electronic Energy, BS2 (a.u.)	-1482.90212183
Number of Imaginary Frequencies	0
Imaginary frequencies (cm-1)	None

Molecular Geometry in Cartesian Coordinates

C	2.243979	1.234280	1.450523
C	-1.245554	2.488445	1.795860
C	-2.540471	-0.983075	1.330330
C	0.957485	-2.254678	1.134766
O	-0.984083	1.816317	2.834175
O	-1.028167	2.126072	0.587083
O	-1.978509	-0.910739	2.461063

O	-2.066790	-0.566370	0.215277
O	0.772661	-1.874851	2.325473
O	0.656127	-1.599651	0.076592
O	1.764224	0.862536	2.560318
O	1.681956	1.129164	0.303201
Rh	-0.192484	0.287621	0.194187
Rh	-0.104008	-0.035611	2.627505
C	-0.187292	0.473328	-1.722780
C	-1.286903	0.013009	-2.574897
O	-2.020419	0.877430	-3.031037
O	-1.429431	-1.297656	-2.700761
C	-2.625165	-1.754557	-3.395777
H	-3.497017	-1.320321	-2.897695
H	-2.597064	-1.383669	-4.425358
H	0.622303	1.006381	-2.230295
H	1.486681	-1.709662	-2.157498
C	1.799471	-1.769057	-3.202756
H	2.888927	-1.848349	-3.256024
H	1.345278	-2.650596	-3.662262
H	1.479917	-0.878878	-3.750981
C	-2.620033	-3.266419	-3.329167
H	-2.635024	-3.606309	-2.289414
H	-3.505234	-3.661017	-3.837946
H	-1.729628	-3.675514	-3.815574
C	3.620701	1.853473	1.445393
H	3.971563	2.016887	2.464621
H	4.310869	1.183066	0.922915
H	3.598362	2.798110	0.895384
C	1.595064	-3.601840	0.900327
H	1.783175	-4.107772	1.847552
H	0.938272	-4.210078	0.271806
H	2.535014	-3.464749	0.357035
C	-3.908635	-1.616708	1.253004
H	-3.881371	-2.451660	0.546425
H	-4.227469	-1.967747	2.234601
H	-4.623967	-0.883094	0.868833
C	-1.868752	3.851867	1.962647
H	-2.802931	3.896884	1.395619
H	-2.057302	4.059958	3.016134
H	-1.195387	4.608477	1.548286

71-Rh2-Ac4-CHCO2Et-CH4_Ethoxy_TS

Charge	0
Electronic Energy, BS1 (a.u.)	-1480.144403
Thermal and entropic correction, BS1 (a.u.)	0.286449
Electronic Energy, BS2 (a.u.)	-1482.88963974
Number of Imaginary Frequencies	1
Imaginary frequencies (cm-1)	-328.6i

Molecular Geometry in Cartesian Coordinates

C	2.329935	1.124130	1.518024
C	-1.181839	2.369401	1.479084
C	-2.433640	-1.125236	1.180552
C	1.099188	-2.383845	1.228624
O	-0.999683	1.772090	2.583339
O	-0.872314	1.933809	0.319904
O	-1.962101	-0.967505	2.346071
O	-1.863252	-0.811333	0.081814

O	0.804188	-1.944723	2.377338
O	0.900321	-1.784218	0.114846
O	1.761804	0.815651	2.607304
O	1.865253	0.943459	0.340431
Rh	0.003629	0.076492	0.140059
Rh	-0.103654	-0.085713	2.570150
C	0.082350	0.266774	-1.964471
C	-1.204775	-0.013725	-2.639264
O	-2.002474	0.879682	-2.851440
O	-1.426964	-1.318387	-2.861159
C	-2.787488	-1.675529	-3.210588
H	-3.454274	-1.261645	-2.448987
H	-3.042048	-1.218884	-4.172922
H	0.614371	1.141201	-2.333056
H	0.856965	-0.748647	-1.971599
C	1.465277	-1.083437	-2.987695
H	2.481229	-0.834439	-2.684434
H	1.220049	-2.142144	-2.922344
H	1.180046	-0.645880	-3.937434
C	-2.849773	-3.187658	-3.261321
H	-2.579895	-3.615765	-2.291215
H	-3.865154	-3.509506	-3.513832
H	-2.165016	-3.584027	-4.017603
C	3.698881	1.758948	1.594883
H	3.970551	1.966780	2.630134
H	4.434787	1.079597	1.152405
H	3.708611	2.682707	1.009591
C	1.724487	-3.755651	1.135871
H	2.066703	-4.089650	2.115857
H	0.976005	-4.460363	0.757487
H	2.555983	-3.740862	0.426615
C	-3.808331	-1.739225	1.053722
H	-3.760035	-2.612379	0.396520
H	-4.195504	-2.028797	2.030959
H	-4.482760	-1.012633	0.589627
C	-1.821666	3.736877	1.517100
H	-2.700780	3.744877	0.866445
H	-2.104629	4.003352	2.535822
H	-1.115491	4.474852	1.123757

72-Rh2-Ac4-CHCO2Et-CH4_Oxy_Int-I

Charge	0
Electronic Energy, BS1 (a.u.)	-1480.157738
Thermal and entropic correction, BS1 (a.u.)	0.283103
Electronic Energy, BS2 (a.u.)	-1482.90151461
Number of Imaginary Frequencies	0
Imaginary frequencies (cm-1)	None

Molecular Geometry in Cartesian Coordinates

C	2.197274	1.214498	1.223316
C	-1.377106	2.209178	1.413924
C	-2.407171	-1.361207	1.236034
C	1.185247	-2.390516	1.188372
O	-1.058517	1.671135	2.512845
O	-1.141909	1.738025	0.247275
O	-1.882833	-1.129261	2.363627
O	-1.929067	-1.039032	0.091855
O	0.925865	-1.934357	2.338440

O	0.890619	-1.840924	0.070655
O	1.739127	0.878889	2.352793
O	1.664385	0.970200	0.082092
Rh	-0.130347	-0.036163	0.021411
Rh	-0.066611	-0.135007	2.473519
C	-0.106396	0.001336	-1.903807
C	-1.225558	-0.434931	-2.743092
O	-1.969942	0.436172	-3.167066
O	-1.339798	-1.744727	-2.920718
C	-2.529980	-2.210885	-3.628233
H	-2.737760	-1.530919	-4.458466
H	-2.235531	-3.184224	-4.024955
H	0.753437	0.424234	-2.433898
H	-0.602845	2.752595	-2.444105
C	0.249143	3.371764	-2.154248
H	-0.116122	4.343451	-1.809752
H	0.795542	2.879659	-1.347756
H	0.907812	3.522409	-3.015067
C	-3.706165	-2.314347	-2.674472
H	-3.967054	-1.329566	-2.280387
H	-4.573479	-2.720758	-3.205791
H	-3.464974	-2.974125	-1.837436
C	3.491191	1.990069	1.181778
H	3.917327	2.080186	2.181165
H	4.198027	1.491297	0.512871
H	3.296469	2.986293	0.771875
C	1.897743	-3.717385	1.092139
H	2.225697	-4.047681	2.078132
H	1.214169	-4.458367	0.665122
H	2.752715	-3.629019	0.416649
C	-3.712677	-2.119505	1.211159
H	-4.365864	-1.723116	0.431351
H	-3.504078	-3.169384	0.977456
H	-4.200833	-2.066805	2.185058
C	-2.106566	3.528612	1.442035
H	-3.090096	3.405867	0.978317
H	-2.220279	3.882578	2.466940
H	-1.552632	4.260505	0.847430

73-Rh2-Ac4-CHCO2Et-CH4_Oxy_TS

Charge	0
Electronic Energy, BS1 (a.u.)	-1480.145766
Thermal and entropic correction, BS1 (a.u.)	0.287656
Electronic Energy, BS2 (a.u.)	-1482.88830694
Number of Imaginary Frequencies	1
Imaginary frequencies (cm-1)	-223.9i

Molecular Geometry in Cartesian Coordinates

C	2.285973	0.925098	1.336274
C	-1.071149	2.508535	1.754103
C	-2.696412	-0.801749	1.135274
C	0.636890	-2.383188	0.750334
O	-0.851989	1.755806	2.746287
O	-0.896122	2.205244	0.521798
O	-2.128513	-0.837958	2.267349
O	-2.191195	-0.362860	0.046395
O	0.492224	-2.076516	1.972104
O	0.423895	-1.625535	-0.254345

O	1.781147	0.506876	2.420419
O	1.711019	0.963950	0.194682
Rh	-0.240687	0.310208	0.050940
Rh	-0.171999	-0.180958	2.441500
C	-0.232511	0.690169	-1.997553
C	-1.233734	-0.046413	-2.813991
O	-2.352626	0.328051	-3.100545
O	-0.704840	-1.249647	-3.106112
C	-1.636646	-2.304943	-3.466679
H	-2.409987	-1.889887	-4.118277
H	-1.032194	-3.016940	-4.033083
H	0.760333	0.799765	-2.435747
H	-0.705408	1.908112	-1.808537
C	-0.770887	2.786806	-2.629054
H	-1.786076	3.110601	-2.402936
H	0.006978	3.465332	-2.282265
H	-0.656089	2.484659	-3.663745
C	-2.226316	-2.928290	-2.212527
H	-2.798696	-2.183991	-1.654369
H	-2.886012	-3.759511	-2.484288
H	-1.428894	-3.298790	-1.563123
C	3.695954	1.464981	1.387422
H	4.219607	1.083520	2.265002
H	4.235182	1.203483	0.474435
H	3.650787	2.557926	1.452632
C	1.076696	-3.796553	0.447107
H	1.851587	-4.103827	1.153196
H	0.220388	-4.466961	0.580349
H	1.438755	-3.879941	-0.578337
C	-4.096363	-1.360129	1.039253
H	-4.666320	-0.831299	0.273013
H	-4.030574	-2.415015	0.749178
H	-4.598542	-1.298967	2.006078
C	-1.618633	3.889668	2.022673
H	-2.695601	3.885005	1.821102
H	-1.454755	4.170709	3.063627
H	-1.155544	4.617107	1.351874

74-Rh2-Ac4-C-Ph-CO2Et-CH4_Ethoxy_Int-I

Charge	0
Electronic Energy, BS1 (a.u.)	-1711.260691
Thermal and entropic correction, BS1 (a.u.)	0.362358
Electronic Energy, BS2 (a.u.)	-1714.0805499
Number of Imaginary Frequencies	0
Imaginary frequencies (cm-1)	None

Molecular Geometry in Cartesian Coordinates

C	2.311924	0.725585	1.326996
C	-0.945750	2.456418	1.932938
C	-2.747776	-0.764573	1.209722
C	0.503336	-2.445593	0.637521
O	-0.746305	1.644039	2.880670
O	-0.827810	2.208829	0.681477
O	-2.145316	-0.870453	2.317385
O	-2.264842	-0.293742	0.122778
O	0.422888	-2.189189	1.874770
O	0.260148	-1.641326	-0.324340
O	1.817878	0.308311	2.412751

O	1.696851	0.850478	0.210248
Rh	-0.294539	0.308857	0.068116
Rh	-0.162336	-0.293538	2.453454
C	-0.377465	0.765239	-1.883245
C	-1.329646	-0.074038	-2.644627
O	-2.464307	0.321107	-2.841541
O	-0.840346	-1.266345	-3.004623
C	-1.807742	-2.272779	-3.415982
H	-2.554159	-1.809637	-4.066544
H	-1.220000	-2.988597	-3.994309
H	1.649168	-1.399485	-2.731403
C	2.661424	-1.057764	-2.511030
H	2.621514	-0.368608	-1.666037
H	3.289440	-1.916541	-2.254564
H	3.084887	-0.552044	-3.384182
C	-2.442749	-2.909256	-2.191143
H	-2.989245	-2.157385	-1.617353
H	-3.136035	-3.699582	-2.498468
H	-1.672144	-3.338871	-1.545980
C	3.776410	1.091897	1.308532
H	4.144744	1.253048	2.322365
H	4.336100	0.267696	0.852610
H	3.934748	1.981352	0.694384
C	0.895359	-3.849819	0.241815
H	1.620572	-4.249929	0.953418
H	0.003967	-4.486382	0.275555
H	1.300617	-3.864370	-0.770895
C	-4.168897	-1.269111	1.133207
H	-4.148598	-2.317379	0.813934
H	-4.643058	-1.217084	2.114669
H	-4.737592	-0.698114	0.397143
C	-1.388027	3.857360	2.281316
H	-2.441255	3.973919	2.004757
H	-1.271981	4.042168	3.349697
H	-0.812484	4.584890	1.703148
C	0.337857	1.783169	-2.582797
C	0.992123	2.830289	-1.878966
C	0.440751	1.750432	-4.002432
C	1.712755	3.794460	-2.569836
C	1.184070	2.703268	-4.680076
C	1.817469	3.727849	-3.963625
H	0.898886	2.872704	-0.803212
H	-0.048041	0.953873	-4.554147
H	2.198923	4.599659	-2.027836
H	1.270640	2.660510	-5.761189
H	2.392000	4.479958	-4.497188

75-Rh2-Ac4-C-Ph-CO2Et-CH4_Ethoxy_TS

Charge	0
Electronic Energy, BS1 (a.u.)	-1711.223865
Thermal and entropic correction, BS1 (a.u.)	0.365384
Electronic Energy, BS2 (a.u.)	-1714.04346398
Number of Imaginary Frequencies	1
Imaginary frequencies (cm-1)	-576.8i

Molecular Geometry in Cartesian Coordinates

C	2.438208	0.408389	1.560201
C	-0.679456	2.488143	1.448562

C	-2.702389	-0.587242	0.878886
C	0.399546	-2.653513	0.979313
O	-0.680073	1.807706	2.520786
O	-0.431990	2.051764	0.276360
O	-2.275977	-0.594291	2.074593
O	-2.015784	-0.343063	-0.168123
O	0.139250	-2.224029	2.143347
O	0.430149	-1.963098	-0.094488
O	1.748031	0.183666	2.595480
O	2.010826	0.408033	0.353040
Rh	-0.003680	0.056512	0.011324
Rh	-0.279789	-0.213348	2.413499
C	0.283742	0.346939	-2.220669
C	-0.738193	-0.514190	-2.865569
O	-1.841247	-0.098575	-3.170424
O	-0.350432	-1.800179	-2.990741
C	-1.388835	-2.759936	-3.311965
H	-2.045893	-2.334925	-4.075602
H	-0.847452	-3.608435	-3.737489
H	1.262993	-0.286813	-1.862221
C	2.101082	-0.589791	-2.803742
H	2.891956	0.108676	-2.540747
H	2.233168	-1.604383	-2.430121
H	1.816239	-0.556602	-3.847673
C	-2.162483	-3.148358	-2.063201
H	-2.664383	-2.272823	-1.647539
H	-2.909930	-3.909977	-2.311118
H	-1.483799	-3.545325	-1.304154
C	3.899979	0.738365	1.744694
H	4.234934	0.459032	2.744185
H	4.499105	0.230665	0.984853
H	4.035858	1.817505	1.611897
C	0.672219	-4.133360	0.842306
H	1.332574	-4.465800	1.646872
H	-0.273780	-4.675989	0.945533
H	1.110722	-4.361241	-0.130041
C	-4.154726	-0.935028	0.657626
H	-4.233262	-2.016234	0.495926
H	-4.747031	-0.674960	1.536566
H	-4.535282	-0.427450	-0.230441
C	-1.032070	3.952426	1.550888
H	-2.101634	4.068431	1.342839
H	-0.829524	4.326670	2.555629
H	-0.479452	4.525370	0.803418
C	0.533297	1.729076	-2.685149
C	1.468245	2.534001	-2.003153
C	-0.139334	2.274405	-3.794119
C	1.725882	3.834830	-2.423148
C	0.105881	3.587316	-4.194883
C	1.041151	4.371027	-3.517666
H	1.980319	2.129874	-1.137872
H	-0.873003	1.679774	-4.323230
H	2.453165	4.437668	-1.886356
H	-0.433266	3.994918	-5.045340
H	1.233895	5.391321	-3.837158

76-Rh2-Ac4-C-Ph-CO2Et-CH4_Oxy_Int-I

Charge	0
Electronic Energy, BS1 (a.u.)	-1711.259749
Thermal and entropic correction, BS1 (a.u.)	0.361378

Electronic Energy, BS2 (a.u.)	-1714.07973407
Number of Imaginary Frequencies	0
Imaginary frequencies (cm-1)	None

Molecular Geometry in Cartesian Coordinates

C	2.495882	0.230637	1.697146
C	-0.466972	2.486049	1.391706
C	-2.657657	-0.522689	0.864664
C	0.282280	-2.730840	1.192430
O	-0.588005	1.847910	2.475286
O	-0.161573	1.991190	0.249802
O	-2.271234	-0.513978	2.069680
O	-1.929346	-0.329270	-0.168709
O	0.064677	-2.229538	2.334719
O	0.350388	-2.100492	0.083605
O	1.762217	0.120436	2.720437
O	2.111178	0.188736	0.476144
Rh	0.101402	-0.048581	0.050071
Rh	-0.269401	-0.198394	2.481612
C	0.366932	0.098012	-1.937221
C	-0.546948	-0.775630	-2.711647
O	-1.583440	-0.337296	-3.180668
O	-0.143895	-2.048095	-2.765277
C	-1.135437	-3.025519	-3.188941
H	-1.669792	-2.641270	-4.062055
H	-0.544345	-3.895055	-3.483401
H	-1.057483	3.110992	-1.735182
C	-1.474105	3.192048	-2.741037
H	-2.362728	3.830274	-2.725353
H	-0.729855	3.628232	-3.413681
H	-1.744380	2.192675	-3.088368
C	-2.081958	-3.339827	-2.042168
H	-2.631368	-2.442224	-1.749230
H	-2.796153	-4.110981	-2.350376
H	-1.519916	-3.695808	-1.175473
C	3.980811	0.411176	1.905601
H	4.200247	0.614220	2.954213
H	4.495539	-0.504950	1.597272
H	4.348798	1.224380	1.274374
C	0.449695	-4.230838	1.119119
H	-0.540247	-4.697246	1.174552
H	0.929432	-4.523478	0.184364
H	1.030680	-4.581690	1.975003
C	-4.112617	-0.821972	0.594261
H	-4.722872	-0.554414	1.458324
H	-4.450341	-0.292181	-0.298327
H	-4.218221	-1.897792	0.413448
C	-0.724845	3.973313	1.407980
H	-1.698392	4.165377	0.944273
H	-0.733334	4.350554	2.431123
H	0.032143	4.491141	0.813934
C	1.214905	0.989137	-2.660428
C	1.993010	1.967795	-1.981960
C	1.289459	0.934520	-4.082573
C	2.804744	2.838249	-2.695283
C	2.107425	1.804117	-4.784607
C	2.865325	2.757543	-4.090773
H	1.932787	2.028740	-0.905587
H	0.689250	0.208732	-4.621001
H	3.390822	3.585820	-2.169832

H	2.156598	1.752441	-5.867641
H	3.502735	3.442365	-4.643234

77-Rh2-Ac4-C-Ph-CO2Et-CH4_Oxy_TS

Charge	0
Electronic Energy, BS1 (a.u.)	-1711.221459
Thermal and entropic correction, BS1 (a.u.)	0.365199
Electronic Energy, BS2 (a.u.)	-1714.04100099
Number of Imaginary Frequencies	1
Imaginary frequencies (cm-1)	-512.2i

Molecular Geometry in Cartesian Coordinates

C	2.406609	0.473141	1.384033
C	-0.669169	2.604915	1.636455
C	-2.780742	-0.442488	1.123696
C	0.324162	-2.526754	0.888946
O	-0.618953	1.867136	2.661335
O	-0.516372	2.222534	0.423138
O	-2.230728	-0.555665	2.261213
O	-2.201653	-0.121537	0.032124
O	0.214935	-2.138523	2.093239
O	0.194879	-1.807578	-0.155316
O	1.816717	0.250450	2.486350
O	1.864469	0.518461	0.231364
Rh	-0.169759	0.214641	0.049875
Rh	-0.207219	-0.158501	2.454837
C	-0.208073	0.664678	-2.177236
C	-1.077999	-0.363878	-2.818902
O	-2.263834	-0.231204	-3.063670
O	-0.385202	-1.497145	-3.032506
C	-1.162837	-2.649280	-3.452938
H	-1.839374	-2.345753	-4.257157
H	-0.415053	-3.337695	-3.853116
H	-0.915047	1.533617	-1.700344
C	-1.428330	2.348209	-2.561304
H	-2.424394	2.282504	-2.126726
H	-0.855756	3.229830	-2.283898
H	-1.420773	2.128998	-3.620734
C	-1.923949	-3.260261	-2.288550
H	-2.649102	-2.546913	-1.891359
H	-2.457677	-4.154854	-2.628053
H	-1.235354	-3.534127	-1.487773
C	3.902702	0.674592	1.427141
H	4.200467	1.115396	2.380308
H	4.388291	-0.303382	1.333964
H	4.226728	1.297917	0.591790
C	0.606590	-3.995130	0.671048
H	-0.337108	-4.548686	0.731716
H	1.050273	-4.160680	-0.311722
H	1.265806	-4.369677	1.456964
C	-4.268469	-0.692197	1.052722
H	-4.790648	0.134204	1.546694
H	-4.601289	-0.762373	0.016588
H	-4.513534	-1.608515	1.595845
C	-0.907754	4.082275	1.836799
H	-1.672418	4.434132	1.139216
H	-1.208247	4.288797	2.864354
H	0.018391	4.623843	1.615765

C	1.069227	1.126158	-2.761286
C	1.772943	2.178654	-2.140695
C	1.610545	0.551545	-3.925081
C	2.971751	2.641737	-2.671428
C	2.829095	0.999511	-4.435188
C	3.510905	2.048484	-3.817328
H	1.372391	2.616253	-1.233114
H	1.089752	-0.260363	-4.417870
H	3.495819	3.457669	-2.181607
H	3.238429	0.533464	-5.326969
H	4.454288	2.401986	-4.223567

78-Rh2-Ac4-C-pCF3Ph-CO2CH2CF3-CH4_Ethoxy_Int-I

Charge	0
Electronic Energy, BS1 (a.u.)	-2346.006138
Thermal and entropic correction, BS1 (a.u.)	0.333204
Electronic Energy, BS2 (a.u.)	-2349.13497302
Number of Imaginary Frequencies	0
Imaginary frequencies (cm-1)	None

Molecular Geometry in Cartesian Coordinates

C	2.118609	1.288459	1.481074
C	-1.449910	2.176291	2.116565
C	-2.431947	-1.362455	1.177658
C	1.116596	-2.175929	0.606685
O	-1.084876	1.373507	3.021747
O	-1.258450	2.035609	0.856974
O	-1.834978	-1.378432	2.292617
O	-2.075665	-0.706867	0.137246
O	0.957362	-2.016634	1.852627
O	0.711213	-1.385615	-0.312914
O	1.721292	0.714488	2.534996
O	1.510710	1.311639	0.353292
Rh	-0.303842	0.342293	0.160257
Rh	-0.055758	-0.344998	2.503876
C	-0.529086	0.855276	-1.753471
C	-1.393262	-0.083925	-2.502042
O	-2.546648	0.195431	-2.759228
O	-0.799386	-1.263887	-2.767766
C	-1.643940	-2.352044	-3.136222
H	-2.689943	-2.138465	-2.910030
H	-1.533121	-2.569035	-4.201781
H	2.153709	-0.873012	-2.230361
C	2.390898	-1.234887	-3.231713
H	3.463308	-1.432743	-3.317731
H	1.827643	-2.152507	-3.409407
H	2.106207	-0.482888	-3.973262
C	3.450037	1.998515	1.520823
H	3.751474	2.189682	2.551352
H	4.201049	1.360389	1.042310
H	3.396970	2.931756	0.955399
C	1.828356	-3.418174	0.132934
H	2.385850	-3.873610	0.952151
H	1.080987	-4.125516	-0.236499
H	2.491655	-3.173778	-0.699679
C	-3.673247	-2.204779	1.019958
H	-3.420553	-3.071825	0.401436
H	-4.034811	-2.544367	1.990965

H	-4.446939	-1.635336	0.499199
C	-2.205793	3.415702	2.528300
H	-3.259543	3.291802	2.256618
H	-2.127255	3.572332	3.604561
H	-1.824020	4.284381	1.986052
C	-0.034710	2.012521	-2.434467
C	0.432566	3.134918	-1.702467
C	0.014384	2.056990	-3.854654
C	0.931842	4.246343	-2.366183
C	0.540050	3.156991	-4.511346
C	0.994963	4.250770	-3.763258
H	0.370247	3.120403	-0.623754
H	-0.349925	1.213238	-4.431569
H	1.270246	5.112587	-1.808840
H	0.586146	3.185165	-5.594147
C	1.609694	5.429757	-4.476995
F	1.449511	6.576287	-3.783895
F	2.938654	5.248484	-4.648837
F	1.070693	5.611256	-5.701131
C	-1.199599	-3.569478	-2.349798
F	0.095108	-3.873128	-2.573674
F	-1.355551	-3.408237	-1.020001
F	-1.944834	-4.630761	-2.726988

79-Rh2-Ac4-C-pCF3Ph-CO2CH2CF3-CH4_Ethoxy_TS

Charge	0
Electronic Energy, BS1 (a.u.)	-2345.975468
Thermal and entropic correction, BS1 (a.u.)	0.337905
Electronic Energy, BS2 (a.u.)	-2349.10362913
Number of Imaginary Frequencies	1
Imaginary frequencies (cm-1)	-496.6i

Molecular Geometry in Cartesian Coordinates

C	2.306155	0.983373	1.606894
C	-1.221206	2.239783	1.539161
C	-2.424799	-1.235892	0.830216
C	1.097211	-2.451275	0.853522
O	-1.058706	1.527127	2.577277
O	-0.869569	1.936374	0.350100
O	-2.007747	-1.180710	2.025824
O	-1.828603	-0.768426	-0.196976
O	0.727023	-2.160727	2.029801
O	0.961148	-1.713893	-0.181887
O	1.692225	0.549215	2.623653
O	1.893577	0.927428	0.396120
Rh	0.024897	0.114239	0.012455
Rh	-0.168884	-0.327533	2.393564
C	0.142110	0.476953	-2.204510
C	-0.904169	-0.411454	-2.761384
O	-2.009129	-0.025560	-3.083847
O	-0.539102	-1.720791	-2.754468
C	-1.595128	-2.668413	-2.864452
H	-2.538277	-2.250981	-2.509821
H	-1.707734	-3.002962	-3.899887
H	1.196732	-0.143084	-1.988723
C	1.946664	-0.476533	-2.964114
H	2.763659	0.216023	-2.768345
H	2.093872	-1.492378	-2.601610

H	1.568128	-0.424308	-3.977734
C	3.642254	1.652927	1.819790
H	4.001144	1.485056	2.835539
H	4.367595	1.277619	1.093220
H	3.528269	2.729241	1.650760
C	1.786720	-3.775936	0.635736
H	1.625036	-4.436732	1.488070
H	1.418757	-4.236248	-0.282655
H	2.862030	-3.599684	0.521210
C	-3.731473	-1.945848	0.574920
H	-3.506189	-2.965514	0.245541
H	-4.327712	-1.993385	1.487040
H	-4.285589	-1.441306	-0.219680
C	-1.922780	3.565706	1.706781
H	-2.973807	3.442236	1.422999
H	-1.874787	3.897394	2.744854
H	-1.482118	4.311713	1.041907
C	0.323387	1.848583	-2.725479
C	1.209953	2.721697	-2.064452
C	-0.366477	2.318436	-3.859260
C	1.405085	4.017666	-2.525445
C	-0.186150	3.623378	-4.307558
C	0.701717	4.472450	-3.644522
H	1.733135	2.375223	-1.181545
H	-1.065470	1.672679	-4.374224
H	2.085380	4.684841	-2.006775
H	-0.734458	3.982178	-5.171785
C	0.951773	5.859149	-4.168984
F	1.242604	6.730511	-3.176603
F	2.002093	5.881846	-5.026887
F	-0.112730	6.349245	-4.841958
C	-1.230414	-3.869912	-2.015298
F	-0.010850	-4.358268	-2.327112
F	-1.232470	-3.600618	-0.694614
F	-2.137898	-4.847263	-2.231194

80-Rh2-Ac4-C-pCF3Ph-CO2CH2CF3-CH4_Oxy_Int-I

Charge	0
Electronic Energy, BS1 (a.u.)	-2346.005354
Thermal and entropic correction, BS1 (a.u.)	0.335121
Electronic Energy, BS2 (a.u.)	-2349.1341499
Number of Imaginary Frequencies	0
Imaginary frequencies (cm-1)	None

Molecular Geometry in Cartesian Coordinates

C	2.629099	-0.662052	1.610027
C	0.492647	2.332507	2.048662
C	-2.440355	0.468592	0.694465
C	-0.231913	-2.564092	0.218553
O	0.122914	1.458382	2.883570
O	0.692319	2.150683	0.795815
O	-2.154454	-0.035893	1.816538
O	-1.612366	0.733091	-0.248317
O	-0.425152	-2.381540	1.454658
O	0.065540	-1.663246	-0.641422
O	1.811522	-0.900686	2.547880
O	2.341648	-0.188032	0.458411
Rh	0.387475	0.274854	-0.015283

Rh	-0.180636	-0.487260	2.247940
C	0.834733	0.822744	-1.883013
C	1.297634	-0.318246	-2.710059
O	0.585763	-0.806756	-3.566057
O	2.499931	-0.800353	-2.343961
C	2.822597	-2.117679	-2.789301
H	1.923030	-2.678810	-3.046788
H	3.490583	-2.078108	-3.653899
H	-2.489871	1.020424	-2.552119
C	-2.491697	0.992583	-3.643732
H	-3.422790	0.543589	-4.001640
H	-2.407825	2.008767	-4.039387
H	-1.642959	0.393395	-3.979688
C	4.092001	-0.930490	1.862399
H	4.220036	-1.519069	2.771492
H	4.526352	-1.445588	1.004582
H	4.610965	0.027353	1.976241
C	-0.328135	-3.970551	-0.317926
H	-0.853547	-4.615611	0.387076
H	-0.831854	-3.969056	-1.287333
H	0.688744	-4.348741	-0.463867
C	-3.885780	0.783741	0.398223
H	-3.967946	1.787836	-0.025832
H	-4.254641	0.077055	-0.352447
H	-4.489530	0.701828	1.302443
C	0.755960	3.729734	2.555735
H	0.332287	4.464818	1.866964
H	0.341373	3.859135	3.555941
H	1.838450	3.893164	2.590822
C	0.639633	2.086234	-2.524371
C	-0.147575	3.092304	-1.903556
C	1.203289	2.357667	-3.801387
C	-0.349312	4.312667	-2.530352
C	1.017052	3.585945	-4.413220
C	0.238685	4.560014	-3.775343
H	-0.609973	2.882916	-0.950141
H	1.786317	1.594116	-4.304972
H	-0.967331	5.073120	-2.065987
H	1.451655	3.791465	-5.384792
C	3.541936	-2.822808	-1.656793
F	2.771069	-2.957192	-0.559260
F	3.893033	-4.060667	-2.067776
F	4.665457	-2.173886	-1.290019
C	0.068774	5.916586	-4.414120
F	-1.136830	6.453438	-4.131441
F	1.005659	6.780964	-3.962863
F	0.189279	5.860950	-5.756788

81-Rh2-Ac4-C-pCF3Ph-CO2CH2CF3-CH4_Oxy_TS

Charge	0
Electronic Energy, BS1 (a.u.)	-2345.973134
Thermal and entropic correction, BS1 (a.u.)	0.338884
Electronic Energy, BS2 (a.u.)	-2349.10225214
Number of Imaginary Frequencies	1
Imaginary frequencies (cm-1)	-438.2i

Molecular Geometry in Cartesian Coordinates

C	2.526688	0.097629	0.926491
C	-0.236071	2.466307	1.751070
C	-2.735821	-0.241946	1.140289
C	0.046753	-2.626616	0.387484
O	-0.091860	1.625020	2.687340
O	-0.325302	2.200701	0.504997
O	-2.097277	-0.464400	2.210143
O	-2.226968	0.068800	0.007018
O	0.061016	-2.358303	1.625238
O	-0.052987	-1.781228	-0.563698
O	2.031863	-0.270947	2.037113
O	1.874109	0.406379	-0.124710
Rh	-0.174473	0.239688	-0.126851
Rh	-0.019144	-0.381922	2.217437
C	-0.214381	0.721701	-2.314131
C	0.129696	-0.569200	-2.976581
O	-0.641966	-1.355734	-3.481967
O	1.461363	-0.796881	-2.846176
C	1.893905	-2.138486	-3.045455
H	1.111261	-2.847144	-2.773276
H	2.193862	-2.300135	-4.085000
H	-1.435714	0.749858	-2.126038
C	-2.209665	0.924149	-3.127634
H	-2.842440	0.064540	-2.919525
H	-2.613409	1.887711	-2.821614
H	-1.800466	0.919405	-4.129593
C	4.028363	0.207618	0.826792
H	4.507565	-0.293921	1.668362
H	4.363496	-0.221604	-0.118993
H	4.306220	1.267050	0.834015
C	0.191516	-4.073319	-0.018138
H	-0.091019	-4.732790	0.803542
H	-0.416187	-4.281240	-0.901764
H	1.241789	-4.253875	-0.269558
C	-4.240490	-0.362880	1.176762
H	-4.694118	0.532488	0.741645
H	-4.543445	-1.218724	0.564107
H	-4.592508	-0.504534	2.199151
C	-0.328330	3.927077	2.121517
H	-1.344532	4.283498	1.918648
H	-0.096988	4.074367	3.177202
H	0.357722	4.506580	1.496992
C	0.384434	2.007632	-2.727879
C	-0.139176	3.215957	-2.226940
C	1.487303	2.066997	-3.600344
C	0.416362	4.436935	-2.584668
C	2.060370	3.289579	-3.937019
C	1.522077	4.476459	-3.438411
H	-0.968911	3.189827	-1.533488
H	1.916229	1.157100	-3.998339
H	-0.001947	5.359878	-2.198600
H	2.913949	3.321970	-4.605193
C	3.102609	-2.363972	-2.158442
F	2.801053	-2.337959	-0.844575
F	3.617702	-3.583855	-2.431413
F	4.066995	-1.446447	-2.370884
C	2.171602	5.789237	-3.774597
F	1.300112	6.821593	-3.698329
F	3.191567	6.074275	-2.928883
F	2.691455	5.788806	-5.024005

82-Rh2-Ac4-CHCF3-CH4_Int-I

Charge	0
Electronic Energy, BS1 (a.u.)	-1549.978836
Thermal and entropic correction, BS1 (a.u.)	0.220149
Electronic Energy, BS2 (a.u.)	-1552.77514851
Number of Imaginary Frequencies	0
Imaginary frequencies (cm-1)	None

Molecular Geometry in Cartesian Coordinates

C	2.386825	1.214532	1.520598
C	-1.107524	2.416508	1.363402
C	-2.306907	-1.154078	1.130316
C	1.202829	-2.322298	1.267473
O	-0.966854	1.833029	2.475785
O	-0.763058	1.958891	0.216497
O	-1.879031	-0.940719	2.300791
O	-1.716731	-0.839648	0.037766
O	0.875959	-1.859904	2.398191
O	1.031812	-1.744021	0.136315
O	1.794807	0.908624	2.594403
O	1.951940	1.002931	0.332427
Rh	0.129713	0.094665	0.073500
Rh	-0.052133	-0.016871	2.516256
C	0.004143	0.305125	-1.813735
C	3.727969	1.897661	1.607928
H	4.058787	1.960113	2.644811
H	4.458037	1.345491	1.009618
H	3.646625	2.903490	1.183938
C	1.848654	-3.683151	1.207138
H	2.060685	-4.051513	2.211084
H	1.172637	-4.375156	0.694811
H	2.769109	-3.626017	0.619862
C	-3.644520	-1.831813	0.969919
H	-3.543950	-2.683957	0.292399
H	-4.026387	-2.158537	1.937407
H	-4.348073	-1.128475	0.513210
C	-1.731288	3.790084	1.352173
H	-2.586763	3.797929	0.670871
H	-2.046537	4.075429	2.355961
H	-1.001105	4.512035	0.973312
H	0.072295	1.301926	-2.262911
C	-0.580834	-0.688649	-2.777484
F	-1.890917	-0.404099	-2.963232
F	0.042728	-0.590595	-3.977503
F	-0.483827	-1.961485	-2.374437
C	2.986328	-0.739512	-2.449653
H	2.425883	-1.538008	-1.959439
H	4.002965	-1.082403	-2.665067
H	3.025246	0.122551	-1.779854
H	2.497218	-0.466021	-3.387733

83-Rh2-Ac4-CHCF3-CH4_TS

Charge	0
Electronic Energy, BS1 (a.u.)	-1549.967954
Thermal and entropic correction, BS1 (a.u.)	0.225001
Electronic Energy, BS2 (a.u.)	-1552.76374477
Number of Imaginary Frequencies	2

Imaginary frequencies (cm-1)	-285.4i, -10.7i

Molecular Geometry in Cartesian Coordinates

C	2.426502	1.273551	1.717863
C	-1.125848	2.309199	1.306477
C	-2.146771	-1.261442	1.018738
C	1.412165	-2.317866	1.436914
O	-1.012792	1.761252	2.443952
O	-0.685925	1.854374	0.196825
O	-1.804726	-1.032568	2.218196
O	-1.487202	-0.954687	-0.030866
O	0.983213	-1.860558	2.534559
O	1.298610	-1.758952	0.289311
O	1.778513	0.956398	2.758356
O	2.089866	1.025845	0.508768
Rh	0.306558	0.041841	0.165796
Rh	-0.027160	-0.045725	2.578672
C	0.618257	0.221244	-1.887886
C	3.713329	2.043190	1.901170
H	4.047605	1.991756	2.937916
H	4.483574	1.652570	1.231636
H	3.538264	3.090801	1.632938
C	2.101237	-3.661089	1.451503
H	2.398512	-3.928268	2.466104
H	1.404681	-4.417288	1.072990
H	2.970554	-3.646209	0.789669
C	-3.474329	-1.942447	0.785892
H	-3.369640	-2.710035	0.015304
H	-3.851949	-2.377766	1.711756
H	-4.190606	-1.199358	0.418937
C	-1.854416	3.629973	1.231069
H	-2.737293	3.517087	0.594284
H	-2.154847	3.963115	2.224796
H	-1.206194	4.377448	0.764383
H	1.181616	1.110220	-2.175417
C	-0.484104	-0.082990	-2.876080
F	-1.535752	0.697133	-2.575737
F	-0.155009	0.182153	-4.173558
F	-0.862944	-1.372724	-2.833610
C	2.177371	-1.082981	-2.779278
H	1.414705	-0.779502	-1.859901
H	3.128654	-0.846270	-2.305865
H	2.032816	-0.598517	-3.737418
H	1.935317	-2.145411	-2.797165

TpBrCF3Ag-THF

Charge	0
Electronic Energy, BS1 (a.u.)	-3140.640053
Thermal and entropic correction, BS1 (a.u.)	0.223335
Electronic Energy, BS2 (a.u.)	-10826.217689
Number of Imaginary Frequencies	0
Imaginary frequencies (cm-1)	None

Molecular Geometry in Cartesian Coordinates

Ag	4.423650	-0.370995	11.144624
Br	7.602715	0.048917	5.769650

Br	-1.307104	0.738035	8.885354
Br	5.959848	5.102973	13.673385
F	5.879729	-2.582504	6.856703
F	4.874029	-2.528207	8.790005
F	7.027719	-2.243746	8.671340
F	7.010393	3.079191	6.044694
F	6.303599	3.898058	7.932375
F	4.904210	3.554255	6.300903
F	-0.455038	-0.309549	11.951355
F	1.628052	-0.776456	12.390969
F	0.603235	-1.789777	10.761015
F	0.130700	2.737274	7.006424
F	2.278855	2.577198	6.702556
F	1.473281	4.135798	7.990968
F	5.884262	1.740943	14.606755
F	7.759868	2.150347	13.588691
F	6.607149	0.376793	13.070647
F	4.186522	6.289635	11.430950
F	2.818113	5.037268	10.295035
F	4.734408	5.614385	9.438963
N	4.784680	0.129186	8.719332
N	4.839307	1.439794	8.428540
N	2.432600	0.923458	10.501975
N	2.580968	1.807860	9.501013
N	5.281480	1.868070	11.288330
N	4.660113	2.812427	10.557734
C	5.648335	-0.486687	7.916295
C	6.294333	0.442330	7.076696
C	5.745332	1.669408	7.438223
C	5.856505	-1.963236	8.048161
C	5.993593	3.056632	6.920090
C	1.183759	0.469246	10.443197
C	0.489725	1.066893	9.372600
C	1.423477	1.920472	8.792748
C	0.731311	-0.598497	11.391273
C	1.320299	2.849542	7.617062
C	5.768647	2.466560	12.372552
C	5.461276	3.840408	12.358263
C	4.744865	4.020658	11.179082
C	6.509579	1.683098	13.413854
C	4.121527	5.251380	10.583038
B	3.962085	2.461322	9.211474
H	3.820461	3.440811	8.570213
O	4.576795	-2.544062	11.837806
C	5.821239	-3.297079	11.735541
H	6.266728	-3.341373	12.736161
H	6.497235	-2.764244	11.063274
C	5.419921	-4.677031	11.217883
H	5.406505	-4.675076	10.123754
H	6.103033	-5.459721	11.556739
C	3.993096	-4.831832	11.767596
H	4.016357	-5.112863	12.826131
H	3.400345	-5.573433	11.226095
C	3.438582	-3.421081	11.604867
H	2.661770	-3.151280	12.322685
H	3.061778	-3.248682	10.589849

TpBrCF3Ag

Charge	0

Electronic Energy, BS1 (a.u.)	-2908.132033
Thermal and entropic correction, BS1 (a.u.)	0.113988
Electronic Energy, BS2 (a.u.)	-10593.631532
Number of Imaginary Frequencies	0
Imaginary frequencies (cm-1)	None

Molecular Geometry in Cartesian Coordinates

```
Ag          4.527924     -0.889697     10.740278
Br          7.648433      0.380047      5.445260
Br         -1.301172      0.579222      8.929853
Br          5.875401      4.258727     14.078818
F           5.864051     -2.510849      6.394792
F           5.551494     -2.651406      8.546362
F           7.528059     -2.189192      7.754209
F           6.878192      3.306529      6.066475
F           6.240644      3.877443      8.066053
F           4.769503      3.628189      6.481047
F          -0.131923     -0.801054     11.979898
F           1.839108     -1.706762     11.777351
F           0.278947     -2.140687     10.319637
F           0.114642      2.743702      7.238386
F           2.246055      2.601926      6.827692
F           1.515438      3.989617      8.337154
F           5.859885      0.881050     14.621696
F           7.739642      1.263227     13.602062
F           6.470268     -0.385923     12.958000
F           4.179104      5.705585     11.942643
F           2.810150      4.578974     10.682908
F           4.715621      5.264019      9.883735
N           5.020779     -0.046460      8.527934
N           4.947607      1.287783      8.367870
N           2.523877      0.407756     10.318847
N           2.653844      1.437114      9.461694
N           5.307752      1.339224     11.295456
N           4.696535      2.358756     10.664265
C           5.890268     -0.506571      7.634078
C           6.404277      0.549026      6.857297
C           5.778056      1.684495      7.363049
C           6.211638     -1.969027      7.575078
C           5.916298      3.133026      6.985513
C           1.262442     -0.005956     10.253903
C           0.534450      0.772681      9.334297
C           1.464998      1.683794      8.843138
C           0.803539     -1.165203     11.085287
C           1.329311      2.764931      7.807679
C           5.748076      1.801333     12.461411
C           5.427390      3.164739     12.604764
C           4.747597      3.482924     11.432817
C           6.460309      0.892502     13.416841
C           4.114214      4.769933     10.983353
B           4.023229      2.158958      9.272195
H           3.849390      3.207474      8.763776
```

TpBrCF3_2Ag-Int-I

Charge	0
Electronic Energy, BS1 (a.u.)	-3393.938410

Thermal and entropic correction, BS1 (a.u.)	0.144842
Electronic Energy, BS2 (a.u.)	-11079.654818
Number of Imaginary Frequencies	0
Imaginary frequencies (cm-1)	None

Molecular Geometry in Cartesian Coordinates

Ag	4.562050	-0.860850	10.672375
Br	7.700577	0.655194	5.393872
Br	-1.252203	0.407675	8.999953
Br	5.878092	4.171903	14.069637
F	6.136098	-2.292843	6.218516
F	5.580765	-2.529075	8.312164
F	7.606367	-1.938962	7.779939
F	6.778080	3.545984	6.022599
F	6.102731	4.056737	8.026649
F	4.653781	3.769142	6.428063
F	-0.205919	-1.443701	11.365956
F	1.766312	-1.246568	12.272870
F	1.527854	-2.384745	10.438648
F	-0.007551	2.675232	7.331378
F	2.116843	2.664779	6.866663
F	1.349055	3.967128	8.432950
F	5.830961	0.088230	13.824517
F	7.553507	1.418267	13.919171
F	7.367298	-0.025337	12.294944
F	4.014041	5.634651	12.081962
F	2.686489	4.483647	10.798974
F	4.509196	5.350247	9.983866
N	5.003983	0.078208	8.397559
N	4.896234	1.411771	8.258180
N	2.614450	0.433612	10.307869
N	2.660276	1.468575	9.453001
N	5.460665	1.428194	11.074003
N	4.729889	2.430566	10.564309
C	5.907881	-0.335160	7.514231
C	6.409711	0.752141	6.771213
C	5.736132	1.856605	7.282202
C	6.305575	-1.777791	7.448115
C	5.818648	3.314907	6.931728
C	1.372009	-0.045908	10.292692
C	0.575740	0.689175	9.393821
C	1.441740	1.652619	8.877589
C	1.096207	-1.281223	11.098306
C	1.217586	2.748256	7.872607
C	5.930069	1.837578	12.249824
C	5.491550	3.147855	12.527601
C	4.724690	3.492524	11.415930
C	6.683387	0.846180	13.079716
C	3.983344	4.752454	11.071908
B	3.988719	2.245589	9.205774
H	3.753718	3.297083	8.727325
C	5.634137	-2.580602	11.782628
H	5.742953	-2.169710	12.784512
N	6.832443	-2.722494	11.176969
N	7.804290	-2.786967	10.616487
F	4.608290	-4.174910	10.378434
C	4.762792	-3.810537	11.666404
F	3.557599	-3.535346	12.190929
F	5.264157	-4.878202	12.325626

TpBrCF3_2Ag-TS_I-II

Charge	0
Electronic Energy, BS1 (a.u.)	-3393.899710
Thermal and entropic correction, BS1 (a.u.)	0.138376
Electronic Energy, BS2 (a.u.)	-11079.624220
Number of Imaginary Frequencies	1
Imaginary frequencies (cm-1)	-15.3i

Molecular Geometry in Cartesian Coordinates

Ag	4.405899	-0.942757	10.711573
Br	7.531949	0.312556	5.382950
Br	-1.258312	0.596662	8.940038
Br	5.774713	4.284648	14.133442
F	5.706939	-2.453719	6.283584
F	5.387315	-2.708018	8.421554
F	7.383875	-2.295056	7.656312
F	6.929895	3.264889	6.091092
F	6.252931	3.861259	8.070206
F	4.814792	3.608404	6.455509
F	-0.361927	-1.330236	11.272645
F	1.588741	-1.252227	12.239057
F	1.342819	-2.356896	10.384510
F	0.124838	2.784620	7.297954
F	2.252002	2.652502	6.867093
F	1.530787	4.020861	8.398606
F	5.682184	0.686849	14.504209
F	7.605684	1.492787	13.892397
F	6.774442	-0.237334	12.864634
F	4.153072	5.748614	11.948702
F	2.775038	4.597307	10.721097
F	4.663867	5.287635	9.887087
N	5.013211	-0.043037	8.566724
N	4.980457	1.293983	8.407259
N	2.583865	0.408430	10.300336
N	2.692953	1.457219	9.468218
N	5.349796	1.392359	11.287324
N	4.719917	2.407792	10.669993
C	5.825104	-0.531489	7.629768
C	6.348778	0.510443	6.841823
C	5.785945	1.665045	7.376489
C	6.075767	-2.004454	7.496683
C	5.946686	3.109524	6.990266
C	1.317978	-0.010663	10.261917
C	0.574396	0.778832	9.365551
C	1.495018	1.706915	8.877515
C	0.953880	-1.239274	11.045044
C	1.343652	2.802982	7.856780
C	5.767391	1.852998	12.462883
C	5.402843	3.205052	12.626942
C	4.731554	3.524385	11.451914
C	6.463371	0.951016	13.434636
C	4.082258	4.801100	11.001911
B	4.059635	2.191061	9.284713
H	3.878678	3.225930	8.752109
C	5.051428	-2.541377	11.774789
H	5.700409	-2.514221	12.656292
N	7.664498	-1.832996	10.602206

N	8.065176	-0.880389	10.212849
F	4.036733	-4.141215	10.331111
C	4.834615	-3.978996	11.394757
F	4.259685	-4.605288	12.445433
F	6.022317	-4.553808	11.118643

TpBrCF3_2Ag-Int-II

Charge	0
Electronic Energy, BS1 (a.u.)	-3393.899781
Thermal and entropic correction, BS1 (a.u.)	0.137944
Electronic Energy, BS2 (a.u.)	-11079.624459
Number of Imaginary Frequencies	1
Imaginary frequencies (cm-1)	-16.4i

Molecular Geometry in Cartesian Coordinates

Ag	4.363049	-0.953993	10.732617
Br	7.529709	0.224249	5.432854
Br	-1.271540	0.630914	8.923013
Br	5.769707	4.293090	14.137719
F	5.686254	-2.501528	6.300599
F	5.237320	-2.747616	8.416106
F	7.284255	-2.382962	7.768789
F	6.986162	3.191549	6.138164
F	6.291147	3.803055	8.106605
F	4.872159	3.573414	6.471621
F	-0.412437	-1.277995	11.290268
F	1.545065	-1.239979	12.245309
F	1.266909	-2.334972	10.389387
F	0.148456	2.792642	7.274722
F	2.279400	2.641330	6.868580
F	1.551646	4.026387	8.381883
F	5.641033	0.713533	14.547362
F	7.576085	1.481622	13.923974
F	6.720136	-0.251623	12.922800
F	4.175707	5.755258	11.932329
F	2.792371	4.608358	10.706501
F	4.692675	5.272345	9.877242
N	4.965683	-0.074791	8.585524
N	4.969336	1.262772	8.430031
N	2.556401	0.417569	10.319571
N	2.682327	1.459947	9.481053
N	5.328280	1.380406	11.314572
N	4.714222	2.397839	10.684515
C	5.772449	-0.582820	7.654500
C	6.332252	0.447258	6.876134
C	5.793891	1.614991	7.408326
C	5.994984	-2.061663	7.533330
C	5.987536	3.055471	7.023136
C	1.286822	0.010604	10.272345
C	0.558843	0.800375	9.363556
C	1.492433	1.716263	8.877529
C	0.903661	-1.212157	11.055074
C	1.361081	2.806092	7.847076
C	5.743763	1.846972	12.488787
C	5.393843	3.204508	12.638611
C	4.733627	3.521107	11.456803
C	6.425886	0.949264	13.474157

C	4.100139	4.800880	10.992724
B	4.057328	2.177982	9.298708
H	3.890666	3.211030	8.757888
C	5.057639	-2.553226	11.761674
H	5.740723	-2.523287	12.617084
N	7.729863	-1.513327	10.516421
N	8.002683	-0.499412	10.174120
F	3.950848	-4.171135	10.409700
C	4.911783	-3.982008	11.323263
F	4.652336	-4.792192	12.367649
F	6.098396	-4.321387	10.769443

TpBrCF3Ag-Int-III

Charge	0
Electronic Energy, BS1 (a.u.)	-3325.036388
Thermal and entropic correction, BS1 (a.u.)	0.186913
Electronic Energy, BS2 (a.u.)	-11010.712552
Number of Imaginary Frequencies	0
Imaginary frequencies (cm-1)	None

Molecular Geometry in Cartesian Coordinates

Ag	4.770107	-1.203923	10.130550
Br	7.614476	1.043772	5.031683
Br	-1.237756	0.376083	8.835066
Br	6.186187	3.512917	14.106897
F	5.973441	-2.041648	5.543369
F	5.781238	-2.547417	7.653300
F	7.689090	-1.853839	6.863315
F	6.745094	3.790111	6.140592
F	6.146519	4.001896	8.220712
F	4.638787	3.943869	6.651338
F	0.026517	-2.294241	10.008157
F	0.732620	-1.466212	11.892091
F	2.112392	-2.525861	10.586466
F	0.053283	2.786897	7.443394
F	2.167878	2.788826	6.938787
F	1.459852	3.885676	8.680966
F	6.461286	0.133116	14.164526
F	8.177993	0.760292	12.990027
F	6.920840	-0.876055	12.289804
F	4.180197	5.105375	12.381882
F	2.800297	4.088651	11.042959
F	4.593205	5.035536	10.249922
N	5.110713	0.000760	8.071117
N	4.974036	1.339078	8.129724
N	2.665862	0.056633	9.990101
N	2.723650	1.220145	9.318686
N	5.511433	0.977551	10.987786
N	4.791284	2.034057	10.568481
C	5.970524	-0.266761	7.092717
C	6.411818	0.923428	6.484070
C	5.753971	1.931384	7.182317
C	6.355992	-1.681696	6.781682
C	5.819940	3.426642	7.041527
C	1.413245	-0.388140	9.905655
C	0.617671	0.514658	9.173282
C	1.500610	1.526743	8.803531

C	1.056786	-1.671192	10.593698
C	1.288768	2.757458	7.965038
C	6.037218	1.301428	12.165793
C	5.657235	2.605954	12.535484
C	4.852684	3.039598	11.486227
C	6.907700	0.329602	12.902104
C	4.107662	4.329942	11.289238
B	4.057494	2.015780	9.192552
H	3.822707	3.121677	8.861713
C	3.587250	-1.651633	13.512289
H	4.441484	-1.067246	13.860711
F	3.153418	-3.853780	12.716721
C	4.119126	-3.004814	13.114473
F	4.800208	-3.599765	14.111097
F	5.004571	-2.907414	12.056222
C	2.503942	-1.758849	14.586627
H	1.644671	-2.320173	14.212710
H	2.162883	-0.760811	14.872965
H	3.188787	-1.171890	12.614978
H	2.887097	-2.259340	15.480477

TpF27-Int-I

Charge	0
Electronic Energy, BS1 (a.u.)	-4710.143248
Thermal and entropic correction, BS1 (a.u.)	0.231111
Electronic Energy, BS2 (a.u.)	-4713.433952
Number of Imaginary Frequencies	0
Imaginary frequencies (cm-1)	None

Molecular Geometry in Cartesian Coordinates

Ag	-0.251801	-0.060386	0.866088
C	-2.589462	-0.184259	1.175525
N	1.254582	1.904550	0.274116
N	2.556951	1.571684	0.146902
N	1.710768	-1.264246	0.076083
N	2.944171	-0.897000	0.494986
N	1.361339	0.162710	2.766532
N	2.594983	0.638965	2.492286
B	3.214884	0.514697	1.074994
C	0.961245	2.806690	-0.651119
C	2.095284	3.083473	-1.459750
C	3.107874	2.255216	-0.904368
C	1.768268	-2.511102	-0.365394
C	3.084671	-3.026800	-0.232207
C	3.814295	-1.942076	0.325893
C	1.038990	0.508844	4.002569
C	2.081666	1.269376	4.596770
C	3.071230	1.329558	3.577943
H	4.380249	0.703702	1.130461
C	4.273609	2.026492	3.797363
C	4.453980	2.637897	5.022536
C	2.288624	1.901632	5.833285
C	3.471288	2.577842	6.039012
C	4.399702	2.236521	-1.460036
C	4.646843	3.050333	-2.547595
C	2.371314	3.899702	-2.568616
C	3.642079	3.880712	-3.101119

C	5.187598	-2.077963	0.604362
C	5.787455	-3.289269	0.323398
C	3.718807	-4.251021	-0.503911
C	5.062520	-4.372928	-0.226831
F	5.371396	1.444413	-0.983899
F	5.857374	3.059982	-3.116434
F	3.955271	4.636035	-4.161872
F	1.420772	4.672096	-3.110796
F	1.355962	1.862114	6.794173
F	3.716021	3.200372	7.199169
F	5.578569	3.317854	5.271315
F	5.227410	2.129136	2.861441
F	5.917552	-1.091245	1.141800
F	7.088141	-3.465918	0.579490
F	5.713155	-5.519264	-0.463397
F	3.040060	-5.286642	-1.012441
C	0.524502	-3.223365	-0.788831
F	-0.447249	-2.332871	-1.147238
F	0.765234	-4.040673	-1.846701
C	-0.421622	3.361134	-0.735625
F	-1.070342	3.185203	0.454849
F	-0.392751	4.690014	-1.020685
C	-0.316599	0.189845	4.540418
F	-0.882868	-0.845053	3.842848
F	-0.259014	-0.160803	5.850699
C	-0.059322	-4.100686	0.345114
F	-0.297769	-3.338259	1.431069
F	-1.201021	-4.681902	-0.033736
F	0.824929	-5.051685	0.681840
C	-1.308211	1.376637	4.439671
F	-2.539481	0.998161	4.809676
F	-0.901871	2.386311	5.213218
F	-1.379369	1.817986	3.161689
C	-1.300255	2.693705	-1.819383
F	-0.728505	2.822700	-3.021593
F	-1.441134	1.375548	-1.549572
F	-2.521145	3.241488	-1.848943
H	-2.605811	-0.439752	2.233198
N	-3.544329	2.052849	0.736855
N	-3.137425	1.030322	0.961203
C	-3.177553	-1.229578	0.253096
F	-2.876266	-0.977840	-1.035052
F	-2.687646	-2.429976	0.592806
F	-4.527403	-1.297786	0.333674

TpF27-TS-II-III

Charge	0
Electronic Energy, BS1 (a.u.)	-4710.099974
Thermal and entropic correction, BS1 (a.u.)	0.223677
Electronic Energy, BS2 (a.u.)	-4713.401758
Number of Imaginary Frequencies	1
Imaginary frequencies (cm-1)	-18.0i

Molecular Geometry in Cartesian Coordinates

Ag	-0.341866	-0.033367	0.774905
C	-2.287058	-0.225594	1.287286
N	1.007263	1.654286	-0.169173

N	2.341188	1.686440	0.063752
N	1.556208	-1.198974	0.114080
N	2.781555	-0.770234	0.495571
N	1.300266	0.180006	2.794023
N	2.454513	0.794059	2.456898
B	3.025934	0.675586	1.025515
C	0.732273	2.437878	-1.203307
C	1.923917	3.024828	-1.705482
C	2.938132	2.520792	-0.845706
C	1.641344	-2.457202	-0.289067
C	2.973604	-2.924872	-0.151676
C	3.677433	-1.797901	0.356724
C	1.110909	0.326951	4.094893
C	2.169573	1.082535	4.672771
C	3.015621	1.369287	3.567891
H	4.186003	0.886454	1.041978
C	4.183341	2.134112	3.737660
C	4.485991	2.575617	5.010798
C	2.503046	1.539838	5.958144
C	3.655336	2.279506	6.118130
C	4.279020	2.917543	-1.016928
C	4.570843	3.772237	-2.060957
C	2.252315	3.884343	-2.769428
C	3.568831	4.252027	-2.937064
C	5.055240	-1.887810	0.632521
C	5.685012	-3.093492	0.396287
C	3.637119	-4.144241	-0.372741
C	4.985580	-4.218350	-0.102943
F	5.268037	2.501233	-0.215999
F	5.830409	4.168998	-2.265971
F	3.932743	5.067339	-3.934069
F	1.320227	4.335466	-3.616261
F	1.721262	1.273349	7.012503
F	4.013058	2.742861	7.323082
F	5.582331	3.312087	5.222691
F	4.982283	2.456756	2.709878
F	5.761013	-0.862954	1.129717
F	6.990092	-3.225879	0.652698
F	5.664049	-5.356494	-0.291904
F	2.979691	-5.220044	-0.820350
C	0.422319	-3.199198	-0.742964
F	-0.532525	-2.333464	-1.194761
F	0.734581	-4.054073	-1.750575
C	-0.644731	2.497013	-1.789736
F	-1.596798	2.224960	-0.850645
F	-0.900158	3.727146	-2.301784
C	-0.080135	-0.282346	4.761117
F	-0.655332	-1.219624	3.951845
F	0.285136	-0.878976	5.928885
C	-0.230068	-4.035148	0.382904
F	-0.602377	-3.224372	1.393273
F	-1.310162	-4.680553	-0.066749
F	0.653140	-4.927604	0.853372
C	-1.196071	0.727885	5.107308
F	-2.202953	0.125876	5.748010
F	-0.720251	1.719122	5.869918
F	-1.680447	1.262967	3.965173
C	-0.842605	1.468140	-2.929876
F	0.019566	1.730053	-3.924421
F	-0.603733	0.225958	-2.469112
F	-2.089933	1.518771	-3.404477
H	-2.702669	-0.217099	2.299510

N	-1.580641	3.436081	1.890992
N	-2.402433	2.716900	1.728963
C	-3.419584	-0.339024	0.307838
F	-3.046722	-0.197170	-0.972262
F	-3.907024	-1.594095	0.467955
F	-4.394339	0.556324	0.553808

TpF27-Int-II

Charge	0
Electronic Energy, BS1 (a.u.)	-4710.104268
Thermal and entropic correction, BS1 (a.u.)	0.221879
Electronic Energy, BS2 (a.u.)	-4713.405719
Number of Imaginary Frequencies	0
Imaginary frequencies (cm-1)	None

Molecular Geometry in Cartesian Coordinates

Ag	-0.285863	-0.367130	0.691687
C	-2.214844	-0.703266	1.185269
N	1.011222	1.432928	-0.072541
N	2.359192	1.423374	0.038671
N	1.637616	-1.476747	0.028102
N	2.851492	-1.041969	0.438035
N	1.336690	-0.019666	2.724852
N	2.520852	0.537534	2.392572
B	3.087323	0.402160	0.958747
C	0.652624	2.401990	-0.902076
C	1.800831	3.077713	-1.390860
C	2.883308	2.418677	-0.743621
C	1.737894	-2.740324	-0.357697
C	3.067227	-3.202062	-0.180331
C	3.753592	-2.068186	0.334862
C	1.025038	0.363167	3.953271
C	2.023069	1.231179	4.476643
C	2.972411	1.320680	3.423312
H	4.243823	0.633457	0.955743
C	4.097121	2.155728	3.541012
C	4.250867	2.869797	4.712525
C	2.202935	1.968545	5.657954
C	3.313155	2.777660	5.768852
C	4.208786	2.840625	-0.964867
C	4.414734	3.886676	-1.841679
C	2.041864	4.130392	-2.291895
C	3.342598	4.528458	-2.506453
C	5.123814	-2.150238	0.648708
C	5.763697	-3.355671	0.440740
C	3.740918	-4.420797	-0.373524
C	5.081719	-4.487485	-0.066954
F	5.261267	2.265025	-0.370404
F	5.655999	4.316504	-2.087548
F	3.625679	5.524362	-3.354482
F	1.040254	4.732910	-2.942345
F	1.311289	1.906569	6.655368
F	3.526493	3.504489	6.873249
F	5.300025	3.684317	4.868583
F	4.990342	2.297753	2.550476
F	5.812069	-1.119061	1.156300
F	7.061462	-3.481385	0.734027

F	5.768781	-5.624636	-0.227373
F	3.100158	-5.502801	-0.830168
C	0.539876	-3.497301	-0.841465
F	-0.417190	-2.644249	-1.311288
F	0.888546	-4.342552	-1.845482
C	-0.787063	2.612986	-1.258654
F	-1.608086	2.185227	-0.254603
F	-1.038118	3.928663	-1.474835
C	-0.276202	-0.040554	4.564644
F	-0.788701	-1.140292	3.935621
F	-0.122095	-0.329046	5.884569
C	-0.127866	-4.351909	0.262025
F	-0.546823	-3.555143	1.266234
F	-1.182396	-5.014218	-0.222902
F	0.756649	-5.230013	0.754584
C	-1.363403	1.058616	4.473640
F	-2.485005	0.670034	5.089921
F	-0.925142	2.195850	5.028323
F	-1.658510	1.305732	3.177964
C	-1.196427	1.845229	-2.538496
F	-0.504329	2.324154	-3.583647
F	-0.908418	0.537294	-2.397413
F	-2.503470	1.975162	-2.778869
H	-2.589535	-0.891165	2.196049
N	0.992196	3.637368	2.601442
N	0.027168	3.396180	2.120361
C	-3.391889	-0.590148	0.260835
F	-3.058378	-0.480566	-1.032368
F	-4.197569	-1.664427	0.387670
F	-4.065719	0.527145	0.616155

TpF27Ag-Int-III

Charge	0
Electronic Energy, BS1 (a.u.)	-4600.574785
Thermal and entropic correction, BS1 (a.u.)	0.224686
Electronic Energy, BS2 (a.u.)	-4603.830093
Number of Imaginary Frequencies	1
Imaginary frequencies (cm-1)	-8.6i

Molecular Geometry in Cartesian Coordinates

Ag	-0.353224	0.046311	0.587828
C	-2.024453	-1.081438	0.299851
N	0.995930	1.790165	-0.165830
N	2.335682	1.602626	-0.215555
N	1.807860	-1.267888	-0.229980
N	2.955776	-0.803337	0.308000
N	1.193928	0.277544	2.409826
N	2.431749	0.773897	2.181497
B	3.080087	0.666411	0.774097
C	0.660971	2.720098	-1.049877
C	1.807280	3.166772	-1.753704
C	2.868418	2.414836	-1.180078
C	1.905348	-2.584796	-0.340905
C	3.155405	-3.042613	0.160522
C	3.800534	-1.848412	0.579732
C	0.894425	0.446606	3.689056
C	1.960332	1.098262	4.363087

C	2.940321	1.279307	3.348107
H	4.216002	0.984471	0.836309
C	4.176286	1.881658	3.649911
C	4.388428	2.303703	4.947072
C	2.197248	1.550862	5.672866
C	3.407456	2.144736	5.955334
C	4.187877	2.579532	-1.641867
C	4.407830	3.488998	-2.656637
C	2.057279	4.081690	-2.791366
C	3.352858	4.239772	-3.230355
C	5.071741	-1.893198	1.179583
C	5.671295	-3.127945	1.331685
C	3.789465	-4.285776	0.321672
C	5.038891	-4.319737	0.903166
F	5.214461	1.870996	-1.153016
F	5.642240	3.670413	-3.135356
F	3.645395	5.093269	-4.218725
F	1.063378	4.777239	-3.355713
F	1.271060	1.423455	6.629995
F	3.682427	2.594358	7.185906
F	5.543635	2.888800	5.278520
F	5.131171	2.068769	2.729424
F	5.694105	-0.790031	1.621437
F	6.875814	-3.222983	1.905531
F	5.681012	-5.480961	1.085862
F	3.195598	-5.421794	-0.066113
C	0.749753	-3.387003	-0.838527
F	-0.119002	-2.596269	-1.538103
F	1.164162	-4.394629	-1.651570
C	-0.761039	3.161962	-1.190808
F	-1.452598	2.888457	-0.046169
F	-0.818331	4.500277	-1.417015
C	-0.443546	0.032996	4.216530
F	-0.965890	-0.976421	3.460020
F	-0.338040	-0.407858	5.496493
C	-0.072878	-4.048295	0.297082
F	-0.479849	-3.108342	1.186123
F	-1.162200	-4.648442	-0.195267
F	0.672398	-4.943151	0.950870
C	-1.472478	1.189774	4.217753
F	-2.663063	0.772569	4.654260
F	-1.030122	2.174618	5.014306
F	-1.616332	1.682218	2.970534
C	-1.517959	2.476193	-2.352194
F	-0.889864	2.725121	-3.509825
F	-1.547045	1.142489	-2.157257
F	-2.775458	2.922626	-2.428844
H	-2.076002	-1.854947	-0.472997
C	-3.228320	-1.267558	1.181341
F	-3.326567	-0.384223	2.182411
F	-4.329250	-1.138846	0.406008
F	-3.198705	-2.503215	1.720080

TpF27Ag-Int-IIIfirst

Charge	0
Electronic Energy, BS1 (a.u.)	-4641.243910
Thermal and entropic correction, BS1 (a.u.)	0.271629
Electronic Energy, BS2 (a.u.)	-4644.493526

Number of Imaginary Frequencies	0
Imaginary frequencies (cm-1)	None

Molecular Geometry in Cartesian Coordinates

Ag	-0.117508	-0.288778	0.531781
C	-4.077873	0.622253	-0.071242
N	1.249490	1.669435	-0.032428
N	2.587861	1.487395	0.001606
N	1.928768	-1.360509	-0.162459
N	3.069076	-0.963304	0.446032
N	1.268960	0.061270	2.544556
N	2.494693	0.602544	2.359809
B	3.233028	0.479263	0.997213
C	0.962822	2.554149	-0.973665
C	2.146906	2.986500	-1.626418
C	3.176632	2.267471	-0.959205
C	2.033999	-2.642328	-0.474078
C	3.288142	-3.153920	-0.049375
C	3.925989	-2.028848	0.541292
C	0.792622	0.473948	3.708215
C	1.717628	1.344031	4.344722
C	2.805986	1.399260	3.431884
H	4.380904	0.729507	1.133390
C	3.933060	2.190949	3.717175
C	3.942363	2.901190	4.901488
C	1.750756	2.077443	5.541809
C	2.860987	2.847135	5.812958
C	4.520004	2.430028	-1.342754
C	4.796272	3.303453	-2.376097
C	2.453807	3.866076	-2.676861
C	3.773226	4.021181	-3.041905
C	5.213702	-2.150994	1.094036
C	5.825559	-3.387761	1.040913
C	3.929932	-4.402508	-0.086026
C	5.192585	-4.510135	0.454388
F	5.518359	1.755486	-0.756231
F	6.058022	3.483296	-2.780799
F	4.120037	4.844669	-4.039142
F	1.487260	4.533834	-3.320054
F	0.724437	2.044183	6.401320
F	2.940937	3.568460	6.938485
F	4.989765	3.673541	5.210156
F	4.973431	2.288676	2.877698
F	5.843471	-1.122866	1.679309
F	7.046246	-3.553068	1.561389
F	5.847347	-5.678154	0.448500
F	3.328948	-5.471602	-0.622946
C	0.875334	-3.353108	-1.097111
F	0.032109	-2.459422	-1.695653
F	1.298187	-4.243825	-2.031333
C	-0.464868	2.881116	-1.264567
F	-1.250487	2.562235	-0.185583
F	-0.624245	4.200703	-1.536986
C	-0.598325	0.106462	4.114431
F	-1.015619	-1.007424	3.444813
F	-0.672052	-0.136725	5.449166
C	0.021573	-4.139267	-0.073324
F	-0.492757	-3.287944	0.842437
F	-0.988666	-4.769608	-0.677764
F	0.783920	-5.037554	0.564875

C	-1.630838	1.220647	3.804739
F	-2.868339	0.830251	4.134907
F	-1.324628	2.331196	4.487598
F	-1.620068	1.516309	2.485977
C	-1.039559	2.094069	-2.466232
F	-0.371520	2.409252	-3.581712
F	-0.914407	0.768853	-2.246908
F	-2.339323	2.369640	-2.636447
H	-4.663758	0.567343	-0.993681
C	-3.405820	-0.715123	0.091150
F	-2.635581	-0.757603	1.228561
F	-4.269614	-1.739018	0.176376
F	-2.553175	-0.982837	-0.935463
C	-4.952211	0.975774	1.133138
H	-5.446645	1.936058	0.966674
H	-4.350964	1.053064	2.042015
H	-3.290324	1.362783	-0.224992
H	-5.723836	0.217283	1.293223

CH3CH2CF3

Charge	0
Electronic Energy, BS1 (a.u.)	-416.881477
Thermal and entropic correction, BS1 (a.u.)	0.051435
Electronic Energy, BS2 (a.u.)	-417.072022
Number of Imaginary Frequencies	0
Imaginary frequencies (cm-1)	None

Molecular Geometry in Cartesian Coordinates

C	3.645617	-1.780880	13.522446
H	4.518876	-1.219931	13.868936
F	3.147338	-3.871493	12.514578
C	4.149303	-3.110581	13.017920
F	4.737198	-3.833508	14.001910
F	5.065792	-2.955870	12.034389
C	2.607449	-1.939696	14.633616
H	1.739705	-2.502260	14.277538
H	2.263554	-0.960328	14.976650
H	3.230231	-1.249577	12.660766
H	3.031918	-2.472794	15.489151

N2

**Charge**	**0**
**Electronic Energy, BS1 (a.u.)**	**-109.515657**
**Thermal and entropic correction, BS1 (a.u.)**	**-0.012844**
**Electronic Energy, BS2 (a.u.)**	**-109.567645**
**Number of Imaginary Frequencies**	**0**
**Imaginary frequencies (cm-1)**	**None**

Molecular Geometry in Cartesian Coordinates

N	-4.052352	1.882824	0.191002
N	-4.052460	2.220713	1.243081

N2CHCF3

Charge	0
Electronic Energy, BS1 (a.u.)	-485.770434
Thermal and entropic correction, BS1 (a.u.)	0.006228
Electronic Energy, BS2 (a.u.)	-485.998138
Number of Imaginary Frequencies	0
Imaginary frequencies (cm-1)	None

Molecular Geometry in Cartesian Coordinates

```
C          -2.893461       -1.043491        0.402926
H          -2.471654       -1.751820       -0.295010
N          -3.099053        0.174031        0.007095
N          -3.271229        1.245747       -0.343047
C          -3.288880       -1.434584        1.780451
F          -3.705975       -0.364515        2.489946
F          -4.290977       -2.347504        1.790140
F          -2.260461       -2.007393        2.448416
```

THF

Charge	0
Electronic Energy, BS1 (a.u.)	-232.468713
Thermal and entropic correction, BS1 (a.u.)	0.088733
Electronic Energy, BS2 (a.u.)	-232.555879
Number of Imaginary Frequencies	0
Imaginary frequencies (cm-1)	None

Molecular Geometry in Cartesian Coordinates

```
O          -2.642218       -0.607441        0.908154
C          -3.520181       -0.557732        2.041683
H          -3.606987        0.482146        2.394147
H          -3.086106       -1.153775        2.853501
C          -4.875777       -1.086999        1.562612
H          -4.901687       -2.181165        1.623141
H          -5.714450       -0.692713        2.143330
C          -4.874696       -0.636146        0.094715
H          -5.140657        0.424861        0.023772
H          -5.560776       -1.203713       -0.540310
C          -3.404832       -0.844492       -0.283631
H          -3.059536       -0.160396       -1.068171
H          -3.231350       -1.874790       -0.632221
```

TpF27Ag-THF

Charge	0
Electronic Energy, BS1 (a.u.)	-4456.846783
Thermal and entropic correction, BS1 (a.u.)	0.311231
Electronic Energy, BS2 (a.u.)	-4459.997256
Number of Imaginary Frequencies	0

Imaginary frequencies (cm-1)	None

Molecular Geometry in Cartesian Coordinates

Ag	-0.416235	0.089559	0.879894
N	1.094039	1.841945	0.092040
N	2.420869	1.585545	0.056468
N	1.498207	-1.170070	-0.072518
N	2.712651	-0.898202	0.452546
N	1.258652	0.098436	2.725361
N	2.441926	0.669388	2.410824
B	3.053808	0.519590	0.990693
C	0.802520	2.707495	-0.866037
C	1.969973	3.049122	-1.599792
C	2.995712	2.297671	-0.963784
C	1.433806	-2.463530	-0.346884
C	2.643882	-3.111363	0.019077
C	3.442874	-2.055266	0.536967
C	0.955254	0.418771	3.974569
C	1.955361	1.258965	4.532163
C	2.899235	1.395657	3.478073
H	4.227456	0.666537	1.037268
C	4.050069	2.185639	3.648354
C	4.230398	2.810978	4.865993
C	2.160778	1.907864	5.760524
C	3.294959	2.674015	5.920093
C	4.322803	2.374020	-1.424232
C	4.587207	3.194300	-2.503094
C	2.265025	3.873406	-2.697936
C	3.568307	3.942587	-3.139477
C	4.736157	-2.314996	1.024188
C	5.197109	-3.615920	0.976156
C	3.135953	-4.425769	-0.018827
C	4.406477	-4.668680	0.456142
F	5.318464	1.671447	-0.866437
F	5.833158	3.291738	-2.980003
F	3.901426	4.710971	-4.184795
F	1.304436	4.573442	-3.315081
F	1.272114	1.798245	6.756727
F	3.535096	3.316776	7.070495
F	5.305221	3.580214	5.071971
F	4.947184	2.362021	2.667596
F	5.512084	-1.352830	1.543136
F	6.417926	-3.911351	1.436450
F	4.920515	-5.905589	0.446340
F	2.387809	-5.429261	-0.496313
C	0.167500	-3.043332	-0.887708
F	-0.601791	-2.060964	-1.447396
F	0.424337	-3.982022	-1.836896
C	-0.621261	3.078542	-1.129188
F	-1.394056	2.853340	-0.025654
F	-0.733678	4.387911	-1.474222
C	-0.322792	-0.065488	4.576401
F	-0.781991	-1.157021	3.892644
F	-0.143115	-0.416689	5.877683
C	-0.711768	-3.731113	0.187070
F	-0.978824	-2.876882	1.197311
F	-1.880310	-4.126071	-0.339986
F	-0.073706	-4.794302	0.689369
C	-1.461639	0.982242	4.553385
F	-2.587332	0.470500	5.066838
F	-1.104595	2.061301	5.260902

F	-1.716649	1.367313	3.284661
C	-1.247289	2.251990	-2.279700
F	-0.610370	2.507888	-3.429054
F	-1.134791	0.932618	-2.013484
F	-2.547690	2.539830	-2.423751
O	-2.695232	-0.320532	0.806913
C	-3.408117	-0.964089	1.898532
H	-3.942372	-0.192691	2.466443
H	-2.676277	-1.439513	2.550729
C	-4.363180	-1.939608	1.220996
H	-3.835845	-2.866497	0.972076
H	-5.225016	-2.183619	1.847214
C	-4.731605	-1.176763	-0.060432
H	-5.474534	-0.401571	0.156815
H	-5.127074	-1.820542	-0.850252
C	-3.395587	-0.546935	-0.449453
H	-3.486304	0.412136	-0.964500
H	-2.791853	-1.225798	-1.058959

TpF27Ag

Charge	0
Electronic Energy, BS1 (a.u.)	-4224.334047
Thermal and entropic correction, BS1 (a.u.)	0.199774
Electronic Energy, BS2 (a.u.)	-4227.409988
Number of Imaginary Frequencies	0
Imaginary frequencies (cm-1)	None

Molecular Geometry in Cartesian Coordinates

Ag	-0.249744	-0.066394	0.807277
N	1.110025	1.809769	0.061495
N	2.450403	1.629440	0.050644
N	1.712458	-1.209787	-0.065601
N	2.909868	-0.830969	0.436619
N	1.303046	0.123286	2.682540
N	2.491471	0.706121	2.406664
B	3.135289	0.602178	0.995547
C	0.785230	2.656002	-0.902592
C	1.942923	3.063384	-1.616145
C	2.999420	2.372931	-0.961788
C	1.768920	-2.492179	-0.385206
C	3.048601	-3.024164	-0.077167
C	3.755239	-1.910232	0.453108
C	0.945179	0.447304	3.914948
C	1.913331	1.301179	4.505012
C	2.898229	1.441504	3.489534
H	4.293521	0.829801	1.064084
C	4.032430	2.244871	3.705637
C	4.150330	2.882605	4.924823
C	2.053851	1.964583	5.734618
C	3.170320	2.745785	5.937664
C	4.326823	2.523228	-1.402933
C	4.559778	3.354078	-2.481036
C	2.205713	3.898147	-2.714436
C	3.509430	4.040962	-3.136239
C	5.085466	-2.054570	0.887260
C	5.668810	-3.301785	0.781177
C	3.662842	-4.283462	-0.169735

C	4.966788	-4.413230	0.255566
F	5.351721	1.878198	-0.829157
F	5.804450	3.520168	-2.940322
F	3.814276	4.822076	-4.179955
F	1.213782	4.534774	-3.349488
F	1.119601	1.854861	6.687471
F	3.350784	3.403099	7.089947
F	5.206688	3.664380	5.171253
F	4.973278	2.423302	2.767891
F	5.783934	-1.037544	1.410321
F	6.928412	-3.487573	1.189469
F	5.598187	-5.592127	0.192222
F	2.997009	-5.341464	-0.649266
C	0.542628	-3.187411	-0.882367
F	-0.350443	-2.282818	-1.390623
F	0.849174	-4.085741	-1.852233
C	-0.653415	2.957758	-1.172276
F	-1.415187	2.692043	-0.066492
F	-0.825424	4.259640	-1.514024
C	-0.376288	-0.005662	4.445479
F	-0.822458	-1.095422	3.749969
F	-0.288771	-0.333503	5.759738
C	-0.209337	-3.954263	0.231679
F	-0.631832	-3.080589	1.174970
F	-1.275318	-4.590644	-0.260278
F	0.606487	-4.843007	0.812413
C	-1.481354	1.069647	4.316088
F	-2.632020	0.641912	4.840603
F	-1.105506	2.196418	4.933264
F	-1.689088	1.348885	3.006554
C	-1.243828	2.101204	-2.318035
F	-0.538305	2.284175	-3.441203
F	-1.182062	0.791804	-1.981871
F	-2.521373	2.418010	-2.543737
